# Molecular Characterization of the Group A *Streptococcus* Virulence‐Regulatory System FasBCAX

**DOI:** 10.1111/mmi.70029

**Published:** 2025-10-18

**Authors:** Sushila Baral, Roshika Roshika, Clay P. Renshaw, Ameya Singh, Ashna Prabhu, Ira Jain, Rebekah Woolsey, David Quilici, Yftah Tal‐Gan, Paul Sumby

**Affiliations:** ^1^ Department of Microbiology & Immunology University of Nevada, Reno School of Medicine Reno Nevada USA; ^2^ Department of Chemistry University of Nevada, Reno Reno Nevada USA; ^3^ Nevada Proteomics Center University of Nevada, Reno Reno Nevada USA

**Keywords:** gene regulation, post‐transcriptional regulation, small regulatory RNA, virulence

## Abstract

By regulating the assortment and abundance of its virulence factors at different anatomic sites, the group A *Streptococcus* (GAS) can cause a range of human diseases. The Fas regulatory system is encoded by a four‐gene locus, *fasBCAX*, with *fasX* encoding the FasX small regulatory RNA effector molecule. FasX post‐transcriptionally regulates target mRNAs through well‐characterized mechanisms. Less characterized are the layers of regulation that occur upstream of FasX activity, such as how the products of the *fasBCA* genes enhance FasX abundance 100‐fold. Here, we present data consistent with FasBCA forming a three‐component regulatory system, with FasBC being sensor kinase‐like proteins that, upon recognizing one or more signals, heterodimerize and phosphorylate FasA, with phosphorylated FasA binding to the *fasX* promoter and inducing transcription. We identified key amino acids involved in phosphate flow, including H246 of FasC and D60 of FasA, and demonstrated that certain domains (e.g., the kinase domain of FasC) are dispensable for activity. Additionally, we show that a proteinaceous factor within human plasma activates the Fas system. This work represents the first molecular analysis of the Fas proteins which, by modulating FasX levels, play a critical role in the ability of GAS to coordinately regulate virulence factor production.

## Graphical abstract

The Fas system of the group A *Streptococcus* is comprised of three proteins (FasBCA) and a small regulatory RNA (FasX), and it represses adhesin production while also promoting the production of the spreading factor streptokinase. Here, we analyze the Fas proteins to assess how they positively regulate FasX production.

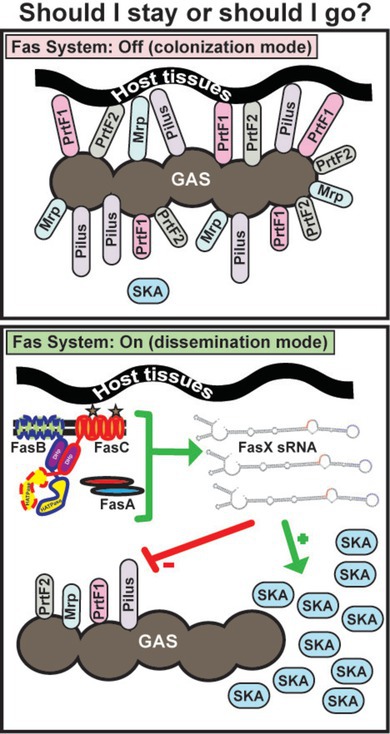

## 1 Introduction

The group A *Streptococcus* (GAS, *Streptococcus pyogenes* ) is a human‐specific bacterial pathogen that causes a wide variety of diseases, from self‐limiting pharyngitis to the severely invasive necrotizing fasciitis (Sims Sanyahumbi et al. 2016). Key to the ability of GAS to cause distinct diseases is the altered production, in assortment and abundance, of an array of virulence factors in response to environmental and metabolic cues (Woo et al. 2022; Kachroo et al. 2019; Velarde et al. 2014; Horstmann et al. 2011; Trevino et al. 2009; Cole et al. 2006). The regulation of virulence factor production is controlled at the transcriptional level by the combined efforts of 13 two‐component regulatory systems and more than 30 standalone transcriptional regulators (Schiavolin et al. 2024; Sarkar and Sumby 2017; Jimenez and Federle 2014; McIver 2009; Kreikemeyer et al. 2003). Additional levels of regulation include post‐transcriptional regulation via the activity of small regulatory RNAs (sRNAs) (Pappesch et al. 2017; Miller et al. 2014), and post‐translational regulation via the action of proteases (Carroll and Musser 2011; Lukomski et al. 1999).

**FIGURE 1:**
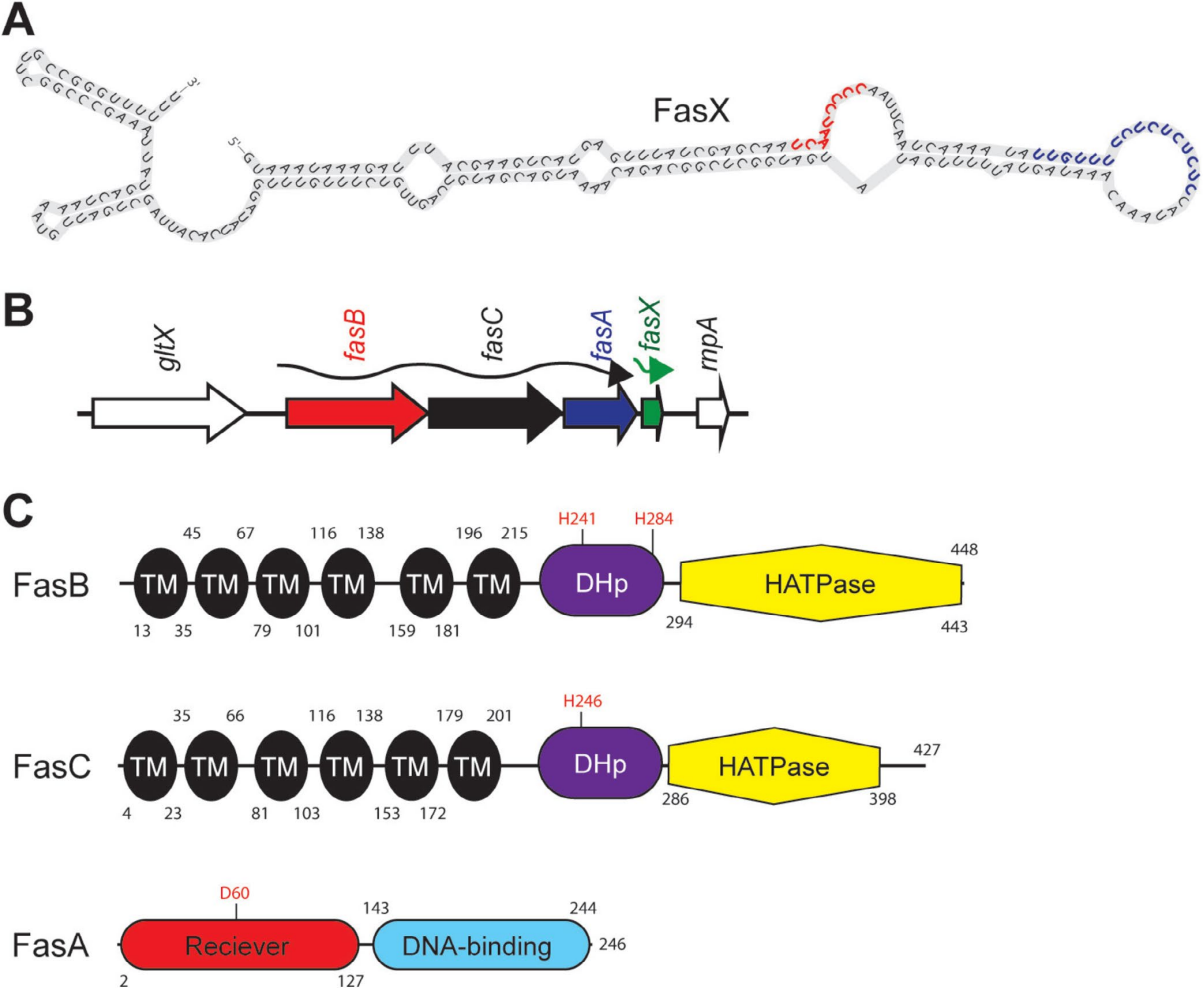
Overview of the Fas system. (A) Predicted secondary structure of the 205 nt FasX sRNA. FasX nucleotides involved in the positive regulation of *ska* mRNA stability (red), and in the negative regulation of pilus, *prtF1*, *prtF2*, and *mrp* mRNA translation (blue), are highlighted. (B) Schematic of the *fas* locus. Genes are represented by arrows that point in the direction of transcription. The *fasB* (red), *fasC* (black), and *fasA* (blue) genes are co‐transcribed, while *fasX* (green) is transcribed independently. Genes flanking the *fas* locus are shown in white. (C) Schematics of the FasB, FasC, and FasA proteins. FasB and FasC have six putative transmembrane domains (TM), a putative dimerization and histidine phosphotransfer domain (DHp), and a putative histidine kinase‐like catalytic domain (HATPase). FasA has putative receiver and DNA‐binding domains. Numbers refer to the amino acid number, starting with the N‐terminal methionine, in each of the proteins. The locations of putative phosphorylation sites in FasB (H241 or H284), FasC (H246), and FasA (D60) are shown.

The best characterized sRNA in GAS is the 205 nt sRNA FasX (Figure 1A) (Miller et al. 2014; Kreikemeyer et al. 2001). FasX positively enhances production of the thrombolytic factor streptokinase (SKA) by binding to, and therefore blocking exoribonuclease access to, the 5′ end of *ska* mRNA, increasing its stability (Ramirez‐Pena et al. 2010), the first described bacterial sRNA that functioned through such a mechanism (Condon and Bechhofer 2011). FasX also serves as a negative regulator, binding to and sequestering the ribosome‐binding sites, and therefore reducing translation of mRNAs encoding for the collagen‐binding pilus, the fibronectin‐binding proteins PrtF1 and PrtF2, and the fibrinogen‐binding M‐related protein (Mrp) (Danger et al. 2022; Danger, Makthal, et al. 2015; Liu et al. 2012). A reduction in the abundance of adhesins on the GAS cell surface, coupled with enhanced production of the “spreading” factor streptokinase, which indirectly degrades blood clots and tissue barriers (Peetermans et al. 2016), have led to the hypothesis that FasX functions at the interphase between GAS colonization and dissemination. FasX promotes virulence, enhancing both GAS lethality in a mouse model of bacteremia infection and the ability of GAS to survive and replicate in human blood (Danger et al. 2022; Danger, Cao, et al. 2015). The importance of the Fas system is also highlighted by the fact that isolates of all tested GAS serotypes, with the exception of serotype M3 isolates (Cao et al. 2014), harbor a functional system.

FasX is produced as a monocistronic transcript while the upstream genes, *fasBCA*, form an operon (Figure 1B) (Ramirez‐Pena et al. 2010). Natural or engineered mutations within *fasBCA* result in a ~100‐fold reduction in FasX abundance (Cao et al. 2014; Kreikemeyer et al. 2001), consistent with the products of the *fasBCA* genes forming a regulatory system that enhances *fasX* transcription. The Fas proteins share amino acid similarity with proteins from bacterial two‐component regulatory systems (Jacob‐Dubuisson et al. 2018). Two‐component systems consist of a membrane‐spanning sensor kinase protein and a cytoplasmic response regulator protein. Commonly, the sensor kinase, which functions as a homodimer, binds to an extracellular signalling molecule resulting in the autophosphorylation of a conserved histidine residue within each monomer. Subsequently, these phosphates are transferred to aspartate residues within the receiver domains of two response regulator monomers, leading to response regulator dimerization and activation of DNA binding. Response regulator binding to the promoter regions of genes can positively or negatively regulate their expression, linking changes in the extracellular environment to the regulation of gene expression (Buschiazzo and Trajtenberg 2019). FasB and FasC share sequence similarities to sensor kinases while FasA shares sequence similarity to response regulators (Kreikemeyer et al. 2001). The presence of two sensor kinase‐like proteins indicates that the mechanics of the Fas system, from signal input to regulatory output, may differ relative to that of standard two‐component systems.

Here, we initiated an investigation of how FasBCA collectively modifies the abundance of FasX in response to extracellular signals. We identified that (*i*) the transmembrane domains of FasB and the kinase domain of FasC are dispensable for regulatory activity; (ii) that FasBC forms a heterodimer or higher form of arrangement; (iii) that H246 within FasC is likely a site of autophosphorylation by the activated FasBC complex; (iv) that phosphorylation of FasA on amino acid D60 is required for regulatory activity; and (v) that a protein‐based factor within human plasma activates Fas system activity. The important virulence‐regulating role of the Fas system, coupled with its non‐standard make‐up, makes this research a valuable addition to the literature concerning the mechanisms by which bacterial pathogens modify gene expression in response to environmental cues.

## 2 Results

### 2.1 The FasBCA Proteins Share Similarities With Two‐Component System Proteins

The FasBCA protein sequences were subjected to computational analyses using a variety of domain analysis software such as InterPro (Paysan‐Lafosse et al. 2023), the Simple Modular Architecture Research Tool (SMART) (Letunic et al. 2021), and the Conserved Domain Database (CDD) (Wang et al. 2023). While there were some minor disagreements about the boundaries of individual domains, the results were largely overlapping and predicted six transmembrane domains, a dimerization and histidine phosphotransfer (DHp) domain, and a histidine kinase‐like catalytic domain (HATPase) for both FasB and FasC (Figure 1C). These domains are common features of the membrane‐spanning sensor kinase component of two‐component regulatory systems (Buschiazzo and Trajtenberg 2019). Analysis of FasA predicted the presence of receiver and DNA‐binding domains (Figure 1C), which are common features of the cytoplasmically located response regulator of two‐component regulatory systems. Along with a previous study (Kreikemeyer et al. 2001), the data are consistent with FasB and FasC being membrane‐spanning proteins that harbor kinase activity, and with FasA being a cytoplasmically located transcriptional regulator.

### 2.2 An N‐Terminally Truncated FasB Consisting of Only the DHp and HATPase Domains Is Sufficient to Complement a *fasB* Mutant GAS Strain

**FIGURE 2:**
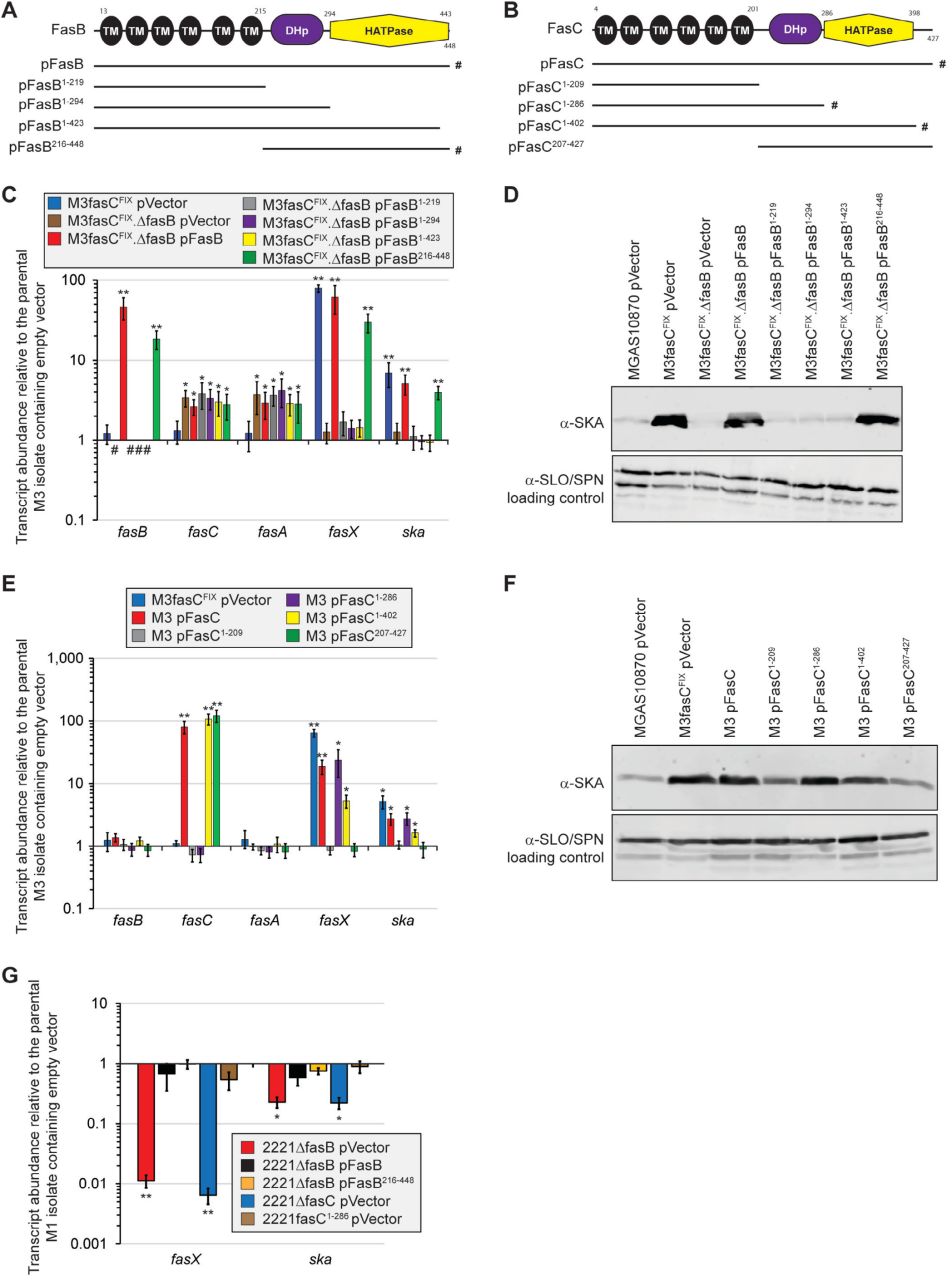
The putative transmembrane domains of FasB and the HATPase domain of FasC are dispensable for regulatory activity. (A) Schematic of the putative domains within FasB and the extent of FasB truncations within a series of constructed plasmids. The hashtags highlight those plasmids capable of complementing a *fasB* mutant strain (see panels C and D below). (B) Schematic of the putative domains within FasC and the extent of FasC truncations within a series of constructed plasmids. The hashtags highlight those plasmids capable of complementing a *fasC* mutant strain (see panels E and F below). (C) Taqman‐based quantitative RT‐PCR analysis to identify which domains of FasB are required for complementation. The plasmids from panel A were individually introduced into the *fasB* mutant GAS strain M3fasC^FIX^.ΔfasB and compared to the parental M3 isolate containing an empty vector, plus additional strains. The abundances of mRNAs from each of the four *fas* genes (*fasBCAX*) were tested, as was the Fas‐regulated mRNA encoding for streptokinase (*ska*). Shown are the averages (±standard deviations) of a minimum of triplicate samples run in triplicate. The asterisks highlight statistical significance relative to the parental M3 isolate containing an empty vector (ANOVA with the Tukey multiple comparisons test; *, *p* < 0.01; **, *p* < 0.001). Hashtags highlight the lack of signal due to the absence of *fasB* Taqman primer or probe binding sites within those strains. (D) Western blot analyses of secreted protein fractions recovered from exponential phase cultures of the same strains used in panel C. The virulence factors streptolysin O (SLO; upper band) and *S. pyogenes* NAD^+^ glycohydrolase (SPN; lower band) are not altered in abundance by the Fas system and hence served as loading controls. The tested protein, which is impacted by Fas system status, is streptokinase (SKA). (E) Taqman‐based analysis to identify which domains of FasC are required for complementation. The plasmids from panel B were individually introduced into a serotype M3 isolate (which is naturally a *fasC* mutant) and compared to the parental isolate containing an empty vector, plus additional strains. Shown are the averages (±standard deviations) of a minimum of triplicate samples run in triplicate. The asterisks highlight statistical significance relative to the parental M3 isolate containing an empty vector (ANOVA with the Tukey multiple comparisons test; *, *p* < 0.01; **, *p* < 0.001). (F) Western blot analyses of secreted protein fractions recovered from exponential phase cultures of the same strains used in panel E. (G) Taqman‐based assessment of the ability of the truncated FasB and FasC proteins to function in a serotype M1 background. The asterisks highlight statistical significance relative to the parental M1 isolate containing an empty vector (ANOVA with the Tukey multiple comparisons test; *, *p* < 0.01; **, *p* < 0.001).

To investigate which domains of FasB are required for regulatory activity, we pursued a complementation‐based approach. Five plasmids, one containing the full‐length *fasB* gene and four containing truncated derivatives (Figure 2A), were created and separately transformed into a *fasB* deletion mutant derivative of GAS strain M3fasC^FIX^ (see Table S1). M3fasC^FIX^ is a serotype M3 GAS strain that harbors a functional Fas system as the natural *fasC* mutation that is present in M3 isolates has been fixed (Cao et al. 2014). The ability of the plasmids to complement our *fasB* deletion mutant strain, M3fasC^FIX^.ΔfasB, were tested by qRT‐PCR and Western blot analyses. Relative to the parental M3 isolate MGAS10870, which naturally lacks Fas system activity, all our plasmid‐containing M3fasC^FIX^.ΔfasB derivatives had ~3‐fold higher levels of expression of the *fasC* and *fasA* genes (Figure 2C). We believe this higher *fasC* and *fasA* expression is a consequence of read‐through transcription from the upstream spectinomycin resistance cassette that was used to replace *fasB*. Turning our attention to the ability of our plasmids to complement the *fasB* mutation, only plasmids pFasB (red bars in Figure 2C) and pFasB^216‐448^ (green bars) resulted in high‐level FasX sRNA and *ska* mRNA abundances, our readouts for a functioning Fas system. The complementing activities of pFasB and pFasB^216‐448^ were confirmed by SKA Western blot using secreted protein fractions (Figure 2D). As it is possible that the inability of the three C‐terminally truncated FasB plasmids to complement could be due to the lack of protein expression, we tested this in our strain containing pFasB^1‐423^. Western blot analyses involving cytoplasmic and membrane protein GAS fractions identified equivalent FasB levels from the non‐complementing pFasB^1‐423^ and the complementing pFasB^216‐448^ (Figure S1), identifying that the lack of complementation by pFasB^1‐423^ is not a consequence of poor expression. Overall, these data are consistent with, at least when overexpressed from a plasmid, the putative transmembrane domains of FasB being dispensable for regulatory activity.

### 2.3 A C‐Terminally Truncated FasC Consisting of Only the Transmembrane and DHp Domains Is Sufficient to Complement a *fasC* Mutant GAS Strain

To investigate the domains of FasC required for regulatory activity, we used an approach like that described above for FasB. Five plasmids, one containing the full‐length *fasC* gene and four containing truncated derivatives (Figure 2B), were created and separately transformed into the natural *fasC* mutant serotype M3 strain MGAS10870. The ability of the plasmids to complement were tested by qRT‐PCR and Western blot analyses. Relative to the empty vector, plasmids producing either the full‐length FasC protein (red bars in Figure 2E) or the truncated versions FasC^1‐286^ (purple bars) or FasC^1‐402^ (yellow bars) were able to enhance the abundance of FasX sRNA and *ska* mRNA. The complementing activities of pFasC, FasC^1‐286^, and FasC^1‐402^ were confirmed by SKA Western blot using secreted protein fractions (Figure 2F). While FasC^1‐286^ fully complemented the mutant strain, FasC^1‐402^ did so only partially, an observation we have not further investigated but could be due to structural issues associated with the removal of the C‐terminal 25 amino acids. It is also possible that the FasC^1‐402^ protein, and the non‐complementing FasC^1‐209^ and FasC^207‐427^ proteins, are only poorly expressed, something that we could not test due to a lack of FasC antibodies. Regardless, at least when overexpressed from a plasmid, we have shown that the putative HATPase domain of FasC is dispensable for regulatory activity.

### 2.4 FasB216 ^−448^ and FasC^1^ ^−286^ Are Also Functional in a Serotype M1 GAS Background

To confirm that truncated versions of the FasB and FasC proteins retain regulatory activity, we introduced the respective genes into a second strain background, that of the serotype M1 strain MGAS2221. Initially, we aimed to create MGAS2221 derivatives in which we replaced the wild‐type chromosomal *fasB* or *fasC* alleles with the corresponding shortened alleles. We were successful with this approach with *fasC*, creating strain 2221fasC^1‐286^ and confirming that this strain retains a functioning Fas system (Figure 2G). Additional data that this strain provided was that overexpression of the truncated FasC^1‐286^ was not required for activity, as the gene was present in single copy at the natural locus in the chromosome. Unfortunately, we were unsuccessful in allelic replacement experiments aimed at swapping out the wild‐type *fasB* gene for the allele expressing FasB^216‐448^. Thus, instead, we used a plasmid‐based approach like that performed in the M3 background, and like the M3‐generated data, we identified that plasmid pFasB^216‐448^ could complement a *fasB* GAS mutant, this time in the M1 background (Figure 2G). The data further support the putative DHp and HATPase domains of FasB, and the putative transmembrane and DHp domains of FasC, being sufficient for regulatory activity. As we gained similar data from both our M1 and M3 background strains, we decided, for subsequent experiments, to work exclusively with M1 GAS strains.

### 2.5 Histidine 246 in the Putative DHp Domain of FasC Is Required for Activity

**FIGURE 3:**
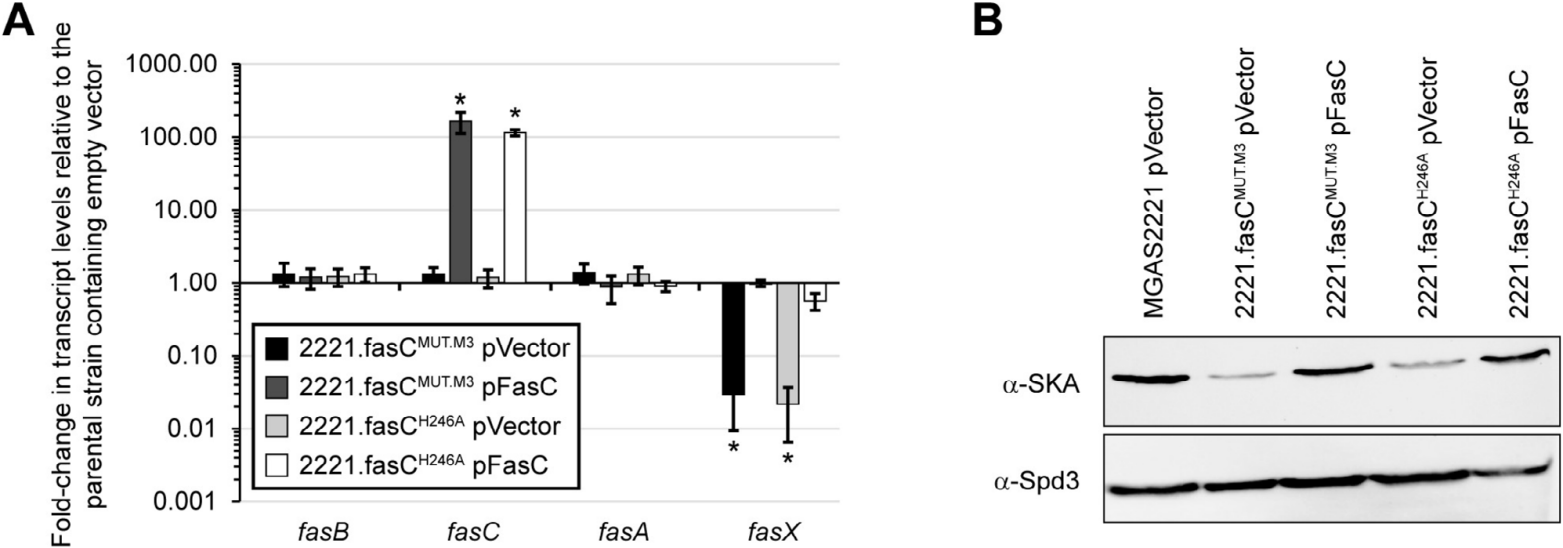
Swapping the putative autophosphorylation histidine in FasC to an alanine disrupts regulatory activity. (A) Taqman‐based analysis of *fasB*, *fasC*, *fasA*, and *fasX* RNA levels in a series of *fasC* mutant GAS strains. Statistical significance was investigated via ANOVA with the Tukey multiple comparisons test (asterisks highlight significance relative to the parental isolate containing the empty vector; *, *p* < 0.0001). (B) Western blot analyses of secreted protein fractions recovered from exponential phase cultures of the same strains used in panel A. The secreted DNase Spd3 is not altered in abundance by the Fas system and hence serves as a loading control.

In a typical bacterial two‐component system, the HATPase domain of the sensor kinase autophosphorylates a histidine residue within the DHp domain. Subsequently, this phosphate is transferred to an aspartate residue of the response regulator protein, activating its DNA‐binding activity (Francis and Porter 2019). The putative DHp domains of FasB and FasC harbor two (H241 and H284) and one (H246) histidine residues, respectively (Figure 1C). To investigate whether these histidine residues are required for regulatory activity, we set out to perform allelic exchange, creating GAS derivatives in which one of the histidines was changed to the non‐phospho‐accepting amino acid alanine. For *fasC*, we were successful in creating 2221.fasC^H246A^, a derivative of the parental M1 strain MGAS2221 containing a chromosomal *fasC* allele that produces FasC with a H246A change. Assessment of Fas system regulatory activity in strain 2221.fasC^H246A^, both in the presence and absence of the *fasC* complementation plasmid pFasC, was performed by Taqman and Western blot analyses. 2221.fasC^H246A^ containing empty vector, but not when containing pFasC, produced the FasX sRNA and SKA protein in reduced abundance, like that of the *fasC* mutant strain 2221.fasC^MUT.M3^ (Figure 3). Thus, the data are consistent with H246 having critical importance for FasC regulatory activity, and we hypothesize that this is a consequence of this residue having a key role in the phospho‐transfer associated with the Fas system.

Next, we turned our attention to *fasB*. Unfortunately, we were unable to generate any derivatives, via allelic exchange, that produced FasB harboring H to A substitutions. Since we could not test the functional roles of H241 and H284 from a chromosomally encoded *fasB* gene, we set out to create derivatives of pFasB that produced FasB with H241A or H284A substitutions. We were successful in creating pFasB^H241A^ but not pFasB^H284A^. Testing the ability of pFasB^H241A^ to complement our *fasB* mutant strain 2221ΔfasB identified that it complemented just as efficiently as the original pFasB plasmid (Figure S2). Thus, at least when expressed from a plasmid, H241 is not required for FasB activity, a finding that is consistent with H284 being the site of phosphorylation within FasB, but this awaits future experimental confirmation.

### 2.6 Evidence for Heterodimerization, Not Homodimerization, by FasB and FasC

**FIGURE 4:**
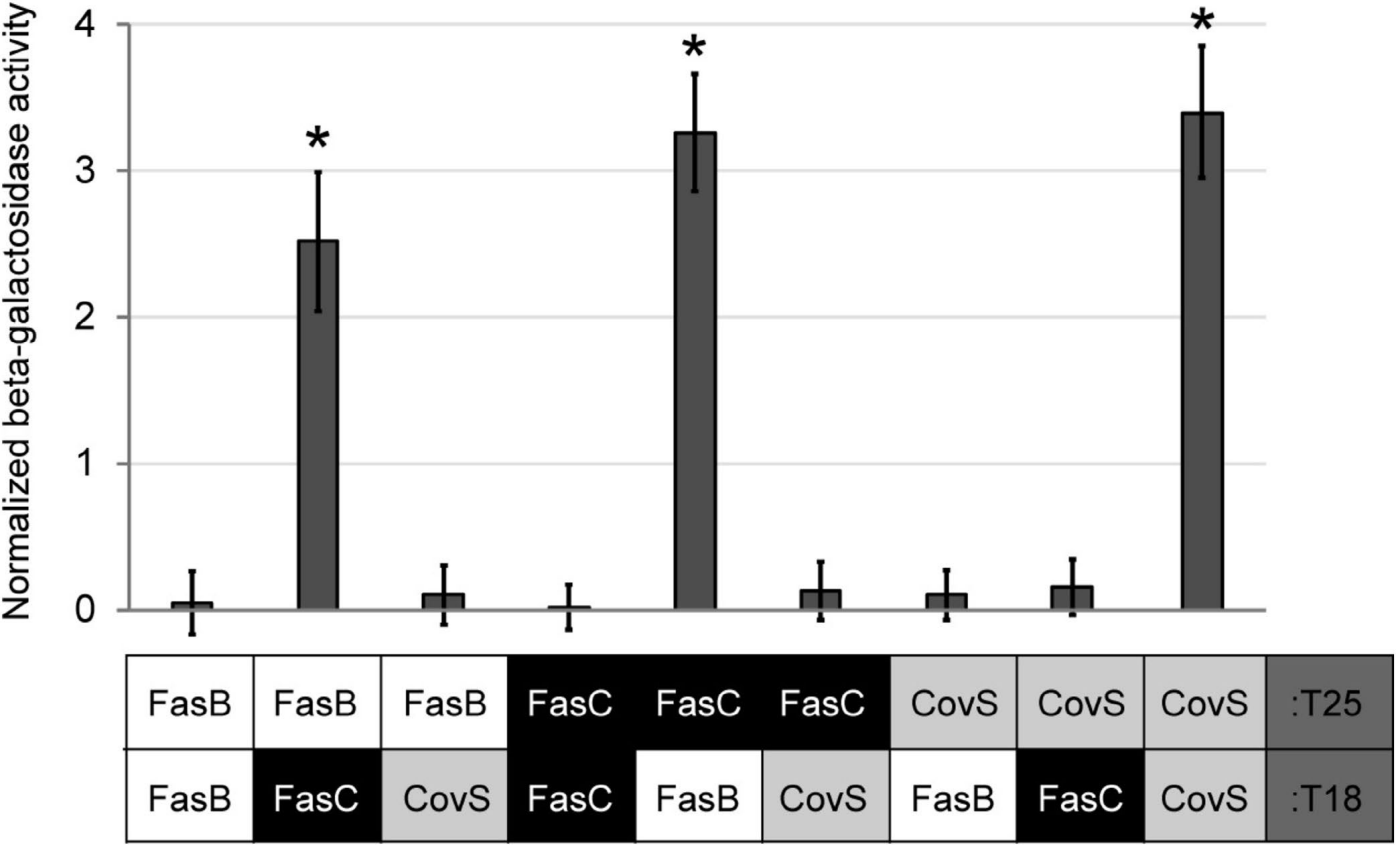
A bacterial two‐hybrid approach indicates that FasB and FasC form heterodimers. FasB, FasC, and CovS (control) fusion proteins were constructed with C‐terminal T18 or T25 fragments of adenylate cyclase. T18‐ and T25‐based plasmids were co‐transformed into *E. coli* and the activity of the β‐galactosidase cAMP‐dependent reporter gene was determined. Shown are the means (± standard deviation) calculated from three independent cultures run in triplicate. The asterisks (*) highlight statistical significance relative to a −ve control strain (one‐way ANOVA followed by the Tukey multiple comparisons test; *, *p* < 0.001).

The sensor kinase component of two‐component systems typically functions as a homodimer, with autophosphorylation occurring in a *cis* (each monomer phosphorylates itself) or *trans* (each monomer phosphorylates its partner monomer within the homodimer) manner, depending on the sensor kinase (Kansari et al. 2024). As the Fas system encodes two sensor kinase‐like proteins, FasB and FasC, this raises questions about the oligomerization of these proteins. To investigate this, we used an *E. coli* ‐based bacterial two‐hybrid system in which test proteins were fused with fragments T18 or T25 of adenylate cyclase (CyaA) (Battesti and Bouveret 2012; Karimova et al. 1998), such that if the test proteins interact, they bring the T18 and T25 fragments together, creating a functional protein, which is detected via β‐galactosidase assays. We successfully used this system previously to identify homodimer formation by CovS, the sensor kinase component of the CovRS two‐component regulatory system in GAS (Jain et al. 2020). Plasmids expressing C‐terminally T25‐tagged FasB, FasC, or CovS proteins, and similar plasmids expressing C‐terminally T18‐tagged FasB, FasC, or CovS proteins, were constructed and transformed in pairs into the *E. coli* strain BTH101. Consistent with previous data (Jain et al. 2020), and serving as a control in these studies, the ability of the CovS sensor kinase to form a homodimer was evident by high β‐galactosidase activity when the T25‐ and T18‐tagged CovS proteins were present in the same cell (Figure 4). In contrast, when T25‐ and T18‐tagged FasB proteins were present in the same cell, there was no appreciable β‐galactosidase activity, consistent with FasB not being able to form a homodimer. Identical results were obtained with T25‐ and T18‐tagged FasC. However, robust β‐galactosidase activity was observed for *E. coli* containing both FasB and FasC, but not when either one was present with CovS (Figure 4). These data are consistent with FasB and FasC interacting in a specific manner, with them forming a heterodimer or higher‐order oligomer.

### 2.7 FasA Is a Member of the ComE‐Like Family of Transcriptional Regulators and Requires an Aspartate Residue at Amino Acid 60 for Regulatory Activity

**FIGURE 5:**
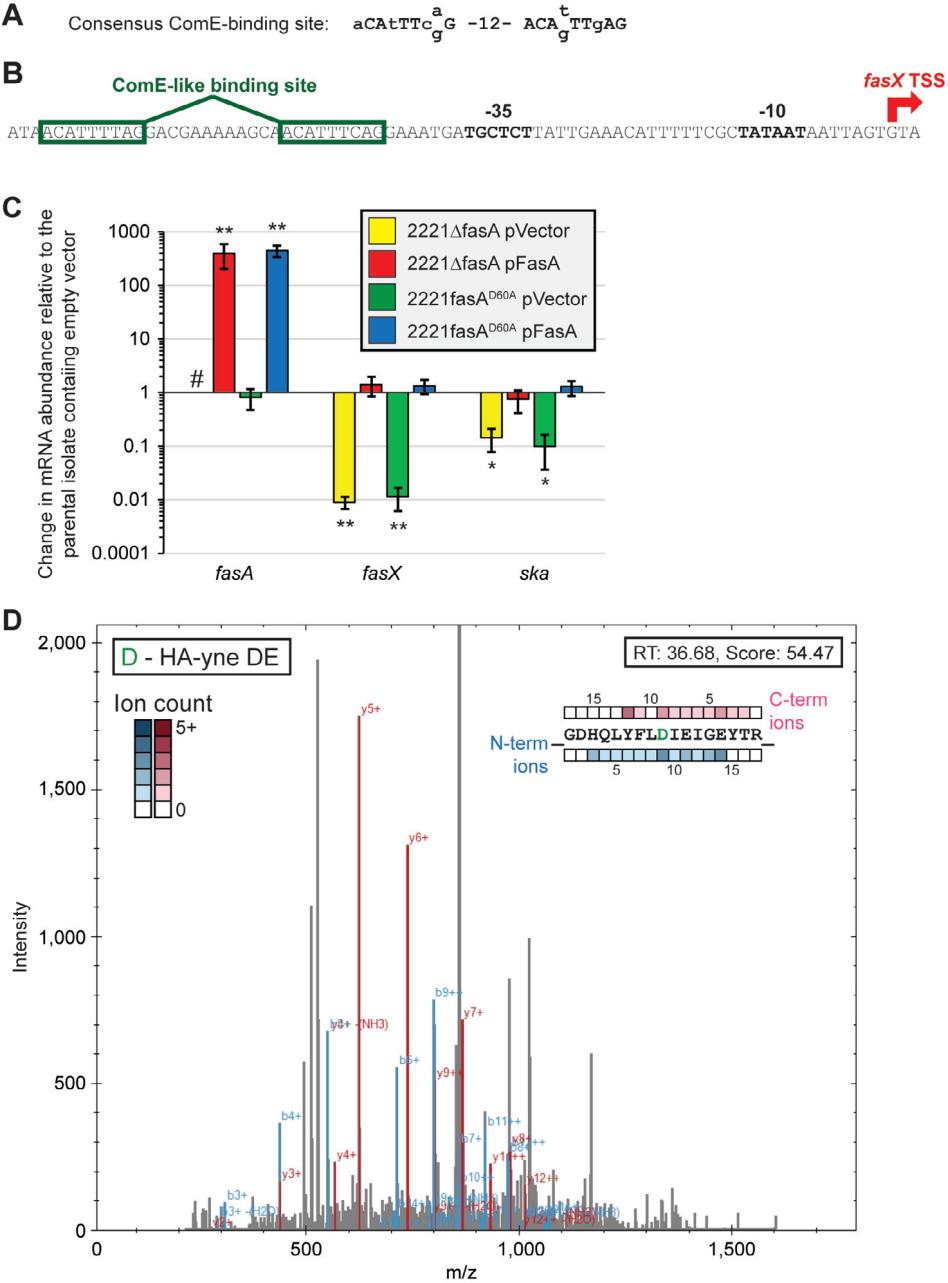
FasA is a ComE‐like transcriptional regulator that is phosphorylated on amino acid D60. (A) Schematic of the consensus binding site for members of the ComE‐like family of transcriptional regulators. (B) Schematic of the *fasX* promoter region showing the location of a putative binding site for a FasA dimer (green boxes). The −10 and −35 promoter sequences of the *fasX* promoter are labeled and in bold. The *fasX* transcriptional start site (TSS) is represented with a red arrow. (C) Taqman‐based analysis identifying that the putatively phosphorylated aspartate within FasA, D60, is required for regulatory activity. Triplicate THY broth cultures of each strain were tested in triplicate with mean values (± standard deviation) shown. Statistical significance was investigated via ANOVA with the Tukey multiple comparisons test (asterisks highlight significance relative to the parental isolate containing empty vector); (*, *p* < 0.01; **, *p* < 0.001). The hashtag highlights the lack of any signal for strain 2221ΔfasA containing empty vector due to the absence of binding sites for the *fasA* Taqman primers and probe. (D) Phosphorylation of D60 in FasA was confirmed by LC–MS/MS analysis. Shown is the annotated MS2 spectrum of the HA‐yne‐modified FasA peptide.

Bioinformatic analysis of FasA identifies it as belonging to the ComE‐like family of transcriptional regulators (data not shown). ComE family proteins bind to a conserved DNA sequence to transcriptionally regulate target genes (Figure 5A). We identified a match to this conserved sequence upstream of the −35 region of the *fasX* promoter (Figure 5B). Given that the FasBCA proteins function together to promote the abundance of FasX transcripts, we propose that a phosphorylated form of FasA binds to the consensus sequence upstream of the *fasX* promoter and enhances *fasX* transcription.

The DNA‐binding activity of ComE family members is activated following phosphate transfer from a histidine residue within the DHp domain of a partner sensor kinase protein to a conserved aspartate residue within the response regulator (Lewis et al. 1999). Alignment of FasA with other ComE family members identified the aspartate at amino acid 60 (D60) as the putative site of activating phosphorylation. We investigated this by allelic replacement, replacing the wild‐type *fasA* allele in MGAS2221 with a derivative harboring a non‐synonymous mutation resulting in a D60A change. Substituting alanine for aspartate is a common method to investigate putative response regulator phosphorylation sites (Garber et al. 2022; Gusa et al. 2006), as the uncharged amino acid alanine cannot be phosphorylated and hence the response regulator is locked into an “off” position. Derivatives of the created strain, 2221fasA^D60A^, were created by transforming with an empty vector or the *fasA* complementation plasmid pFasA, and the strains were compared with a *fasA* deletion mutant strain, 2221ΔfasA, and its plasmid‐complemented derivative. The D60A change in FasA abolished regulatory activity, as evident by this strain having similar FasX and *ska* mRNA levels as the *fasA* deletion mutant strain (Figure 5C). Both 2221fasA^D60A^ and 2221ΔfasA could be complemented by plasmid pFasA. The data are consistent with FasA being activated by phosphorylation on residue D60.

### 2.8 FasA Is Phosphorylated at D60

A major impediment to the study of phosphate flow between two‐component system proteins, and hence of the mechanics of two‐component system activity, is that phosphorylation of the key aspartate residue within the response regulator is transient and hydrolytically unstable (Allihn et al. 2021). This means that analytical methods commonly used to detect other phosphorylated amino acids (e.g., serine and tyrosine), such as immobilized metal affinity chromatography (IMAC) followed by liquid chromatography tandem mass spectrometry (LC‐MS/MS), are of limited value. Several research groups have risen to this challenge by creating chemical approaches that modify phosphorylated aspartates into stable forms, forms that subsequently can be detected using standard proteomic workflows (Allihn et al. 2021; Chang et al. 2018). We utilized one of these chemical proteomic strategies to investigate the potential phosphorylation of D60 in FasA. Protein fractions from GAS strain MGAS2221 were treated with a hydroxylamine alkyne probe (HA‐yne) that reacts with phosphorylated aspartate residues, converting them into a stable form. Desthiobiotin azide was clicked to the alkyne functional group of HA‐yne, the samples were trypsin digested, and affinity enrichment of the modified peptides was performed using streptavidin agarose beads. Enriched samples were subjected to LC–MS/MS, which confirmed the presence of the HA‐yne modification on D60 within FasA (Figure 5D), and hence that this amino acid is a site of phosphorylation. While not a focus of this study, we note and report that additional sites of aspartate phosphorylation were identified across the MGAS2221 proteome (Table S3).

### 2.9 Fas System Regulatory Activity Is Enhanced Following GAS Exposure to Human Plasma

**FIGURE 6:**
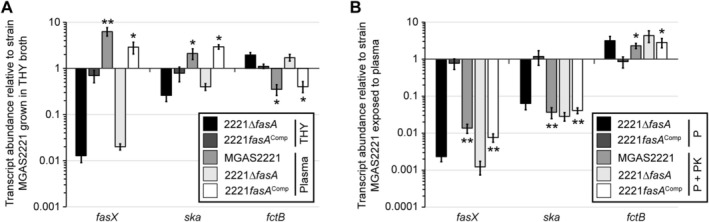
A proteinaceous factor within human plasma enhances the activity of the Fas system. Relative *fasX*, *ska*, and *fctB* transcript levels were compared between parental, *fasA* mutant, and complemented mutant GAS strains by Taqman analysis. Triplicate cultures of each strain were tested in triplicate, with mean values (± standard deviation) shown. (A) GAS strains were grown in THY broth and compared following exposure to plasma. Statistical significance was investigated via ANOVA with the Tukey multiple comparisons test (asterisks highlight significance of plasma samples relative to their THY broth grown counterparts (**, *p* < 0.0001; *, *p* < 0.01)). (B) GAS strains were exposed to human plasma (P) or to human plasma that had been pretreated with proteinase K (*P* + PK). Statistical significance was investigated via ANOVA with the Tukey multiple comparisons test (asterisks highlight significance of proteinase K‐treated plasma samples relative to their untreated counterparts (**, *p* < 0.0001; *, *p* < 0.01)).

Given that the Fas system enhances the production of the thrombolytic agent streptokinase while reducing the production of cell surface adhesins, we hypothesized that the Fas system is activated in the presence of human blood/plasma. To investigate this, we grew wild‐type (MGAS2221), *fasA* mutant (2221Δ*fasA*), and *fasA* complemented mutant (2221*fasA* ^Comp^) GAS strains in THY broth before exposing them to human plasma for 15 min. Pre‐ and post‐plasma exposure samples were collected, total RNA isolated, and the RNA used in Taqman‐based quantitative RT‐PCR analysis. Relative to the THY samples, the wild‐type and complemented mutant strains generated a greater abundance of FasX sRNA following plasma exposure (Figure 6A). This plasma‐enhancing effect on FasX abundance was not observed for the *fasA* mutant strain, consistent with the requirement of a functioning Fas system. The downstream regulatory consequences of enhanced FasX production on *ska* and *fctB* (pilus) mRNA levels following plasma exposure are as predicted, with increased *ska* and decreased *fctB* mRNA abundances (Figure 6A).

### 2.10 A Proteinaceous Factor Within Human Plasma Contributes to the Plasma‐Induced Enhancement of Fas System Regulatory Activity

If the factor within human plasma responsible for increasing Fas system activity is protein‐based, we reasoned we could identify this by comparing untreated and proteinase K‐treated plasma samples. When using the same strains as in Figure 6A, the wild‐type and complemented strains produced FasX at approximately 100‐fold lower levels when exposed to proteinase K‐treated plasma relative to untreated plasma (Figure 6B). Fitting with reduced FasX abundance, proteinase K‐treated samples have approximately 25‐fold lower levels of *ska* mRNA abundance and 2.5‐fold higher levels of *fctB* mRNA abundance, relative to non‐proteinase K‐treated samples (Figure 6B). The data are consistent with a protein‐based factor within human plasma being an activator of the Fas regulatory system.

## 3 Discussion

**FIGURE 7:**
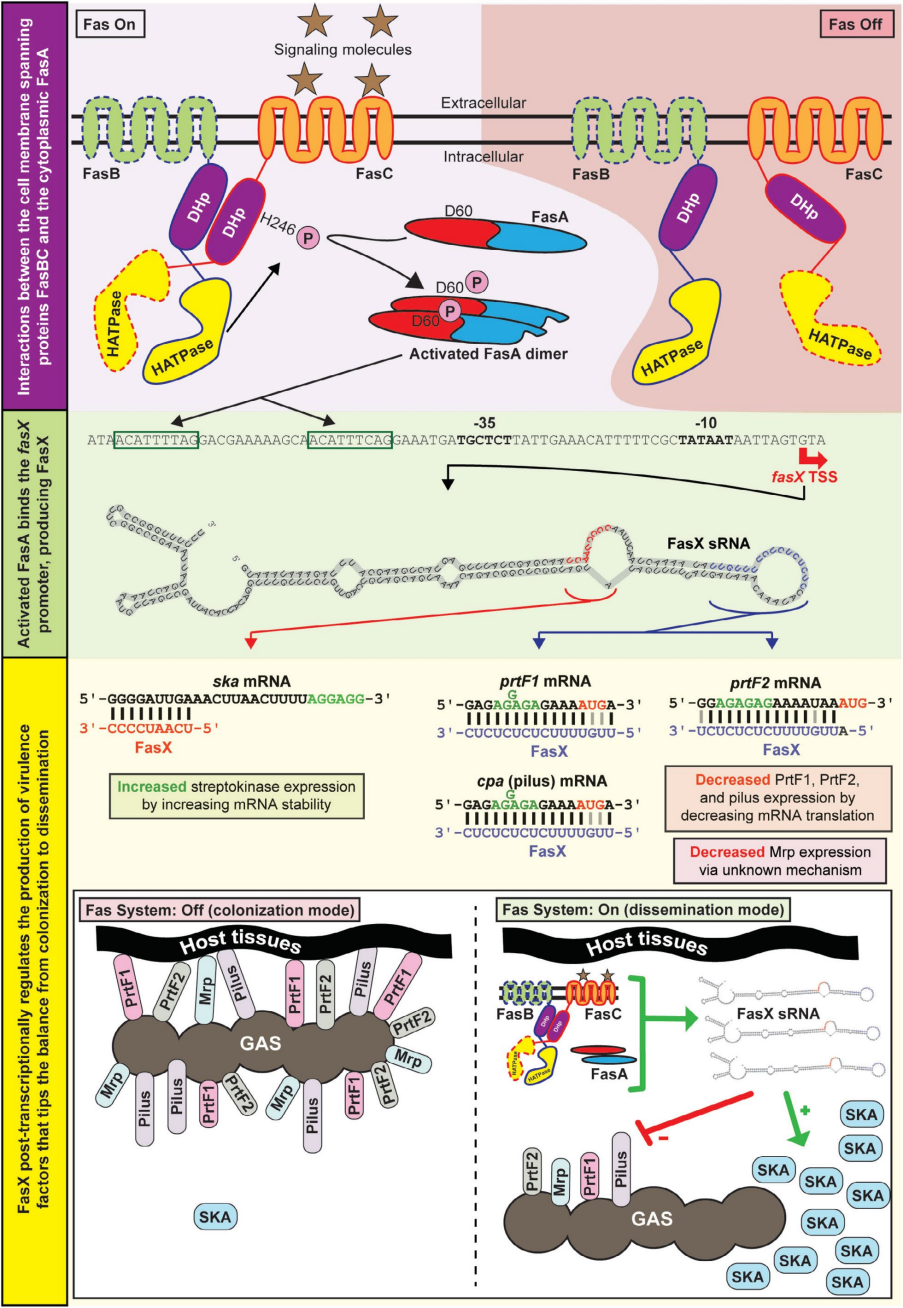
Model of Fas system activity. The Fas system is activated by external signaling molecules (represented by brown stars). FasB and FasC domains that are dispensable for regulatory activity are highlighted by dashed lines surrounding these domains. Activation of the Fas system, via signal molecule binding by the N‐terminous of FasC, leads to the generation and transfer of phosphate groups, ultimately resulting in activation of FasA. Activated FasA binds the ComE‐like consensus sequence (green rectangles) and promotes the significant upregulation of *fasX* transcription, giving high levels of FasX sRNA. FasX positively regulates the abundance of SKA protein by binding to, and increasing the stability of, *ska* mRNA. FasX negatively regulates the abundance of the adhesins PrtF1, PrtF2, Mrp, and pilus by binding to their respective mRNAs and inhibiting their translation (and in some instances, also negatively regulating mRNA stability). The reduced production of adhesins result in detachment of GAS from the host, while the enhanced production of SKA promotes the degradation of blood clots and tissue barriers. Together, these consequences of FasX activity switch GAS from a colonization mode to a dissemination mode.

We used the data generated here and previously to create a model of Fas system activity (Figure 7). In this model, while both FasB and FasC are anchored in the GAS cell membrane via their transmembrane domains, these domains are dispensable for the regulatory activity of FasB (Figure 2C,D). This is consistent with the N‐terminal half of FasC having the sensing activity of the Fas system that binds to appropriate signaling molecules. Upon signal molecule binding by FasC, this leads to FasB and FasC heterodimerization via their DHp domains, which subsequently activates the kinase activity of the FasBC dimer. As the kinase domain of FasC is dispensable for regulatory activity (Figure 2E,F), this is consistent with the FasB kinase domain being both necessary and sufficient for Fas system activity (we propose that FasC is actually a pseudokinase). In our model, the activated FasB kinase domain phosphorylates histidine 246 (H246) within the DHp domain of FasC, although we have not ruled out the possibility that phosphorylation could also occur *in cis* on H284 within the FasB DHp domain (Casino et al. 2014). Subsequently, phosphotransfer occurs from FasBC to aspartate 60 (D60) within FasA (Figure 5D), which we propose promotes dimer formation and activation of DNA‐binding activity. Activated FasA binds to the ComE‐like consensus sequence located upstream of *fasX* and promotes transcription (Figure 5B). Unpublished expression microarray data is consistent with the transcriptome of a *fasA* mutant GAS strain essentially overlapping that of a *fasX* mutant, supporting the hypothesis that FasA functions exclusively at the *fasX* promoter, and with FasX being the main effector molecule of the Fas system. Through previously characterized FasX:mRNA interactions (Danger et al. 2022; Danger, Makthal, et al. 2015; Liu et al. 2012; Ramirez‐Pena et al. 2010), streptokinase levels are increased while levels of the adhesins PrtF1, PrtF2, pilus, and Mrp are reduced (Bessen et al. 2024; Svensson et al. 2002; Podbielski et al. 1996; Jaffe et al. 1996). Thus, activation of the Fas system moves GAS from colonization mode into dissemination mode (Figure 7), promoting pathogen spread.

Our data from Figure 6 is consistent with a protein‐based signaling molecule being present in human plasma that activates Fas system activity. An appropriate next step would be to test whether the molecule is also present in human serum, with the fractionation of plasma/serum proteins/peptides being appropriate for subsequent efforts aimed at identifying the signaling factor. Importantly, while FasX levels are increased in a FasA‐dependent manner by plasma component/s, the Fas system is nevertheless highly active outside of this host fluid (i.e., in THY broth; e.g., compare the parental and *fasA* deletion strains in Figure 5C). The integration of multiple signals into the activity of a sensor kinase is not uncommon; for example, the CovS sensor kinase is regulated in opposite directions by the antimicrobial peptide LL‐37 and extracellular Mg^2+^ levels (Gryllos et al. 2007; Velarde et al. 2014). Thus, it is likely that there are signaling molecules in addition to the protein‐based factor in human plasma that feed into the Fas system to modulate activity, with at least one of these being in THY broth.

Our data (Figure 5) support a model in which FasB and FasC heterodimerize, diverging from the homodimeric architecture typical of bacterial sensor kinases. There are rare reports of two sensor kinases forming a heterodimer, such as the GacS and RetS sensor kinases of *Pseudomonas aeruginosa* (Mancl et al. 2019), but in this example GacS and RetS can also form homodimers, with RetS inhibiting the activity of GacS in both homo‐ and hetero‐dimer forms (Ryan Kaler et al. 2021). While we believe it unlikely, it is possible that conditions were not conducive for FasB or FasC homodimerization in our bacterial two‐hybrid study, even though they were for heterodimerization. The activities of some sensor kinases are modulated by interactions with accessory proteins. For example, the accessory proteins RocA and LiaF interact with their sensor kinase partners CovS and LiaS, respectively, modulating their activity (Chiang‐Ni et al. 2020; Jain et al. 2017; Lynskey et al. 2019; Lin et al. 2020; Vega et al. 2024). Our finding that the FasC kinase domain can be deleted (Figure 2E,F) is consistent with FasC serving an accessory role to the FasB sensor kinase. Biochemical studies, including co‐immunoprecipitation assays and in vitro kinase assays, will be crucial to dissect the complexities of Fas protein interactions and functions.

Phosphorylation is a common modification that can have a profound impact on protein function. Thus, phosphoproteomic studies represent important tools to investigate the prevalence and locations of this protein modification in organisms ranging from bacteria to humans (Franciosa et al. 2023; Marques et al. 2024; Mijakovic and Macek 2012). While a limited number of phosphoproteomic studies have been performed in GAS (Mikkat et al. 2024; Birk et al. 2021), due to the unstable nature of phosphor‐aspartate not lending itself to study by traditional methods, all studies to date in GAS have focused on the identification of phosphorylated serine, threonine, or tyrosine residues rather than aspartate (Mikkat et al. 2024). Thus, our study here, using the hydroxylamine alkyne probe (HA‐yne) to convert phosphorylated aspartate residues into a stable alkyne‐containing hydroxamate, is the first that characterizes phosphorylated aspartates in GAS across the proteome. The data confirmed our hypothesis, generated from our homology and mutagenesis data (Figure 5C), that aspartate 60 of FasA was phosphorylated (Figure 5D). While we isolated and analyzed both soluble and insoluble protein fractions, we detected FasA only in the insoluble fraction, which was unexpected given what we know and hypothesize about the function and localization (the cytoplasm) of FasA. This data could signal strong interactions between FasA and the membrane‐spanning FasBC proteins. However, we believe the most likely explanation is that our cell lysis step (via sonication) was inefficient, leaving a significant number of unlysed cells that pelleted along with the insoluble fraction. Consistent with this, the protein concentration was significantly higher for the unlysed sample relative to the lysed, and also two to three times as many peptides were identified in the insoluble fraction relative to the soluble (data not shown). While unfortunate, if true, it does not negatively impact our interpretation of the FasA data. Indeed, the best studied response regulator in GAS is CovR, a cytoplasmic protein that is phosphorylated by its cognate sensor kinase on aspartate D53 (Horstmann et al. 2017). Peptides overlapping D53 in CovR, all of which harbored the appropriate HA‐yne modification, were only detected in the insoluble fraction, mirroring our data with FasA (Table S3). In total, we detected peptides containing an HA‐yne modified aspartate that mapped to 160 distinct GAS proteins, with some having multiple modified aspartates. Included within the 160 proteins, aside from FasA and CovR, are additional transcriptional regulators such as CodY, which controls gene expression in response to amino acid availability (McDowell et al. 2012), and CopR, which belongs to a family of copper‐sensing transcriptional repressors (O'Brien et al. 2020). The virulence factors, M protein and C5a peptidase, are also identified in our study as being phosphorylated (Table S3) (Fischetti 2016). Currently, there are no published reports of any of these proteins being phosphorylated on aspartate residues, and hence this data may spur additional studies to assess the functional consequences of these modifications.

The replacement of the wild‐type *fasC* gene within the parental isolate MGAS2221 with a mutant version that produces FasC lacking the kinase domain, creating strain 2221fasC^1‐286^, does not impact Fas system function (Figure 2G). This is consistent with FasB harboring the kinase activity of the Fas system. A common additional activity harbored by sensor kinase proteins is phosphatase activity (Landry et al. 2018). By harboring dual kinase and phosphatase activities, sensor kinases can fine‐tune target protein phosphorylation. While not investigated in this study, we propose that FasB has phosphatase activity that reduces FasA activation and hence the activity of the Fas system. This, along with potential activators of phosphatase activity, will be a focus of downstream studies.

In summary, we present the first molecular analysis of the Fas proteins, identifying key domains and amino acids required for regulatory activity. Importantly, the Fas system is not restricted to GAS, with species including *S. zooepidemicus*, *S. equi* , *S. equisimilis*, and *S. uberis* also harboring the *fas* genes (Ramirez‐Pena et al. 2010). Thus, the value of our data likely extends beyond GAS to include a range of animal and zoonotic pathogens. Future studies will be aimed at uncovering the identity of the activator of the Fas system found in human plasma, using biochemical approaches to flesh out protein activities and interactions and testing the hypothesis that Fas promotes GAS dissemination. Longer‐term, this work may promote clinical advances by spurring discovery of factors that inhibit Fas system activity that could be administered to a patient as a novel therapeutic.

## 4 Experimental Procedures

### 4.1 Bacterial Strains and Culture Conditions

The GAS strains used in this study are listed in Table S1. All GAS strains created in this study were verified by PCR and targeted sequencing. Routine growth of liquid GAS cultures made use of Todd‐Hewitt broth with 0.2% yeast extract (THY broth), and cultures were incubated without shaking at 37°C (with 5% CO_2_). Routine growth of GAS on solid media made use of TSA blood agar plates (Fisher Scientific) or THY agar plates. Chloramphenicol (4 μg/mL) and/or spectinomycin (150 μg/mL) were added when required.

### 4.2 Creation of a Series of *fasB* Mutant and Complemented Mutant Derivatives of the Serotype M3 GAS Strain M3fasC^FIX^

M3fasC^FIX^ was previously created (previously named M3fasC^COMP^ (Cao et al. 2014)) and is an M3 GAS derivative in which the naturally occurring *fasC* mutation has been fixed, leading to a functional Fas system. To create a *fasB* mutant derivative of M3fasC^FIX^, strain M3fasC^FIX^ΔfasB, we replaced *fasB* with a spectinomycin resistance cassette, like we have previously described (Sumby et al. 2005). M3fasC^FIX^ΔfasB was created to enable assessment of which domains of FasB were required for regulatory activity, which we tested by comparing derivatives of this strain containing empty vector or plasmids expressing full‐length or truncated *fasB*. PCR primers used in these endeavors, and those below, are listed in Table S2.

### 4.3 Creation of a Series of *fasC* Mutant and Complemented Mutant Derivatives of Strain MGAS10870

MGAS10870 is a clinical serotype M3 GAS isolate and hence is naturally a *fasC* mutant strain (Cao et al. 2014). We used MGAS10870 as a parental strain to enable assessment of which domains of FasC were required for regulatory activity, which we tested by comparing derivatives of MGAS10870 containing an empty vector or plasmids expressing full‐length or truncated *fasC*.

### 4.4 Creation of a Series of *fasB* and *fasC* Mutant and Complemented Mutant Derivatives of Strain MGAS2221

MGAS2221 is a clinical serotype M1 GAS isolate that has a functioning Fas system (Ramirez‐Pena et al. 2010). We used MGAS2221 as a parental strain to enable assessment of which domains of FasB and FasC were required for regulatory activity, which we tested by comparing *fasB* and *fasC* mutant derivatives of MGAS2221 containing empty vector or plasmids expressing full‐length or truncated *fasB* or *fasC*. The *fasB* MGAS2221 mutant derivative, strain 2221ΔfasB, was created by replacing *fasB* with a spectinomycin resistance cassette. The *fasC* MGAS2221 mutant derivative, 2221ΔfasC, was created previously via a similar approach (Cao et al. 2014).

### 4.5 Creation of the *fasC* Mutant Strain 2221fasC^1‐286^

2221fasC^1‐286^ is a *fasC* mutant derivative of the clinical M1 GAS isolate MGAS2221. The wild‐type *fasC* gene in MGAS2221 was replaced with a truncated derivative that produces only amino acids 1–286 of FasC. Markerless replacement of the *fasC* allele made use of a homologous recombination‐based protocol using vector pBBL740, as we have described previously (Cao et al. 2014).

### 4.6 Creation of the *fasC* Mutant Strain 2221.fasC^MUTM3^

2221.fasC^MUT.M3^ is a *fasC* mutant derivative of MGAS2221 where the introduced *fasC* mutation, which is a 4 bp deletion, mirrors that naturally present within M3 GAS isolates. The MGAS2221 *fasC* gene was replaced with a derivative containing the 4 bp deletion, and this was achieved via a homologous recombination‐based protocol using vector pBBL740.

### 4.7 Creation of the *fasC* Mutant Strain 2221.fasC^H246A^

2221.fasC^H246A^ is a *fasC* mutant derivative of MGAS2221 in which the putative site of autophosphorylation in FasC, the histidine at amino acid 246, has been converted into an alanine. This strain was created using a homologous recombination‐based protocol with vector pBBL740.

### 4.8 Creation of the *fasA* Mutant Strain 2221ΔfasA and Its Complemented Derivative 2221fasA^Comp^

2221ΔfasA is a *fasA* deletion mutant derivative of MGAS2221. The *fasA* gene in MGAS2221 was deleted in a markerless fashion using a homologous recombination‐based protocol with vector pBBL740. A *fasA*‐complemented derivative of 2221ΔfasA was created by replacing the *fasA* deletion with a wild‐type *fasA* allele, and this was also achieved using a homologous recombination‐based protocol with vector pBBL740.

### 4.9 Creation of the *fasA* Mutant Strain 2221fasA^D60A^

2221fasA^D60A^ is a *fasA* mutant derivative of MGAS2221 in which the putative phosphorylation site in FasA, aspartate at amino acid 60, has been converted into an alanine. The *fasA* gene in MGAS2221 was replaced with the mutant allele using a homologous recombination‐based protocol with vector pBBL740.

### 4.10 Isolation of Total GAS RNA

Total RNA was isolated from the tested GAS strains as previously described (Perez et al. 2009). Briefly, the strains of interest were grown to the exponential phase of growth (O.D.600 of 0.5) in THY broth. Two volumes of RNA protect bacterial reagent (Qiagen) were added to 1 volume of GAS culture and incubated at room temperature for 5 min. Following centrifugation (5000×*g* for 10 min at 4°C), the supernatant was discarded, the cell pellets were snap‐frozen in liquid nitrogen, and the frozen pellets were placed at −80°C until ready for processing. Cells were processed using a mechanical lysis method with lysing matrix B tubes in conjunction with a FastPrep24 homogenizer (MP Biomedicals). RNA was isolated using the RNeasy kit (Qiagen) with contaminating DNA being removed with three treatments with TURBO‐DNA‐free (Life Technologies). The quality and quantity of purified RNAs were determined using a TapeStation 4150 (Agilent Tech).

### 4.11 Taqman‐Based Quantitative RT‐PCR Analyses

cDNA was synthesized from total GAS RNA using the Superscript III (ThermoFisher) reverse transcriptase as per the manufacturers' instructions. TaqMan quantitative RT‐PCR was performed using a CFX Connect real‐time system (Bio‐Rad). Gene transcript levels were compared between strains using the ΔΔCT method. TaqMan primers and probes for the genes of interest and the internal control gene *proS* are listed in Table S2.

### 4.12 Isolation of GAS Protein Fractions

To isolate GAS secreted protein fractions, aliquots (10 mL) were recovered from GAS strains grown in THY broth to mid‐exponential phase (O.D.600 = 0.5). The cells were pelleted by centrifugation (5000×*g* for 20 min at 4°C) and the supernatant filtered through a 0.22‐μm filter into 35 mL of 100% ethanol and precipitated overnight at −20°C. Precipitated proteins were collected by centrifugation (5000×*g* for 20 min at 4°C) and resuspended in SDS‐PAGE buffer.

To isolate GAS cytoplasmic and membrane protein fractions, strains were grown in 40 mL of THY broth to mid‐exponential phase (O.D.600 = 0.5). The culture was treated with 40 μL of hyaluronidase (7.5 mg/mL) and incubated on ice for 5 min before pelleting the cells by centrifugation (5000×*g* for 10 min at 4°C). The supernatant was discarded, and the pellet was resuspended in 1 mL of buffer A (2.5 mL 1 M Tris–HCl pH 7–8, 15 g sucrose, 200 μL 0.5 M EDTA, 2 mL lysozyme (20 mg/mL), 3 mL 5 U/μL mutanolysin, 5 mL protease inhibitor (Sigma), water to 50 mL). The resuspended cells were added to an additional 5 mL aliquots of buffer A, moved to 15 mL tubes, and incubated at 37°C for 2 h with end‐end rotation. Following incubation, the tubes were centrifuged (2000×*g* at 4°C for 10 min), the supernatants were discarded, and the pellets were resuspended in 600 μL of buffer B (i.e., buffer A but without the mutanolysin and lysozyme). The resuspended cells were transferred to FastPrep bead beating tubes and processed using a FastPrep‐24 machine (MP Biomedicals) at speed 5 for 20 s. This step was repeated twice, with the tubes being placed on ice for 2 min after each run. The beads were pelleted by centrifugation at 1×*g* for 10 s and the supernatants were transferred to high‐speed ultra‐centrifuge tubes filled with 1 mL of buffer B. Ultra‐centrifugation was performed for 1 h at 4°C and 50,000×*g*. The supernatants, containing cytoplasmic fractions, were isolated, mixed with equal volumes of 2X Lamelli buffer containing beta‐mercaptoethanol, and used for cytoplasmic protein Western blots. The pellets, containing the membrane protein fractions, were dissolved in 500 μL of buffer C (500 μL 1 M Na2PO4, 600 μL 5 M NaCl, 1 mL of protease inhibitor tablet, and water to 10 mL) and stored at 4°C overnight. The next day, 200 μL of 4X Lamelli buffer were added to each sample, mixed, and heated at 70°C for 15 min. After centrifuging for 2 min at 14,000×*g*, the supernatants were used to perform membrane protein Western blots.

### 4.13 Western Blot Analyses

Protein samples were separated on 10% SDS‐polyacrylamide gels and transferred to nitrocellulose membranes. The membranes were used in western blot analyses with custom sheep anti‐SKA polyclonal antibodies (created by Pacific Immunology Inc), custom rabbit anti‐FasB polyclonal antibodies (ThermoFisher; raised against amino acids 216‐448 of FasB), a commercial rabbit anti‐SLO/SPN polyclonal antibody (American Research Products Inc), and the custom rabbit polyclonal antibody anti‐Spd3 (Pacific Immunology Inc) as primary antibodies. The blots were blocked with 5% non‐fat milk in PBST buffer (2.7 mM potassium chloride, 137 mM sodium chloride pH 7.4, and 0.1% Tween 20) and incubated overnight at 4°C with specific primary antibodies. The proteins were detected using Alexa Fluor 680 donkey anti‐rabbit/sheep IgG (at a dilution of 1:10,000) secondary antibodies. The fluorescent signal was detected using a Li‐Cor Odyssey Near‐Infrared System.

### 4.14 Bacterial Two Hybrid Assay

Bacterial two‐hybrid assays were performed using GAS proteins fused to the N‐terminus of either the T18 or T25 portions of CyaA in plasmids pUT18C/pKT25 (Euromedex) (Jain et al. 2020; Karimova et al. 2000). These plasmids were constructed via conventional methods using the primers listed in Table S2. The assay was performed in BTH101 *E. coli* cells co‐transformed with the pKT25 and pUT18C‐based plasmids. Interactions between the target proteins were quantified using β‐galactosidase activity as a measure of reconstructed CyaA function via a 96‐well plate format. Briefly, co‐transformed BTH101 cells were grown overnight in 1 mL of rich media (lysogeny broth) supplemented with kanamycin (50 μg/mL), ampicillin (100 μg/mL), and IPTG (0.5 mM) at 30°C in deep‐well plates. The next morning, 50 μL of cells were transferred to a microplate containing 150 μL of water, and the plate was read at 630 nm to record the optical densities. Next, 100 μL of the overnight cultures were permeabilized in a polypropylene deep‐well plate by the addition of 20 μL of 0.1% SDS, 40 μL of chloroform, and 100 μL of Z‐buffer (60 mM sodium phosphate septahydrate, 10 mM potassium chloride, 40 mM sodium phosphate monohydrate, 1 mM magnesium sulfate septahydrate, and 50 mM beta‐mercaptoethanol), followed by aspiration and mixing. Subsequently, 100 μL of the permeabilized cells were transferred to a microplate for the enzymatic reaction. 20 μL of ONPG (4 mg/mL) was added to this solution, and the plate was incubated at room temperature for 1 h, after which time 50 μL of 1 M sodium carbonate was added to each well, and the plate was read at 415 nm. This activity was then normalized to the cell density and to a negative control strain that contained empty plasmids.

### 4.15 Proteomic Analysis of FasA Phosphorylation

This protocol was a modification of that presented by Allihn and colleagues (Allihn et al. 2021). An exponential phase (O.D.600 = 0.5) THY broth culture of GAS strain MGAS2221 was grown, and 80 mL of cells was harvested by centrifugation (6000×*g*, 4°C, 10 min), washed with ice‐cold PBS, and resuspended in 1 mL of HA‐yne buffer (20 mM HEPES, pH 4.0, 125 mM HA‐yne, 1% (w/v) LDAO). Cells were lyzed by sonication (80% int., 10 s on, 10 s off, for 10 min) under constant cooling with ice. The reaction proceeded for 1 h at 37°C without shaking. Fractions were separated by centrifugation (17,000×*g*, 4°C, 30 min). The insoluble fraction was washed twice with 1 mL ice‐cold PBS and stored at −20°C until subjection to click chemistry. The soluble fraction was precipitated in 4 mL of cold acetone (−80°C) and incubated overnight at −20°C. The precipitate was gained by centrifugation (9000×*g*, 4°C, 10 min) and washed twice by resuspension in 1 mL MeOH (chilled to −80°C) with the help of a sonicating water bath, centrifugation (9000×*g*, 4°C, 10 min), and removal of the supernatant. Soluble and insoluble fractions were resuspended in 1 mL 0.8% SDS in PBS using a sonicating water bath. The protein concentration of both fractions was determined using a Qubit fluorometer (Thermo Fisher Scientific) before adjusting to 1 mg/mL with 0.8% SDS in PBS. 1 mL of each sample was clicked to desthiobiotin azide by addition of 60 μL TBTA ligand (0.9 mL/mL in 4:1 tBuOH/DMSO), 20 μL desthiobiotin azide (5 mM in DMSO), 20 μL TCEP (13 mg/mL in water), and 20 μL CuSO4 (12.5 mg/mL in water). The click reaction was incubated for 1 h at room temperature and quenched by addition of 4 mL of cold acetone (chilled to −80°C) and stored overnight at −20°C. Precipitates were gained by centrifugation (9000×*g*, 4°C, 10 min) and washed twice by resuspension in 1 mL MeOH (chilled to −80°C), with the help of a sonicating water bath, centrifugation (9000×*g*, 4°C, 10 min), and removal of the supernatant. Pellets were dissolved in 300 μL 8 M urea in 0.1 M triethylammonium bicarbonate (TEAB) using a sonicating water bath. After reduction of disulfides by addition of 15 μL dithiothreitol (DTT; 31 mg/mL) and incubation at room temperature with shaking at 850 rpm for 1 h, free thiols were alkylated by adding 15 μL iodoacetamide (IAA; 74 mg/mL) and incubating in the dark at 25°C with shaking at 850 rpm for 30 min. Any remaining IAA was quenched by addition of 15 μL DTT (31 mg/mL) and incubating at 25°C with shaking at 850 rpm for 30 min. 900 μL 0.1 M TEAB were added to obtain a urea concentration of 2 M for trypsin digestion. 20 μL of 0.5 mg/mL sequencing grade modified trypsin (10 μg; Thermo Scientific) was added, and samples were incubated at 37°C with shaking at 220 rpm overnight. Each trypsin digest was added to 1.2 mL of washed streptavidin agarose beads (50 μL initial slurry; Thermo Scientific) in 0.2% nonyl phenoxypolyethoxylethanol (NP‐40 alternative), which were previously washed by addition of 0.2% NP‐40 alternative in PBS (4 × 1 mL), centrifugation (400 rpm, 2 min), and removal of the supernatant. The samples were incubated by rotation at room temperature for 1 h. To remove unbound peptides, the beads were centrifuged (1000×*g*, 2 min), and the supernatants removed. The beads were resuspended in 600 μL 0.1% NP‐40 alternative in PBS and transferred to a centrifuge column (Thermo Scientific). The beads were washed twice with 600 μL of 0.1% NP‐40 alternative, three times with 600 μL of PBS, and three times with 600 μL of mass spec grade water. The peptides were eluted by addition of 200 μL elution buffer (0.1% formic acid (FA) in 1:1 ACN/H2O), waiting 1 min, and then centrifuging (5000×*g*, 3 min). Two additional elution steps, each with 100 μL elution buffer, were performed. Eluted fractions were dried using a vacuum centrifuge, dissolved in 0.1% formic acid in water, and loaded onto Evosep evotips using standard protocols.

Liquid chromatography mass spectrometry (LCMS) was performed on an Evosep One LC platform (Odense, Denmark) interfaced with a Bruker timsTOF Pro 2 (Billerica, MA). Peptides from digests were separated on the Evosep one LC using the 30 sample per day method on a PepSep C18 column (15 cm × 150 μm, 1.5 um packing). Separated peptides were then ionized using a CaptiveSpray (Bruker, Billerica, MA) ionization source prior to mass spectral analysis. Mass Spectral analysis was performed on a timsTOF Pro2 mass spectrometer using DDA‐PASEF in positive mode over a mass range of 100–1700 m/z with a mobility range from 0.60 to 1.60 1/K0 and a ramp time of 100 ms. Data analysis was performed using Spectromine v4.5 (Biognosys, Schlieren, Switzerland) using the species specific FASTA database for *Streptococcus pyogenes* (UP000325300) containing 2281 entries. Default search parameters were used with the inclusion of oxidation (M) and desthiobiotin azide (DENQ) variable modifications. Data files have been deposited to the ProteomeXchange Consortium (proteomecentral.proteomexchange.org) via the PRIDE partner repository with the project accession identifier PXD066570.

### 4.16 RNA Isolation From GAS Grown in Culture and in Human Plasma

Each GAS strain was grown in triplicate THY broth cultures to the exponential phase, corresponding to an O.D.600 of 0.5. Two milliliter of each culture was transferred to separate 15 mL falcon tubes containing 4 mL of RNAprotect bacteria reagent (Qiagen) and incubated at room temperature for 5 min before centrifuging for 10 min at 5000×*g* (at 4°C). The supernatants were removed and the pellets quick‐frozen in liquid nitrogen before storing at −80°C until ready for RNA isolation. In addition to the above, 2 mL of each THY broth GAS culture was also transferred to empty 15 mL falcon tubes and the GAS pelleted by centrifugation for 10 min at 5000×*g* (at 4°C). Each cell pellet was resuspended in 1 mL of sterile PBS and transferred to 15 mL tubes containing 10 mL of pre‐warmed, mixed human plasma (Innovative Research) before incubating without shaking at 37°C (5% CO_2_) for 15 min. Tubes were centrifuged to pellet the GAS cells (10 min at 5000×*g* (at 4°C)) with the supernatants subsequently being discarded. Cell pellets were resuspended in 1 mL PBS and added to separate tubes containing 2 mL of RNAprotect bacteria reagent (Qiagen). After 5 min incubation at room temperature, the tubes were centrifuged (10 min at 5000×*g* (at 4°C)), the supernatants discarded, and the cell pellets flash frozen in liquid nitrogen. Frozen cell pellets were stored at −80°C until ready for RNA isolation. RNA was isolated from cell pellets as described above.

For some experiments, human plasma was pre‐treated with proteinase K prior to inoculation with GAS. To do this, proteinase K was added to the plasma to a final concentration of 0.4 mg/mL, incubated at 50°C for 1 h, and then treated to inactivate the proteinase K by heating to 95°C for 10 min. After cooling to room temperature, the treated plasma was centrifuged (5000×*g* at room temperature) to remove particulate matter and then aliquoted in 10 mL volumes for use as described above for GAS growth and subsequent RNA isolation.

## Author Contributions

**Sushila Baral:** writing – original draft, investigation, methodology, validation, writing – review and editing, formal analysis, data curation. **Roshika Roshika:** investigation, validation, writing – review and editing. **Clay P. Renshaw:** investigation, writing – original draft, writing – review and editing, methodology, validation. **Ameya Singh:** investigation, writing – review and editing, validation. **Ashna Prabhu:** investigation, validation, writing – review and editing. **Ira Jain:** investigation, validation, writing – review and editing. **Rebekah Woolsey:** formal analysis, data curation, investigation, writing – review and editing. **David Quilici:** writing – review and editing, formal analysis, data curation, investigation. **Yftah Tal‐Gan:** writing – review and editing, supervision, validation. **Paul Sumby:** conceptualization, funding acquisition, writing – original draft, methodology, visualization, writing – review and editing, formal analysis, project administration, data curation, supervision, resources.

## Supporting information


**Data S1:** mmi70029‐sup‐0001‐supinfo.pdf.

## Data Availability

The data that support the findings of this study are openly available in ProteomeXchange Consortium at https://proteomecentral.proteomexchange.org/, reference number PXD066570.
