# Supplementary material for: Molecular Characterization of the Group A Streptococcus Virulence‐Regulatory System FasBCAX
Source: Mol Microbiol. 2025 Oct 18;124(6):521–37. doi: 10.1111/mmi.70029 (PMC12675982; doi:10.1111/mmi.70029)
Supplement: Supplementary file 1 — Data S1: mmi70029‐sup‐0001‐supinfo.pdf. [file MMI-124-521-s001.pdf]

Supplementary Information for the manuscript by Baral, *et al.*, entitled "Molecular characterization of the group A *Streptococcus* virulence-regulatory system FasBCAX"

Figure S1
The truncated proteins FasB$^{1\text{-}423}$ and FasB$^{216\text{-}448}$ are expressed from their respective plasmids.

Figure S2
H241 of FasB is not essential for regulatory activity.

Table S1
GAS strains used in this study.

Table S2
DNA primers and probes used in this study.

Table S3
List of all 1,335 peptides identified in the proteomic study that contained an HAyne-labeled aspartate residue.

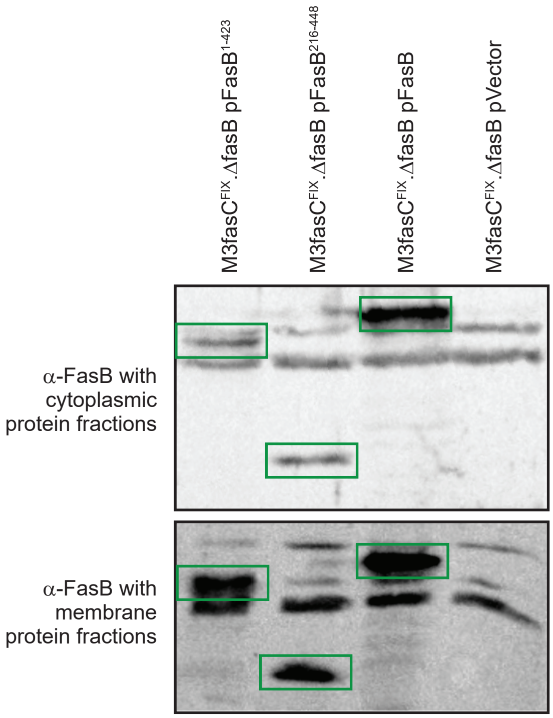

Figure S1.

The truncated proteins FasB$^{1\text{-}423}$ and FasB$^{216\text{-}448}$ are expressed from their respective plasmids. Shown are Western blots generated using cytoplasmic (top) or membrane (bottom) protein fractions from the four indicated GAS strains in association with an anti-FasB polyclonal antibody. The bands corresponding to the truncated (lanes 1 and 2) or full-length (lane 3) FasB proteins are highlighted within green rectangles.

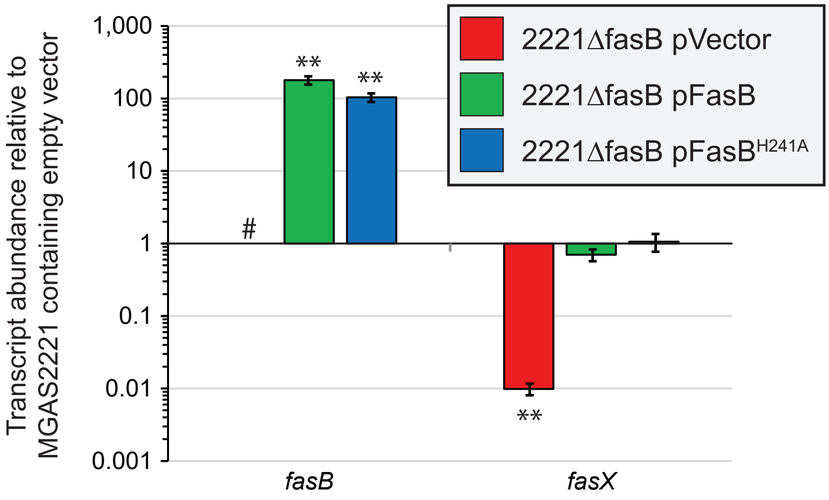

Figure S2

H241 of FasB is not essential for regulatory activity. Taqman-based quantitative RT-PCR analysis to assess whether plasmid-based expression of a FasB derivative containing a H241A substitution retains the ability to complement a *fasB* mutant GAS strain. The four strains compared in this experiment were the parental strain MGAS2221 containing empty vector, the *fasB* deletion mutant derivative 2221ΔfasB containing empty vector (red bar), a complemented derivative of 2221ΔfasB containing the FasB-expressing plasmid pFasB (green bars), and the test strain which is 2221ΔfasB containing a derivative of pFasB that expresses a H241A altered FasB protein (blue bars). Shown are the averages (± standard deviations) of triplicate samples run in triplicate. The asterisks highlight statistical significance relative to MGAS2221 containing empty vector (ANOVA with the Tukey multiple comparisons test; **, $P < 0.001$). The hashtag highlights the lack of signal due to the absence of *fasB* Taqman primer or probe binding sites within the *fasB* deletion mutant strain.

GAS strain	Description	Reference
MGAS10870	A well-characterized serotype M3 GAS isolate that was recovered in the 1980s (from Texas, USA).	Cao *et al*., 2014. *Infect Immun* **82**:1744-54.
M3fasC^{FIX}	MGAS10870 derivative in which the 4 bp deletion within the *fasC* gene that is naturally present in M3 isolates has been fixed via homologous recombination. Strain is also known as PGAS374.	Cao *et al*., 2014. *Infect Immun* **82**:1744-54.
M3fasC^{FIX} pVector	M3fasC^{FIX} derivative harboring the empty *E. coli*-GAS shuttle vector pDCBB. Strain is also known as PGAS541.	This study
M3fasC^{FIX}ΔfasB	M3fasC^{FIX} derivative in which the *fasB* gene has been replaced with a non-polar spectinomycin resistance cassette. Strain is also known as PGAS1123.	This study
M3fasC^{FIX}ΔfasB pVector	M3fasC^{FIX}ΔfasB derivative harboring the empty E. coli-GAS shuttle vector pDCBB. Strain is also known as PGAS 1129.	This study
M3fasC^{FIX}ΔfasB pFasB	M3fasC^{FIX}ΔfasB derivative harboring the *E. coli*-GAS shuttle vector pDCBB expressing the full-length and functional *fasB* gene. Strain is also known as PGAS 1128.	This study
M3fasC^{FIX}ΔfasB pFasB$^{1-219}$	M3fasC^{FIX}ΔfasB derivative harboring the *E. coli*-GAS shuttle vector pDCBB expressing a truncated *fasB* gene such that only amino acids 1-219 will be made. Strain is also known as PGAS1124.	This study
M3fasC^{FIX}ΔfasB pFasB$^{1-294}$	M3fasC^{FIX}ΔfasB derivative harboring the *E. coli*-GAS shuttle vector pDCBB expressing a truncated *fasB* gene such that only amino acids 1-294 will be made. Strain is also known as PGAS1125.	This study
M3fasC^{FIX}ΔfasB pFasB$^{1-423}$	M3fasC^{FIX}ΔfasB derivative harboring the *E. coli*-GAS shuttle vector pDCBB expressing a truncated *fasB* gene such that only amino acids 1-423 will be made. Strain is also known as PGAS1126.	This study
M3fasC^{FIX}ΔfasB pFasB$^{216-448}$	M3fasC^{FIX}ΔfasB derivative harboring the *E. coli*-GAS shuttle vector pDCBB expressing a truncated fasB gene such that only amino acids 216-448 will be made. Strain is also known as PGAS 1127.	This study
M3 pVector	MGAS10870 derivative harboring the empty *E. coli*-GAS shuttle vector pDCBB. Strain is also known as PGAS425.	Cao *et al*., 2014. *Infect Immun* **82**:1744-54.
M3 pFasC	MGAS10870 derivative harboring the *E. coli*-GAS shuttle vector pDCBB expressing the full-length and functional *fasC* gene. Strain is also known as PGAS786.	This study
M3 pFasC$^{1-209}$	MGAS10870 derivative harboring the *E. coli*-GAS shuttle vector pDCBB expressing a truncated *fasC* gene such that only amino acids 1-209 will be made. Strain is also known as PGAS798.	This study
M3 pFasC$^{1-286}$	MGAS10870 derivative harboring the *E. coli*-GAS shuttle vector pDCBB expressing a truncated *fasC* gene such that only amino acids 1-286 will be made. Strain is also known as PGAS1044.	This study
M3 pFasC$^{1-402}$	MGAS10870 derivative harboring the *E. coli*-GAS shuttle vector pDCBB expressing a truncated *fasC* gene such that only amino acids 1-402 will be made. Strain is also known as PGAS801.	This study
M3 pFasC$^{207-427}$	MGAS10870 derivative harboring the *E. coli*-GAS shuttle vector pDCBB expressing a truncated *fasC* gene such that only amino acids 207-427 will be made. Strain is also known as PGAS803.	This study
MGAS2221	A well-characterized serotype M1 GAS isolate that was recovered in 1988 (from Australia).	Sumby *et al*., 2006. *PLoS Pathog* **2**:e5.
MGAS2221 pVector	An MGAS2221 derivative harboring the empty *E. coli*-GAS shuttle vector pDCBB. Strain is also known as PGAS20.	Trevino *et al*., 2013. *Infect Immun* **81**:364-72.
2221ΔfasB	MGAS2221 derivative in which the *fasB* gene has been replaced by a non-polar spectinomycin resistance cassette. Strain is also known as PGAS38.	This study
2221 ΔfasB pVector	2221ΔfasB derivative harboring the empty *E. coli*-GAS shuttle vector pDCBB. Strain is also known as PGAS68.	This study
2221ΔfasB pFasB	2221ΔfasB derivative harboring the *E. coli*-GAS shuttle vector pDCBB expressing the full-length and functional *fasB* gene. Strain is also known as PGAS60.	This study
2221ΔfasB pFasB^{H241A}	2221ΔfasB derivative harboring the *E. coli*-GAS shuttle vector pDCBB expressing a full-length *fasB* gene that produces a protein with a H241A substitition.	This study
2221ΔfasB pFasB$^{216-448}$	2221ΔfasB derivative harboring the *E. coli*-GAS shuttle vector pDCBB expressing a truncated *fasB* gene such that only amino acids 216-448 will be made. Strain is also known as PGAS1138.	This study
2221ΔfasC	MGAS2221 derivative in which the *fasC* gene has been replaced by a non-polar spectinomycin resistance cassette. Strain is also known as PGAS65.	Cao *et al*., 2014. *Infect Immun* **82**:1744-54.
2221ΔfasC pVector	2221ΔfasC derivative harboring the empty *E. coli*-GAS shuttle vector pDCBB. Strain is also known as PGAS69.	This study
2221ΔfasC pFasC	2221ΔfasC derivative harboring the *E. coli*-GAS shuttle vector pDCBB expressing the full-length and functional fasC gene. Strain is also known as PGAS785.	This study
2221ΔfasC pFasC$^{1-286}$	2221ΔfasC derivative harboring the *E. coli*-GAS shuttle vector pDCBB expressing a truncated fasC gene such that only amino acids 1-286 will be made. Strain is also known as PGAS1139.	This study
2221fasC$^{1-286}$	MGAS2221 derivative in which *fasC* has been truncated by homologus recombination such that only amino acids 1-286 will be made. Strain is also known as PGAS1130.	This study
2221fasC$^{1-286}$ pVector	2221fasC$^{1-286}$ derivative harboring the empty *E. coli*-GAS shuttle vector pDCBB. Strain is also known as PGAS1135.	This study
2221.fasC$^{MUT.M3}$	MGAS2221 derivative in which a four bp region of fasC has been deleted similar to the mutation naturally found in serotype M3 GAS. Strain is also known as PGAS1002.	This study
2221.fasC$^{MUT.M3}$ pVector	2221.fasC$^{MUT.M3}$ derivative harboring the empty *E. coli*-GAS shuttle vector pDCBB. Strain is also known as PGAS1014.	This study
2221.fasC$^{MUT.M3}$ pFasC	2221.fasC$^{MUT.M3}$ derivative harboring the *E. coli*-GAS shuttle vector pDCBB expressing the full length and functional *fasC* gene. Strain is also known as PGAS1015.	This study
2221.fasC^{H246A}	MGAS2221 derivative in which the histidine in *fasC* at amino acid position 246 has been replaced by alanine via use of homologous recombination.	This study
2221.fasC^{H246A} pVector	2221.fasC^{H246A} derivative harboring the empty *E. coli*-GAS shuttle vector pDCBB. Strain is also known as PGAS1053.	This study
2221.fasC^{H246A} pFasC	2221.fasC^{H246A} derivative harboring the *E. coli*-GAS shuttle vector pDCBB expressing the full-length and functional *fasC* gene. Strain is also known as PGAS1054.	This study
2221ΔfasA	MGAS2221 derivative in which *fasA* has been deleted via homologous recombination. Strain is also known as PGAS142.	This study
2221ΔfasA pVector	2221ΔfasA derivative harboring the empty *E. coli*-GAS shuttle vector pDCBB. Strain is also known as PGAS145.	This study
2221ΔfasA pFasA	2221ΔfasA derivative harboring the *E. coli*-GAS shuttle vector pDCBB expressing the full-length and functional *fasA* gene. Strain is also known as PGAS162.	This study
2221fasA^{D60A}	MGAS2221 derivative in which *fasA* has been altered via homologus recombination such that there is a D60A change in the encoded FasA protein. Strain is also known as PGAS685.	This study
2221fasA^{D60A} pVector	2221fasA^{D60A} derivative harboring the empty *E. coli*-GAS shuttle vector pDCBB. Strain is also known as PGAS1055.	This study
2221fasA^{D60A} pFasA	2221fasA^{D60A} derivative harboring the *E. coli*-GAS shuttle vector pDCBB expressing the full-length and functional *fasA* gene. Strain is also known as PGAS1056.	This study
2221fasA^{Comp}	2221ΔfasA derivative in which a functional *fasA* gene has been reintroduced into the chromosome via homologus recomination at its natural site. Strain is also known as PGAS1141.	This study
2221ΔfasX	MGAS2221 derivative in which the *fasX* gene has been replaced by a non-polar spectinomycin resistance cassette. Strain is also known as PGAS1.	Ramirez-Pena *et al*., 2010. *Mol Microbiol*. **78**:1332-47.
2221 ΔfasX pVector	2221ΔfasX derivative harboring the *E. coli* -GAS shuttle vector pDCBB. Strain is also known as PGAS22.	Ramirez-Pena *et al*., 2010. *Mol Microbiol*. **78**:1332-47.
2221 ΔfasX pfasX	2221ΔfasX derivative harboring the *E. coli* -GAS shuttle vector pDCBB expressing the *FasX* sRNA. Strain is also known as PGAS10.	Ramirez-Pena *et al*., 2010. *Mol Microbiol*. **78**:1332-47.

Table S1
GAS strains used in this study.

Primer or Probe Name	Sequence (5'-3')	Purpose
PROSTMF	TACCACTGGCAAATCGTACC	Taqman primer for *proS*
PROSTMR	CATTTCAACAGCACCGATCT	Taqman primer for *proS*
PROSTMP	CACGCATGATGGTCTTGAATTTCTCA	Taqman probe for *proS*
FasBTMF	GCTATTCTTCAAAAGGTTCAGAACGT	Taqman primer for *fasB*
FasBTMR	GTCCGCAAGGATAAATAAGGATACTT	Taqman primer for *fasB*
FasBTMP	TATCGGCCTTTCTAATGCGCAGCG	Taqman probe for *fasB*
FasCTMF	CACAAAAACCAATCGTGTGGAT	Taqman primer for *fasC*
FasCTMR	ACAGTCAAAAAGTGGGCTGAGTT	Taqman primer for *fasC*
FasCTMP	CCAAACGCTTTGATGCTCAGCAGCA	Taqman probe for *fasC*
FasATMF	CGTTGTGGGCTTGAATTAGCT	Taqman primer for *fasA*
FasATMR	ATTCTGAATGAGTGGTGACAAACAC	Taqman primer for *fasA*
FasATMP	CAGCTATTCGACAAAAAGATCCTAATGCCGTTAT	Taqman probe for *fasA*
FasXTMF	ATTTACGAAGTCATGAGTTTATCGAGC	Taqman primer for *fasX*
FasXTMR	TGTCTGCCGAGCCATCATATC	Taqman primer for *fasX*
FasXTMP	CCCCAATTCAATCAAAATATTGTTTTCTCTCTCTCC	Taqman probe for *fasX*
SKATMF	CGGCTACTTTGAGGTCATTGATT	Taqman primer for *ska*
SKATMR	CCGAACCATCTTTGTCAGCAA	Taqman primer for *ska*
SKATMP	CAAGCGATGCAACCATTACTGATCGAAAC	Taqman probe for *ska*
UNR 1069	CTTTACCGAGAAATCAGAACAGTTAAAgctGATTCTGAAAATATTTTAATTAGTC	Use with UNR1157 to amplify a flank of *fasC* for use in creating the MGAS2221 derivative 2221.fasC^{H246A} which has a H246A change in FasC.
UNR 1157	caaatttcatcaagctctagtcaATGGTCACTTTTAGTCGAAAGC	Use with UNR1069 to amplify a flank of *fasC* for use in creating the MGAS2221 derivative 2221.fasC^{H246A} which has a H246A change in FasC.
UNR 1070	GACTAATTAAAATATTTTCAGAATCagcTTTAACTGTTCTGATTTCTCGGTAAAG	Use with UNR1155 to amplify a flank of *fasC* for use in creating the MGAS2221 derivative 2221.fasC^{H246A} which has a H246A change in FasC.
UNR 1155	TGTACAATTGCTAGCGTACGTAGGCATTAGTTTTGTAATTGC	Use with UNR1070 to amplify a flank of *fasC* for use in creating the MGAS2221 derivative 2221.fasC^{H246A} which has a H246A change in FasC.
UNR 1156	GCAATTACAAAACTAATGCCTACGTACGCTAGCAATTGTACA	Use with UNR1158 to amplify pBBL740 for use in creating the MGAS2221 derivative 2221.fasC^{H246A} which has a H246A change in FasC.
UNR 1158	GCTTTCGACTAAAAGTGACCATtgactagagcttgatgaaaatttg	Use with UNR1156 to amplify pBBL740 for use in creating the MGAS2221 derivative 2221.fasC^{H246A} which has a H246A change in FasC.
UNR423	TGTACAATTGCTAGCGTACGggtttatcagtgaagataagatg	Use with UNR434 to amplify a flank of *fasA* for use in creating the MGAS2221 derivative 2221fasA^{D60A} which has a D60A change in FasA.
UNR434	GTATATTCACCAATTTCAATAgCTAAAAAATACAGCTGATGG	Use with UNR423 to amplify a flank of *fasA* for use in creating the MGAS2221 derivative 2221fasA^{D60A} which has a D60A change in FasA.
UNR425	CAAACAAATTTTCATCAAGCTCTAGTTCtttcagattaagcctctttctgtac	Use with UNR433 to amplify a flank of *fasA* for use in creating the MGAS2221 derivative 2221fasA^{D60A} which has a D60A change in FasA.
UNR433	CCATCAGCTGTATTTTTTAGcTATTGAAATTGGTGAATATAC	Use with UNR425 to amplify a flank of *fasA* for use in creating the MGAS2221 derivative 2221fasA^{D60A} which has a D60A change in FasA.
UNR427	catcttatcttcactgataaaccCGTACGCTAGCAATTGTACA	Use with UNR428 to amplify pBBL740 for use in creating the MGAS2221 derivative 2221fasA^{D60A} which has a D60A change in FasA.
UNR428	gtacagaaagaggcttaatctgaaaGAACTAGAGCTTGATGAAAATTTGTTTG	Use with UNR427 to amplify pBBL740 for use in creating the MGAS2221 derivative 2221fasA^{D60A} which has a D60A change in FasA.
UNR1078	CAGTACCGGTCTATTTTAGTATTTATTTAGCTATTTTTATTTGGGTTATTG	Use with UNR1075 to amplify a flank of *fasC* for use in creating the MGAS2221 derivative 2221.fasC$^{MUT.M3}$ in which *fasC* harbors the 4 bp deletion that is naturally found in M3 isolates.
UNR1075	caaacaaatttcatcaagctctagttcCATATCGTAAGTATATCGGATACATTC	Use with UNR1078 to amplify a flank of *fasC* for use in creating the MGAS2221 derivative 2221.fasC$^{MUT.M3}$ in which *fasC* harbors the 4 bp deletion that is naturally found in M3 isolates.
UNR1079	CCAAATAAAAATAGCTAAATAAATACTAAAATAGACCGGTACTGGAAATATAG	Use with UNR1071 to amplify a flank of *fasC* for use in creating the MGAS2221 derivative 2221.fasC$^{MUT.M3}$ in which *fasC* harbors the 4 bp deletion that is naturally found in M3 isolates.
UNR1071	TGTACAATTGCTAGCGTACGGCCgttaggtgactatcagatgtttgttgtg	Use with UNR1079 to amplify a flank of *fasC* for use in creating the MGAS2221 derivative 2221.fasC$^{MUT.M3}$ in which *fasC* harbors the 4 bp deletion that is naturally found in M3 isolates.
UNR1072	cacaacaaacatctgatagtcacctaacGGCCGTACGCTAGCAATTGTACA	Use with UNR1074 to amplify pBBL740 for use in creating the MGAS2221 derivative 2221.fasC$^{MUT.M3}$ in which *fasC* harbors the 4 bp deletion that is naturally found in M3 isolates.
UNR1074	GAATGTATCCGATATACTTACGATATGgaactagagcttgatgaaaatttgtttg	Use with UNR1072 to amplify pBBL740 for use in creating the MGAS2221 derivative 2221.fasC$^{MUT.M3}$ in which *fasC* harbors the 4 bp deletion that is naturally found in M3 isolates.
UNR1160	GTCTAAAGAAGATATTTCcTAATTTGTGCGCATCATAGAAGTTG	Use with UNR1161 to amplify a flank of *fasC* for use in creating the MGAS2221 derivative 2221fasC$^{1-286}$ which makes a truncated FasC consisting of the first 286 amino acids.
UNR1161	GTACAATTGCTAGCGTACGGACGGGTTAGCATTATAGGCATTAG	Use with UNR1160 to amplify a flank of *fasC* for use in creating the MGAS2221 derivative 2221fasC$^{1-286}$ which makes a truncated FasC consisting of the first 286 amino acids.
UNR1159	CAACTTCTATGATGCGCACAAATTAgGAAATATCTTCTTTAGAC	Use with UNR1163 to amplify a flank of *fasC* for use in creating the MGAS2221 derivative 2221fasC$^{1-286}$ which makes a truncated FasC consisting of the first 286 amino acids.
UNR1163	CAAATTTTCATCAAGCTCTAGGATACATTCTTCGATTTGTTC	Use with UNR1159 to amplify a flank of *fasC* for use in creating the MGAS2221 derivative 2221fasC$^{1-286}$ which makes a truncated FasC consisting of the first 286 amino acids.
UNR1164	GAACAAATCGAAGAATGTATCCTAGAGCTTGATGAAAATTTG	Use with UNR1162 to amplify pBBL740 for use in creating the MGAS2221 derivative 2221fasC$^{1-286}$ which makes a truncated FasC consisting of the first 286 amino acids.
UNR1162	CTAATGCCTATAATGCTAACCCGTCCGTACGCTAGCAATTGTAC	Use with UNR1164 to amplify pBBL740 for use in creating the MGAS2221 derivative 2221fasC$^{1-286}$ which makes a truncated FasC consisting of the first 286 amino acids.
UNR1209	caaatttcatcaagctctagttc CCCAACCACTAGATGATTGTGCCGTTACC	Use with UNR423 to amplify *fasA* and 1kb flanking regions to make strain 2221fasA^{COMP} which is a *fasA* complemented derivative of strain 2221ΔfasA (aka. PGAS142)
UNR423	TGTACAATTGCTAGCGTACGggtttatcagtgaagataagatg	Use with UNR1209 to amplify *fasA* and 1kb flanking regions to make strain 2221fasA^{COMP} which is a *fasA* complemented derivative of strain 2221ΔfasA (aka. PGAS142)
UNR427	catcttatcttcactgataaaccCGTACGCTAGCAATTGTACA	Use with UNR1210 to amplify pBBL740 for use in making strain 2221fasACOMP which is a fasA complemented derivative of strain 2221ΔfasA (aka. PGAS142)
UNR1210	ggtaacggcacaatcatctagtggttgggGAACTAGAGCTTGATGAAAATTTGTTTG	Use with UNR427 to amplify pBBL740 for use in making strain 2221fasACOMP which is a fasA complemented derivative of strain 2221ΔfasA (aka. PGAS142)
CMPFASBF1	GCATGCATGGAAAGGAGAAAAAAATGGC	Use with CMPFASBR2 to amplify *fasB* from MGAS2221 and clone it into pDC123 to make the *fasB* complementing plasmid pFasB (transcription is driven from plasmid promoter).
CMPFASBR2	GCGGATCCCATCATCTATCCACCTCCTC	Use with CMPFASBF1 to amplify *fasB* from MGAS2221 and clone it into pDC123 to make the *fasB* complementing plasmid pFasB (transcription is driven from plasmid promoter).
UNR1090	GTTCTAAAGAATACtgatgggatctgatcttcagaacttaaggcgattaag	Use with UNR1091 and pFasB to make plasmid pFasB$^{1-219,}$ which is a derivative of pFasB that produces a truncted FasB consisting of amino acids 1-219.
UNR1091	cagatcccatcaGTATTCTTTAGAACTATGATTAAAATAAGATAG	Use with UNR1090 and pFasB to make plasmid pFasB$^{1-219,}$ which is a derivative of pFasB that produces a truncted FasB consisting of amino acids 1-219.
UNR1092	GATGATAAACACtgatgggatctgatcttcagaacttaaggcgattaag	Use with UNR1093 and pFasB to make plasmid pFasB$^{1-294}$, which is a derivative of pFasB that produces a truncated FasB consisting of amino acids 1-294.
UNR1093	cagatcccatcaGTGTTTATCATCCCAGTAGTCGTTAGCTTCATG	Use with UNR1092 and pFasB to make plasmid pFasB$^{1-294}$, which is a derivative of pFasB that produces a truncated FasB consisting of amino acids 1-294.
UNR1094	CAAAAAGTATCCTTATtgatgggatctgatcttcagaacttaaggcgattaag	Use with UNR1095 and pFasB to make plasmid pFasB$^{1-423}$, which is a derivative of pFasB that produces a truncated FasB consisting of amino acids 1-423.
UNR1095	cagatcccatcaATAAGGATACTTTTTGAGAATACGCTGCGCATTAG	Use with UNR1094 and pFasB to make plasmid pFasB$^{1-423}$, which is a derivative of pFasB that produces a truncated FasB consisting of amino acids 1-423.
UNR1096	GGAAAGGAGAAAAAAATGtctaaagaataccttaaaaatgagttgagaagagaac	Use with UNR1097 and pFasB to make plasmid pFasB$^{216-448}$, which is a derivative of pFasB that produces a truncated FasB consisting of amino acids 216-448.
UNR1097	aggtattctttagaCATTTTTTTCTCCTTTCCATGCATCGATATCTAGAG	Use with UNR1096 and pFasB to make plasmid pFasB$^{216-448}$, which is a derivative of pFasB that produces a truncated FasB consisting of amino acids 216-448.
CMPFASCF2	GCATGCATTGACTTTATCTACAAGGTGTGGCATAATGTGTGGACAAAGGAGGAGGTGGATAGATG	Use with CMPFASCR2 to amplify *fasC* from MGAS2221 and clone it into pDC123 to make the *fasC* complementing plasmid pFasC (transcription is driven from P_{SPAC} promoter).
CMPFASCR2	GCGGATCCACTTTATCTCATTTCTAAC	Use with CMPFASCF2 to amplify *fasC* from MGAS2221 and clone it into pDC123 to make the *fasC* complementing plasmid pFasC (transcription is driven from P_{SPAC} promoter).
UNR658	GATCGATTTGCTAAAtaaagtatgatgaacattttCATGCAACCTCTGTTTG	Use with UNR659 and pFasC to make plasmid pPFasC$^{1-209}$, which is a derivative of pFasC that produces a truncated FasC consisting of amino acids 1-209.
UNR659	aatgttcatcatactttaTTTAGCAAATCGATCtaatttcacaataacccaaataa	Use with UNR658 and pFasC to make plasmid pPFasC$^{1-209}$, which is a derivative of pFasC that produces a truncated FasC consisting of amino acids 1-209.
UNR1080	aaaatgttcatcatactttaATTTGTGCGCATCATAGAAGTTGCCGATTGC	Use with UNR1081 and pFasC to make plasmid pFasC$^{1-286}$, which is a derivative of pFasC that produces a truncated FasC consisting of amino acids 1-286.
UNR1081	CTTCTATGATGCGCACAAATtaaagtatgatgaacattttCATGCAACCTCTGTTTG	Use with UNR1080 and pFasC to make plasmid pFasC$^{1-286}$, which is a derivative of pFasC that produces a truncated FasC consisting of amino acids 1-286.
UNR707	CCACTTTTTGACTGTTtaaagtatgatgaacattttCATGCAACCTCTGTTTG	Use with UNR708 and pFasC to make plasmid pFasC$^{1-402}$, which is a derivative of pFasC that produces a truncated FasC consisting of amino acids 1-402.
UNR708	aaaatgttcatcatactttaAACAGTCAAAAAGTGGGCTGAGTTCTGCTG	Use with UNR707 and pFasC to make plasmid pFasC$^{1-402}$, which is a derivative of pFasC that produces a truncated FasC consisting of amino acids 1-402.
UNR711	cataatgtgtggatgTTTGCTAAAGATCAGCTGTCACAAAAATTAACAC	Use with UNR712 and pFasC to make plasmid pFasC$^{207-427}$, which is a derivative of pFasC that produces a truncated FasC consisting of amino acids 207-427.
UNR712	CTGATCTTTAGCAAAcatccacacattatgccacaccttgtagataaagtc	Use with UNR711 and pFasC to make plasmid pFasC$^{207-427}$, which is a derivative of pFasC that produces a truncated FasC consisting of amino acids 207-427.
CMPFASAF2	GCATGCATTGACTTTATCTACAAGGTGTGGCATAATGTGTGGAGAAATGAGATAAAGTATGATG	Use with CMPFASAR to amplify *fasA* from MGAS2221 and clone it into pDC123 to make the *fasA* complementing plasmid pFasA (transcription is driven from P_{SPAC} promoter).
CMPFASAR	GCAGATCTCTTTAGCGAAGAGCCTCCCAC	Use with CMPFASAF2 to amplify *fasA* from MGAS2221 and clone it into pDC123 to make the *fasA* complementing plasmid pFasA (transcription is driven from P_{SPAC} promoter).
FASBA	TTCTTGGCTGGAATCCTGGCGGAGAAG	Use with FASBD to amplify the mutated *fasB* gene (due to insertion of spectinomycin resistance cassette) from 2221ΔfasB (aka. PGAS38) and transform into M3fasC^{FIX} to create strain M3fasC^{FIX}ΔfasB.
FASBD	GGTAAATGATCGATAACATCTGG	Use with FASBA to amplify the mutated *fasB* gene (due to insertion of spectinomycin resistance cassette) from 2221ΔfasB (aka. PGAS38) and transform into M3fasC^{FIX} to create strain M3fasC^{FIX}ΔfasB.
FASBA	TTCTTGGCTGGAATCCTGGCGGAGAAG	Use with FASBB to amplify a flank of *fasB* for use in creating the MGAS2221 derivative strain 2221ΔfasB in which a spectinomycin resistance cassette has replaced the *fasB* gene
FASBB	GTTATAGTTATTATAACATGTATTGTAATGTGATATCAGATAGAAAGG	Use with FASBA to amplify a flank of *fasB* for use in creating the MGAS2221 derivative strain 2221ΔfasB in which a spectinomycin resistance cassette has replaced the *fasB* gene
FASBC	CTATTTAAATAACAGATTAAAAAAATTATAAGTTTTGATAAGGAATTTAG	Use with FASBD to amplify a flank of *fasB* for use in creating the MGAS2221 derivative strain 2221ΔfasB in which a spectinomycin resistance cassette has replaced the *fasB* gene
FASBD	GGTAAATGATCGATAACATCTGG	Use with FASBC to amplify a flank of *fasB* for use in creating the MGAS2221 derivative strain 2221ΔfasB in which a spectinomycin resistance cassette has replaced the *fasB* gene
FASBSPECF	CCTTTCTATCTGATATCACATTACAATACATGTTATAATAACTATAAC	Use with FASBSPECR to amplify a spectinomycin resistance cassette for use in creating the MGAS2221 derivative strain 2221ΔfasB in which the cassette has replaced the *fasB* gene
FASBSPECR	CTAAATTCCTTATCAAAACTTATAATTTTTTTAATCTGTTATTTAAATAG	Use with FASBSPECF to amplify a spectinomycin resistance cassette for use in creating the MGAS2221 derivative strain 2221ΔfasB in which the cassette has replaced the *fasB* gene
PGFASAA	GCGGATCCGCTGTCTTAGGATTTAGCATTGG	Use with PGFASAB to amplify a flank of *fasA* for use in creating the MGAS2221 derivative strain 2221ΔfasA in which *fasA* has been removed via pBBL740-based homologous recombination
PGFASAB	CTTTAGCGAAGAGCCTCCTCAAGAATAAAAATGTTCATC	Use with PGFASAA to amplify a flank of *fasA* for use in creating the MGAS2221 derivative strain 2221ΔfasA in which *fasA* has been removed via pBBL740-based homologous recombination
PGFASAC	GATGAACATTTTTATTCTTGAGGAGGCTCTTCGCTAAAG	Use with PGFASAD to amplify a flank of *fasA* for use in creating the MGAS2221 derivative strain 2221ΔfasA in which *fasA* has been removed via pBBL740-based homologous recombination
PGFASAD	GCGGATCCCCAACCACTAGATGATTGTGCCG	Use with PGFASAC to amplify a flank of *fasA* for use in creating the MGAS2221 derivative strain 2221ΔfasA in which *fasA* has been removed via pBBL740-based homologous recombination
UNR1262	TATGGGAAAgcTCTTGAGAAACTCTATCGAGATGTAAGAGC	Use with UNR1263 to amplify the whole of plasmid pFasB but make a H241A change in FasB. Used in Gibson Assembly by itself.
UNR1263	TTCTCAAGAgcTTTCCCATAAATCTCTAAGTTAGTCATATAAGC	Use with UNR1262 to amplify the whole of plasmid pFasB but make a H241A change in FasB. Used in Gibson Assembly by itself.

Table S2
DNA primers and probes used in this study

**Protein Fraction.** This column highlights whether the phosphorylated peptide identified was detected in the soluble or in the unsoluble protein fraction	**Gene names.** This column lists the name of the gene that produces the protein from which the identified phosphorylated peptide is derived from	**PG.ProteinAccessions.** This column lists the UniProt accession number for the protein from which the identified phosphorylated peptide is derived from	**PG.ProteinDescriptions.** This column follows the naming convention used by the UniProt database, which uniquely identifies a protein and the organism it came from	**PG.ProteinNames.** This column lists the descriptive protein names from the FASTA database using Streptococcus pyogenes (STRPY) as the reference genome	**PEP.IsProteinGroupSpecific.** This indicates whether a peptide is specific to a particular protein group, with TRUE meaning that the peptide uniquely belongs to one protein group and FALSE meaning that the peptide is shared among multiple protein groups	**PEP.IsProteotypic.** This indicates if a peptide is unique to a single protein within the database. TRUE represents a single protein or isoform while FALSE does not	**PEP.StrippedSequence.** This column shows the peptide sequence that has been "stripped" of any variable post-translational modifications (PTMs), giving the core, underlying amino acid sequence	**MS1Quantity.** This column represents peptide quantification values based on the intensity of their intact, unfragmented precursor ions	**PSM.Label-Free Quant [Raw].** This represents the unnormalised raw intensity value for the peptide-spectrum match (PSM) generated without using chemical or isotopic labels	**PP.Charge.** This represents the charge state (z) of the precursor ion associated with the peptide post-processing	**P.MoleculeID.** An internal identifier used by SpectroMine to track the molecule (peptide or modified peptide) across the analysis, ensuring that all data points, such as mass-to-charge (m/z) ratio, retention time, and intensity, are correctly associated with the same precursor molecule	**PSM.MS2ScanNumber.** This represents the scan number of the MS2 spectrum in which the peptide is identified. This is useful for linking identification back to raw data	**PSM.Search.** The search engine or method used to identify the peptide and create Peptide-Spectrum Matches (PSMs)	**PSM.IsUsedForQuantification.** In spectromine, not all Peptide Spectrum Matches are used. Those PSMs that have qualities like a low false discovery rate (FDR) might be excluded. This coulumn represents a Boolean flag (TRUE/FALSE) indicating whether this particular PSM was used for quantification. "TRUE" means inclusion of PSM that matches the quality filter set by spectromine software
Insoluble	acoA	A0A4V6IY50	Acetoin dehydrogenase (TPP-dependent)	A0A4V6IY50_STRPY	FALSE	FALSE	AEAYGIPGFYCEDGNDVMAVYETMGK	776.2945	776.2945	3	AEAYGIPGFYC[Carbamidor	59316	1	TRUE
Insoluble	acoA	A0A4V6IY50	Acetoin dehydrogenase (TPP-dependent)	A0A4V6IY50_STRPY	FALSE	FALSE	AEAYGIPGFYCEDGNDVMAVYETMGK	445.1703	445.1703	4	AEAYGIPGFYC[Carbamidor	59410	1	TRUE
Soluble	adk	A0A4U9C4E5	Adenylate kinase	A0A4U9C4E5_STRPY	TRUE	TRUE	LGLVTDIEGNQEITDVFADVEK	311.09344	311.09344	3	LGLVTD[HAyneDE]IEGNQE	68743	1	TRUE
Soluble	adk	A0A4U9C4E5	Adenylate kinase	A0A4U9C4E5_STRPY	TRUE	TRUE	LGLVTDIEGNQEITDVFADVEK	312.4453	312.4453	3	LGLVTD[HAyneDE]IEGNQE	68714	1	TRUE
Soluble	adk	A0A4U9C4E5	Adenylate kinase	A0A4U9C4E5_STRPY	TRUE	TRUE	TIEQAHALDATLEELGLR	655.1177	655.1177	3	TIEQAHALD[HAyneDE]ATLI	56015	1	TRUE
Soluble	adk	A0A4U9C4E5	Adenylate kinase	A0A4U9C4E5_STRPY	TRUE	TRUE	TIEQAHALDATLEELGLR	655.1177	655.1177	3	TIEQAHALD[HAyneDE]ATLI	56003	1	TRUE
Soluble	adk	A0A4U9C4E5	Adenylate kinase	A0A4U9C4E5_STRPY	TRUE	TRUE	TIEQAHALDATLEELGLR	655.1135	655.1135	3	TIEQAHALD[HAyneDE]ATLI	56100	1	TRUE
Soluble	adk	A0A4U9C4E5	Adenylate kinase	A0A4U9C4E5_STRPY	TRUE	TRUE	TIEQAHALDATLEELGLR	655.1177	655.1177	3	TIEQAHALD[HAyneDE]ATLI	56016	1	FALSE
Soluble	adk	A0A4U9C4E5	Adenylate kinase	A0A4U9C4E5_STRPY	TRUE	TRUE	LDGVINIK	452.58484	452.58484	3	LD[HAyneDE]GVINIK	32901	1	TRUE
Soluble	adk	A0A4U9C4E5	Adenylate kinase	A0A4U9C4E5_STRPY	TRUE	TRUE	LDGVINIK	463.8091	463.8091	3	LD[HAyneDE]GVINIK	32948	1	TRUE
Soluble	adk	A0A4U9C4E5	Adenylate kinase	A0A4U9C4E5_STRPY	TRUE	TRUE	LDGVINIK	307.50882	307.50882	3	LD[HAyneDE]GVINIK	34920	1	TRUE
Soluble	adk	A0A4U9C4E5	Adenylate kinase	A0A4U9C4E5_STRPY	TRUE	TRUE	LDGVINIK	309.09985	309.09985	3	LD[HAyneDE]GVINIK	34976	1	FALSE
Soluble	adk	A0A4U9C4E5	Adenylate kinase	A0A4U9C4E5_STRPY	TRUE	TRUE	LDGVINIK	463.8091	463.8091	3	LD[HAyneDE]GVINIK	32937	1	FALSE
Soluble	adk	A0A4U9C4E5	Adenylate kinase	A0A4U9C4E5_STRPY	TRUE	TRUE	LDGVINIK	310.92407	310.92407	3	LD[HAyneDE]GVINIK	34866	1	FALSE
Soluble	adk	A0A4U9C4E5	Adenylate kinase	A0A4U9C4E5_STRPY	TRUE	TRUE	LDGVINIK	307.50882	307.50882	3	LD[HAyneDE]GVINIK	34887	1	FALSE
Soluble	adk	A0A4U9C4E5	Adenylate kinase	A0A4U9C4E5_STRPY	TRUE	TRUE	LDGVINIK	265.61166	265.61166	2	LD[HAyneDE]GVINIK	34992	1	TRUE
Soluble	adk	A0A4U9C4E5	Adenylate kinase	A0A4U9C4E5_STRPY	TRUE	TRUE	LDGVINIK	542.01135	542.01135	2	LD[HAyneDE]GVINIK	32911	1	TRUE
Insoluble	ahpC	A0A5S4TE32	Alkyl hydroperoxide reductase C	A0A5S4TE32_STRPY	TRUE	TRUE	SLGVEVYSVSTDTHFVHK	1349.855	1349.855	4	SLGVEVYSVSTD[HAyneDE]	33322	1	TRUE
Insoluble	ahpC	A0A5S4TE32	Alkyl hydroperoxide reductase C	A0A5S4TE32_STRPY	TRUE	TRUE	SLGVEVYSVSTDTHFVHK	173.9815	173.9815	3	SLGVEVYSVSTD[HAyneDE]	33361	1	TRUE
Insoluble	ahpC	A0A5S4TE32	Alkyl hydroperoxide reductase C	A0A5S4TE32_STRPY	TRUE	TRUE	DASTLIDK	336.03186	336.03186	2	D[HAyneDE]ASTLIDK	25365	1	TRUE
Insoluble	ahpC	A0A5S4TE32	Alkyl hydroperoxide reductase C	A0A5S4TE32_STRPY	TRUE	TRUE	DASTLIDK	305.85184	305.85184	3	D[HAyneDE]ASTLIDK	25273	2	TRUE
Soluble	ahpC	A0A5S4TE32	Alkyl hydroperoxide reductase C	A0A5S4TE32_STRPY	TRUE	TRUE	SLGVEVYSVSTDTHFVHK	1284.4288	1284.4288	4	SLGVEVYSVSTD[HAyneDE]	28695	1	TRUE
Soluble	ahpC	A0A5S4TE32	Alkyl hydroperoxide reductase C	A0A5S4TE32_STRPY	TRUE	TRUE	SLGVEVYSVSTDTHFVHK	1284.4288	1284.4288	4	SLGVEVYSVSTD[HAyneDE]	29619	1	TRUE
Soluble	ahpC	A0A5S4TE32	Alkyl hydroperoxide reductase C	A0A5S4TE32_STRPY	TRUE	TRUE	SLGVEVYSVSTDTHFVHK	1284.4288	1284.4288	4	SLGVEVYSVSTD[HAyneDE]	28689	1	TRUE
Soluble	ahpC	A0A5S4TE32	Alkyl hydroperoxide reductase C	A0A5S4TE32_STRPY	TRUE	TRUE	SLGVEVYSVSTDTHFVHK	1284.4288	1284.4288	4	SLGVEVYSVSTD[HAyneDE]	29572	1	FALSE
Soluble	ahpC	A0A5S4TE32	Alkyl hydroperoxide reductase C	A0A5S4TE32_STRPY	TRUE	TRUE	SLGVEVYSVSTDTHFVHK	391.0425	391.0425	3	SLGVEVYSVSTD[HAyneDE]	28680	1	TRUE
Soluble	ahpC	A0A5S4TE32	Alkyl hydroperoxide reductase C	A0A5S4TE32_STRPY	TRUE	TRUE	SLGVEVYSVSTDTHFVHK	566.2832	566.2832	3	SLGVEVYSVSTD[HAyneDE]	29570	1	TRUE
Soluble	ahpC	A0A5S4TE32	Alkyl hydroperoxide reductase C	A0A5S4TE32_STRPY	TRUE	TRUE	SLGVEVYSVSTDTHFVHK	391.0425	391.0425	3	SLGVEVYSVSTD[HAyneDE]	28672	1	TRUE
Soluble	ahpC	A0A5S4TE32	Alkyl hydroperoxide reductase C	A0A5S4TE32_STRPY	FALSE	FALSE	EGAETLTPSLDLVGK	381.1754	381.1754	3	EGAETLTPSLD[HAyneDE]L\	45935	3	TRUE
Soluble	ahpC	A0A5S4TE32	Alkyl hydroperoxide reductase C	A0A5S4TE32_STRPY	TRUE	TRUE	DASTLIDK	411.49527	411.49527	2	D[HAyneDE]ASTLIDK	21894	1	TRUE
Soluble	ahpC	A0A5S4TE32	Alkyl hydroperoxide reductase C	A0A5S4TE32_STRPY	TRUE	TRUE	DASTLIDK	406.3183	406.3183	2	D[HAyneDE]ASTLIDK	21867	1	TRUE
Soluble	ahpC	A0A5S4TE32	Alkyl hydroperoxide reductase C	A0A5S4TE32_STRPY	TRUE	TRUE	DASTLIDK	384.59778	384.59778	3	D[HAyneDE]ASTLIDK	22304	1	TRUE
Soluble	ahpC	A0A5S4TE32	Alkyl hydroperoxide reductase C	A0A5S4TE32_STRPY	TRUE	TRUE	DASTLIDK	384.6794	384.6794	3	D[HAyneDE]ASTLIDK	22382	1	TRUE
Soluble	ahpC	A0A5S4TE32	Alkyl hydroperoxide reductase C	A0A5S4TE32_STRPY	TRUE	TRUE	DASTLIDK	394.89856	394.89856	3	D[HAyneDE]ASTLIDK	21923	1	TRUE
Soluble	ahpC	A0A5S4TE32	Alkyl hydroperoxide reductase C	A0A5S4TE32_STRPY	TRUE	TRUE	DASTLIDK	413.05527	413.05527	2	DASTLID[HAyneDE]K	27640	1	TRUE
Insoluble	ahpF	A0A5S4TF38	Alkyl hydroperoxide reductase subunit F	A0A5S4TF38_STRPY	TRUE	TRUE	SYDVDIIK	323.2543	323.2543	2	SYD[HAyneDE]VDIIK	36949	1	TRUE
Insoluble	ahpF	A0A5S4TF38	Alkyl hydroperoxide reductase subunit F	A0A5S4TF38_STRPY	TRUE	TRUE	SYDVDIIK	333.5959	333.5959	2	SYD[HAyneDE]VDIIK	36888	1	TRUE
Insoluble	argS	A0A5S4TGV4	Arginine--tRNA ligase	A0A5S4TGV4_STRPY	TRUE	TRUE	SDGATLYITR	339.06573	339.06573	3	SD[HAyneDE]GATLYITR	33318	2	TRUE
Insoluble	argS	A0A5S4TGV4	Arginine--tRNA ligase	A0A5S4TGV4_STRPY	TRUE	TRUE	VVPELEQDAIFNLLETPK	223.34346	223.34346	2	VVPELEQD[HAyneDE]AIFN	76189	1	TRUE
Soluble	aroD	A0A4U7IGY1	3-dehydroquinate dehydratase	A0A4U7IGY1_STRPY	TRUE	TRUE	IAVMPQSEQDVLDLMNYTR	406.508	406.508	2	IAVM[Oxidation (M)]PQSE(	47438	2	TRUE
Insoluble	asnC	A0A4U7GWX4	Asparagine--tRNA ligase	A0A4U7GWX4_STRPY	TRUE	TRUE	EDNYDALVAK	254.84471	254.84471	2	ED[HAyneDE]NYDALVAK	33985	1	TRUE
Insoluble	asnC	A0A4U7GWX4	Asparagine--tRNA ligase	A0A4U7GWX4_STRPY	TRUE	TRUE	EDNYDALVAK	247.20703	247.20703	2	ED[HAyneDE]NYDALVAK	33980	1	TRUE
Insoluble	aspS	A0A5S4TEY4	Aspartate--tRNA ligase	A0A5S4TEY4_STRPY	TRUE	TRUE	VTDGQLAGPVAK	242.39084	242.39084	3	VTD[HAyneDE]GQLAGPVAI	24245	1	TRUE
Insoluble	atpA	A0A4V6KLC8	ATP synthase subunit alpha	A0A4V6KLC8_STRPY	TRUE	TRUE	TSVAIDAILNQK	283.75647	283.75647	3	TSVAID[HAyneDE]AILNQK	46899	1	TRUE
Insoluble	atpA	A0A4V6KLC8	ATP synthase subunit alpha	A0A4V6KLC8_STRPY	TRUE	TRUE	TSVAIDAILNQK	268.36304	268.36304	3	TSVAID[HAyneDE]AILNQK	46878	2	TRUE
Insoluble	atpA	A0A4V6KLC8	ATP synthase subunit alpha	A0A4V6KLC8_STRPY	TRUE	TRUE	TSVAIDAILNQK	268.36304	268.36304	3	TSVAID[HAyneDE]AILNQK	46888	1	TRUE
Insoluble	atpA	A0A4V6KLC8	ATP synthase subunit alpha	A0A4V6KLC8_STRPY	TRUE	TRUE	TSVAIDAILNQK	268.36304	268.36304	3	TSVAID[HAyneDE]AILNQK	46832	1	FALSE
Insoluble	atpA	A0A4V6KLC8	ATP synthase subunit alpha	A0A4V6KLC8_STRPY	TRUE	TRUE	TSVAIDAILNQK	199.499	199.499	2	TSVAID[HAyneDE]AILNQK	46941	1	TRUE
Insoluble	atpA	A0A4V6KLC8	ATP synthase subunit alpha	A0A4V6KLC8_STRPY	TRUE	TRUE	ELEAFTQFGSDLDAATQAK	155.66685	155.66685	2	ELEAFTQFGSD[HAyneDE]L	59629	1	TRUE
Insoluble	atpA	A0A4V6KLC8	ATP synthase subunit alpha	A0A4V6KLC8_STRPY	TRUE	TRUE	AIDALVPIGR	153.46342	153.46342	3	AID[HAyneDE]ALVPIGR	50047	1	TRUE
Insoluble	atpD	A0A4U9BWH6	ATP synthase subunit beta	A0A4U9BWH6_STRPY	TRUE	TRUE	VIDLLAPYLK	317.00342	317.00342	2	VID[HAyneDE]LLAPYLK	69300	1	TRUE
Insoluble	clpX	A0A4U7GJW1	ATP-dependent Clp protease ATP-binding	A0A4U7GJW1_STRPY	FALSE	FALSE	LIQAADYNVER	441.14636	441.14636	3	LIQAAD[HAyneDE]YNVER	27496	3	TRUE
Soluble	clpX	A0A4U7GJW1	ATP-dependent Clp protease ATP-binding	A0A4U7GJW1_STRPY	TRUE	TRUE	SIIEETMLDIMFEIPSQEDVTK	996.0642	996.0642	3	SIIEETM[Oxidation (M)]L[	63920	3	TRUE
Insoluble	codY	A0A4U7IGJ4	Global transcriptional regulator CodY	A0A4U7IGJ4_STRPY	TRUE	TRUE	LADIIDCNACIINGGGTLLGYAMK	552.8602	552.8602	4	LADIID[HAyneDE]C[Carba	74985	2	TRUE
Insoluble	codY	A0A4U7IGJ4	Global transcriptional regulator CodY	A0A4U7IGJ4_STRPY	TRUE	TRUE	LADIIDCNACIINGGGTLLGYAMK	310.3368	310.3368	3	LADIID[HAyneDE]C[Carba	72247	1	TRUE
Insoluble	codY	A0A4U7IGJ4	Global transcriptional regulator CodY	A0A4U7IGJ4_STRPY	TRUE	TRUE	LADIIDCNACIINGGGTLLGYAMK	244.25735	244.25735	3	LADIID[HAyneDE]C[Carba	72441	1	TRUE
Insoluble	codY	A0A4U7IGJ4	Global transcriptional regulator CodY	A0A4U7IGJ4_STRPY	TRUE	TRUE	LADIIDCNACIINGGGTLLGYAMK	656.088	656.088	3	LADIID[HAyneDE]C[Carba	71906	1	TRUE
Insoluble	copR	A0A4U7GQ41	DNA-binding response regulator	A0A4U7GQ41_STRPY	TRUE	TRUE	AKDNAYDIMILDIMMPIK	1360.7433	1360.7433	3	AKDNAYDIM[Oxidation (M)	48340	1	TRUE
Insoluble	copR	A0A4U7GQ41	DNA-binding response regulator	A0A4U7GQ41_STRPY	TRUE	TRUE	AKDNAYDIMILDIMMPIK	1359.3011	1359.3011	3	AKDNAYDIM[Oxidation (M)	48461	1	TRUE
Insoluble	copR	A0A4U7GQ41	DNA-binding response regulator	A0A4U7GQ41_STRPY	TRUE	TRUE	AKDNAYDIMILDIMMPIK	1301.6797	1301.6797	4	AKDNAYDIM[Oxidation (M)	48410	1	TRUE
Insoluble	copR	A0A4U7GQ41	DNA-binding response regulator	A0A4U7GQ41_STRPY	TRUE	TRUE	AKDNAYDIMILDIMMPIK	1301.6797	1301.6797	3	AKDNAYDIM[Oxidation (M)	48462	1	TRUE
Insoluble	copR	A0A4U7GQ41	DNA-binding response regulator	A0A4U7GQ41_STRPY	TRUE	TRUE	AKDNAYDIMILDIMMPIK	1301.6797	1301.6797	4	AKDNAYDIM[Oxidation (M)	48373	1	TRUE
Insoluble	copR	A0A4U7GQ41	DNA-binding response regulator	A0A4U7GQ41_STRPY	TRUE	TRUE	AKDNAYDIMILDIMMPIK	145.86351	145.86351	3	AKDNAYDIMILD[HAyneDE]	76360	1	TRUE
Insoluble	copR	A0A4U7GQ41	DNA-binding response regulator	A0A4U7GQ41_STRPY	TRUE	TRUE	DNAYDIMILDIMMPIK	631.436	631.436	3	DNAYDIM[Oxidation (M)]IL	62417	1	TRUE
Insoluble	copR	A0A4U7GQ41	DNA-binding response regulator	A0A4U7GQ41_STRPY	TRUE	TRUE	DNAYDIMILDIMMPIK	704.85077	704.85077	3	DNAYDIM[Oxidation (M)]IL	62332	1	TRUE
Insoluble	copR	A0A4U7GQ41	DNA-binding response regulator	A0A4U7GQ41_STRPY	TRUE	TRUE	DNAYDIMILDIMMPIK	631.436	631.436	3	DNAYDIM[Oxidation (M)]IL	62491	1	TRUE
Insoluble	copR	A0A4U7GQ41	DNA-binding response regulator	A0A4U7GQ41_STRPY	TRUE	TRUE	DNAYDIMILDIMMPIK	631.436	631.436	3	DNAYDIM[Oxidation (M)]IL	62497	1	FALSE
Insoluble	copR	A0A4U7GQ41	DNA-binding response regulator	A0A4U7GQ41_STRPY	TRUE	TRUE	DNAYDIMILDIMMPIK	697.91626	697.91626	3	DNAYDIM[Oxidation (M)]IL	62427	1	FALSE
Insoluble	copR	A0A4U7GQ41	DNA-binding response regulator	A0A4U7GQ41_STRPY	TRUE	TRUE	DNAYDIMILDIMMPIK	628.6291	628.6291	3	DNAYDIM[Oxidation (M)]IL	62312	1	FALSE
Insoluble	copR	A0A4U7GQ41	DNA-binding response regulator	A0A4U7GQ41_STRPY	TRUE	TRUE	DNAYDIMILDIMMPIK	501.9967	501.9967	2	DNAYDIM[Oxidation (M)]IL	62349	1	TRUE
Insoluble	copR	A0A4U7GQ41	DNA-binding response regulator	A0A4U7GQ41_STRPY	TRUE	TRUE	DNAYDIMILDIMMPIK	434.20508	434.20508	4	DNAYDIM[Oxidation (M)]IL	62435	1	TRUE
Insoluble	copR	A0A4U7GQ41	DNA-binding response regulator	A0A4U7GQ41_STRPY	TRUE	TRUE	DNAYDIMILDIMMPIK	429.00732	429.00732	4	DNAYDIM[Oxidation (M)]IL	62274	2	TRUE
Insoluble	covR	A0A0K0P1B7	CovR	A0A0K0P1B7_STRPY	TRUE	TRUE	EFDLILLDLMLPEMDGFEVTR	1157.2576	1157.2576	4	EFDLILLD[HAyneDE]LM[O:	77828	1	TRUE
Insoluble	covR	A0A0K0P1B7	CovR	A0A0K0P1B7_STRPY	TRUE	TRUE	EFDLILLDLMLPEMDGFEVTR	1158.5387	1158.5387	4	EFDLILLD[HAyneDE]LM[O:	77134	2	TRUE
Insoluble	covR	A0A0K0P1B7	CovR	A0A0K0P1B7_STRPY	TRUE	TRUE	EFDLILLDLMLPEMDGFEVTR	936.1511	936.1511	3	EFDLILLD[HAyneDE]LM[O:	77972	1	TRUE
Insoluble	covR	A0A0K0P1B7	CovR	A0A0K0P1B7_STRPY	TRUE	TRUE	EFDLILLDLMLPEMDGFEVTR	936.1511	936.1511	3	EFDLILLD[HAyneDE]LMLPI	77985	1	TRUE
Insoluble	covR	A0A0K0P1B7	CovR	A0A0K0P1B7_STRPY	TRUE	TRUE	EFDLILLDLMLPEMDGFEVTR	786.66425	786.66425	3	EFDLILLD[HAyneDE]LM[O:	77826	1	TRUE
Insoluble	covR	A0A0K0P1B7	CovR	A0A0K0P1B7_STRPY	TRUE	TRUE	EFDLILLDLMLPEMDGFEVTR	786.66425	786.66425	3	EFDLILLD[HAyneDE]LM[O:	77847	1	TRUE
Insoluble	covR	A0A0K0P1B7	CovR	A0A0K0P1B7_STRPY	TRUE	TRUE	EFDLILLDLMLPEMDGFEVTR	777.4856	777.4856	3	EFDLILLD[HAyneDE]LMLPI	78169	1	TRUE
Insoluble	covR	A0A0K0P1B7	CovR	A0A0K0P1B7_STRPY	TRUE	TRUE	EFDLILLDLMLPEMDGFEVTR	234.8139	234.8139	4	EFDLILLD[HAyneDE]LMLPI	78171	1	TRUE
Insoluble	covR	A0A0K0P1B7	CovR	A0A0K0P1B7_STRPY	TRUE	TRUE	EFDLILLDLMLPEMDGFEVTRR	955.9178	955.9178	4	EFDLILLD[HAyneDE]LM[O:	77161	2	TRUE
Insoluble	covR	A0A0K0P1B7	CovR	A0A0K0P1B7_STRPY	TRUE	TRUE	EFDLILLDLMLPEMDGFEVTRR	955.36536	955.36536	4	EFDLILLD[HAyneDE]LM[O:	77243	1	TRUE
Insoluble	covR	A0A0K0P1B7	CovR	A0A0K0P1B7_STRPY	TRUE	TRUE	EFDLILLDLMLPEMDGFEVTRR	955.3881	955.3881	4	EFDLILLD[HAyneDE]LM[O:	77233	1	TRUE
Insoluble	covR	A0A0K0P1B7	CovR	A0A0K0P1B7_STRPY	TRUE	TRUE	EFDLILLDLMLPEMDGFEVTRR	500.79807	500.79807	3	EFDLILLD[HAyneDE]LM[O:	77168	2	TRUE
Insoluble	covR	A0A0K0P1B7	CovR	A0A0K0P1B7_STRPY	TRUE	TRUE	EFDLILLDLMLPEMDGFEVTRR	499.3092	499.3092	3	EFDLILLD[HAyneDE]LM[O:	77232	1	TRUE
Insoluble	covR	A0A0K0P1B7	CovR	A0A0K0P1B7_STRPY	TRUE	TRUE	EFDLILLDLMLPEMDGFEVTRR	499.3092	499.3092	3	EFDLILLD[HAyneDE]LM[O:	77209	1	TRUE
Insoluble	covR	A0A0K0P1B7	CovR	A0A0K0P1B7_STRPY	TRUE	TRUE	EFDLILLDLMLPEMDGFEVTRR	499.3092	499.3092	3	EFDLILLD[HAyneDE]LM[O:	77239	1	FALSE
Insoluble	covR	A0A0K0P1B7	CovR	A0A0K0P1B7_STRPY	TRUE	TRUE	EFDLILLDLMLPEMDGFEVTRR	499.3092	499.3092	3	EFDLILLD[HAyneDE]LM[O:	77231	1	FALSE
Insoluble	covR	A0A0K0P1B7	CovR	A0A0K0P1B7_STRPY	TRUE	TRUE	EFDLILLDLMLPEMDGFEVTRR	499.2775	499.2775	3	EFDLILLD[HAyneDE]LM[O:	77304	1	FALSE
Insoluble	covR	A0A0K0P1B7	CovR	A0A0K0P1B7_STRPY	TRUE	TRUE	EFDLILLDLMLPEMDGFEVTRR	153.65068	153.65068	5	EFDLILLD[HAyneDE]LM[O:	77236	1	TRUE
Insoluble	cypB	A0A4U9SZ07	Peptidyl-prolyl cis-trans isomerase	A0A4U9SZ07_STRPY	TRUE	TRUE	VINDFMIQSGDPK	1716.7257	1716.7257	3	VIND[HAyneDE]FM[Oxidati	37660	2	TRUE
Insoluble	cysK	A0A5S4TGN0	Cysteine synthase	A0A5S4TGN0_STRPY	TRUE	TRUE	YLSTELYDVPVIK	190.25702	190.25702	2	YLSTELYD[HAyneDE]VPVIK	54379	1	TRUE
Soluble	deoB	A0A5S4TGL8	Phosphopentomutase	A0A5S4TGL8_STRPY	TRUE	TRUE	GFSFTNLVDFDANFGHR	306.96466	306.96466	3	GFSFTNLVD[HAyneDE]FDA	61907	1	TRUE
Soluble	deoB	A0A5S4TGL8	Phosphopentomutase	A0A5S4TGL8_STRPY	TRUE	TRUE	GFSFTNLVDFDANFGHR	334.3815	334.3815	3	GFSFTNLVD[HAyneDE]FDA	61917	1	TRUE
Soluble	deoB	A0A5S4TGL8	Phosphopentomutase	A0A5S4TGL8_STRPY	TRUE	TRUE	GFSFTNLVDFDANFGHR	248.72348	248.72348	4	GFSFTNLVDFD[HAyneDE]A	63544	1	TRUE
Soluble	deoB	A0A5S4TGL8	Phosphopentomutase	A0A5S4TGL8_STRPY	TRUE	TRUE	GFSFTNLVDFDANFGHR	244.65532	244.65532	4	GFSFTNLVDFD[HAyneDE]A	63534	1	TRUE
Soluble	deoB	A0A5S4TGL8	Phosphopentomutase	A0A5S4TGL8_STRPY	TRUE	TRUE	LADAGVPTYAVGKINDIFNGSGITNDMGH	462.78067	462.78067	4	LADAGVPTYAVGKIND[HAyn	72280	2	TRUE
Soluble	deoB	A0A5S4TGL8	Phosphopentomutase	A0A5S4TGL8_STRPY	TRUE	TRUE	LADAGVPTYAVGKINDIFNGSGITNDMGH	978.21405	978.21405	4	LADAGVPTYAVGKIND[HAyn	69659	1	TRUE
Soluble	deoB	A0A5S4TGL8	Phosphopentomutase	A0A5S4TGL8_STRPY	TRUE	TRUE	LADAGVPTYAVGKINDIFNGSGITNDMGH	980.92847	980.92847	4	LADAGVPTYAVGKIND[HAyn	69196	2	TRUE
Soluble	deoB	A0A5S4TGL8	Phosphopentomutase	A0A5S4TGL8_STRPY	TRUE	TRUE	LADAGVPTYAVGKINDIFNGSGITNDMGH	987.376	987.376	4	LADAGVPTYAVGKIND[HAyn	69224	1	FALSE
Soluble	deoB	A0A5S4TGL8	Phosphopentomutase	A0A5S4TGL8_STRPY	TRUE	TRUE	IHLVVLDSVGIGAAPDADK	447.9984	447.9984	3	IHLVVLD[HAyneDE]SVGIG/	45835	1	TRUE
Soluble	deoB	A0A5S4TGL8	Phosphopentomutase	A0A5S4TGL8_STRPY	TRUE	TRUE	IHLVVLDSVGIGAAPDADK	337.87704	337.87704	3	IHLVVLD[HAyneDE]SVGIG/	45848	1	TRUE
Soluble	deoB	A0A5S4TGL8	Phosphopentomutase	A0A5S4TGL8_STRPY	TRUE	TRUE	IHLVVLDSVGIGAAPDADK	215.62378	215.62378	4	IHLVVLD[HAyneDE]SVGIG/	45909	1	TRUE
Soluble	deoB	A0A5S4TGL8	Phosphopentomutase	A0A5S4TGL8_STRPY	TRUE	TRUE	IHLVVLDSVGIGAAPDADK	211.74493	211.74493	4	IHLVVLD[HAyneDE]SVGIG/	45919	1	TRUE
Soluble	deoB	A0A5S4TGL8	Phosphopentomutase	A0A5S4TGL8_STRPY	TRUE	TRUE	IHLVVLDSVGIGAAPDADK	214.33742	214.33742	4	IHLVVLD[HAyneDE]SVGIG/	45930	1	TRUE
Soluble	deoB	A0A5S4TGL8	Phosphopentomutase	A0A5S4TGL8_STRPY	TRUE	TRUE	IHLVVLDSVGIGAAPDADK	215.62378	215.62378	4	IHLVVLD[HAyneDE]SVGIG/	45873	1	FALSE
Soluble	deoB	A0A5S4TGL8	Phosphopentomutase	A0A5S4TGL8_STRPY	TRUE	TRUE	EDDLLLITADHGNDPTYAGTDHTR	378.7372	378.7372	4	EDDLLLITADHGND[HAyne	37155	1	TRUE
Soluble	deoB	A0A5S4TGL8	Phosphopentomutase	A0A5S4TGL8_STRPY	TRUE	TRUE	EDDLLLITADHGNDPTYAGTDHTR	378.6864	378.6864	4	EDDLLLITAD[HAyneDE]HG	37143	1	TRUE
Soluble	deoB	A0A5S4TGL8	Phosphopentomutase	A0A5S4TGL8_STRPY	TRUE	TRUE	EDDLLLITADHGNDPTYAGTDHTR	378.68414	378.68414	4	EDD[HAyneDE]LLLITADHG	37250	1	TRUE
Soluble	deoB	A0A5S4TGL8	Phosphopentomutase	A0A5S4TGL8_STRPY	TRUE	TRUE	EDDLLLITADHGNDPTYAGTDHTR	377.89542	377.89542	4	ED[HAyneDE]DLLLITADHG	37283	1	TRUE
Soluble	dltC	A0A4U7II67	D-alanyl carrier protein	A0A4U7II67_STRPY	TRUE	TRUE	DDWNTVTK	302.0768	302.0768	2	D[HAyneDE]DWNTVTK	26056	1	TRUE
Soluble	dltC	A0A4U7II67	D-alanyl carrier protein	A0A4U7II67_STRPY	TRUE	TRUE	DDWNTVTK	184.81332	184.81332	2	D[HAyneDE]DWNTVTK	24239	1	TRUE
Soluble	dltC	A0A4U7II67	D-alanyl carrier protein	A0A4U7II67_STRPY	TRUE	TRUE	DDWNTVTK	191.81967	191.81967	3	DD[HAyneDE]WNTVTK	26582	1	TRUE
Soluble	dnaG_1	A0A4U9IKA5	RNA polymerase sigma factor SigA	A0A4U9IKA5_STRPY	TRUE	TRUE	LTDGGISITDK	71.33964	71.33964	3	LTD[HAyneDE]GGISITDK	28464	1	TRUE
Soluble	dnaG_1	A0A4U9IKA5	RNA polymerase sigma factor SigA	A0A4U9IKA5_STRPY	TRUE	TRUE	LTDGGISITDK	104.96918	104.96918	3	LTD[HAyneDE]GGISITDK	28419	1	TRUE
Insoluble	dnaK	A0A4U9C4I9	Chaperone protein DnaK	A0A4U9C4I9_STRPY	TRUE	TRUE	FQLTDIPAAPR	1635.811	1635.811	2	FQLTD[HAyneDE]IPAAPR	45101	2	TRUE
Insoluble	dnaK	A0A4U9C4I9	Chaperone protein DnaK	A0A4U9C4I9_STRPY	TRUE	TRUE	SNDGLSEEEIDR	489.03357	489.03357	3	SND[HAyneDE]GLSEEEIDR	26721	1	TRUE
Insoluble	dnaK	A0A4U9C4I9	Chaperone protein DnaK	A0A4U9C4I9_STRPY	TRUE	TRUE	SNDGLSEEEIDR	415.3274	415.3274	2	SND[HAyneDE]GLSEEEIDR	26751	1	TRUE
Insoluble	dnaK	A0A4U9C4I9	Chaperone protein DnaK	A0A4U9C4I9_STRPY	TRUE	TRUE	NEVDQAIFATEK	210.88171	210.88171	2	NEVD[HAyneDE]QAIFATEK	38900	1	TRUE
Insoluble	dnaK	A0A4U9C4I9	Chaperone protein DnaK	A0A4U9C4I9_STRPY	TRUE	TRUE	NEVDQAIFATEK	206.44879	206.44879	2	NEVD[HAyneDE]QAIFATEK	38983	1	TRUE
Insoluble	dnaK	A0A4U9C4I9	Chaperone protein DnaK	A0A4U9C4I9_STRPY	TRUE	TRUE	DAEANAEADAK	86.67515	86.67515	2	D[HAyneDE]AEANAEADAK	12725	2	TRUE
Soluble	dnaK	A0A4U9C4I9	Chaperone protein DnaK	A0A4U9C4I9_STRPY	TRUE	TRUE	QALSDAGLSLSEIDEVILVGGSTR	423.12958	423.12958	3	QALSDAGLSLSEID[HAyneD	72257	1	TRUE
Soluble	dnaK	A0A4U9C4I9	Chaperone protein DnaK	A0A4U9C4I9_STRPY	TRUE	TRUE	QALSDAGLSLSEIDEVILVGGSTR	423.12958	423.12958	3	QALSDAGLSLSEID[HAyneD	72246	1	TRUE
Soluble	dnaK	A0A4U9C4I9	Chaperone protein DnaK	A0A4U9C4I9_STRPY	TRUE	TRUE	QALSDAGLSLSEIDEVILVGGSTR	266.95844	266.95844	4	QALSDAGLSLSEID[HAyneD	72251	1	TRUE
Soluble	dnaK	A0A4U9C4I9	Chaperone protein DnaK	A0A4U9C4I9_STRPY	TRUE	TRUE	SNDGLSEEEIDR	1880.5876	1880.5876	3	SND[HAyneDE]GLSEEEIDR	23151	1	TRUE
Soluble	dnaK	A0A4U9C4I9	Chaperone protein DnaK	A0A4U9C4I9_STRPY	TRUE	TRUE	SNDGLSEEEIDR	1878.1233	1878.1233	3	SND[HAyneDE]GLSEEEIDR	23540	1	TRUE
Soluble	dnaK	A0A4U9C4I9	Chaperone protein DnaK	A0A4U9C4I9_STRPY	TRUE	TRUE	SNDGLSEEEIDR	842.34064	842.34064	2	SND[HAyneDE]GLSEEEIDR	23174	1	TRUE
Soluble	dnaK	A0A4U9C4I9	Chaperone protein DnaK	A0A4U9C4I9_STRPY	TRUE	TRUE	SNDGLSEEEIDR	842.74133	842.74133	2	SND[HAyneDE]GLSEEEIDR	23187	1	TRUE
Soluble	dnaK	A0A4U9C4I9	Chaperone protein DnaK	A0A4U9C4I9_STRPY	TRUE	TRUE	SNDGLSEEEIDR	845.989	845.989	2	SND[HAyneDE]GLSEEEIDR	23661	1	TRUE
Soluble	dnaK	A0A4U9C4I9	Chaperone protein DnaK	A0A4U9C4I9_STRPY	TRUE	TRUE	SNDGLSEEEIDR	846.2744	846.2744	2	SND[HAyneDE]GLSEEEIDR	23569	1	FALSE
Soluble	dnaK	A0A4U9C4I9	Chaperone protein DnaK	A0A4U9C4I9_STRPY	TRUE	TRUE	SNDGLSEEEIDR	846.28046	846.28046	2	SND[HAyneDE]GLSEEEIDR	23627	1	FALSE
Soluble	dnaK	A0A4U9C4I9	Chaperone protein DnaK	A0A4U9C4I9_STRPY	TRUE	TRUE	GYAEDYLGEK	156.06477	156.06477	2	GYAED[HAyneDE]YLGEK	27154	1	TRUE
Soluble	dnaK	A0A4U9C4I9	Chaperone protein DnaK	A0A4U9C4I9_STRPY	TRUE	TRUE	AVITVPAYFNDAQR	1056.4595	1056.4595	3	AVITVPAYFND[HAyneDE]AC	46693	1	TRUE
Soluble	dnaK	A0A4U9C4I9	Chaperone protein DnaK	A0A4U9C4I9_STRPY	TRUE	TRUE	AVITVPAYFNDAQR	695.986	695.986	2	AVITVPAYFND[HAyneDE]AC	46685	1	TRUE
Soluble	dnaK	A0A4U9C4I9	Chaperone protein DnaK	A0A4U9C4I9_STRPY	TRUE	TRUE	GFDTERDAAQSALDELK	250.79506	250.79506	3	GFDTERD[HAyneDE]AAQSA	35162	1	TRUE
Soluble	dnaK	A0A4U9C4I9	Chaperone protein DnaK	A0A4U9C4I9_STRPY	TRUE	TRUE	SVNPDEVVAMGAAIQGGVITGDVK	894.077	894.077	3	SVNPD[HAyneDE]EVVAM(	47365	1	TRUE
Soluble	dnaK	A0A4U9C4I9	Chaperone protein DnaK	A0A4U9C4I9_STRPY	TRUE	TRUE	ENGIDLSQDK	130.85432	130.85432	2	ENGID[HAyneDE]LSQDK	20865	1	TRUE
Soluble	dnaK	A0A4U9C4I9	Chaperone protein DnaK	A0A4U9C4I9_STRPY	TRUE	TRUE	SQVFSTAADNQPAVDIHVLQGERPMAAD	635.0311	635.0311	4	SQVFSTAADNQPAVDIHVL(	32541	1	TRUE
Soluble	dnaK	A0A4U9C4I9	Chaperone protein DnaK	A0A4U9C4I9_STRPY	TRUE	TRUE	SQVFSTAADNQPAVDIHVLQGERPMAAD	621.3201	621.3201	4	SQVFSTAADNQPAVD[HAyn	32609	1	TRUE
Soluble	dnaK	A0A4U9C4I9	Chaperone protein DnaK	A0A4U9C4I9_STRPY	TRUE	TRUE	SQVFSTAADNQPAVDIHVLQGERPMAAD	476.33426	476.33426	4	SQVFSTAAD[HAyneDE]NQI	31711	1	TRUE
Soluble	dnaK	A0A4U9C4I9	Chaperone protein DnaK	A0A4U9C4I9_STRPY	TRUE	TRUE	SQVFSTAADNQPAVDIHVLQGERPMAAD	497.47797	497.47797	4	SQVFSTAAD[HAyneDE]NQI	29980	1	TRUE
Soluble	dnaK	A0A4U9C4I9	Chaperone protein DnaK	A0A4U9C4I9_STRPY	TRUE	TRUE	SQVFSTAADNQPAVDIHVLQGERPMAAD	389.16556	389.16556	5	SQVFSTAADNQPAVDIHVL(	32478	1	TRUE
Soluble	dnaK	A0A4U9C4I9	Chaperone protein DnaK	A0A4U9C4I9_STRPY	TRUE	TRUE	SQVFSTAADNQPAVDIHVLQGERPMAAD	482.5117	482.5117	5	SQVFSTAADNQPAVDIHVL(	30034	1	TRUE
Soluble	dnaK	A0A4U9C4I9	Chaperone protein DnaK	A0A4U9C4I9_STRPY	TRUE	TRUE	SQVFSTAADNQPAVDIHVLQGERPMAAD	389.17264	389.17264	5	SQVFSTAADNQPAVDIHVL(	32559	1	TRUE
Soluble	dnaK	A0A4U9C4I9	Chaperone protein DnaK	A0A4U9C4I9_STRPY	TRUE	TRUE	SQVFSTAADNQPAVDIHVLQGERPMAAD	469.23672	469.23672	5	SQVFSTAADNQPAVDIHVL(	30292	2	FALSE
Soluble	dnaK	A0A4U9C4I9	Chaperone protein DnaK	A0A4U9C4I9_STRPY	TRUE	TRUE	SQVFSTAADNQPAVDIHVLQGERPMAAD	479.15375	479.15375	5	SQVFSTAADNQPAVDIHVL(	29971	1	FALSE
Soluble	dnaK	A0A4U9C4I9	Chaperone protein DnaK	A0A4U9C4I9_STRPY	TRUE	TRUE	IVNEPTAAALAYGMDK	838.7638	838.7638	3	IVNEPTAAALAYGM[Oxidati	32413	1	TRUE
Soluble	dnaK	A0A4U9C4I9	Chaperone protein DnaK	A0A4U9C4I9_STRPY	TRUE	TRUE	IVNEPTAAALAYGMDK	900.4545	900.4545	3	IVNEPTAAALAYGM[Oxidati	32404	1	TRUE
Soluble	dnaK	A0A4U9C4I9	Chaperone protein DnaK	A0A4U9C4I9_STRPY	TRUE	TRUE	IVNEPTAAALAYGMDK	976.2522	976.2522	3	IVNEPTAAALAYGM[Oxidati	33065	1	TRUE
Soluble	dnaK	A0A4U9C4I9	Chaperone protein DnaK	A0A4U9C4I9_STRPY	TRUE	TRUE	IVNEPTAAALAYGMDK	975.6079	975.6079	3	IVNEPTAAALAYGM[Oxidati	33001	1	FALSE
Soluble	dnaK	A0A4U9C4I9	Chaperone protein DnaK	A0A4U9C4I9_STRPY	TRUE	TRUE	IVNEPTAAALAYGMDK	976.2319	976.2319	3	IVNEPTAAALAYGM[Oxidati	32897	1	FALSE
Soluble	dnaK	A0A4U9C4I9	Chaperone protein DnaK	A0A4U9C4I9_STRPY	TRUE	TRUE	IVNEPTAAALAYGMDK	761.56305	761.56305	2	IVNEPTAAALAYGM[Oxidati	32986	1	TRUE
Soluble	dnaK	A0A4U9C4I9	Chaperone protein DnaK	A0A4U9C4I9_STRPY	TRUE	TRUE	IVNEPTAAALAYGMDK	761.56305	761.56305	2	IVNEPTAAALAYGM[Oxidati	33015	1	TRUE
Soluble	dnaK	A0A4U9C4I9	Chaperone protein DnaK	A0A4U9C4I9_STRPY	TRUE	TRUE	IVNEPTAAALAYGMDK	552.07587	552.07587	4	IVNEPTAAALAYGM[Oxidati	32978	1	TRUE
Soluble	dnaK	A0A4U9C4I9	Chaperone protein DnaK	A0A4U9C4I9_STRPY	TRUE	TRUE	IVNEPTAAALAYGMDK	548.22156	548.22156	4	IVNEPTAAALAYGM[Oxidati	32961	1	TRUE
Soluble	dnaK	A0A4U9C4I9	Chaperone protein DnaK	A0A4U9C4I9_STRPY	TRUE	TRUE	NEVDQAIFATEK	515.36755	515.36755	2	NEVD[HAyneDE]QAIFATEK	34662	1	TRUE
Soluble	dnaK	A0A4U9C4I9	Chaperone protein DnaK	A0A4U9C4I9_STRPY	TRUE	TRUE	NEVDQAIFATEK	515.36755	515.36755	2	NEVD[HAyneDE]QAIFATEK	34653	1	TRUE
Soluble	dnaK	A0A4U9C4I9	Chaperone protein DnaK	A0A4U9C4I9_STRPY	TRUE	TRUE	NEVDQAIFATEK	446.9815	446.9815	3	NEVD[HAyneDE]QAIFATEK	34865	1	TRUE
Soluble	dnaK	A0A4U9C4I9	Chaperone protein DnaK	A0A4U9C4I9_STRPY	TRUE	TRUE	NEVDQAIFATEK	458.35205	458.35205	3	NEVD[HAyneDE]QAIFATEK	34682	1	TRUE
Soluble	dnaK	A0A4U9C4I9	Chaperone protein DnaK	A0A4U9C4I9_STRPY	TRUE	TRUE	NEVDQAIFATEK	468.23672	468.23672	3	NEVD[HAyneDE]QAIFATEK	34877	1	TRUE
Soluble	dnaK	A0A4U9C4I9	Chaperone protein DnaK	A0A4U9C4I9_STRPY	TRUE	TRUE	IIDFLVAEFKK	188.3964	188.3964	3	IID[HAyneDE]FLVAEFKK	53448	1	TRUE
Soluble	dnaK	A0A4U9C4I9	Chaperone protein DnaK	A0A4U9C4I9_STRPY	TRUE	TRUE	IIDFLVAEFKK	141.32376	141.32376	3	IID[HAyneDE]FLVAEFKK	53461	1	TRUE
Soluble	dnaK	A0A4U9C4I9	Chaperone protein DnaK	A0A4U9C4I9_STRPY	TRUE	TRUE	FQLTDIPAAPR	521.7556	521.7556	3	FQLTD[HAyneDE]IPAAPR	40952	1	TRUE
Soluble	dnaK	A0A4U9C4I9	Chaperone protein DnaK	A0A4U9C4I9_STRPY	TRUE	TRUE	DAEANAEADAK	285.99274	285.99274	2	D[HAyneDE]AEANAEADAK	10765	1	TRUE
Soluble	dnaK	A0A4U9C4I9	Chaperone protein DnaK	A0A4U9C4I9_STRPY	TRUE	TRUE	DAEANAEADAK	285.9467	285.9467	2	D[HAyneDE]AEANAEADAK	10753	1	TRUE
Soluble	dnaK	A0A4U9C4I9	Chaperone protein DnaK	A0A4U9C4I9_STRPY	TRUE	TRUE	DAEANAEADAK	272.7775	272.7775	3	D[HAyneDE]AEANAEADAK	10816	1	TRUE
Soluble	dnaK	A0A4U9C4I9	Chaperone protein DnaK	A0A4U9C4I9_STRPY	TRUE	TRUE	DAEANAEADAK	735.0936	735.0936	3	D[HAyneDE]AEANAEADAK	10023	1	TRUE
Soluble	dnaK	A0A4U9C4I9	Chaperone protein DnaK	A0A4U9C4I9_STRPY	TRUE	TRUE	GIPQIEVTFDIDK	201.77477	201.77477	2	GIPQIEVTFD[HAyneDE]IDK	55363	1	TRUE
Soluble	dnaK	A0A4U9C4I9	Chaperone protein DnaK	A0A4U9C4I9_STRPY	TRUE	TRUE	GIPQIEVTFDIDK	201.77477	201.77477	2	GIPQIEVTFD[HAyneDE]IDK	55351	1	TRUE
Soluble	dnaK	A0A4U9C4I9	Chaperone protein DnaK	A0A4U9C4I9_STRPY	TRUE	TRUE	GIPQIEVTFDIDK	198.75166	198.75166	2	GIPQIEVTFD[HAyneDE]IDK	55318	1	TRUE
Soluble	dnaK	A0A4U9C4I9	Chaperone protein DnaK	A0A4U9C4I9_STRPY	TRUE	TRUE	LGGDDFDQK	1082.3073	1082.3073	3	LGGD[HAyneDE]DFDQK	23113	1	TRUE
Soluble	dnaK	A0A4U9C4I9	Chaperone protein DnaK	A0A4U9C4I9_STRPY	TRUE	TRUE	LGGDDFDQK	239.04515	239.04515	3	LGGD[HAyneDE]DFDQK	21790	1	TRUE
Soluble	dnaK	A0A4U9C4I9	Chaperone protein DnaK	A0A4U9C4I9_STRPY	TRUE	TRUE	LGGDDFDQK	851.7095	851.7095	2	LGGDDFD[HAyneDE]QK	22707	1	TRUE
Soluble	dnaK	A0A4U9C4I9	Chaperone protein DnaK	A0A4U9C4I9_STRPY	TRUE	TRUE	LIDRNTTIPTSK	264.68958	264.68958	4	LID[HAyneDE]RNTTIPTSK	16546	2	TRUE
Soluble	dnaK	A0A4U9C4I9	Chaperone protein DnaK	A0A4U9C4I9_STRPY	TRUE	TRUE	DAAQSALDELK	368.24078	368.24078	3	D[HAyneDE]AAQSALDELK	31045	2	TRUE
Soluble	dnaK	A0A4U9C4I9	Chaperone protein DnaK	A0A4U9C4I9_STRPY	TRUE	TRUE	DAAQSALDELK	340.91013	340.91013	2	D[HAyneDE]AAQSALDELK	40572	1	TRUE
Soluble	dnaK	A0A4U9C4I9	Chaperone protein DnaK	A0A4U9C4I9_STRPY	TRUE	TRUE	SKIIGIDLGTTNSAVAVLEGTESK	351.71643	351.71643	3	[Acetyl (Protein N-term)]SK	55569	1	TRUE
Insoluble	E0F66_00335	A0A4U7H3E7	Pyruvate dehydrogenase E1 component s	A0A4U7H3E7_STRPY	FALSE	FALSE	LASEDVPVPYAR	149.94833	149.94833	2	LASED[HAyneDE]VPVPYAR	36935	1	TRUE
Soluble	E0F66_00335	A0A4U7H3E7	Pyruvate dehydrogenase E1 component s	A0A4U7H3E7_STRPY	TRUE	TRUE	KEEVNQDPDFYIPLGK	108.3223	108.3223	4	KEEVNQD[HAyneDE]PDFYI	40920	1	TRUE
Soluble	E0F66_00335	A0A4U7H3E7	Pyruvate dehydrogenase E1 component s	A0A4U7H3E7_STRPY	TRUE	TRUE	KEEVNQDPDFYIPLGK	168.07454	168.07454	4	KEEVNQD[HAyneDE]PDFYI	39419	2	TRUE
Soluble	E0F66_00335	A0A4U7H3E7	Pyruvate dehydrogenase E1 component s	A0A4U7H3E7_STRPY	FALSE	FALSE	LASEDVPVPYAR	125.39426	125.39426	2	LASED[HAyneDE]VPVPYAR	32878	1	TRUE
Soluble	E0F66_00335	A0A4U7H3E7	Pyruvate dehydrogenase E1 component s	A0A4U7H3E7_STRPY	FALSE	FALSE	LASEDVPVPYAR	305.43195	305.43195	3	LASED[HAyneDE]VPVPYAR	31268	2	TRUE
Soluble	E0F66_00335	A0A4U7H3E7	Pyruvate dehydrogenase E1 component s	A0A4U7H3E7_STRPY	FALSE	FALSE	LASEDVPVPYAR	305.40927	305.40927	3	LASED[HAyneDE]VPVPYAR	31256	2	TRUE
Soluble	E0F66_01350	A0A4U7IIM9	Flavodoxin	A0A4U7IIM9_STRPY	TRUE	TRUE	SVDDFSEQFALTGAIK	306.14362	306.14362	3	SVDD[HAyneDE]FSEQFALT	53181	3	TRUE
Soluble	E0F66_01350	A0A4U7IIM9	Flavodoxin	A0A4U7IIM9_STRPY	TRUE	TRUE	IVYASMTGNTEEIADIVANK	258.1121	258.1121	3	IVYASM[Oxidation (M)]TGN	41906	2	TRUE
Soluble	E0F66_04035	A0A4U7HZV3	DUF4298 domain-containing protein	A0A4U7HZV3_STRPY	TRUE	TRUE	QDQLIVEK	335.34464	335.34464	3	QD[HAyneDE]QLIVEK	26958	1	TRUE
Soluble	E0F66_04035	A0A4U7HZV3	DUF4298 domain-containing protein	A0A4U7HZV3_STRPY	TRUE	TRUE	QDQLIVEK	335.34464	335.34464	3	QD[HAyneDE]QLIVEK	26945	1	TRUE
Soluble	E0F66_04365	A0A4U7HCJ0	UPF0297 protein E0F66_04365	A0A4U7HCJ0_STRPY	TRUE	TRUE	DEIVEELVR	407.544	407.544	2	D[HAyneDE]EIVEELVR	49339	1	TRUE
Soluble	E0F66_04365	A0A4U7HCJ0	UPF0297 protein E0F66_04365	A0A4U7HCJ0_STRPY	TRUE	TRUE	DEIVEELVR	345.98895	345.98895	3	D[HAyneDE]EIVEELVR	49369	3	TRUE
Soluble	E0F66_04365	A0A4U7HCJ0	UPF0297 protein E0F66_04365	A0A4U7HCJ0_STRPY	TRUE	TRUE	DEIVEELVR	336.42816	336.42816	3	D[HAyneDE]EIVEELVR	49437	1	TRUE
Soluble	E0F66_04365	A0A4U7HCJ0	UPF0297 protein E0F66_04365	A0A4U7HCJ0_STRPY	TRUE	TRUE	GFTDETVR	318.4969	318.4969	3	[Acetyl (Protein N-term)]GI	30407	1	TRUE
Soluble	E0F66_04365	A0A4U7HCJ0	UPF0297 protein E0F66_04365	A0A4U7HCJ0_STRPY	TRUE	TRUE	GFTDETVR	181.04155	181.04155	2	GFTD[HAyneDE]ETVR	21098	1	TRUE
Soluble	E0F66_04365	A0A4U7HCJ0	UPF0297 protein E0F66_04365	A0A4U7HCJ0_STRPY	TRUE	TRUE	LDDGDKR	282.19443	282.19443	3	LDD[HAyneDE]GDKR	8083	1	TRUE
Insoluble	E0F66_04905	A0A4U7GIT6	Stress response regulator gls24 homolog	A0A4U7GIT6_STRPY	TRUE	TRUE	GQLTYDDK	293.9424	293.9424	2	GQLTYDD[HAyneDE]K	20844	1	TRUE

Soluble	E0F66_04905	A0A4U7GIT6	Stress response regulator gls24 homolog	A0A4U7GIT6_STRPY	TRUE	TRUE	RMTDLDVIEVNVK	1466.3973	1466.3973	3	RM[Oxidation (M)]TDLD[HA	31848	1	TRUE
Soluble	E0F66_04905	A0A4U7GIT6	Stress response regulator gls24 homolog	A0A4U7GIT6_STRPY	TRUE	TRUE	RMTDLDVIEVNVK	1188.5522	1188.5522	4	RM[Oxidation (M)]TDLD[HA	31565	1	TRUE
Soluble	E0F66_04905	A0A4U7GIT6	Stress response regulator gls24 homolog	A0A4U7GIT6_STRPY	TRUE	TRUE	RMTDLDVIEVNVK	1173.2666	1173.2666	4	RM[Oxidation (M)]TD[HAyn	31602	1	TRUE
Soluble	E0F66_04905	A0A4U7GIT6	Stress response regulator gls24 homolog	A0A4U7GIT6_STRPY	TRUE	TRUE	RMTDLDVIEVNVK	1169.3021	1169.3021	4	RM[Oxidation (M)]TD[HAyn	31539	2	TRUE
Soluble	E0F66_04905	A0A4U7GIT6	Stress response regulator gls24 homolog	A0A4U7GIT6_STRPY	TRUE	TRUE	RMTDLDVIEVNVK	1169.2969	1169.2969	4	RM[Oxidation (M)]TDLD[HA	31855	1	TRUE
Soluble	E0F66_04905	A0A4U7GIT6	Stress response regulator gls24 homolog	A0A4U7GIT6_STRPY	TRUE	TRUE	RMTDLDVIEVNVK	1188.5522	1188.5522	4	RM[Oxidation (M)]TDLD[HA	31588	1	TRUE
Soluble	E0F66_04905	A0A4U7GIT6	Stress response regulator gls24 homolog	A0A4U7GIT6_STRPY	TRUE	TRUE	QVAVDLDIVAEYQK	453.3785	453.3785	2	QVAVD[HAyneDE]LDIVAEY	48227	1	TRUE
Soluble	E0F66_04905	A0A4U7GIT6	Stress response regulator gls24 homolog	A0A4U7GIT6_STRPY	TRUE	TRUE	QVAVDLDIVAEYQK	374.6451	374.6451	3	QVAVDLD[HAyneDE]IVAEY	49603	1	TRUE
Soluble	E0F66_04905	A0A4U7GIT6	Stress response regulator gls24 homolog	A0A4U7GIT6_STRPY	TRUE	TRUE	QVAVDLDIVAEYQK	332.9521	332.9521	3	QVAVD[HAyneDE]LDIVAEY	48411	1	TRUE
Soluble	E0F66_04905	A0A4U7GIT6	Stress response regulator gls24 homolog	A0A4U7GIT6_STRPY	TRUE	TRUE	GQLTYDDK	535.1921	535.1921	2	GQLTYDD[HAyneDE]K	17317	1	TRUE
Soluble	E0F66_04905	A0A4U7GIT6	Stress response regulator gls24 homolog	A0A4U7GIT6_STRPY	TRUE	TRUE	GQLTYDDK	607.00635	607.00635	2	GQLTYDD[HAyneDE]K	18002	1	TRUE
Soluble	E0F66_04905	A0A4U7GIT6	Stress response regulator gls24 homolog	A0A4U7GIT6_STRPY	TRUE	TRUE	GQLTYDDK	574.2569	574.2569	2	GQLTYDD[HAyneDE]K	17327	1	TRUE
Soluble	E0F66_04905	A0A4U7GIT6	Stress response regulator gls24 homolog	A0A4U7GIT6_STRPY	TRUE	TRUE	GQLTYDDK	640.0592	640.0592	2	GQLTYD[HAyneDE]DK	17266	1	TRUE
Soluble	E0F66_04905	A0A4U7GIT6	Stress response regulator gls24 homolog	A0A4U7GIT6_STRPY	TRUE	TRUE	GQLTYDDK	657.4085	657.4085	2	GQLTYD[HAyneDE]DK	17129	1	TRUE
Soluble	E0F66_04905	A0A4U7GIT6	Stress response regulator gls24 homolog	A0A4U7GIT6_STRPY	TRUE	TRUE	DLTSAIR	311.016	311.016	2	D[HAyneDE]LTSAIR	24435	1	TRUE
Soluble	E0F66_04905	A0A4U7GIT6	Stress response regulator gls24 homolog	A0A4U7GIT6_STRPY	TRUE	TRUE	DLTSAIR	211.77539	211.77539	3	D[HAyneDE]LTSAIR	24446	1	TRUE
Soluble	E0F66_04905	A0A4U7GIT6	Stress response regulator gls24 homolog	A0A4U7GIT6_STRPY	TRUE	TRUE	MTDLDVIEVNVK	562.9626	562.9626	3	M[Oxidation (M)]TDLD[HA	42574	1	TRUE
Soluble	E0F66_04905	A0A4U7GIT6	Stress response regulator gls24 homolog	A0A4U7GIT6_STRPY	TRUE	TRUE	MTDLDVIEVNVK	562.9352	562.9352	3	M[Oxidation (M)]TDLD[HA	42586	1	TRUE
Soluble	E0F66_04905	A0A4U7GIT6	Stress response regulator gls24 homolog	A0A4U7GIT6_STRPY	TRUE	TRUE	MTDLDVIEVNVK	446.82574	446.82574	2	M[Oxidation (M)]TDLD[HA	42521	1	TRUE
Soluble	E0F66_04905	A0A4U7GIT6	Stress response regulator gls24 homolog	A0A4U7GIT6_STRPY	TRUE	TRUE	MTDLDVIEVNVK	440.73254	440.73254	2	M[Oxidation (M)]TD[HAyne	42311	1	TRUE
Soluble	E0F66_04905	A0A4U7GIT6	Stress response regulator gls24 homolog	A0A4U7GIT6_STRPY	TRUE	TRUE	DGVNVEVGKK	318.7986	318.7986	3	D[HAyneDE]GVNVEVGKK	13229	1	TRUE
Soluble	E0F66_04905	A0A4U7GIT6	Stress response regulator gls24 homolog	A0A4U7GIT6_STRPY	TRUE	TRUE	DGVNVEVGK	272.16638	272.16638	3	D[HAyneDE]GVNVEVGK	20754	1	TRUE
Soluble	E0F66_04905	A0A4U7GIT6	Stress response regulator gls24 homolog	A0A4U7GIT6_STRPY	TRUE	TRUE	DGVNVEVGK	285.8189	285.8189	3	D[HAyneDE]GVNVEVGK	20722	1	TRUE
Soluble	E0F66_06710	A0A4Q1PWI7	DAK2 domain-containing protein	A0A4Q1PWI7_STRPY	TRUE	TRUE	VHVHTEDPGLVMQEGLK	325.74396	325.74396	4	VHVHTED[HAyneDE]PGLVI	16408	1	TRUE
Soluble	E0F66_06710	A0A4Q1PWI7	DAK2 domain-containing protein	A0A4Q1PWI7_STRPY	TRUE	TRUE	VHVHTEDPGLVMQEGLK	331.71732	331.71732	4	VHVHTED[HAyneDE]PGLVI	16416	1	TRUE
Soluble	E0F66_06710	A0A4Q1PWI7	DAK2 domain-containing protein	A0A4Q1PWI7_STRPY	TRUE	TRUE	DTTIDGLEIHENDFLGMVDGK	363.53058	363.53058	3	DTTIDGLEIHENDFLGM[Oxi	52186	1	TRUE
Insoluble	E0F66_07220	A0A4U7G861	MarR family transcriptional regulator	A0A4U7G861_STRPY	TRUE	TRUE	NMVGHVIADMSDEEMQALVR	396.70367	396.70367	4	NMVGHVIADMSD[HAyneD	32127	2	TRUE
Soluble	E0F66_08175	A0A4U7I7S5	PepSY domain-containing protein	A0A4U7I7S5_STRPY	TRUE	TRUE	DDHMDNQPK	281.9788	281.9788	3	DD[HAyneDE]HM[Oxidatio	6634	1	TRUE
Soluble	E0F66_08175	A0A4U7I7S5	PepSY domain-containing protein	A0A4U7I7S5_STRPY	TRUE	TRUE	DDHMDNQPK	1324.4585	1324.4585	3	DD[HAyneDE]HM[Oxidatio	5893	1	TRUE
Insoluble	E0F67_00060	A0A5S4TG77	Amino acid ABC transporter ATP-binding	A0A5S4TG77_STRPY	TRUE	TRUE	DLAEQGMTMLIVTHEMGFAR	270.02997	270.02997	3	D[HAyneDE]LAEQGM[Oxid	38398	1	TRUE
Insoluble	E0F67_00060	A0A5S4TG77	Amino acid ABC transporter ATP-binding	A0A5S4TG77_STRPY	TRUE	TRUE	LIEFLDK	267.6885	267.6885	3	LIEFLD[HAyneDE]K	48488	1	TRUE
Insoluble	E0F67_00060	A0A5S4TG77	Amino acid ABC transporter ATP-binding	A0A5S4TG77_STRPY	TRUE	TRUE	LIEFLDK	270.72763	270.72763	3	LIEFLD[HAyneDE]K	48503	1	TRUE
Insoluble	E0F67_00060	A0A5S4TG77	Amino acid ABC transporter ATP-binding	A0A5S4TG77_STRPY	TRUE	TRUE	LIEFLDK	181.03714	181.03714	2	LIEFLD[HAyneDE]K	48603	1	TRUE
Insoluble	E0F67_00060	A0A5S4TG77	Amino acid ABC transporter ATP-binding	A0A5S4TG77_STRPY	TRUE	TRUE	LIEFLDK	182.38496	182.38496	2	LIEFLD[HAyneDE]K	48552	1	TRUE
Insoluble	E0F67_00060	A0A5S4TG77	Amino acid ABC transporter ATP-binding	A0A5S4TG77_STRPY	TRUE	TRUE	LIEFLDK	181.03714	181.03714	2	LIEFLD[HAyneDE]K	48591	1	TRUE
Insoluble	E0F67_00065	A0A5S4TFX5	Transporter substrate-binding domain-co	A0A5S4TFX5_STRPY	TRUE	TRUE	YVGIDIELIK	215.87885	215.87885	2	YVGID[HAyneDE]IELIK	57199	1	TRUE
Insoluble	E0F67_00065	A0A5S4TFX5	Transporter substrate-binding domain-co	A0A5S4TFX5_STRPY	TRUE	TRUE	YVGIDIELIK	213.45668	213.45668	2	YVGID[HAyneDE]IELIK	57153	1	TRUE
Insoluble	E0F67_00065	A0A5S4TFX5	Transporter substrate-binding domain-co	A0A5S4TFX5_STRPY	TRUE	TRUE	YVGIDIELIK	152.62508	152.62508	3	YVGID[HAyneDE]IELIK	59787	1	TRUE
Insoluble	E0F67_00065	A0A5S4TFX5	Transporter substrate-binding domain-co	A0A5S4TFX5_STRPY	TRUE	TRUE	DSDQIYK	641.201	641.201	2	D[HAyneDE]SDQIYK	19529	1	TRUE
Insoluble	E0F67_00295	A0A5S4TKH7	Stress response regulator gls24 homolog	A0A5S4TKH7_STRPY	TRUE	TRUE	AIVSQNVDSMTHLK	175.50381	175.50381	3	AIVSQNVD[HAyneDE]SM[C	18286	3	TRUE
Soluble	E0F67_00295	A0A5S4TKH7	Stress response regulator gls24 homolog	A0A5S4TKH7_STRPY	TRUE	TRUE	AIVSQNVDSMTHLK	685.87726	685.87726	3	AIVSQNVD[HAyneDE]SM[C	15876	1	TRUE
Soluble	E0F67_00295	A0A5S4TKH7	Stress response regulator gls24 homolog	A0A5S4TKH7_STRPY	TRUE	TRUE	AIVSQNVDSMTHLK	683.34863	683.34863	3	AIVSQNVD[HAyneDE]SM[C	15952	1	TRUE
Soluble	E0F67_00295	A0A5S4TKH7	Stress response regulator gls24 homolog	A0A5S4TKH7_STRPY	TRUE	TRUE	AIVSQNVDSMTHLK	319.24844	319.24844	4	AIVSQNVD[HAyneDE]SM[C	15451	1	TRUE
Soluble	E0F67_00295	A0A5S4TKH7	Stress response regulator gls24 homolog	A0A5S4TKH7_STRPY	TRUE	TRUE	STLTYDDK	412.64557	412.64557	2	STLTYD[HAyneDE]DK	18065	1	TRUE
Soluble	E0F67_00295	A0A5S4TKH7	Stress response regulator gls24 homolog	A0A5S4TKH7_STRPY	TRUE	TRUE	STLTYDDK	409.23508	409.23508	2	STLTYDD[HAyneDE]K	18874	1	TRUE
Soluble	E0F67_00295	A0A5S4TKH7	Stress response regulator gls24 homolog	A0A5S4TKH7_STRPY	TRUE	TRUE	STLTYDDK	380.69012	380.69012	2	STLTYDD[HAyneDE]K	18898	1	TRUE
Soluble	E0F67_00295	A0A5S4TKH7	Stress response regulator gls24 homolog	A0A5S4TKH7_STRPY	TRUE	TRUE	TKEEHEAASVTVQDR	201.19179	201.19179	4	TKEEHEAASVTVQD[HAyneI	8771	1	TRUE
Insoluble	E0F67_00455	A0A5S4TMC9	Phosphoglucomutase	A0A5S4TMC9_STRPY	TRUE	TRUE	QFNNTDIVLLEDFQK	249.0231	249.0231	3	QFNNTD[HAyneDE]IVLLED	62158	1	TRUE
Insoluble	E0F67_01215	A0A5S4TF76	Dihydrolipoamide acetyltransferase comp	A0A5S4TF76_STRPY	TRUE	TRUE	FVNLGIAVGLDDGLIVPVIHGADK	298.07565	298.07565	4	FVNLGIAVGLD[HAyneDE]D	74174	1	TRUE
Soluble	E0F67_01215	A0A5S4TF76	Dihydrolipoamide acetyltransferase comp	A0A5S4TF76_STRPY	FALSE	FALSE	LGVDMQEGEIIEWK	711.1861	711.1861	3	LGVD[HAyneDE]M[Oxidati	44830	1	TRUE
Soluble	E0F67_01215	A0A5S4TF76	Dihydrolipoamide acetyltransferase comp	A0A5S4TF76_STRPY	TRUE	TRUE	FVNLGIAVGLDDGLIVPVIHGADK	315.38205	315.38205	3	FVNLGIAVGLD[HAyneDE]D	69962	1	TRUE
Soluble	E0F67_01275	A0A5S4TF86	Tetratricopeptide repeat protein	A0A5S4TF86_STRPY	TRUE	TRUE	MIASLDQQDLAHAEK	1664.05	1664.05	3	M[Oxidation (M)]IASLDQQ	20696	1	TRUE
Soluble	E0F67_01275	A0A5S4TF86	Tetratricopeptide repeat protein	A0A5S4TF86_STRPY	TRUE	TRUE	MIASLDQQDLAHAEK	168.53758	168.53758	4	M[Oxidation (M)]IASLD[HA	20821	1	TRUE
Insoluble	E0F67_01945	A0A5S4TGT8	Alpha-L-Rha alpha-1,3-L-rhamnosyltrans	A0A5S4TGT8_STRPY	TRUE	TRUE	NHCLVDEFLQR	1801.448	1801.448	3	NHC[Carbamidomethyl (C	60144	1	TRUE
Insoluble	E0F67_01945	A0A5S4TGT8	Alpha-L-Rha alpha-1,3-L-rhamnosyltrans	A0A5S4TGT8_STRPY	TRUE	TRUE	NHCLVDEFLQR	603.6605	603.6605	2	NHC[Carbamidomethyl (C	60148	1	TRUE
Insoluble	E0F67_01945	A0A5S4TGT8	Alpha-L-Rha alpha-1,3-L-rhamnosyltrans	A0A5S4TGT8_STRPY	TRUE	TRUE	NHCLVDEFLQR	404.37057	404.37057	4	NHC[Carbamidomethyl (C	60145	1	TRUE
Insoluble	E0F67_01945	A0A5S4TGT8	Alpha-L-Rha alpha-1,3-L-rhamnosyltrans	A0A5S4TGT8_STRPY	TRUE	TRUE	NHCLVDEFLQR	404.38376	404.38376	4	NHC[Carbamidomethyl (C	60187	1	TRUE
Insoluble	E0F67_02685	A0A5S4TFZ0	LacI family transcriptional regulator	A0A5S4TFZ0_STRPY	TRUE	TRUE	DIAELAK	369.19974	369.19974	2	D[HAyneDE]IAELAK	22483	2	TRUE
Soluble	E0F67_02795	A0A5S4THH7	Transcriptional coactivator p15 (PC4) C-t	A0A5S4THH7_STRPY	TRUE	TRUE	TWSPDHTK	469.52295	469.52295	3	TWSPD[HAyneDE]HTK	13132	1	TRUE
Insoluble	E0F67_03135	A0A5S4TG64	Aminopeptidase P family protein	A0A5S4TG64_STRPY	TRUE	TRUE	TIYAEFDHLNVNK	195.96684	195.96684	3	TIYAEFD[HAyneDE]HLNVN	36099	1	TRUE
Insoluble	E0F67_03135	A0A5S4TG64	Aminopeptidase P family protein	A0A5S4TG64_STRPY	TRUE	TRUE	TIYAEFDHLNVNK	195.96684	195.96684	3	TIYAEFD[HAyneDE]HLNVN	36136	1	TRUE
Insoluble	E0F67_03135	A0A5S4TG64	Aminopeptidase P family protein	A0A5S4TG64_STRPY	TRUE	TRUE	SADEINK	268.31393	268.31393	2	SAD[HAyneDE]EINK	15547	1	TRUE
Insoluble	E0F67_04385	A0A5S4TFD9	Endopeptidase	A0A5S4TFD9_STRPY	TRUE	TRUE	VIDESLSLVENAQNLAK	293.1736	293.1736	3	VID[HAyneDE]ESLSLVENA	55311	1	TRUE
Insoluble	E0F67_04385	A0A5S4TFD9	Endopeptidase	A0A5S4TFD9_STRPY	TRUE	TRUE	VIDESLSLVENAQNLAK	159.45074	159.45074	2	VID[HAyneDE]ESLSLVENA	55343	1	TRUE
Insoluble	E0F67_04475	A0A5S4TGY9	ATP-dependent Clp protease ATP-binding	A0A5S4TGY9_STRPY	TRUE	TRUE	ALAEVLFDDEAALIR	218.87817	218.87817	3	ALAEVLFDD[HAyneDE]EAA	70692	1	TRUE
Soluble	E0F67_04655	A0A4U9C0G5	ABC transporter	A0A4U9C0G5_STRPY	TRUE	TRUE	VVDGEEVTEYTTK	546.45593	546.45593	3	VVD[HAyneDE]GEEVTEYTTI	23168	1	TRUE
Soluble	E0F67_04655	A0A4U9C0G5	ABC transporter	A0A4U9C0G5_STRPY	TRUE	TRUE	VVDGEEVTEYTTK	511.6887	511.6887	3	VVD[HAyneDE]GEEVTEYTTI	22950	1	TRUE
Soluble	E0F67_04655	A0A4U9C0G5	ABC transporter	A0A4U9C0G5_STRPY	TRUE	TRUE	VVDGEEVTEYTTK	285.0197	285.0197	2	VVD[HAyneDE]GEEVTEYTTI	23179	1	TRUE
Soluble	E0F67_04655	A0A4U9C0G5	ABC transporter	A0A4U9C0G5_STRPY	TRUE	TRUE	VVDGEEVTEYTTK	287.2854	287.2854	2	VVD[HAyneDE]GEEVTEYTTI	22912	1	TRUE
Soluble	E0F67_04655	A0A4U9C0G5	ABC transporter	A0A4U9C0G5_STRPY	TRUE	TRUE	DAGGSGAPAK	307.8401	307.8401	3	D[HAyneDE]AGGSGAPAK	7878	1	TRUE
Soluble	E0F67_04655	A0A4U9C0G5	ABC transporter	A0A4U9C0G5_STRPY	TRUE	TRUE	DAGGSGAPAK	188.34752	188.34752	2	D[HAyneDE]AGGSGAPAK	7860	1	TRUE
Soluble	E0F67_04655	A0A4U9C0G5	ABC transporter	A0A4U9C0G5_STRPY	TRUE	TRUE	DAGGSGAPAK	188.03618	188.03618	2	D[HAyneDE]AGGSGAPAK	7924	2	TRUE
Soluble	E0F67_04655	A0A4U9C0G5	ABC transporter	A0A4U9C0G5_STRPY	TRUE	TRUE	DAGGSGAPAK	187.63818	187.63818	2	D[HAyneDE]AGGSGAPAK	7905	1	TRUE
Insoluble	E0F67_04720	A0A5S4TF85	C5a peptidase	A0A5S4TF85_STRPY	TRUE	TRUE	NYNNDLASTLGSTR	1771.8545	1771.8545	3	NYNND[HAyneDE]LASTLGS	36403	2	TRUE
Soluble	E0F67_04965	A0A5S4TG01	SseB family protein	A0A5S4TG01_STRPY	TRUE	TRUE	DDNHYDR	420.05966	420.05966	3	DD[HAyneDE]NHYDR	9047	1	TRUE
Insoluble	E0F67_05010	A0A5S4TH93	DegV family protein	A0A5S4TH93_STRPY	TRUE	TRUE	TYFLVDDLFHLMR	491.93152	491.93152	3	TYFLVDD[HAyneDE]LFHLM	67565	1	TRUE
Insoluble	E0F67_05010	A0A5S4TH93	DegV family protein	A0A5S4TH93_STRPY	TRUE	TRUE	TYFLVDDLFHLMR	252.33392	252.33392	3	TYFLVDD[HAyneDE]LFHLM	67553	1	TRUE
Insoluble	E0F67_05790	A0A5S4TDR2	Nicotinate phosphoribosyltransferase	A0A5S4TDR2_STRPY	TRUE	TRUE	DCVFLVDTYDTLK	202.23827	202.23827	3	D[HAyneDE]C[Carbamidom	75521	1	TRUE
Insoluble	E0F67_05930	A0A5S4TJP2	Bifunctional Cof-type HAD-IIB family hyd	A0A5S4TJP2_STRPY	TRUE	TRUE	GDGLQLPAELAEHLR	420.70056	420.70056	3	GD[HAyneDE]GLQLPAELAI	55182	1	TRUE
Soluble	E0F67_06385	A0A4U7GDP0	DivIVA domain-containing protein	A0A4U7GDP0_STRPY	TRUE	TRUE	TVLNEDIPESDDSASFDATR	521.74097	521.74097	3	TVLNED[HAyneDE]IPESDD	36123	1	TRUE
Soluble	E0F67_06385	A0A4U7GDP0	DivIVA domain-containing protein	A0A4U7GDP0_STRPY	TRUE	TRUE	TVLNEDIPESDDSASFDATR	521.74097	521.74097	3	TVLNEDIPESDD[HAyneDE]	36155	1	TRUE
Soluble	E0F67_06385	A0A4U7GDP0	DivIVA domain-containing protein	A0A4U7GDP0_STRPY	TRUE	TRUE	TVLNEDIPESDDSASFDATR	282.2236	282.2236	2	TVLNEDIPESDD[HAyneDE]	36223	1	TRUE
Soluble	E0F67_06385	A0A4U7GDP0	DivIVA domain-containing protein	A0A4U7GDP0_STRPY	TRUE	TRUE	TVLNEDIPESDDSASFDATR	204.34697	204.34697	2	TVLNED[HAyneDE]IPESDD	37907	1	TRUE
Soluble	E0F67_06385	A0A4U7GDP0	DivIVA domain-containing protein	A0A4U7GDP0_STRPY	TRUE	TRUE	ATYDAQHLLDESK	554.2147	554.2147	3	ATYDAQHLLD[HAyneDE]ES	28263	1	TRUE
Soluble	E0F67_06385	A0A4U7GDP0	DivIVA domain-containing protein	A0A4U7GDP0_STRPY	TRUE	TRUE	ATYDAQHLLDESK	518.7684	518.7684	3	ATYD[HAyneDE]AQHLLDES	21868	1	TRUE
Soluble	E0F67_06385	A0A4U7GDP0	DivIVA domain-containing protein	A0A4U7GDP0_STRPY	TRUE	TRUE	ATYDAQHLLDESK	547.5333	547.5333	3	ATYD[HAyneDE]AQHLLDES	20949	1	TRUE
Soluble	E0F67_06385	A0A4U7GDP0	DivIVA domain-containing protein	A0A4U7GDP0_STRPY	TRUE	TRUE	ATYDAQHLLDESK	547.91205	547.91205	3	ATYD[HAyneDE]AQHLLDES	20940	1	TRUE
Soluble	E0F67_06385	A0A4U7GDP0	DivIVA domain-containing protein	A0A4U7GDP0_STRPY	TRUE	TRUE	ATYDAQHLLDESK	99.47052	99.47052	2	ATYD[HAyneDE]AQHLLDES	21926	1	TRUE
Soluble	E0F67_06385	A0A4U7GDP0	DivIVA domain-containing protein	A0A4U7GDP0_STRPY	TRUE	TRUE	ATYDAQHLLDESK	101.70165	101.70165	2	ATYD[HAyneDE]AQHLLDES	21031	1	TRUE
Soluble	E0F67_06385	A0A4U7GDP0	DivIVA domain-containing protein	A0A4U7GDP0_STRPY	TRUE	TRUE	DATDEAK	149.49203	149.49203	2	D[HAyneDE]ATDEAK	9694	1	TRUE
Insoluble	E0F67_06405	A0A5S4TEI8	ATP-dependent Clp protease ATP-binding	A0A5S4TEI8_STRPY	TRUE	TRUE	TIQDYIEDAITDYYLEHPTEK	247.35487	247.35487	3	TIQDYIED[HAyneDE]AITDY	72029	1	TRUE
Insoluble	E0F67_07075	A0A5S4TD33	BMP family ABC transporter substrate-bin	A0A5S4TD33_STRPY	TRUE	TRUE	NVASITFSDHEAAYLAGVAAAK	352.06363	352.06363	3	NVASITFSD[HAyneDE]HEA	47476	1	TRUE
Insoluble	E0F67_07600	A0A5S4TDS8	Peptide ABC transporter substrate-bindin	A0A5S4TDS8_STRPY	TRUE	TRUE	SDALYVVEDSIK	238.50465	238.50465	2	SD[HAyneDE]ALYVVEDSIK	56555	1	TRUE
Insoluble	E0F67_08075	A0A4U9C786	NADP-dependent glyceraldehyde-3-phos	A0A4U9C786_STRPY	TRUE	TRUE	EGNLISPVLFDHVTTDMR	631.7291	631.7291	3	EGNLISPVLFD[HAyneDE]H	51098	1	TRUE
Insoluble	E0F67_08075	A0A4U9C786	NADP-dependent glyceraldehyde-3-phos	A0A4U9C786_STRPY	TRUE	TRUE	EGNLISPVLFDHVTTDMR	555.04504	555.04504	4	EGNLISPVLFDHVTTD[HAyn	51619	1	TRUE
Insoluble	E0F67_08075	A0A4U9C786	NADP-dependent glyceraldehyde-3-phos	A0A4U9C786_STRPY	TRUE	TRUE	EGNLISPVLFDHVTTDMR	553.113	553.113	4	EGNLISPVLFD[HAyneDE]H	51102	1	TRUE
Insoluble	E0F67_08075	A0A4U9C786	NADP-dependent glyceraldehyde-3-phos	A0A4U9C786_STRPY	TRUE	TRUE	EGNLISPVLFDHVTTDMR	550.99713	550.99713	4	EGNLISPVLFD[HAyneDE]H	51115	1	TRUE
Insoluble	E0F67_08075	A0A4U9C786	NADP-dependent glyceraldehyde-3-phos	A0A4U9C786_STRPY	TRUE	TRUE	VADQLAAEIK	689.925	689.925	3	VAD[HAyneDE]QLAAEIK	36469	1	TRUE
Insoluble	E0F67_08075	A0A4U9C786	NADP-dependent glyceraldehyde-3-phos	A0A4U9C786_STRPY	TRUE	TRUE	VADQLAAEIK	464.62625	464.62625	2	VAD[HAyneDE]QLAAEIK	36371	1	TRUE
Insoluble	E0F67_08075	A0A4U9C786	NADP-dependent glyceraldehyde-3-phos	A0A4U9C786_STRPY	TRUE	TRUE	DSAIVLEDADLALAAK	566.6081	566.6081	3	DSAIVLED[HAyneDE]ADLA	62051	2	TRUE
Insoluble	E0F67_08075	A0A4U9C786	NADP-dependent glyceraldehyde-3-phos	A0A4U9C786_STRPY	TRUE	TRUE	DSAIVLEDADLALAAK	180.53874	180.53874	2	D[HAyneDE]SAIVLEDADLA	59947	1	TRUE
Insoluble	E0F67_08075	A0A4U9C786	NADP-dependent glyceraldehyde-3-phos	A0A4U9C786_STRPY	TRUE	TRUE	GTDNFPFLGAK	565.16095	565.16095	3	GTD[HAyneDE]NFPFLGAK	52746	1	TRUE
Insoluble	E0F67_08075	A0A4U9C786	NADP-dependent glyceraldehyde-3-phos	A0A4U9C786_STRPY	TRUE	TRUE	GTDNFPFLGAK	565.12994	565.12994	3	GTD[HAyneDE]NFPFLGAK	52485	1	TRUE
Insoluble	E0F67_08075	A0A4U9C786	NADP-dependent glyceraldehyde-3-phos	A0A4U9C786_STRPY	TRUE	TRUE	GTDNFPFLGAK	565.1295	565.1295	3	GTD[HAyneDE]NFPFLGAK	52735	1	TRUE
Insoluble	E0F67_08075	A0A4U9C786	NADP-dependent glyceraldehyde-3-phos	A0A4U9C786_STRPY	TRUE	TRUE	GTDNFPFLGAK	255.86392	255.86392	2	GTD[HAyneDE]NFPFLGAK	52574	1	TRUE
Insoluble	E0F67_08760	A0A5S4TET5	ATP-binding cassette domain-containing	A0A5S4TET5_STRPY	TRUE	TRUE	ADFSEEDGVR	184.81505	184.81505	2	AD[HAyneDE]FSEEDGVR	23868	1	TRUE
Insoluble	E0F67_08760	A0A5S4TET5	ATP-binding cassette domain-containing	A0A5S4TET5_STRPY	TRUE	TRUE	ADFSEEDGVR	189.14563	189.14563	2	AD[HAyneDE]FSEEDGVR	23887	1	TRUE
Insoluble	efp	A0A4U7GCX0	Elongation factor P	A0A4U7GCX0_STRPY	TRUE	TRUE	TGSTFDTTYRPDEK	390.62482	390.62482	3	TGSTFD[HAyneDE]TTYRPDI	19166	1	TRUE
Insoluble	efp	A0A4U7GCX0	Elongation factor P	A0A4U7GCX0_STRPY	TRUE	TRUE	TGSTFDTTYRPDEK	375.97598	375.97598	3	TGSTFD[HAyneDE]TTYRPDI	20044	1	TRUE
Insoluble	efp	A0A4U7GCX0	Elongation factor P	A0A4U7GCX0_STRPY	TRUE	TRUE	TGSTFDTTYRPDEK	400.23834	400.23834	3	TGSTFD[HAyneDE]TTYRPDI	19988	1	TRUE
Insoluble	efp	A0A4U7GCX0	Elongation factor P	A0A4U7GCX0_STRPY	TRUE	TRUE	TGSTFDTTYRPDEK	176.6922	176.6922	4	TGSTFD[HAyneDE]TTYRPDI	19154	1	TRUE
Insoluble	efp	A0A4U7GCX0	Elongation factor P	A0A4U7GCX0_STRPY	TRUE	TRUE	TGSTFDTTYRPDEK	171.63835	171.63835	4	TGSTFD[HAyneDE]TTYRPDI	19188	1	TRUE
Insoluble	efp	A0A4U7GCX0	Elongation factor P	A0A4U7GCX0_STRPY	TRUE	TRUE	TGSTFDTTYRPDEK	171.63835	171.63835	4	TGSTFD[HAyneDE]TTYRPDI	19197	1	TRUE
Insoluble	emm	A0A5S4TNZ2	YSIRK-type signal peptide-containing pro	A0A5S4TNZ2_STRPY	TRUE	TRUE	QISDASR	561.5756	561.5756	3	QISD[HAyneDE]ASR	13673	1	TRUE
Insoluble	eno	A3F8V6	Enolase	A3F8V6_STRPY	TRUE	TRUE	SIITDVYAR	2799.2512	2799.2512	2	SIITD[HAyneDE]VYAR	38735	1	TRUE
Insoluble	eno	A3F8V6	Enolase	A3F8V6_STRPY	TRUE	TRUE	SIITDVYAR	157.3304	157.3304	2	SIITD[HAyneDE]VYAR	37209	1	TRUE
Insoluble	eno	A3F8V6	Enolase	A3F8V6_STRPY	TRUE	TRUE	SIITDVYAR	2799.2512	2799.2512	2	SIITD[HAyneDE]VYAR	38527	1	TRUE
Insoluble	eno	A3F8V6	Enolase	A3F8V6_STRPY	TRUE	TRUE	SIITDVYAR	2799.2512	2799.2512	2	SIITD[HAyneDE]VYAR	38626	1	FALSE
Insoluble	eno	A3F8V6	Enolase	A3F8V6_STRPY	TRUE	TRUE	SIITDVYAR	2412.0693	2412.0693	3	SIITD[HAyneDE]VYAR	38558	1	TRUE
Insoluble	eno	A3F8V6	Enolase	A3F8V6_STRPY	TRUE	TRUE	SIITDVYAR	2412.1917	2412.1917	3	SIITD[HAyneDE]VYAR	38675	1	TRUE
Insoluble	eno	A3F8V6	Enolase	A3F8V6_STRPY	TRUE	TRUE	SIITDVYAR	2412.1995	2412.1995	3	SIITD[HAyneDE]VYAR	38629	1	TRUE
Insoluble	eno	A3F8V6	Enolase	A3F8V6_STRPY	TRUE	TRUE	SIITDVYAR	2412.0693	2412.0693	3	SIITD[HAyneDE]VYAR	38578	1	FALSE
Insoluble	eno	A3F8V6	Enolase	A3F8V6_STRPY	TRUE	TRUE	RVQLVGDDFFVTNTEYLAR	784.3456	784.3456	3	RVQLVGDD[HAyneDE]FFV	56129	1	TRUE
Insoluble	eno	A3F8V6	Enolase	A3F8V6_STRPY	TRUE	TRUE	RVQLVGDDFFVTNTEYLAR	926.3607	926.3607	3	RVQLVGDD[HAyneDE]FFV	56120	1	TRUE
Insoluble	eno	A3F8V6	Enolase	A3F8V6_STRPY	TRUE	TRUE	TSAEQVDYLEELVNK	1423.892	1423.892	3	TSAEQVD[HAyneDE]YLEEL	62090	1	TRUE
Insoluble	eno	A3F8V6	Enolase	A3F8V6_STRPY	TRUE	TRUE	TSAEQVDYLEELVNK	1423.892	1423.892	3	TSAEQVD[HAyneDE]YLEEL	62128	1	TRUE
Insoluble	eno	A3F8V6	Enolase	A3F8V6_STRPY	TRUE	TRUE	TSAEQVDYLEELVNK	816.31244	816.31244	2	TSAEQVD[HAyneDE]YLEEL	62198	2	TRUE
Insoluble	eno	A3F8V6	Enolase	A3F8V6_STRPY	TRUE	TRUE	IEDQLGEVAQYK	1538.6155	1538.6155	3	IED[HAyneDE]QLGEVAQYK	38170	1	TRUE
Insoluble	eno	A3F8V6	Enolase	A3F8V6_STRPY	TRUE	TRUE	IEDQLGEVAQYK	1538.6002	1538.6002	3	IED[HAyneDE]QLGEVAQYK	38214	1	TRUE
Insoluble	eno	A3F8V6	Enolase	A3F8V6_STRPY	TRUE	TRUE	IEDQLGEVAQYK	1488.4924	1488.4924	2	IED[HAyneDE]QLGEVAQYK	38202	1	TRUE
Insoluble	eno	A3F8V6	Enolase	A3F8V6_STRPY	TRUE	TRUE	GLVTAVGDEGGFAPK	835.9198	835.9198	3	GLVTAVGD[HAyneDE]EGGI	40335	1	TRUE
Insoluble	eno	A3F8V6	Enolase	A3F8V6_STRPY	TRUE	TRUE	GLVTAVGDEGGFAPK	290.4907	290.4907	2	GLVTAVGD[HAyneDE]EGGI	40058	1	TRUE
Insoluble	eno	A3F8V6	Enolase	A3F8V6_STRPY	TRUE	TRUE	GLVTAVGDEGGFAPK	290.49573	290.49573	2	GLVTAVGD[HAyneDE]EGGI	40064	1	TRUE
Insoluble	eno	A3F8V6	Enolase	A3F8V6_STRPY	TRUE	TRUE	SGETEDSTIADIAVATNAGQIK	302.97708	302.97708	3	SGETED[HAyneDE]STIADIA	52548	1	TRUE
Insoluble	eno	A3F8V6	Enolase	A3F8V6_STRPY	TRUE	TRUE	SGETEDSTIADIAVATNAGQIK	563.05786	563.05786	3	SGETED[HAyneDE]STIADIA	50706	2	TRUE
Insoluble	eno	A3F8V6	Enolase	A3F8V6_STRPY	TRUE	TRUE	SGETEDSTIADIAVATNAGQIK	455.3586	455.3586	3	SGETED[HAyneDE]STIADIA	52524	1	TRUE
Insoluble	eno	A3F8V6	Enolase	A3F8V6_STRPY	TRUE	TRUE	SGETEDSTIADIAVATNAGQIK	246.17307	246.17307	3	SGETEDSTIAD[HAyneDE]IA	48315	1	TRUE
Insoluble	eno	A3F8V6	Enolase	A3F8V6_STRPY	TRUE	TRUE	SGETEDSTIADIAVATNAGQIK	159.51587	159.51587	3	SGETEDSTIAD[HAyneDE]IA	48381	1	TRUE
Insoluble	eno	A3F8V6	Enolase	A3F8V6_STRPY	TRUE	TRUE	SGETEDSTIADIAVATNAGQIK	246.17307	246.17307	3	SGETEDSTIAD[HAyneDE]IA	48357	1	TRUE
Insoluble	eno	A3F8V6	Enolase	A3F8V6_STRPY	TRUE	TRUE	SGETEDSTIADIAVATNAGQIK	159.51587	159.51587	3	SGETEDSTIAD[HAyneDE]IA	48370	1	FALSE
Insoluble	eno	A3F8V6	Enolase	A3F8V6_STRPY	TRUE	TRUE	DQQAIDR	1258.9974	1258.9974	2	D[HAyneDE]QQAIDR	13972	1	TRUE
Insoluble	eno	A3F8V6	Enolase	A3F8V6_STRPY	TRUE	TRUE	DQQAIDR	1262.4241	1262.4241	2	D[HAyneDE]QQAIDR	14034	1	TRUE
Insoluble	eno	A3F8V6	Enolase	A3F8V6_STRPY	TRUE	TRUE	DQQAIDR	864.8463	864.8463	3	D[HAyneDE]QQAIDR	14036	1	TRUE
Insoluble	eno	A3F8V6	Enolase	A3F8V6_STRPY	TRUE	TRUE	DQQAIDR	864.81055	864.81055	3	D[HAyneDE]QQAIDR	13996	1	TRUE
Insoluble	eno	A3F8V6	Enolase	A3F8V6_STRPY	TRUE	TRUE	DQQAIDR	864.8455	864.8455	3	D[HAyneDE]QQAIDR	14097	1	TRUE
Insoluble	eno	A3F8V6	Enolase	A3F8V6_STRPY	TRUE	TRUE	DQQAIDR	430.7036	430.7036	3	DQQAID[HAyneDE]R	17817	1	TRUE
Insoluble	eno	A3F8V6	Enolase	A3F8V6_STRPY	TRUE	TRUE	DQQAIDR	314.77457	314.77457	2	DQQAID[HAyneDE]R	17808	1	TRUE
Insoluble	eno	A3F8V6	Enolase	A3F8V6_STRPY	TRUE	TRUE	VQLVGDDFFVTNTEYLAR	1191.2594	1191.2594	3	VQLVGDD[HAyneDE]FFVTN	67204	1	TRUE
Insoluble	eno	A3F8V6	Enolase	A3F8V6_STRPY	TRUE	TRUE	AVDNVNNIIAEAIIGYDVR	685.1463	685.1463	3	AVD[HAyneDE]NVNNIIAEA	76434	1	TRUE
Insoluble	eno	A3F8V6	Enolase	A3F8V6_STRPY	TRUE	TRUE	AVDNVNNIIAEAIIGYDVR	598.38763	598.38763	2	AVD[HAyneDE]NVNNIIAEA	76398	1	TRUE
Insoluble	eno	A3F8V6	Enolase	A3F8V6_STRPY	TRUE	TRUE	AIEAAGYEAGENGIMIGFDCASSEFYDK	699.0815	699.0815	3	AIEAAGYEAGENGIM[Oxida	70977	2	TRUE
Insoluble	eno	A3F8V6	Enolase	A3F8V6_STRPY	TRUE	TRUE	AIEAAGYEAGENGIMIGFDCASSEFYDK	699.2244	699.2244	3	AIEAAGYEAGENGIM[Oxida	71393	1	TRUE
Insoluble	eno	A3F8V6	Enolase	A3F8V6_STRPY	TRUE	TRUE	AIEAAGYEAGENGIMIGFDCASSEFYDK	2895.4536	2895.4536	3	AIEAAGYEAGENGIM[Oxida	69519	1	TRUE
Soluble	eno	A3F8V6	Enolase	A3F8V6_STRPY	TRUE	TRUE	DQQAIDR	3732.1409	3732.1409	2	D[HAyneDE]QQAIDR	11976	1	TRUE
Soluble	eno	A3F8V6	Enolase	A3F8V6_STRPY	TRUE	TRUE	DQQAIDR	3743.5002	3743.5002	2	D[HAyneDE]QQAIDR	12647	1	TRUE
Soluble	eno	A3F8V6	Enolase	A3F8V6_STRPY	TRUE	TRUE	DQQAIDR	3732.1519	3732.1519	2	D[HAyneDE]QQAIDR	11998	1	TRUE
Soluble	eno	A3F8V6	Enolase	A3F8V6_STRPY	TRUE	TRUE	DQQAIDR	3732.1233	3732.1233	2	D[HAyneDE]QQAIDR	12059	1	FALSE
Soluble	eno	A3F8V6	Enolase	A3F8V6_STRPY	TRUE	TRUE	DQQAIDR	2210.866	2210.866	3	D[HAyneDE]QQAIDR	12145	1	TRUE
Soluble	eno	A3F8V6	Enolase	A3F8V6_STRPY	TRUE	TRUE	DQQAIDR	2211.2864	2211.2864	3	D[HAyneDE]QQAIDR	11994	1	TRUE
Soluble	eno	A3F8V6	Enolase	A3F8V6_STRPY	TRUE	TRUE	DQQAIDR	2215.516	2215.516	3	D[HAyneDE]QQAIDR	12067	1	TRUE
Soluble	eno	A3F8V6	Enolase	A3F8V6_STRPY	TRUE	TRUE	DQQAIDR	2215.516	2215.516	3	D[HAyneDE]QQAIDR	12061	1	FALSE
Soluble	eno	A3F8V6	Enolase	A3F8V6_STRPY	TRUE	TRUE	DQQAIDR	2211.2864	2211.2864	3	D[HAyneDE]QQAIDR	12004	1	FALSE
Soluble	eno	A3F8V6	Enolase	A3F8V6_STRPY	TRUE	TRUE	DQQAIDR	275.68835	275.68835	3	D[HAyneDE]QQAIDR	17263	1	FALSE
Soluble	eno	A3F8V6	Enolase	A3F8V6_STRPY	TRUE	TRUE	DQQAIDR	2211.3123	2211.3123	3	D[HAyneDE]QQAIDR	12595	2	FALSE
Soluble	eno	A3F8V6	Enolase	A3F8V6_STRPY	TRUE	TRUE	TSAEQVDYLEELVNK	539.11	539.11	3	TSAEQVD[HAyneDE]YLEEL	58072	1	TRUE
Soluble	eno	A3F8V6	Enolase	A3F8V6_STRPY	TRUE	TRUE	TSAEQVDYLEELVNK	539.09424	539.09424	3	TSAEQVD[HAyneDE]YLEEL	58010	1	TRUE
Soluble	eno	A3F8V6	Enolase	A3F8V6_STRPY	TRUE	TRUE	TSAEQVDYLEELVNK	209.15817	209.15817	2	TSAEQVD[HAyneDE]YLEEL	58131	1	TRUE
Soluble	eno	A3F8V6	Enolase	A3F8V6_STRPY	TRUE	TRUE	GLVTAVGDEGGFAPK	222.36197	222.36197	3	GLVTAVGD[HAyneDE]EGGI	37922	1	TRUE
Soluble	eno	A3F8V6	Enolase	A3F8V6_STRPY	TRUE	TRUE	IEDQLGEVAQYK	2692.4126	2692.4126	3	IED[HAyneDE]QLGEVAQYK	34034	1	TRUE
Soluble	eno	A3F8V6	Enolase	A3F8V6_STRPY	TRUE	TRUE	IEDQLGEVAQYK	2692.4124	2692.4124	3	IED[HAyneDE]QLGEVAQYK	34119	1	TRUE
Soluble	eno	A3F8V6	Enolase	A3F8V6_STRPY	TRUE	TRUE	IEDQLGEVAQYK	2583.12	2583.12	2	IED[HAyneDE]QLGEVAQYK	33985	1	TRUE
Soluble	eno	A3F8V6	Enolase	A3F8V6_STRPY	TRUE	TRUE	IEDQLGEVAQYK	2587.7668	2587.7668	2	IED[HAyneDE]QLGEVAQYK	34003	1	TRUE
Soluble	eno	A3F8V6	Enolase	A3F8V6_STRPY	TRUE	TRUE	IEDQLGEVAQYK	2587.76	2587.76	2	IED[HAyneDE]QLGEVAQYK	34087	1	TRUE
Soluble	eno	A3F8V6	Enolase	A3F8V6_STRPY	TRUE	TRUE	IEDQLGEVAQYK	2587.7468	2587.7468	2	IED[HAyneDE]QLGEVAQYK	34066	1	FALSE
Soluble	eno	A3F8V6	Enolase	A3F8V6_STRPY	TRUE	TRUE	VLPTPMMNIINGGSHSDAPIAFQEFMIMP	509.67633	509.67633	4	VLPTPMMNIINGGSHSD[HA	63481	1	TRUE
Soluble	eno	A3F8V6	Enolase	A3F8V6_STRPY	TRUE	TRUE	VLPTPMMNIINGGSHSDAPIAFQEFMIMP	317.13898	317.13898	4	VLPTPM[Oxidation (M)]M[C	58404	1	TRUE
Soluble	eno	A3F8V6	Enolase	A3F8V6_STRPY	TRUE	TRUE	SIITDVYAR	402.79575	402.79575	3	SIITD[HAyneDE]VYAR	32900	1	TRUE
Soluble	eno	A3F8V6	Enolase	A3F8V6_STRPY	TRUE	TRUE	SIITDVYAR	5812.499	5812.499	3	SIITD[HAyneDE]VYAR	34460	1	TRUE
Soluble	eno	A3F8V6	Enolase	A3F8V6_STRPY	TRUE	TRUE	SIITDVYAR	5812.501	5812.501	3	SIITD[HAyneDE]VYAR	34436	1	TRUE
Soluble	eno	A3F8V6	Enolase	A3F8V6_STRPY	TRUE	TRUE	SIITDVYAR	5812.5005	5812.5005	3	SIITD[HAyneDE]VYAR	34383	1	FALSE
Soluble	eno	A3F8V6	Enolase	A3F8V6_STRPY	TRUE	TRUE	SIITDVYAR	5812.499	5812.499	3	SIITD[HAyneDE]VYAR	34279	1	FALSE
Soluble	eno	A3F8V6	Enolase	A3F8V6_STRPY	TRUE	TRUE	SIITDVYAR	5812.5005	5812.5005	3	SIITD[HAyneDE]VYAR	34378	1	FALSE
Soluble	eno	A3F8V6	Enolase	A3F8V6_STRPY	TRUE	TRUE	SIITDVYAR	304.4378	304.4378	2	SIITD[HAyneDE]VYAR	32896	1	TRUE
Soluble	eno	A3F8V6	Enolase	A3F8V6_STRPY	TRUE	TRUE	SIITDVYAR	304.4378	304.4378	2	SIITD[HAyneDE]VYAR	32887	1	TRUE
Soluble	eno	A3F8V6	Enolase	A3F8V6_STRPY	TRUE	TRUE	SIITDVYAR	6412.047	6412.047	2	SIITD[HAyneDE]VYAR	34664	1	TRUE
Soluble	eno	A3F8V6	Enolase	A3F8V6_STRPY	TRUE	TRUE	SIITDVYAR	6412.0464	6412.0464	2	SIITD[HAyneDE]VYAR	34276	1	FALSE
Soluble	eno	A3F8V6	Enolase	A3F8V6_STRPY	TRUE	TRUE	SIITDVYAR	6412.047	6412.047	2	SIITD[HAyneDE]VYAR	34375	1	FALSE
Soluble	eno	A3F8V6	Enolase	A3F8V6_STRPY	TRUE	TRUE	SIITDVYAR	1625.7955	1625.7955	2	[Acetyl (Protein N-term)]SI	46664	1	TRUE
Soluble	eno	A3F8V6	Enolase	A3F8V6_STRPY	TRUE	TRUE	SIITDVYAR	74.53307	74.53307	4	SIITD[HAyneDE]VYAR	34473	1	TRUE
Soluble	eno	A3F8V6	Enolase	A3F8V6_STRPY	TRUE	TRUE	AVDNVNNIIAEAIIGYDVR	1576.2864	1576.2864	2	AVD[HAyneDE]NVNNIIAEA	72338	1	TRUE
Soluble	eno	A3F8V6	Enolase	A3F8V6_STRPY	TRUE	TRUE	AVDNVNNIIAEAIIGYDVR	1068.5499	1068.5499	3	AVD[HAyneDE]NVNNIIAEA	72075	2	TRUE
Soluble	eno	A3F8V6	Enolase	A3F8V6_STRPY	TRUE	TRUE	AVDNVNNIIAEAIIGYDVR	1068.5499	1068.5499	3	AVD[HAyneDE]NVNNIIAEA	72317	1	TRUE
Soluble	eno	A3F8V6	Enolase	A3F8V6_STRPY	TRUE	TRUE	AVDNVNNIIAEAIIGYDVR	1187.5045	1187.5045	4	AVD[HAyneDE]NVNNIIAEA	72333	1	TRUE
Soluble	eno	A3F8V6	Enolase	A3F8V6_STRPY	TRUE	TRUE	AVDNVNNIIAEAIIGYDVRDQQAIDR	320.1407	320.1407	4	AVDNVNNIIAEAIIGYDVRD(	69261	1	TRUE
Insoluble	fabF	A0A4Q1QZM0	3-oxoacyl-[acyl-carrier-protein] synthase	A0A4Q1QZM0_STRPY	TRUE	TRUE	FGTQDVMIVGGSEAAITK	784.3039	784.3039	3	FGTQD[HAyneDE]VM[Oxid	39862	2	TRUE
Insoluble	fabF	A0A4Q1QZM0	3-oxoacyl-[acyl-carrier-protein] synthase	A0A4Q1QZM0_STRPY	TRUE	TRUE	FGTQDVMIVGGSEAAITK	575.60187	575.60187	2	FGTQD[HAyneDE]VM[Oxid	39878	1	TRUE
Insoluble	fabF	A0A4Q1QZM0	3-oxoacyl-[acyl-carrier-protein] synthase	A0A4Q1QZM0_STRPY	TRUE	TRUE	FGTQDVMIVGGSEAAITK	575.6179	575.6179	2	FGTQD[HAyneDE]VM[Oxid	39916	1	TRUE
Insoluble	fabF	A0A4Q1QZM0	3-oxoacyl-[acyl-carrier-protein] synthase	A0A4Q1QZM0_STRPY	TRUE	TRUE	FAIAGFQSLTALSTTEDPSR	800.5194	800.5194	3	FAIAGFQSLTALSTTED[HAy	68428	1	TRUE
Insoluble	fabF	A0A4Q1QZM0	3-oxoacyl-[acyl-carrier-protein] synthase	A0A4Q1QZM0_STRPY	TRUE	TRUE	SINTACASSNDAIGDAFR	602.1453	602.1453	3	SINTAC[Carbamidomethyl	48839	1	TRUE
Insoluble	fabF	A0A4Q1QZM0	3-oxoacyl-[acyl-carrier-protein] synthase	A0A4Q1QZM0_STRPY	TRUE	TRUE	FDTTDYAVK	411.11237	411.11237	2	FD[HAyneDE]TTDYAVK	28623	2	TRUE
Insoluble	fabF	A0A4Q1QZM0	3-oxoacyl-[acyl-carrier-protein] synthase	A0A4Q1QZM0_STRPY	TRUE	TRUE	FDTTDYAVK	544.8621	544.8621	2	FD[HAyneDE]TTDYAVK	26195	1	TRUE
Insoluble	fabF	A0A4Q1QZM0	3-oxoacyl-[acyl-carrier-protein] synthase	A0A4Q1QZM0_STRPY	TRUE	TRUE	FDTTDYAVK	392.66632	392.66632	2	FDTTD[HAyneDE]YAVK	28639	1	TRUE
Insoluble	fabG	A0A5S4TDJ8	3-oxoacyl-[acyl-carrier-protein] reductase	A0A5S4TDJ8_STRPY	TRUE	TRUE	NICVNAIAPGFIESDMTGVLPEK	499.36102	499.36102	3	NIC[Carbamidomethyl (C)	59579	1	TRUE
Insoluble	fabG	A0A5S4TDJ8	3-oxoacyl-[acyl-carrier-protein] reductase	A0A5S4TDJ8_STRPY	TRUE	TRUE	NICVNAIAPGFIESDMTGVLPEK	340.28955	340.28955	3	NIC[Carbamidomethyl (C)	61736	2	TRUE
Insoluble	fabG	A0A5S4TDJ8	3-oxoacyl-[acyl-carrier-protein] reductase	A0A5S4TDJ8_STRPY	TRUE	TRUE	NICVNAIAPGFIESDMTGVLPEK	375.43707	375.43707	4	NIC[Carbamidomethyl (C)	59791	2	TRUE
Insoluble	fabG	A0A5S4TDJ8	3-oxoacyl-[acyl-carrier-protein] reductase	A0A5S4TDJ8_STRPY	TRUE	TRUE	NICVNAIAPGFIESDMTGVLPEK	375.43707	375.43707	4	NIC[Carbamidomethyl (C)	59801	2	TRUE
Insoluble	fabG	A0A5S4TDJ8	3-oxoacyl-[acyl-carrier-protein] reductase	A0A5S4TDJ8_STRPY	TRUE	TRUE	MTEEDFER	274.07343	274.07343	3	M[Oxidation (M)]TEED[HA	19753	1	TRUE
Insoluble	fabG	A0A5S4TDJ8	3-oxoacyl-[acyl-carrier-protein] reductase	A0A5S4TDJ8_STRPY	TRUE	TRUE	MTEEDFER	274.18613	274.18613	3	M[Oxidation (M)]TEED[HA	19744	1	TRUE
Insoluble	fabK	A0A4U9C1P1	Enoyl-[acyl-carrier-protein] reductase Fal	A0A4U9C1P1_STRPY	TRUE	TRUE	HAVIEGDVVNGSVMAGQIAGLVR	601.8246	601.8246	4	HAVIEGD[HAyneDE]VVNGS	46743	1	TRUE
Insoluble	fabK	A0A4U9C1P1	Enoyl-[acyl-carrier-protein] reductase Fal	A0A4U9C1P1_STRPY	TRUE	TRUE	TATDIEEMGAGSLR	340.3271	340.3271	3	TATD[HAyneDE]IEEM[Oxid	25315	1	TRUE

Insoluble	*fabK*	A0A4U9C1P1	Enoyl-[acyl-carrier-protein] reductase Fa	A0A4U9C1P1_STRPY	TRUE	TRUE	TATDIEEMGAGSLR	340.3439	340.3439	3	TATD[HAyneDE]IEEM[Oxida	25363	1	TRUE
Insoluble	*fabK*	A0A4U9C1P1	Enoyl-[acyl-carrier-protein] reductase Fa	A0A4U9C1P1_STRPY	TRUE	TRUE	DIDTVISAQVVGHPVR	288.31436	288.31436	3	D[HAyneDE]IDTVISAQVVG	47886	1	TRUE
Insoluble	*fabK*	A0A4U9C1P1	Enoyl-[acyl-carrier-protein] reductase Fa	A0A4U9C1P1_STRPY	TRUE	TRUE	DIDTVISAQVVGHPVR	151.34317	151.34317	4	D[HAyneDE]IDTVISAQVVG	47998	3	TRUE
Insoluble	*fasA*	A0A4U7G2Y1;WP_002986100.1	Response regulator transcription factor;L	A0A4U7G2Y1_STRPY;WP_0029	TRUE	FALSE	GDHQLYFLDIEIGEYTR	361.30334	361.30334	3	GDHQLYFLD[HAyneDE]IEI	62891	1	TRUE
Insoluble	*fasA*	A0A4U7G2Y1;WP_002986100.1	Response regulator transcription factor;L	A0A4U7G2Y1_STRPY;WP_0029	TRUE	FALSE	GDHQLYFLDIEIGEYTR	353.1165	353.1165	4	GDHQLYFLD[HAyneDE]IEI	62810	1	TRUE
Insoluble	*fba*	A0A4U9TD41	Fructose-bisphosphate aldolase	A0A4U9TD41_STRPY	TRUE	TRUE	IDVFGSANKA	240.14557	240.14557	3	ID[HAyneDE]VFGSANKA	36509	3	TRUE
Insoluble	*fba*	A0A4U9TD41	Fructose-bisphosphate aldolase	A0A4U9TD41_STRPY	TRUE	TRUE	GVSVEAEVGTIGGEEDGIIGK	479.03778	479.03778	3	GVSVEAEVGTIGGEED[HAy	49573	1	TRUE
Insoluble	*fba*	A0A4U9TD41	Fructose-bisphosphate aldolase	A0A4U9TD41_STRPY	TRUE	TRUE	GVSVEAEVGTIGGEEDGIIGK	484.14203	484.14203	3	GVSVEAEVGTIGGEED[HAy	49582	1	TRUE
Insoluble	*fba*	A0A4U9TD41	Fructose-bisphosphate aldolase	A0A4U9TD41_STRPY	TRUE	TRUE	GELAPIEDAK	699.9362	699.9362	2	GELAPIED[HAyneDE]AK	31574	2	TRUE
Insoluble	*fba*	A0A4U9TD41	Fructose-bisphosphate aldolase	A0A4U9TD41_STRPY	TRUE	TRUE	NYEANEAEYDGK	421.54974	421.54974	3	NYEANEAEYD[HAyneDE]G	18620	2	TRUE
Soluble	*fba*	A0A4U9TD41	Fructose-bisphosphate aldolase	A0A4U9TD41_STRPY	TRUE	TRUE	AMVETGIDFLAAGIGNIHGPYPENWEGLA	1043.1869	1043.1869	4	AM[Oxidation (M)]VETGIDF	68974	2	TRUE
Soluble	*fba*	A0A4U9TD41	Fructose-bisphosphate aldolase	A0A4U9TD41_STRPY	TRUE	TRUE	AMVETGIDFLAAGIGNIHGPYPENWEGLA	359.60153	359.60153	5	AM[Oxidation (M)]VETGIDF	68786	1	TRUE
Soluble	*fba*	A0A4U9TD41	Fructose-bisphosphate aldolase	A0A4U9TD41_STRPY	TRUE	TRUE	GELAPIEDAK	229.76805	229.76805	2	GELAPIED[HAyneDE]AK	26477	1	TRUE
Soluble	*fba*	A0A4U9TD41	Fructose-bisphosphate aldolase	A0A4U9TD41_STRPY	TRUE	TRUE	GELAPIEDAK	229.03041	229.03041	2	GELAPIED[HAyneDE]AK	26498	1	TRUE
Soluble	*fba*	A0A4U9TD41	Fructose-bisphosphate aldolase	A0A4U9TD41_STRPY	TRUE	TRUE	GELAPIEDAK	630.81555	630.81555	3	GELAPIED[HAyneDE]AK	26452	1	TRUE
Soluble	*fba*	A0A4U9TD41	Fructose-bisphosphate aldolase	A0A4U9TD41_STRPY	TRUE	TRUE	GELAPIEDAK	678.4218	678.4218	3	GELAPIED[HAyneDE]AK	26465	1	TRUE
Soluble	*fba*	A0A4U9TD41	Fructose-bisphosphate aldolase	A0A4U9TD41_STRPY	TRUE	TRUE	GVSVEAEVGTIGGEEDGIIGK	179.74966	179.74966	2	GVSVEAEVGTIGGEED[HAy	45615	1	TRUE
Insoluble	*ffh*	A0A5S4THN2	Signal recognition particle protein	A0A5S4THN2_STRPY	TRUE	TRUE	LALLEADVALPVVK	435.30328	435.30328	2	LALLEAD[HAyneDE]VALPV	66075	1	TRUE
Insoluble	*ffh*	A0A5S4THN2	Signal recognition particle protein	A0A5S4THN2_STRPY	TRUE	TRUE	ITDIETFHPDR	237.3364	237.3364	3	ITD[HAyneDE]IETFHPDR	27365	1	TRUE
Soluble	*frr*	A0A5S4TH24	Ribosome-recycling factor	A0A5S4TH24_STRPY	TRUE	TRUE	VLLISPFDK	77.49765	77.49765	3	VLLISPFD[HAyneDE]K	54618	1	TRUE
Soluble	*frr*	A0A5S4TH24	Ribosome-recycling factor	A0A5S4TH24_STRPY	TRUE	TRUE	ALNASDLGITPANDGSVIR	410.81494	410.81494	3	ALNASD[HAyneDE]LGITPA	40826	1	TRUE
Soluble	*frr*	A0A5S4TH24	Ribosome-recycling factor	A0A5S4TH24_STRPY	TRUE	TRUE	ALNASDLGITPANDGSVIR	410.81494	410.81494	3	ALNASD[HAyneDE]LGITPA	40800	1	TRUE
Soluble	*frr*	A0A5S4TH24	Ribosome-recycling factor	A0A5S4TH24_STRPY	TRUE	TRUE	ALNASDLGITPANDGSVIR	146.32826	146.32826	2	ALNASD[HAyneDE]LGITPA	40846	1	TRUE
Insoluble	*ftsA*	A0A4U7GQI4	Cell division protein FtsA	A0A4U7GQI4_STRPY	FALSE	FALSE	EVISLVPEEFIVDGFQGIRDPR	222.73788	222.73788	3	EVISLVPEEFIVD[HAyneDE]	73790	1	TRUE
Insoluble	*ftsA*	A0A4U7GQI4	Cell division protein FtsA	A0A4U7GQI4_STRPY	FALSE	FALSE	EVISLVPEEFIVDGFQGIRDPR	222.73672	222.73672	3	EVISLVPEEFIVDGFQGIRD[	73797	1	TRUE
Insoluble	*ftsH*	A0A4U9T4V6	ATP-dependent zinc metalloprotease Fts	A0A4U9T4V6_STRPY	TRUE	TRUE	MPVDLETDDNEAHALSYDEIK	391.66992	391.66992	4	M[Oxidation (M)]PVDLETD	36373	1	TRUE
Insoluble	*ftsH*	A0A4U9T4V6	ATP-dependent zinc metalloprotease Fts	A0A4U9T4V6_STRPY	TRUE	TRUE	AYSAQTAQMIDDEVR	532.4738	532.4738	3	AYSAQTAQM[Oxidation (M	27978	1	TRUE
Insoluble	*ftsH*	A0A4U9T4V6	ATP-dependent zinc metalloprotease Fts	A0A4U9T4V6_STRPY	TRUE	TRUE	AIIFIDEIDAVGR	295.1764	295.1764	3	AIIFID[HAyneDE]EIDAVGR	65044	2	TRUE
Insoluble	*ftsH*	A0A4U9T4V6	ATP-dependent zinc metalloprotease Fts	A0A4U9T4V6_STRPY	TRUE	TRUE	AIIFIDEIDAVGR	295.1764	295.1764	3	AIIFID[HAyneDE]EIDAVGR	65032	2	TRUE
Insoluble	*ftsH*	A0A4U9T4V6	ATP-dependent zinc metalloprotease Fts	A0A4U9T4V6_STRPY	TRUE	TRUE	AIIFIDEIDAVGR	245.39258	245.39258	2	AIIFID[HAyneDE]EIDAVGR	65106	1	TRUE
Insoluble	*ftsZ*	A0A5S4TMN0	Cell division protein FtsZ	A0A5S4TMN0_STRPY	TRUE	TRUE	AFSFDTASIQGAIIK	258.25226	258.25226	3	[Acetyl (Protein N-term)]AF	66759	1	TRUE
Insoluble	*ftsZ*	A0A5S4TMN0	Cell division protein FtsZ	A0A5S4TMN0_STRPY	TRUE	TRUE	AFSFDTASIQGAIIK	258.04822	258.04822	3	[Acetyl (Protein N-term)]AF	66819	1	TRUE
Insoluble	*ftsZ*	A0A5S4TMN0	Cell division protein FtsZ	A0A5S4TMN0_STRPY	TRUE	TRUE	AFSFDTASIQGAIIK	258.25226	258.25226	3	[Acetyl (Protein N-term)]AF	66809	1	TRUE
Insoluble	*ftsZ*	A0A5S4TMN0	Cell division protein FtsZ	A0A5S4TMN0_STRPY	TRUE	TRUE	AFSFDTASIQGAIIK	255.08926	255.08926	2	AFSFD[HAyneDE]TASIQGA	56999	1	TRUE
Insoluble	*ftsZ*	A0A5S4TMN0	Cell division protein FtsZ	A0A5S4TMN0_STRPY	TRUE	TRUE	AFSFDTASIQGAIIK	255.11188	255.11188	2	AFSFD[HAyneDE]TASIQGA	57012	1	TRUE
Soluble	*ftsZ*	A0A5S4TMN0	Cell division protein FtsZ	A0A5S4TMN0_STRPY	TRUE	TRUE	AFSFDTASIQGAIIK	252.69983	252.69983	3	[Acetyl (Protein N-term)]AF	62583	1	TRUE
Insoluble	*fusA*	A0A4U9BZP4	Elongation factor G	A0A4U9BZP4_STRPY	TRUE	TRUE	LYDGSYHDVDSSETAFK	763.40955	763.40955	3	LYD[HAyneDE]GSYHDVDS	25857	1	TRUE
Insoluble	*fusA*	A0A4U9BZP4	Elongation factor G	A0A4U9BZP4_STRPY	TRUE	TRUE	LYDGSYHDVDSSETAFK	745.741	745.741	3	LYD[HAyneDE]GSYHDVDS	25784	1	TRUE
Insoluble	*fusA*	A0A4U9BZP4	Elongation factor G	A0A4U9BZP4_STRPY	TRUE	TRUE	LYDGSYHDVDSSETAFK	756.9158	756.9158	3	LYDGSYHD[HAyneDE]VDS	26365	1	TRUE
Insoluble	*fusA*	A0A4U9BZP4	Elongation factor G	A0A4U9BZP4_STRPY	TRUE	TRUE	LYDGSYHDVDSSETAFK	631.55963	631.55963	3	LYDGSYHDVD[HAyneDE]S	27028	1	TRUE
Insoluble	*fusA*	A0A4U9BZP4	Elongation factor G	A0A4U9BZP4_STRPY	TRUE	TRUE	LYDGSYHDVDSSETAFK	621.28937	621.28937	4	LYDGSYHD[HAyneDE]VDS	26278	1	TRUE
Insoluble	*fusA*	A0A4U9BZP4	Elongation factor G	A0A4U9BZP4_STRPY	TRUE	TRUE	LYDGSYHDVDSSETAFK	618.67737	618.67737	4	LYD[HAyneDE]GSYHDVDS	25785	1	TRUE
Insoluble	*fusA*	A0A4U9BZP4	Elongation factor G	A0A4U9BZP4_STRPY	TRUE	TRUE	LYDGSYHDVDSSETAFK	617.10516	617.10516	4	LYD[HAyneDE]GSYHDVDS	26536	2	TRUE
Insoluble	*fusA*	A0A4U9BZP4	Elongation factor G	A0A4U9BZP4_STRPY	TRUE	TRUE	LYDGSYHDVDSSETAFK	628.30566	628.30566	4	LYD[HAyneDE]GSYHDVDS	25988	3	TRUE
Insoluble	*fusA*	A0A4U9BZP4	Elongation factor G	A0A4U9BZP4_STRPY	TRUE	TRUE	SAQPAILEPMMLVTITAPEDNLGDVMGH	444.4697	444.4697	4	SAQPAILEPM[Oxidation (M	55242	1	TRUE
Insoluble	*fusA*	A0A4U9BZP4	Elongation factor G	A0A4U9BZP4_STRPY	TRUE	TRUE	SAQPAILEPMMLVTITAPEDNLGDVMGH	444.08618	444.08618	4	SAQPAILEPM[Oxidation (M	54969	1	TRUE
Insoluble	*fusA*	A0A4U9BZP4	Elongation factor G	A0A4U9BZP4_STRPY	TRUE	TRUE	SAQPAILEPMMLVTITAPEDNLGDVMGH	444.08618	444.08618	4	SAQPAILEPM[Oxidation (M	54959	1	TRUE
Insoluble	*fusA*	A0A4U9BZP4	Elongation factor G	A0A4U9BZP4_STRPY	TRUE	TRUE	SAQPAILEPMMLVTITAPEDNLGDVMGH	368.23892	368.23892	4	SAQPAILEPM[Oxidation (M	55812	1	TRUE
Insoluble	*fusA*	A0A4U9BZP4	Elongation factor G	A0A4U9BZP4_STRPY	TRUE	TRUE	SAQPAILEPMMLVTITAPEDNLGDVMGH	368.23892	368.23892	4	SAQPAILEPM[Oxidation (M	55755	1	TRUE
Insoluble	*fusA*	A0A4U9BZP4	Elongation factor G	A0A4U9BZP4_STRPY	TRUE	TRUE	SAQPAILEPMMLVTITAPEDNLGDVMGH	408.68384	408.68384	4	SAQPAILEPM[Oxidation (M	55780	1	FALSE
Insoluble	*fusA*	A0A4U9BZP4	Elongation factor G	A0A4U9BZP4_STRPY	TRUE	TRUE	LQANAHPIQLPIGAEDDFR	2414.0454	2414.0454	4	LQANAHPIQLPIGAEDD[HA	44326	1	TRUE
Insoluble	*fusA*	A0A4U9BZP4	Elongation factor G	A0A4U9BZP4_STRPY	TRUE	TRUE	LQANAHPIQLPIGAEDDFR	2414.0454	2414.0454	4	LQANAHPIQLPIGAEDD[HA	44300	1	TRUE
Insoluble	*fusA*	A0A4U9BZP4	Elongation factor G	A0A4U9BZP4_STRPY	TRUE	TRUE	LQANAHPIQLPIGAEDDFR	2414.0454	2414.0454	4	LQANAHPIQLPIGAEDD[HA	44362	1	TRUE
Insoluble	*fusA*	A0A4U9BZP4	Elongation factor G	A0A4U9BZP4_STRPY	TRUE	TRUE	LQANAHPIQLPIGAEDDFR	2414.0454	2414.0454	4	LQANAHPIQLPIGAEDD[HA	44381	1	FALSE
Insoluble	*fusA*	A0A4U9BZP4	Elongation factor G	A0A4U9BZP4_STRPY	TRUE	TRUE	LQANAHPIQLPIGAEDDFR	2414.0454	2414.0454	4	LQANAHPIQLPIGAED[HAy	44835	1	TRUE
Insoluble	*fusA*	A0A4U9BZP4	Elongation factor G	A0A4U9BZP4_STRPY	TRUE	TRUE	LQANAHPIQLPIGAEDDFR	1426.7407	1426.7407	3	LQANAHPIQLPIGAEDD[HA	44355	1	TRUE
Insoluble	*fusA*	A0A4U9BZP4	Elongation factor G	A0A4U9BZP4_STRPY	TRUE	TRUE	VNIIDTPGHVDFTIEVQR	791.92334	791.92334	4	VNIID[HAyneDE]TPGHVDF	47968	1	TRUE
Insoluble	*fusA*	A0A4U9BZP4	Elongation factor G	A0A4U9BZP4_STRPY	TRUE	TRUE	VNIIDTPGHVDFTIEVQR	792.251	792.251	4	VNIID[HAyneDE]TPGHVDF	48090	2	TRUE
Insoluble	*fusA*	A0A4U9BZP4	Elongation factor G	A0A4U9BZP4_STRPY	TRUE	TRUE	VNIIDTPGHVDFTIEVQR	462.39606	462.39606	3	VNIIDTPGHVD[HAyneDE]F	46771	1	TRUE
Insoluble	*fusA*	A0A4U9BZP4	Elongation factor G	A0A4U9BZP4_STRPY	TRUE	TRUE	VNIIDTPGHVDFTIEVQR	371.9551	371.9551	4	VNIIDTPGHVD[HAyneDE]F	46706	1	TRUE
Insoluble	*fusA*	A0A4U9BZP4	Elongation factor G	A0A4U9BZP4_STRPY	TRUE	TRUE	VNIIDTPGHVDFTIEVQR	792.2675	792.2675	4	VNIIDTPGHVD[HAyneDE]F	48427	1	TRUE
Insoluble	*fusA*	A0A4U9BZP4	Elongation factor G	A0A4U9BZP4_STRPY	TRUE	TRUE	VNIIDTPGHVDFTIEVQR	371.9551	371.9551	4	VNIIDTPGHVD[HAyneDE]F	46728	1	TRUE
Insoluble	*fusA*	A0A4U9BZP4	Elongation factor G	A0A4U9BZP4_STRPY	TRUE	TRUE	LIEAVAETDEDLMMK	1577.1207	1577.1207	3	LIEAVAETD[HAyneDE]EDLM	46870	1	TRUE
Insoluble	*fusA*	A0A4U9BZP4	Elongation factor G	A0A4U9BZP4_STRPY	TRUE	TRUE	LIEAVAETDEDLMMK	983.8705	983.8705	3	LIEAVAETDED[HAyneDE]LM	45356	1	TRUE
Insoluble	*fusA*	A0A4U9BZP4	Elongation factor G	A0A4U9BZP4_STRPY	TRUE	TRUE	LIEAVAETDEDLMMK	947.1935	947.1935	3	LIEAVAETDED[HAyneDE]LM	37666	1	TRUE
Insoluble	*fusA*	A0A4U9BZP4	Elongation factor G	A0A4U9BZP4_STRPY	TRUE	TRUE	LIEAVAETDEDLMMK	941.0842	941.0842	3	LIEAVAETDED[HAyneDE]LM	38018	1	TRUE
Insoluble	*fusA*	A0A4U9BZP4	Elongation factor G	A0A4U9BZP4_STRPY	TRUE	TRUE	LIEAVAETDEDLMMK	439.41083	439.41083	2	LIEAVAETD[HAyneDE]EDLM	39700	1	TRUE
Insoluble	*fusA*	A0A4U9BZP4	Elongation factor G	A0A4U9BZP4_STRPY	TRUE	TRUE	LIEAVAETDEDLMMK	439.422	439.422	2	LIEAVAETD[HAyneDE]EDLM	39490	1	TRUE
Insoluble	*fusA*	A0A4U9BZP4	Elongation factor G	A0A4U9BZP4_STRPY	TRUE	TRUE	LIEAVAETDEDLMMK	466.86053	466.86053	2	LIEAVAETD[HAyneDE]EDLM	37828	1	TRUE
Insoluble	*fusA*	A0A4U9BZP4	Elongation factor G	A0A4U9BZP4_STRPY	TRUE	TRUE	LIEAVAETDEDLMMK	335.65842	335.65842	4	LIEAVAETD[HAyneDE]EDLM	37833	1	TRUE
Insoluble	*fusA*	A0A4U9BZP4	Elongation factor G	A0A4U9BZP4_STRPY	TRUE	TRUE	GVNPDTDAEEERPASDEEPFAALAFK	744.93964	744.93964	4	GVNPD[HAyneDE]TDAEEE	48799	1	TRUE
Insoluble	*fusA*	A0A4U9BZP4	Elongation factor G	A0A4U9BZP4_STRPY	TRUE	TRUE	GVNPDTDAEEERPASDEEPFAALAFK	640.1209	640.1209	4	GVNPDTDAEEERPASD[HAy	50944	1	TRUE
Insoluble	*fusA*	A0A4U9BZP4	Elongation factor G	A0A4U9BZP4_STRPY	TRUE	TRUE	GVNPDTDAEEERPASDEEPFAALAFK	402.7887	402.7887	4	GVNPDTD[HAyneDE]AEEE	48743	2	TRUE
Insoluble	*fusA*	A0A4U9BZP4	Elongation factor G	A0A4U9BZP4_STRPY	TRUE	TRUE	GVNPDTDAEEERPASDEEPFAALAFK	619.3594	619.3594	4	GVNPDTD[HAyneDE]AEEE	51531	1	TRUE
Insoluble	*fusA*	A0A4U9BZP4	Elongation factor G	A0A4U9BZP4_STRPY	TRUE	TRUE	IGADFLYSVQTLHDR	743.254	743.254	3	IGAD[HAyneDE]FLYSVQTLH	49574	1	TRUE
Insoluble	*fusA*	A0A4U9BZP4	Elongation factor G	A0A4U9BZP4_STRPY	TRUE	TRUE	IGADFLYSVQTLHDR	716.3933	716.3933	3	IGAD[HAyneDE]FLYSVQTLH	49583	1	TRUE
Insoluble	*fusA*	A0A4U9BZP4	Elongation factor G	A0A4U9BZP4_STRPY	TRUE	TRUE	IGADFLYSVQTLHDR	743.254	743.254	3	IGADFLYSVQTLHD[HAyneD	49905	1	TRUE
Insoluble	*fusA*	A0A4U9BZP4	Elongation factor G	A0A4U9BZP4_STRPY	TRUE	TRUE	IGADFLYSVQTLHDR	645.8926	645.8926	4	IGAD[HAyneDE]FLYSVQTLH	49691	1	TRUE
Insoluble	*fusA*	A0A4U9BZP4	Elongation factor G	A0A4U9BZP4_STRPY	TRUE	TRUE	IGADFLYSVQTLHDR	641.68024	641.68024	4	IGADFLYSVQTLHD[HAyneD	49913	1	TRUE
Insoluble	*fusA*	A0A4U9BZP4	Elongation factor G	A0A4U9BZP4_STRPY	TRUE	TRUE	YLEGEEITNDELIAGIR	413.6063	413.6063	3	YLEGEEITND[HAyneDE]ELI	58115	1	TRUE
Insoluble	*fusA*	A0A4U9BZP4	Elongation factor G	A0A4U9BZP4_STRPY	TRUE	TRUE	YLEGEEITNDELIAGIR	412.8675	412.8675	3	YLEGEEITND[HAyneDE]ELI	58209	1	TRUE
Insoluble	*fusA*	A0A4U9BZP4	Elongation factor G	A0A4U9BZP4_STRPY	TRUE	TRUE	YLEGEEITNDELIAGIR	384.30182	384.30182	3	YLEGEEITND[HAyneDE]ELI	58061	3	TRUE
Insoluble	*fusA*	A0A4U9BZP4	Elongation factor G	A0A4U9BZP4_STRPY	TRUE	TRUE	YLEGEEITNDELIAGIR	412.8675	412.8675	3	YLEGEEITND[HAyneDE]ELI	58197	2	FALSE
Insoluble	*fusA*	A0A4U9BZP4	Elongation factor G	A0A4U9BZP4_STRPY	TRUE	TRUE	YLEGEEITNDELIAGIR	368.6516	368.6516	2	YLEGEEITND[HAyneDE]ELI	58046	1	TRUE
Insoluble	*fusA*	A0A4U9BZP4	Elongation factor G	A0A4U9BZP4_STRPY	TRUE	TRUE	YLEGEEITNDELIAGIR	536.41516	536.41516	2	YLEGEEITND[HAyneDE]ELI	58055	1	TRUE
Insoluble	*fusA*	A0A4U9BZP4	Elongation factor G	A0A4U9BZP4_STRPY	TRUE	TRUE	AEIYTNDLGTDILEEDIPEEYLEQAQEYR	263.56046	263.56046	4	AEIYTNDLGTDILEED[HAyn	74648	1	TRUE
Insoluble	*fusA*	A0A4U9BZP4	Elongation factor G	A0A4U9BZP4_STRPY	TRUE	TRUE	IMTDPFVGR	720.2405	720.2405	2	IM[Oxidation (M)]TD[HAyn	41299	1	TRUE
Insoluble	*fusA*	A0A4U9BZP4	Elongation factor G	A0A4U9BZP4_STRPY	TRUE	TRUE	IMTDPFVGR	447.2345	447.2345	3	IM[Oxidation (M)]TD[HAyn	41290	1	TRUE
Insoluble	*fusA*	A0A4U9BZP4	Elongation factor G	A0A4U9BZP4_STRPY	TRUE	TRUE	GTFMMVFDHYEDVPK	332.92618	332.92618	4	GTFM[Oxidation (M)]M[Oxi	36245	2	TRUE
Insoluble	*fusA*	A0A4U9BZP4	Elongation factor G	A0A4U9BZP4_STRPY	TRUE	TRUE	VDGMEAHGNSQIVR	798.5019	798.5019	4	VD[HAyneDE]GM[Oxidatio	12040	1	TRUE
Insoluble	*fusA*	A0A4U9BZP4	Elongation factor G	A0A4U9BZP4_STRPY	TRUE	TRUE	VDGMEAHGNSQIVR	369.82272	369.82272	4	VD[HAyneDE]GM[Oxidatio	11299	1	TRUE
Insoluble	*fusA*	A0A4U9BZP4	Elongation factor G	A0A4U9BZP4_STRPY	TRUE	TRUE	VDGMEAHGNSQIVR	810.8106	810.8106	4	VD[HAyneDE]GM[Oxidatio	12046	1	TRUE
Insoluble	*fusA*	A0A4U9BZP4	Elongation factor G	A0A4U9BZP4_STRPY	TRUE	TRUE	VDGMEAHGNSQIVR	830.2532	830.2532	4	VD[HAyneDE]GM[Oxidatio	12383	1	FALSE
Insoluble	*fusA*	A0A4U9BZP4	Elongation factor G	A0A4U9BZP4_STRPY	TRUE	TRUE	VDGMEAHGNSQIVR	651.7659	651.7659	3	VD[HAyneDE]GM[Oxidatio	11246	1	TRUE
Insoluble	*fusA*	A0A4U9BZP4	Elongation factor G	A0A4U9BZP4_STRPY	TRUE	TRUE	LAEEDPTFR	718.76404	718.76404	3	LAEED[HAyneDE]PTFR	29403	1	TRUE
Insoluble	*fusA*	A0A4U9BZP4	Elongation factor G	A0A4U9BZP4_STRPY	TRUE	TRUE	QEIETVYAGDIAAAVGLK	325.70874	325.70874	3	QEIETVYAGD[HAyneDE]IAA	64327	1	TRUE
Insoluble	*fusA*	A0A4U9BZP4	Elongation factor G	A0A4U9BZP4_STRPY	TRUE	TRUE	NIGIMAHVDAGK	478.82257	478.82257	3	NIGIM[Oxidation (M)]AHVD	19375	1	TRUE
Insoluble	*fusA*	A0A4U9BZP4	Elongation factor G	A0A4U9BZP4_STRPY	TRUE	TRUE	DTTTGDSLTDEK	206.01564	206.01564	3	D[HAyneDE]TTTGDSLTDEK	19111	1	TRUE
Insoluble	*fusA*	A0A4U9BZP4	Elongation factor G	A0A4U9BZP4_STRPY	TRUE	TRUE	DTTTGDSLTDEK	258.05472	258.05472	3	D[HAyneDE]TTTGDSLTDEK	18421	2	TRUE
Insoluble	*glmS*	A0A5S4TPH3	Glutamine--fructose-6-phosphate amino	A0A5S4TPH3_STRPY	TRUE	TRUE	HGTISLIEEDTPVIALISSSQLVASHTR	355.38705	355.38705	3	HGTISLIEED[HAyneDE]TPV	69778	1	TRUE
Insoluble	*glmS*	A0A5S4TPH3	Glutamine--fructose-6-phosphate amino	A0A5S4TPH3_STRPY	TRUE	TRUE	GYDSAGIFVANANQTNLIK	263.77286	263.77286	3	GYD[HAyneDE]SAGIFVANA	49748	1	TRUE
Insoluble	*glmS*	A0A5S4TPH3	Glutamine--fructose-6-phosphate amino	A0A5S4TPH3_STRPY	TRUE	TRUE	SPLLIGLGEGYNMVCSDAMAMIR	602.5478	602.5478	3	SPLLIGLGEGYNM[Oxidatio	69793	3	TRUE
Insoluble	*glmS*	A0A5S4TPH3	Glutamine--fructose-6-phosphate amino	A0A5S4TPH3_STRPY	TRUE	TRUE	SPLLIGLGEGYNMVCSDAMAMIR	414.93683	414.93683	3	SPLLIGLGEGYNMVC[Carba	64603	1	TRUE
Insoluble	*glmS*	A0A5S4TPH3	Glutamine--fructose-6-phosphate amino	A0A5S4TPH3_STRPY	TRUE	TRUE	GAHVLTVVEEGLDREGDDIIVNK	392.12366	392.12366	4	GAHVLTVVEEGLDREGDD[H	47403	1	TRUE
Insoluble	*glmS*	A0A5S4TPH3	Glutamine--fructose-6-phosphate amino	A0A5S4TPH3_STRPY	TRUE	TRUE	GAHVLTVVEEGLDREGDDIIVNK	350.7625	350.7625	3	GAHVLTVVEEGLDREGDD[H	43596	1	TRUE
Insoluble	*glmS*	A0A5S4TPH3	Glutamine--fructose-6-phosphate amino	A0A5S4TPH3_STRPY	TRUE	TRUE	GAHVLTVVEEGLDREGDDIIVNK	238.92952	238.92952	5	GAHVLTVVEEGLDREGDD[H	48924	2	TRUE
Insoluble	*glmS*	A0A5S4TPH3	Glutamine--fructose-6-phosphate amino	A0A5S4TPH3_STRPY	TRUE	TRUE	DSYTAELDLSDIGK	336.75244	336.75244	2	D[HAyneDE]SYTAELDLSDI	51528	1	TRUE
Insoluble	*glmS*	A0A5S4TPH3	Glutamine--fructose-6-phosphate amino	A0A5S4TPH3_STRPY	TRUE	TRUE	DSYTAELDLSDIGK	336.75244	336.75244	2	D[HAyneDE]SYTAELDLSDI	51561	1	TRUE
Insoluble	*glnA*	A0A4U9TI37	Glutamate--ammonia ligase	A0A4U9TI37_STRPY	TRUE	TRUE	NEAGIIDLPSTLHNALK	395.80576	395.80576	3	NEAGIID[HAyneDE]LPSTLH	49511	1	TRUE
Soluble	*gloA2*	A0A4V6KIZ4	Aldoketomutase	A0A4V6KIZ4_STRPY	TRUE	TRUE	DLDQSVAFYTSAFPFK	278.93518	278.93518	2	D[HAyneDE]LDQSVAFYTSA	67464	1	TRUE
Soluble	*gloA2*	A0A4V6KIZ4	Aldoketomutase	A0A4V6KIZ4_STRPY	TRUE	TRUE	DLDQSVAFYTSAFPFK	278.934	278.934	2	D[HAyneDE]LDQSVAFYTSA	67430	1	TRUE
Soluble	*gloA2*	A0A4V6KIZ4	Aldoketomutase	A0A4V6KIZ4_STRPY	TRUE	TRUE	DLDQSVAFYTSAFPFK	270.45956	270.45956	2	DLD[HAyneDE]QSVAFYTSA	72350	1	TRUE
Insoluble	*gpmA*	A0A5S4TEK8	2,3-bisphosphoglycerate-dependent pho	A0A5S4TEK8_STRPY	TRUE	TRUE	DDEYSAHK	175.47087	175.47087	3	D[HAyneDE]DEYSAHK	9553	1	TRUE
Insoluble	*gpmA*	A0A5S4TEK8	2,3-bisphosphoglycerate-dependent pho	A0A5S4TEK8_STRPY	TRUE	TRUE	DDEYSAHK	177.71353	177.71353	3	D[HAyneDE]DEYSAHK	9536	1	TRUE
Insoluble	*gpmA*	A0A5S4TEK8	2,3-bisphosphoglycerate-dependent pho	A0A5S4TEK8_STRPY	TRUE	TRUE	YADLDPALIPDAENLK	626.194	626.194	2	YADLDPALIPD[HAyneDE]A	56077	1	TRUE
Insoluble	*gpmA*	A0A5S4TEK8	2,3-bisphosphoglycerate-dependent pho	A0A5S4TEK8_STRPY	TRUE	TRUE	YADLDPALIPDAENLK	528.5012	528.5012	3	YAD[HAyneDE]LDPALIPDA	56617	1	TRUE
Insoluble	*gpmA*	A0A5S4TEK8	2,3-bisphosphoglycerate-dependent pho	A0A5S4TEK8_STRPY	TRUE	TRUE	YADLDPALIPDAENLK	530.2339	530.2339	3	YAD[HAyneDE]LDPALIPDA	56206	1	TRUE
Insoluble	*gpmA*	A0A5S4TEK8	2,3-bisphosphoglycerate-dependent pho	A0A5S4TEK8_STRPY	TRUE	TRUE	YADLDPALIPDAENLK	528.64294	528.64294	3	YAD[HAyneDE]LDPALIPDA	56557	1	TRUE
Insoluble	*gpmA*	A0A5S4TEK8	2,3-bisphosphoglycerate-dependent pho	A0A5S4TEK8_STRPY	TRUE	TRUE	YADLDPALIPDAENLK	528.63824	528.63824	3	YAD[HAyneDE]LDPALIPDA	56577	1	FALSE
Insoluble	*gpmA*	A0A5S4TEK8	2,3-bisphosphoglycerate-dependent pho	A0A5S4TEK8_STRPY	TRUE	TRUE	YADLDPALIPDAENLK	626.194	626.194	2	YAD[HAyneDE]LDPALIPDA	56106	1	TRUE
Soluble	*gpmA*	A0A5S4TEK8	2,3-bisphosphoglycerate-dependent pho	A0A5S4TEK8_STRPY	TRUE	TRUE	ANLFTGWADVDLSEK	395.6483	395.6483	3	ANLFTGWADVD[HAyneDE]	60016	1	TRUE
Soluble	*gpmA*	A0A5S4TEK8	2,3-bisphosphoglycerate-dependent pho	A0A5S4TEK8_STRPY	TRUE	TRUE	ANLFTGWADVDLSEK	388.87988	388.87988	3	ANLFTGWADVD[HAyneDE]	60028	1	TRUE
Soluble	*gpmA*	A0A5S4TEK8	2,3-bisphosphoglycerate-dependent pho	A0A5S4TEK8_STRPY	TRUE	TRUE	YADLDPALIPDAENLK	1125.9878	1125.9878	2	YADLDPALIPD[HAyneDE]A	51765	1	TRUE
Soluble	*gpmA*	A0A5S4TEK8	2,3-bisphosphoglycerate-dependent pho	A0A5S4TEK8_STRPY	TRUE	TRUE	YADLDPALIPDAENLK	888.5819	888.5819	3	YAD[HAyneDE]LDPALIPDA	52302	1	TRUE
Soluble	*gpmA*	A0A5S4TEK8	2,3-bisphosphoglycerate-dependent pho	A0A5S4TEK8_STRPY	TRUE	TRUE	YADLDPALIPDAENLK	888.5815	888.5815	3	YAD[HAyneDE]LDPALIPDA	52343	1	TRUE
Soluble	*gpmA*	A0A5S4TEK8	2,3-bisphosphoglycerate-dependent pho	A0A5S4TEK8_STRPY	TRUE	TRUE	YADLDPALIPDAENLK	914.7378	914.7378	3	YADLD[HAyneDE]PALIPDA	51999	1	TRUE
Soluble	*gpmA*	A0A5S4TEK8	2,3-bisphosphoglycerate-dependent pho	A0A5S4TEK8_STRPY	TRUE	TRUE	YADLDPALIPDAENLK	906.386	906.386	3	YADLD[HAyneDE]PALIPDA	51890	1	TRUE
Soluble	*gpmA*	A0A5S4TEK8	2,3-bisphosphoglycerate-dependent pho	A0A5S4TEK8_STRPY	TRUE	TRUE	YADLDPALIPDAENLK	906.386	906.386	3	YADLD[HAyneDE]PALIPDA	51909	1	TRUE
Soluble	*gpmA*	A0A5S4TEK8	2,3-bisphosphoglycerate-dependent pho	A0A5S4TEK8_STRPY	TRUE	TRUE	YADLDPALIPDAENLK	906.3933	906.3933	3	YADLDPALIPD[HAyneDE]A	51866	1	TRUE
Soluble	*gpmA*	A0A5S4TEK8	2,3-bisphosphoglycerate-dependent pho	A0A5S4TEK8_STRPY	TRUE	TRUE	YADLDPALIPDAENLK	91.32357	91.32357	4	YADLDPALIPD[HAyneDE]A	51965	1	TRUE
Soluble	*gpmA*	A0A5S4TEK8	2,3-bisphosphoglycerate-dependent pho	A0A5S4TEK8_STRPY	TRUE	TRUE	EAGIEFDLAFTSVLTR	372.48163	372.48163	3	EAGIEFD[HAyneDE]LAFTS	72208	1	TRUE
Soluble	*gpmA*	A0A5S4TEK8	2,3-bisphosphoglycerate-dependent pho	A0A5S4TEK8_STRPY	TRUE	TRUE	EAGIEFDLAFTSVLTR	361.7909	361.7909	3	EAGIEFD[HAyneDE]LAFTS	71688	1	TRUE
Soluble	*gpmA*	A0A5S4TEK8	2,3-bisphosphoglycerate-dependent pho	A0A5S4TEK8_STRPY	TRUE	TRUE	EAGIEFDLAFTSVLTR	341.80865	341.80865	2	EAGIEFD[HAyneDE]LAFTS	71710	1	TRUE
Soluble	*gpmA*	A0A5S4TEK8	2,3-bisphosphoglycerate-dependent pho	A0A5S4TEK8_STRPY	TRUE	TRUE	EAGIEFDLAFTSVLTR	341.77924	341.77924	2	EAGIEFD[HAyneDE]LAFTS	71703	1	TRUE
Soluble	*gpmA*	A0A5S4TEK8	2,3-bisphosphoglycerate-dependent pho	A0A5S4TEK8_STRPY	TRUE	TRUE	DDEYSAHK	636.4986	636.4986	3	D[HAyneDE]DEYSAHK	10024	1	TRUE
Soluble	*gpmA*	A0A5S4TEK8	2,3-bisphosphoglycerate-dependent pho	A0A5S4TEK8_STRPY	TRUE	TRUE	DDEYSAHK	605.0329	605.0329	3	D[HAyneDE]DEYSAHK	7921	1	TRUE
Soluble	*gpmA*	A0A5S4TEK8	2,3-bisphosphoglycerate-dependent pho	A0A5S4TEK8_STRPY	TRUE	TRUE	DDEYSAHK	240.69156	240.69156	3	D[HAyneDE]DEYSAHK	9431	1	TRUE
Soluble	*gpmA*	A0A5S4TEK8	2,3-bisphosphoglycerate-dependent pho	A0A5S4TEK8_STRPY	TRUE	TRUE	DDEYSAHK	480.71463	480.71463	3	D[HAyneDE]DEYSAHK	9477	1	FALSE
Soluble	*gpmA*	A0A5S4TEK8	2,3-bisphosphoglycerate-dependent pho	A0A5S4TEK8_STRPY	TRUE	TRUE	DDEYSAHK	605.031	605.031	3	D[HAyneDE]DEYSAHK	7749	1	FALSE
Soluble	*gpmA*	A0A5S4TEK8	2,3-bisphosphoglycerate-dependent pho	A0A5S4TEK8_STRPY	TRUE	TRUE	GLSDDEIMDVEIPNFPPLVFELDEK	253.63643	253.63643	4	GLSDDEIM[Oxidation (M)]I	73089	1	TRUE
Soluble	*greA*	A0A4Q1PRF5	Transcription elongation factor GreA	A0A4Q1PRF5_STRPY	TRUE	TRUE	TVIVQEVGTTDK	420.08432	420.08432	3	TVIVQEVGTTD[HAyneDE]K	25082	1	TRUE
Soluble	*greA*	A0A4Q1PRF5	Transcription elongation factor GreA	A0A4Q1PRF5_STRPY	TRUE	TRUE	DTYHIVGAAGADIFSGK	337.6419	337.6419	3	D[HAyneDE]TYHIVGAAGAD	37195	1	TRUE
Soluble	*greA*	A0A4Q1PRF5	Transcription elongation factor GreA	A0A4Q1PRF5_STRPY	TRUE	TRUE	YAEIIDSDAVAK	416.55295	416.55295	3	YAEIID[HAyneDE]SDAVAK	31121	1	TRUE
Soluble	*greA*	A0A4Q1PRF5	Transcription elongation factor GreA	A0A4Q1PRF5_STRPY	TRUE	TRUE	YAEIIDSDAVAK	545.67255	545.67255	3	YAEIID[HAyneDE]SDAVAK	29683	1	TRUE
Soluble	*greA*	A0A4Q1PRF5	Transcription elongation factor GreA	A0A4Q1PRF5_STRPY	TRUE	TRUE	YAEIIDSDAVAK	545.7328	545.7328	3	YAEIID[HAyneDE]SDAVAK	29692	1	TRUE
Soluble	*greA*	A0A4Q1PRF5	Transcription elongation factor GreA	A0A4Q1PRF5_STRPY	TRUE	TRUE	YAEIIDSDAVAK	287.67984	287.67984	2	YAEIID[HAyneDE]SDAVAK	29702	1	TRUE
Soluble	*greA*	A0A4Q1PRF5	Transcription elongation factor GreA	A0A4Q1PRF5_STRPY	TRUE	TRUE	DEQAFVEGQISTLETK	174.90248	174.90248	3	D[HAyneDE]EQAFVEGQIST	42267	3	TRUE
Soluble	*greA*	A0A4Q1PRF5	Transcription elongation factor GreA	A0A4Q1PRF5_STRPY	TRUE	TRUE	IESPAATYDVEIISVEK	277.1708	277.1708	2	IESPAATYD[HAyneDE]VEIIS	46762	1	TRUE
Soluble	*greA*	A0A4Q1PRF5	Transcription elongation factor GreA	A0A4Q1PRF5_STRPY	TRUE	TRUE	VRIESPAATYDVEIISVEK	445.675	445.675	3	VRIESPAATYD[HAyneDE]VE	39955	1	TRUE
Insoluble	*groES*	A0A4U7IKP7	Co-chaperonin GroES	A0A4U7IKP7_STRPY	TRUE	TRUE	ESDIIAIVTK	318.00375	318.00375	2	ESD[HAyneDE]IIAIVTK	51078	1	TRUE
Insoluble	*groL*	A0A4U9I1I0	Chaperonin GroEL	A0A4U9I1I0_STRPY	TRUE	TRUE	MVADLENPFILITDK	333.04715	333.04715	3	M[Oxidation (M)]VAD[HAyn	70840	1	TRUE
Insoluble	*groL*	A0A4U9I1I0	Chaperonin GroEL	A0A4U9I1I0_STRPY	TRUE	TRUE	MVADLENPFILITDK	336.81146	336.81146	3	M[Oxidation (M)]VAD[HAyn	71084	2	TRUE
Insoluble	*groL*	A0A4U9I1I0	Chaperonin GroEL	A0A4U9I1I0_STRPY	TRUE	TRUE	EIELEDHFENMGAK	544.6426	544.6426	4	EIELED[HAyneDE]HFENM[	30078	1	TRUE
Insoluble	*groL*	A0A4U9I1I0	Chaperonin GroEL	A0A4U9I1I0_STRPY	TRUE	TRUE	GVDMLADTVK	284.634	284.634	2	GVD[HAyneDE]M[Oxidatio	30909	1	TRUE
Insoluble	*groL*	A0A4U9I1I0	Chaperonin GroEL	A0A4U9I1I0_STRPY	TRUE	TRUE	MVADLENPFILITDKK	205.84831	205.84831	4	M[Oxidation (M)]VAD[HAyn	59040	3	TRUE
Insoluble	*groL*	A0A4U9I1I0	Chaperonin GroEL	A0A4U9I1I0_STRPY	TRUE	TRUE	DATMTALGQAAK	693.79364	693.79364	2	D[HAyneDE]ATM[Oxidation	22666	1	TRUE
Soluble	*groL*	A0A4U9I1I0	Chaperonin GroEL	A0A4U9I1I0_STRPY	TRUE	TRUE	EIELEDHFENMGAK	564.74274	564.74274	4	EIELED[HAyneDE]HFENM[	26673	1	TRUE
Soluble	*groL*	A0A4U9I1I0	Chaperonin GroEL	A0A4U9I1I0_STRPY	TRUE	TRUE	EIELEDHFENMGAK	695.041	695.041	3	EIELED[HAyneDE]HFENM[	26666	1	TRUE
Soluble	*groL*	A0A4U9I1I0	Chaperonin GroEL	A0A4U9I1I0_STRPY	TRUE	TRUE	GYLSQYMVTDNEK	308.8992	308.8992	3	GYLSQYM[Oxidation (M)]V	28655	2	TRUE
Soluble	*groL*	A0A4U9I1I0	Chaperonin GroEL	A0A4U9I1I0_STRPY	TRUE	TRUE	MVADLENPFILITDK	317.0786	317.0786	3	M[Oxidation (M)]VAD[HAyn	66698	1	TRUE
Soluble	*groL*	A0A4U9I1I0	Chaperonin GroEL	A0A4U9I1I0_STRPY	TRUE	TRUE	MVADLENPFILITDK	320.6955	320.6955	3	M[Oxidation (M)]VAD[HAyn	66606	1	TRUE
Soluble	*groL*	A0A4U9I1I0	Chaperonin GroEL	A0A4U9I1I0_STRPY	TRUE	TRUE	GVDMLADTVK	106.36969	106.36969	2	GVD[HAyneDE]M[Oxidatio	27446	1	TRUE
Insoluble	*guaA*	A0A5S4TFU8	GMP synthase [glutamine-hydrolyzing]	A0A5S4TFU8_STRPY	TRUE	TRUE	AIGDQLTCIFVDHGLLR	1081.6041	1081.6041	3	AIGD[HAyneDE]QLTC[Carb	70075	1	TRUE
Insoluble	*guaB*	A0A5S4TD94	Inosine-5'-monophosphate dehydrogena	A0A5S4TD94_STRPY	TRUE	TRUE	FISDYNAPISEHMTSEHLVTAAVGTDLETA	311.98135	311.98135	4	FISD[HAyneDE]YNAPISEH	40200	1	TRUE
Soluble	*guaB*	A0A5S4TD94	Inosine-5'-monophosphate dehydrogena	A0A5S4TD94_STRPY	TRUE	TRUE	FISDYNAPISEHMTSEHLVTAAVGTDLETA	508.1342	508.1342	4	FISD[HAyneDE]YNAPISEH	36182	1	TRUE
Soluble	*guaB*	A0A5S4TD94	Inosine-5'-monophosphate dehydrogena	A0A5S4TD94_STRPY	TRUE	TRUE	FISDYNAPISEHMTSEHLVTAAVGTDLETA	492.37344	492.37344	4	FISD[HAyneDE]YNAPISEH	36192	1	TRUE
Soluble	*guaB*	A0A5S4TD94	Inosine-5'-monophosphate dehydrogena	A0A5S4TD94_STRPY	TRUE	TRUE	LPLVDNSGR	531.7421	531.7421	3	LPLVD[HAyneDE]NSGR	30883	1	TRUE
Soluble	*guaB*	A0A5S4TD94	Inosine-5'-monophosphate dehydrogena	A0A5S4TD94_STRPY	TRUE	TRUE	LPLVDNSGR	543.0784	543.0784	3	LPLVD[HAyneDE]NSGR	31025	2	TRUE
Soluble	*guaB*	A0A5S4TD94	Inosine-5'-monophosphate dehydrogena	A0A5S4TD94_STRPY	TRUE	TRUE	LPLVDNSGR	531.81647	531.81647	3	LPLVD[HAyneDE]NSGR	30908	1	TRUE
Soluble	*guaB*	A0A5S4TD94	Inosine-5'-monophosphate dehydrogena	A0A5S4TD94_STRPY	TRUE	TRUE	LPLVDNSGR	257.8747	257.8747	2	LPLVD[HAyneDE]NSGR	29430	1	TRUE
Soluble	*guaB*	A0A5S4TD94	Inosine-5'-monophosphate dehydrogena	A0A5S4TD94_STRPY	TRUE	TRUE	LPLVDNSGR	275.44724	275.44724	2	LPLVD[HAyneDE]NSGR	30949	1	TRUE
Insoluble	*hpt*	A0A4U7GHG9	Hypoxanthine phosphoribosyltransferase	A0A4U7GHG9_STRPY	TRUE	TRUE	DIIIVEDIIDTGR	207.71184	207.71184	3	DIIIVED[HAyneDE]IIDTGR	71256	1	TRUE
Insoluble	*ileS*	A0A5S4TG33	Isoleucine--tRNA ligase	A0A5S4TG33_STRPY	TRUE	TRUE	SYNAFVCDHSAK	353.03354	353.03354	2	SYNAFVC[Carbamidometh	32780	3	TRUE
Insoluble	*infA*	A0A2W5AM85	Translation initiation factor IF-1	A0A2W5AM85_STRPY	TRUE	TRUE	EDVIEIEGK	643.39166	643.39166	2	ED[HAyneDE]VIEIEGK	37358	1	TRUE
Insoluble	*infA*	A0A2W5AM85	Translation initiation factor IF-1	A0A2W5AM85_STRPY	TRUE	TRUE	EDVIEIEGK	655.06177	655.06177	2	ED[HAyneDE]VIEIEGK	37388	1	TRUE
Insoluble	*infB*	A0A5S4TEW0	Translation initiation factor IF-2	A0A5S4TEW0_STRPY	TRUE	TRUE	SAEPSTPVSITGLNETPMAGDHFAVYADE	4144.0347	4144.0347	4	SAEPSTPVSITGLNETPM[Ox	44219	1	TRUE
Insoluble	*infB*	A0A5S4TEW0	Translation initiation factor IF-2	A0A5S4TEW0_STRPY	TRUE	TRUE	SAEPSTPVSITGLNETPMAGDHFAVYADE	4167.9727	4167.9727	4	SAEPSTPVSITGLNETPM[Ox	44231	1	TRUE
Insoluble	*ldh*	A0A4U7H3E6	L-lactate dehydrogenase	A0A4U7H3E6_STRPY	TRUE	TRUE	AIIDEAFAK	220.52412	220.52412	2	AIID[HAyneDE]EAFAK	40700	1	TRUE
Insoluble	*ldh*	A0A4U7H3E6	L-lactate dehydrogenase	A0A4U7H3E6_STRPY	TRUE	TRUE	TQGDAEDLSHALAFTSPK	740.09314	740.09314	3	TQGDAED[HAyneDE]LSHA	40780	1	TRUE
Insoluble	*ldh*	A0A4U7H3E6	L-lactate dehydrogenase	A0A4U7H3E6_STRPY	TRUE	TRUE	TQGDAEDLSHALAFTSPK	686.5284	686.5284	3	TQGD[HAyneDE]AEDLSHA	42167	1	TRUE
Insoluble	*ldh*	A0A4U7H3E6	L-lactate dehydrogenase	A0A4U7H3E6_STRPY	TRUE	TRUE	TQGDAEDLSHALAFTSPK	692.6524	692.6524	3	TQGD[HAyneDE]AEDLSHA	42156	2	TRUE
Insoluble	*ldh*	A0A4U7H3E6	L-lactate dehydrogenase	A0A4U7H3E6_STRPY	TRUE	TRUE	DAAYSIINK	270.79495	270.79495	2	D[HAyneDE]AAYSIINK	33339	1	TRUE
Insoluble	*ldh*	A0A4U7H3E6	L-lactate dehydrogenase	A0A4U7H3E6_STRPY	TRUE	TRUE	IYAADYSDCHDADLVVLTAGAPQKPGETR	601.1249	601.1249	4	IYAADYSDC[Carbamidome	39434	1	TRUE
Insoluble	*ldh*	A0A4U7H3E6	L-lactate dehydrogenase	A0A4U7H3E6_STRPY	TRUE	TRUE	IYAADYSDCHDADLVVLTAGAPQKPGETR	528.5421	528.5421	4	IYAAD[HAyneDE]YSDC[Car	37049	1	TRUE
Insoluble	*ldh*	A0A4U7H3E6	L-lactate dehydrogenase	A0A4U7H3E6_STRPY	TRUE	TRUE	IYAADYSDCHDADLVVLTAGAPQKPGETR	523.7428	523.7428	4	IYAADYSD[HAyneDE]C[Car	37166	1	TRUE
Insoluble	*ldh*	A0A4U7H3E6	L-lactate dehydrogenase	A0A4U7H3E6_STRPY	TRUE	TRUE	IYAADYSDCHDADLVVLTAGAPQKPGETR	498.7405	498.7405	4	IYAADYSDC[Carbamidome	37042	1	TRUE
Insoluble	*ldh*	A0A4U7H3E6	L-lactate dehydrogenase	A0A4U7H3E6_STRPY	TRUE	TRUE	VIGSGTSLDSAR	494.0245	494.0245	3	VIGSGTSLD[HAyneDE]SAR	24590	1	TRUE
Insoluble	*ldh*	A0A4U7H3E6	L-lactate dehydrogenase	A0A4U7H3E6_STRPY	TRUE	TRUE	VIGSGTSLDSAR	478.57913	478.57913	3	VIGSGTSLD[HAyneDE]SAR	25201	1	TRUE
Insoluble	*ldh*	A0A4U7H3E6	L-lactate dehydrogenase	A0A4U7H3E6_STRPY	TRUE	TRUE	VIGSGTSLDSAR	189.33516	189.33516	2	VIGSGTSLD[HAyneDE]SAR	24655	1	TRUE
Insoluble	*ldh*	A0A4U7H3E6	L-lactate dehydrogenase	A0A4U7H3E6_STRPY	TRUE	TRUE	VIGSGTSLDSAR	190.79796	190.79796	2	VIGSGTSLD[HAyneDE]SAR	24648	1	TRUE
Insoluble	*ldh*	A0A4U7H3E6	L-lactate dehydrogenase	A0A4U7H3E6_STRPY	TRUE	TRUE	IYAADYSDCHDADLVVLTAGAPQK	246.57585	246.57585	4	IYAADYSDC[Carbamidome	43453	2	TRUE
Insoluble	*lepA*	A0A5S4TG26	Elongation factor 4	A0A5S4TG26_STRPY	TRUE	TRUE	TFDVTEVGIFTPK	250.86362	250.86362	2	TFD[HAyneDE]VTEVGIFTPK	60002	3	TRUE
Insoluble	*leuS*	A0A5S4TFT5	Leucine--tRNA ligase	A0A5S4TFT5_STRPY	TRUE	TRUE	TFYDHTAIEPK	303.02716	303.02716	3	TFYD[HAyneDE]HTAIEPK	21573	1	TRUE
Insoluble	*leuS*	A0A5S4TFT5	Leucine--tRNA ligase	A0A5S4TFT5_STRPY	TRUE	TRUE	TFYDHTAIEPK	293.55774	293.55774	3	TFYD[HAyneDE]HTAIEPK	21583	1	TRUE
Insoluble	*lpdA*	A0A5S4TMZ1	Dihydrolipoyl dehydrogenase	A0A5S4TMZ1_STRPY	TRUE	TRUE	LVLTSDDILDLR	201.22737	201.22737	3	LVLTSDD[HAyneDE]ILDLR	64711	2	TRUE
Insoluble	*lpdA*	A0A5S4TMZ1	Dihydrolipoyl dehydrogenase	A0A5S4TMZ1_STRPY	TRUE	TRUE	LVLTSDDILDLR	147.6222	147.6222	3	LVLTSDD[HAyneDE]ILDLR	64741	1	TRUE
Soluble	*lpdA*	A0A5S4TMZ1	Dihydrolipoyl dehydrogenase	A0A5S4TMZ1_STRPY	TRUE	TRUE	TTEDLEAAGLEVPK	1082.2687	1082.2687	3	TTED[HAyneDE]LEAAGLEV	36096	1	TRUE
Soluble	*lpdA*	A0A5S4TMZ1	Dihydrolipoyl dehydrogenase	A0A5S4TMZ1_STRPY	TRUE	TRUE	TTEDLEAAGLEVPK	1082.2687	1082.2687	3	TTED[HAyneDE]LEAAGLEV	36075	1	TRUE
Soluble	*lpdA*	A0A5S4TMZ1	Dihydrolipoyl dehydrogenase	A0A5S4TMZ1_STRPY	TRUE	TRUE	TTEDLEAAGLEVPK	785.02435	785.02435	2	TTED[HAyneDE]LEAAGLEV	36055	1	TRUE
Soluble	*lpdA*	A0A5S4TMZ1	Dihydrolipoyl dehydrogenase	A0A5S4TMZ1_STRPY	TRUE	TRUE	TTEDLEAAGLEVPK	399.02066	399.02066	2	TTED[HAyneDE]LEAAGLEV	37940	1	TRUE
Soluble	*lpdA*	A0A5S4TMZ1	Dihydrolipoyl dehydrogenase	A0A5S4TMZ1_STRPY	TRUE	TRUE	EQYGDVLIGK	375.00742	375.00742	2	EQYGD[HAyneDE]VLIGK	37504	1	TRUE
Soluble	*lpdA*	A0A5S4TMZ1	Dihydrolipoyl dehydrogenase	A0A5S4TMZ1_STRPY	TRUE	TRUE	EQYGDVLIGK	391.4424	391.4424	2	EQYGD[HAyneDE]VLIGK	37592	1	TRUE
Soluble	*lpdA*	A0A5S4TMZ1	Dihydrolipoyl dehydrogenase	A0A5S4TMZ1_STRPY	TRUE	TRUE	EQYGDVLIGK	391.4424	391.4424	2	EQYGD[HAyneDE]VLIGK	37566	1	TRUE
Soluble	*lpdA*	A0A5S4TMZ1	Dihydrolipoyl dehydrogenase	A0A5S4TMZ1_STRPY	TRUE	TRUE	GINLASTNYTIDMDK	322.47278	322.47278	3	GINLASTNYTIDM[Oxidatior	34997	1	TRUE
Insoluble	*manL*	A0A4U7FZ73	PTS system mannose-specific EIIAB com	A0A4U7FZ73_STRPY	TRUE	TRUE	ALPEDLNPVEETAATEK	274.83115	274.83115	3	ALPED[HAyneDE]LNPVEET	42011	1	TRUE
Insoluble	*manL*	A0A4U7FZ73	PTS system mannose-specific EIIAB com	A0A4U7FZ73_STRPY	TRUE	TRUE	ALPEDLNPVEETAATEK	521.9206	521.9206	3	ALPED[HAyneDE]LNPVEET	42176	2	TRUE
Insoluble	*manL*	A0A4U7FZ73	PTS system mannose-specific EIIAB com	A0A4U7FZ73_STRPY	TRUE	TRUE	IIVASDEVAQDDLRK	317.44498	317.44498	3	IIVASD[HAyneDE]EVAQDD	27401	2	TRUE
Insoluble	*manL*	A0A4U7FZ73	PTS system mannose-specific EIIAB com	A0A4U7FZ73_STRPY	TRUE	TRUE	IIVASDEVAQDDLRK	267.77084	267.77084	3	IIVASD[HAyneDE]EVAQDD	27409	1	TRUE
Insoluble	*manL*	A0A4U7FZ73	PTS system mannose-specific EIIAB com	A0A4U7FZ73_STRPY	TRUE	TRUE	EDVATFEK	376.27274	376.27274	2	ED[HAyneDE]VATFEK	26578	1	TRUE
Insoluble	*manN*	A0A4U9BZ12	PTS mannose/fructose/sorbose transport	A0A4U9BZ12_STRPY	TRUE	TRUE	DMSGGILQDITK	195.0201	195.0201	3	D[HAyneDE]M[Oxidation (M	44535	2	TRUE

Insoluble	*manN*	A0A4U9BZ12	PTS mannose/fructose/sorbose transport	A0A4U9BZ12_STRPY	TRUE	TRUE	LYTSPEDR	294.69586	294.69586	2	LYTSPED[HAyneDE]R	20817	1	TRUE
Soluble	*map*	A0A4U9C6M0	Methionine aminopeptidase	A0A4U9C6M0_STRPY	TRUE	TRUE	TLDGGLSCQYEHQFVITK	726.1398	726.1398	3	TLD[HAyneDE]GGLSC[Carl	32648	1	TRUE
Soluble	*map*	A0A4U9C6M0	Methionine aminopeptidase	A0A4U9C6M0_STRPY	TRUE	TRUE	TLDGGLSCQYEHQFVITK	726.1139	726.1139	3	TLD[HAyneDE]GGLSC[Carl	32557	1	TRUE
Soluble	*map*	A0A4U9C6M0	Methionine aminopeptidase	A0A4U9C6M0_STRPY	TRUE	TRUE	TLDGGLSCQYEHQFVITK	720.803	720.803	3	TLD[HAyneDE]GGLSC[Carl	32798	1	TRUE
Soluble	*map*	A0A4U9C6M0	Methionine aminopeptidase	A0A4U9C6M0_STRPY	TRUE	TRUE	TLDGGLSCQYEHQFVITK	698.53534	698.53534	4	TLD[HAyneDE]GGLSC[Carl	32603	1	TRUE
Soluble	*map*	A0A4U9C6M0	Methionine aminopeptidase	A0A4U9C6M0_STRPY	TRUE	TRUE	TLDGGLSCQYEHQFVITK	700.50885	700.50885	4	TLD[HAyneDE]GGLSC[Carl	32756	1	TRUE
Soluble	*map*	A0A4U9C6M0	Methionine aminopeptidase	A0A4U9C6M0_STRPY	TRUE	TRUE	TLDGGLSCQYEHQFVITK	698.53534	698.53534	4	TLD[HAyneDE]GGLSC[Carl	32595	1	TRUE
Soluble	*map*	A0A4U9C6M0	Methionine aminopeptidase	A0A4U9C6M0_STRPY	TRUE	TRUE	VDMVLSEPLDK	1368.6543	1368.6543	3	VD[HAyneDE]M[Oxidation	32571	1	TRUE
Soluble	*map*	A0A4U9C6M0	Methionine aminopeptidase	A0A4U9C6M0_STRPY	TRUE	TRUE	VDMVLSEPLDK	1368.6	1368.6	3	VD[HAyneDE]M[Oxidation	32493	1	TRUE
Soluble	*map*	A0A4U9C6M0	Methionine aminopeptidase	A0A4U9C6M0_STRPY	TRUE	TRUE	VDMVLSEPLDK	1334.7289	1334.7289	3	VD[HAyneDE]M[Oxidation	32584	1	TRUE
Soluble	*map*	A0A4U9C6M0	Methionine aminopeptidase	A0A4U9C6M0_STRPY	TRUE	TRUE	VDMVLSEPLDK	1337.5143	1337.5143	3	VD[HAyneDE]M[Oxidation	32661	1	FALSE
Soluble	*map*	A0A4U9C6M0	Methionine aminopeptidase	A0A4U9C6M0_STRPY	TRUE	TRUE	SIVDVAALDFDNVPEMK	426.73718	426.73718	3	SIVDVAALDFD[HAyneDE]N	58447	1	TRUE
Soluble	*map*	A0A4U9C6M0	Methionine aminopeptidase	A0A4U9C6M0_STRPY	TRUE	TRUE	SIVDVAALDFDNVPEMK	397.98264	397.98264	3	SIVDVAALDFD[HAyneDE]N	58055	1	TRUE
Soluble	*map*	A0A4U9C6M0	Methionine aminopeptidase	A0A4U9C6M0_STRPY	TRUE	TRUE	AGDFLAGIHIGLR	330.73178	330.73178	3	AGD[HAyneDE]FLAGIHIGL	54654	1	TRUE
Soluble	*map*	A0A4U9C6M0	Methionine aminopeptidase	A0A4U9C6M0_STRPY	TRUE	TRUE	AGDFLAGIHIGLR	304.31244	304.31244	3	AGD[HAyneDE]FLAGIHIGL	54668	1	TRUE
Soluble	*map*	A0A4U9C6M0	Methionine aminopeptidase	A0A4U9C6M0_STRPY	TRUE	TRUE	AGDFLAGIHIGLR	321.86954	321.86954	3	AGD[HAyneDE]FLAGIHIGL	53067	1	TRUE
Soluble	*map*	A0A4U9C6M0	Methionine aminopeptidase	A0A4U9C6M0_STRPY	TRUE	TRUE	CKEDNVLPLQIGVDGHMMDYPYATCCGI	1867.8591	1867.8591	5	C[Carbamidomethyl (C)]K	44714	1	TRUE
Soluble	*map*	A0A4U9C6M0	Methionine aminopeptidase	A0A4U9C6M0_STRPY	TRUE	TRUE	CKEDNVLPLQIGVDGHMMDYPYATCCGI	1231.1848	1231.1848	5	C[Carbamidomethyl (C)]K	54611	1	TRUE
Soluble	*map*	A0A4U9C6M0	Methionine aminopeptidase	A0A4U9C6M0_STRPY	TRUE	TRUE	CKEDNVLPLQIGVDGHMMDYPYATCCGI	421.5936	421.5936	5	C[Carbamidomethyl (C)]K	49995	1	TRUE
Soluble	*map*	A0A4U9C6M0	Methionine aminopeptidase	A0A4U9C6M0_STRPY	TRUE	TRUE	WTGSYTGGLADSCWAYAVGTPSDEIK	568.78064	568.78064	4	WTGSYTGGLAD[HAyneDE]	64625	2	TRUE
Soluble	*map*	A0A4U9C6M0	Methionine aminopeptidase	A0A4U9C6M0_STRPY	TRUE	TRUE	EDNVLPLQIGVDGHMMDYPYATCCGLN	370.0405	370.0405	4	EDNVLPLQIGVD[HAyneDE	56665	1	TRUE
Insoluble	*napIr*	Q54A60	Glyceraldehyde-3-phosphate dehydroger	Q54A60_STRPY	TRUE	TRUE	TVVFNTNHDILDGTETVISGASCTTNCLAP	421.70026	421.70026	4	TVVFNTNHD[HAyneDE]ILD	67475	3	TRUE
Insoluble	*napIr*	Q54A60	Glyceraldehyde-3-phosphate dehydroger	Q54A60_STRPY	TRUE	TRUE	GLMTTIHAYTGDQMILDGPHR	644.2266	644.2266	4	GLM[Oxidation (M)]TTIHAY	30136	1	TRUE
Insoluble	*napIr*	Q54A60	Glyceraldehyde-3-phosphate dehydroger	Q54A60_STRPY	TRUE	TRUE	AASNDSFGYTEDPIVSSDIVGVSYGSLFDA	824.07764	824.07764	4	AASND[HAyneDE]SFGYTED	73444	1	TRUE
Insoluble	*napIr*	Q54A60	Glyceraldehyde-3-phosphate dehydroger	Q54A60_STRPY	TRUE	TRUE	AASNDSFGYTEDPIVSSDIVGVSYGSLFDA	766.7814	766.7814	4	AASNDSFGYTEDPIVSSDIV(	73941	1	TRUE
Insoluble	*napIr*	Q54A60	Glyceraldehyde-3-phosphate dehydroger	Q54A60_STRPY	TRUE	TRUE	AASNDSFGYTEDPIVSSDIVGVSYGSLFDA	738.68695	738.68695	4	AASNDSFGYTEDPIVSSD[HA	73934	1	TRUE
Insoluble	*napIr*	Q54A60	Glyceraldehyde-3-phosphate dehydroger	Q54A60_STRPY	TRUE	TRUE	AASNDSFGYTEDPIVSSDIVGVSYGSLFDA	496.33542	496.33542	3	AASND[HAyneDE]SFGYTED	73383	1	TRUE
Insoluble	*napIr*	Q54A60	Glyceraldehyde-3-phosphate dehydroger	Q54A60_STRPY	TRUE	TRUE	VPVPTGSVTELVVTLDK	776.8601	776.8601	3	VPVPTGSVTELVVTLD[HAyn	68089	1	TRUE
Insoluble	*napIr*	Q54A60	Glyceraldehyde-3-phosphate dehydroger	Q54A60_STRPY	TRUE	TRUE	VPVPTGSVTELVVTLDK	566.4989	566.4989	4	VPVPTGSVTELVVTLD[HAyn	68097	1	TRUE
Insoluble	*napIr*	Q54A60	Glyceraldehyde-3-phosphate dehydroger	Q54A60_STRPY	TRUE	TRUE	VPVPTGSVTELVVTLDK	568.60583	568.60583	4	VPVPTGSVTELVVTLD[HAyn	68111	1	TRUE
Insoluble	*napIr*	Q54A60	Glyceraldehyde-3-phosphate dehydroger	Q54A60_STRPY	TRUE	TRUE	NVSVDEINAAMK	726.3707	726.3707	3	NVSVD[HAyneDE]EINAAMI	26370	1	TRUE
Insoluble	*napIr*	Q54A60	Glyceraldehyde-3-phosphate dehydroger	Q54A60_STRPY	TRUE	TRUE	FDGTVEVK	636.60785	636.60785	2	FD[HAyneDE]GTVEVK	27767	2	TRUE
Insoluble	*napIr*	Q54A60	Glyceraldehyde-3-phosphate dehydroger	Q54A60_STRPY	TRUE	TRUE	FDGTVEVK	628.09546	628.09546	2	FD[HAyneDE]GTVEVK	23971	1	TRUE
Insoluble	*napIr*	Q54A60	Glyceraldehyde-3-phosphate dehydroger	Q54A60_STRPY	TRUE	TRUE	FDGTVEVK	505.264	505.264	3	FD[HAyneDE]GTVEVK	24018	1	TRUE
Insoluble	*napIr*	Q54A60	Glyceraldehyde-3-phosphate dehydroger	Q54A60_STRPY	TRUE	TRUE	FDGTVEVK	505.22766	505.22766	3	FD[HAyneDE]GTVEVK	24011	1	TRUE
Insoluble	*napIr*	Q54A60	Glyceraldehyde-3-phosphate dehydroger	Q54A60_STRPY	TRUE	TRUE	LDGAAQR	384.8442	384.8442	2	LD[HAyneDE]GAAQR	15929	1	TRUE
Insoluble	*napIr*	Q54A60	Glyceraldehyde-3-phosphate dehydroger	Q54A60_STRPY	TRUE	TRUE	LDGAAQR	384.8442	384.8442	2	LD[HAyneDE]GAAQR	15943	1	TRUE
Insoluble	*napIr*	Q54A60	Glyceraldehyde-3-phosphate dehydroger	Q54A60_STRPY	TRUE	TRUE	LDGAAQR	372.77634	372.77634	3	LD[HAyneDE]GAAQR	15887	1	TRUE
Insoluble	*napIr*	Q54A60	Glyceraldehyde-3-phosphate dehydroger	Q54A60_STRPY	TRUE	TRUE	LDGAAQR	362.4261	362.4261	3	LD[HAyneDE]GAAQR	15898	1	TRUE
Insoluble	*napIr*	Q54A60	Glyceraldehyde-3-phosphate dehydroger	Q54A60_STRPY	TRUE	TRUE	INDLTDPNMLAHLLK	498.18915	498.18915	3	INDLTD[HAyneDE]PNM[Ox	46392	2	TRUE
Insoluble	*napIr*	Q54A60	Glyceraldehyde-3-phosphate dehydroger	Q54A60_STRPY	TRUE	TRUE	INDLTDPNMLAHLLK	483.31763	483.31763	3	INDLTD[HAyneDE]PNM[Ox	46591	1	TRUE
Insoluble	*napIr*	Q54A60	Glyceraldehyde-3-phosphate dehydroger	Q54A60_STRPY	TRUE	TRUE	INDLTDPNMLAHLLK	336.51596	336.51596	4	INDLTD[HAyneDE]PNM[Ox	46542	1	TRUE
Insoluble	*napIr*	Q54A60	Glyceraldehyde-3-phosphate dehydroger	Q54A60_STRPY	TRUE	TRUE	INDLTDPNMLAHLLK	403.76636	403.76636	3	IND[HAyneDE]LTDPNM[Ox	51231	1	TRUE
Insoluble	*napIr*	Q54A60	Glyceraldehyde-3-phosphate dehydroger	Q54A60_STRPY	TRUE	TRUE	YDTTQGR	442.0691	442.0691	2	YD[HAyneDE]TTQGR	11916	1	TRUE
Insoluble	*napIr*	Q54A60	Glyceraldehyde-3-phosphate dehydroger	Q54A60_STRPY	TRUE	TRUE	YDTTQGR	264.79657	264.79657	3	YD[HAyneDE]TTQGR	11941	1	TRUE
Insoluble	*napIr*	Q54A60	Glyceraldehyde-3-phosphate dehydroger	Q54A60_STRPY	TRUE	TRUE	YDTTQGR	194.94084	194.94084	3	YD[HAyneDE]TTQGR	13861	1	TRUE
Soluble	*napIr*	Q54A60	Glyceraldehyde-3-phosphate dehydroger	Q54A60_STRPY	TRUE	TRUE	GLMTTIHAYTGDQMILDGPHR	594.23846	594.23846	4	GLM[Oxidation (M)]TTIHAY	26960	1	TRUE
Soluble	*napIr*	Q54A60	Glyceraldehyde-3-phosphate dehydroger	Q54A60_STRPY	TRUE	TRUE	TVVFNTNHDILDGTETVISGASCTTNCLAP	2915.1616	2915.1616	5	TVVFNTNHDILD[HAyneDE]	55373	2	TRUE
Soluble	*napIr*	Q54A60	Glyceraldehyde-3-phosphate dehydroger	Q54A60_STRPY	TRUE	TRUE	TVVFNTNHDILDGTETVISGASCTTNCLAP	300.3171	300.3171	3	TVVFNTNHD[HAyneDE]ILD	59378	2	TRUE
Soluble	*napIr*	Q54A60	Glyceraldehyde-3-phosphate dehydroger	Q54A60_STRPY	TRUE	TRUE	AASNDSFGYTEDPIVSSDIVGVSYGSLFDA	800.8464	800.8464	4	AASND[HAyneDE]SFGYTED	69447	1	TRUE
Soluble	*napIr*	Q54A60	Glyceraldehyde-3-phosphate dehydroger	Q54A60_STRPY	TRUE	TRUE	AASNDSFGYTEDPIVSSDIVGVSYGSLFDA	854.89966	854.89966	4	AASND[HAyneDE]SFGYTED	69305	1	TRUE
Soluble	*napIr*	Q54A60	Glyceraldehyde-3-phosphate dehydroger	Q54A60_STRPY	TRUE	TRUE	AASNDSFGYTEDPIVSSDIVGVSYGSLFDA	241.7938	241.7938	3	AASNDSFGYTEDPIVSSD[HA	69734	1	TRUE
Soluble	*napIr*	Q54A60	Glyceraldehyde-3-phosphate dehydroger	Q54A60_STRPY	TRUE	TRUE	AASNDSFGYTEDPIVSSDIVGVSYGSLFDA	127.26895	127.26895	3	AASNDSFGYTED[HAyneDE	71092	1	TRUE
Soluble	*napIr*	Q54A60	Glyceraldehyde-3-phosphate dehydroger	Q54A60_STRPY	TRUE	TRUE	VPVPTGSVTELVVTLDK	1106.4517	1106.4517	3	VPVPTGSVTELVVTLD[HAyn	63855	1	TRUE
Soluble	*napIr*	Q54A60	Glyceraldehyde-3-phosphate dehydroger	Q54A60_STRPY	TRUE	TRUE	VPVPTGSVTELVVTLDK	900.7951	900.7951	4	VPVPTGSVTELVVTLD[HAyn	63866	1	TRUE
Soluble	*napIr*	Q54A60	Glyceraldehyde-3-phosphate dehydroger	Q54A60_STRPY	TRUE	TRUE	VPVPTGSVTELVVTLDK	900.7997	900.7997	4	VPVPTGSVTELVVTLD[HAyn	64025	1	TRUE
Soluble	*napIr*	Q54A60	Glyceraldehyde-3-phosphate dehydroger	Q54A60_STRPY	TRUE	TRUE	VPVPTGSVTELVVTLDK	273.70746	273.70746	2	VPVPTGSVTELVVTLD[HAyn	63902	1	TRUE
Soluble	*napIr*	Q54A60	Glyceraldehyde-3-phosphate dehydroger	Q54A60_STRPY	TRUE	TRUE	FDGTVEVK	1473.8386	1473.8386	2	FD[HAyneDE]GTVEVK	21034	1	TRUE
Soluble	*napIr*	Q54A60	Glyceraldehyde-3-phosphate dehydroger	Q54A60_STRPY	TRUE	TRUE	FDGTVEVK	125.55188	125.55188	2	FD[HAyneDE]GTVEVK	24344	1	TRUE
Soluble	*napIr*	Q54A60	Glyceraldehyde-3-phosphate dehydroger	Q54A60_STRPY	TRUE	TRUE	FDGTVEVK	1473.8356	1473.8356	2	FD[HAyneDE]GTVEVK	21115	1	TRUE
Soluble	*napIr*	Q54A60	Glyceraldehyde-3-phosphate dehydroger	Q54A60_STRPY	TRUE	TRUE	FDGTVEVK	118.03829	118.03829	2	FD[HAyneDE]GTVEVK	24317	1	FALSE
Soluble	*napIr*	Q54A60	Glyceraldehyde-3-phosphate dehydroger	Q54A60_STRPY	TRUE	TRUE	FDGTVEVK	118.03829	118.03829	2	FD[HAyneDE]GTVEVK	24305	1	FALSE
Soluble	*napIr*	Q54A60	Glyceraldehyde-3-phosphate dehydroger	Q54A60_STRPY	TRUE	TRUE	FDGTVEVK	1133.6119	1133.6119	3	FD[HAyneDE]GTVEVK	21044	1	TRUE
Soluble	*napIr*	Q54A60	Glyceraldehyde-3-phosphate dehydroger	Q54A60_STRPY	TRUE	TRUE	FDGTVEVK	1133.6003	1133.6003	3	FD[HAyneDE]GTVEVK	21117	1	TRUE
Soluble	*napIr*	Q54A60	Glyceraldehyde-3-phosphate dehydroger	Q54A60_STRPY	TRUE	TRUE	FDGTVEVK	209.91438	209.91438	3	FD[HAyneDE]GTVEVK	24370	1	TRUE
Soluble	*napIr*	Q54A60	Glyceraldehyde-3-phosphate dehydroger	Q54A60_STRPY	TRUE	TRUE	FDGTVEVK	209.91438	209.91438	3	FD[HAyneDE]GTVEVK	24353	1	FALSE
Soluble	*napIr*	Q54A60	Glyceraldehyde-3-phosphate dehydroger	Q54A60_STRPY	TRUE	TRUE	NVSVDEINAAMK	903.99896	903.99896	3	NVSVD[HAyneDE]EINAAMI	23293	1	TRUE
Soluble	*napIr*	Q54A60	Glyceraldehyde-3-phosphate dehydroger	Q54A60_STRPY	TRUE	TRUE	INDLTDPNMLAHLLK	734.9633	734.9633	3	INDLTD[HAyneDE]PNM[Ox	42634	1	TRUE
Soluble	*napIr*	Q54A60	Glyceraldehyde-3-phosphate dehydroger	Q54A60_STRPY	TRUE	TRUE	INDLTDPNMLAHLLK	734.9633	734.9633	3	INDLTD[HAyneDE]PNM[Ox	42623	1	TRUE
Soluble	*napIr*	Q54A60	Glyceraldehyde-3-phosphate dehydroger	Q54A60_STRPY	TRUE	TRUE	INDLTDPNMLAHLLK	616.7434	616.7434	4	INDLTD[HAyneDE]PNM[Ox	42537	1	TRUE
Soluble	*napIr*	Q54A60	Glyceraldehyde-3-phosphate dehydroger	Q54A60_STRPY	TRUE	TRUE	YDTTQGR	738.4318	738.4318	2	YD[HAyneDE]TTQGR	9974	1	TRUE
Soluble	*napIr*	Q54A60	Glyceraldehyde-3-phosphate dehydroger	Q54A60_STRPY	TRUE	TRUE	YDTTQGR	160.75407	160.75407	3	YD[HAyneDE]TTQGR	11891	2	TRUE
Insoluble	*nox*	A0A5S4TKQ5	NADH oxidase	A0A5S4TKQ5_STRPY	TRUE	TRUE	LALTDIFFLPHFNK	571.72766	571.72766	3	LALTD[HAyneDE]IFFLPHFN	70017	1	TRUE
Insoluble	*nox*	A0A5S4TKQ5	NADH oxidase	A0A5S4TKQ5_STRPY	TRUE	TRUE	EVVLIDVVDTCLAGYYDRDLTDLMAK	256.4647	256.4647	4	EVVLID[HAyneDE]VVDTC[C	74194	1	TRUE
Insoluble	*nox*	A0A5S4TKQ5	NADH oxidase	A0A5S4TKQ5_STRPY	TRUE	TRUE	EVVLIDVVDTCLAGYYDRDLTDLMAK	240.3824	240.3824	3	EVVLIDVVD[HAyneDE]TC[C	76626	1	TRUE
Insoluble	*nox*	A0A5S4TKQ5	NADH oxidase	A0A5S4TKQ5_STRPY	TRUE	TRUE	DTNYIALASNAVR	199.7758	199.7758	2	D[HAyneDE]TNYIALASNAVI	48056	1	TRUE
Soluble	*nrdF*	A0A5S4TDF4	Ribonucleoside-diphosphate reductase s	A0A5S4TDF4_STRPY	TRUE	TRUE	VFGGLTLLDTMQSETGVEAIR	637.9729	637.9729	3	VFGGLTLLD[HAyneDE]TM(	58097	1	TRUE
Soluble	*nrdF*	A0A5S4TDF4	Ribonucleoside-diphosphate reductase s	A0A5S4TDF4_STRPY	TRUE	TRUE	VFGGLTLLDTMQSETGVEAIR	643.79474	643.79474	3	VFGGLTLLD[HAyneDE]TM(	58107	1	TRUE
Soluble	*nrdF*	A0A5S4TDF4	Ribonucleoside-diphosphate reductase s	A0A5S4TDF4_STRPY	TRUE	TRUE	VFGGLTLLDTMQSETGVEAIR	626.3282	626.3282	3	VFGGLTLLD[HAyneDE]TM(	59748	1	TRUE
Soluble	*nrdF*	A0A5S4TDF4	Ribonucleoside-diphosphate reductase s	A0A5S4TDF4_STRPY	TRUE	TRUE	VFGGLTLLDTMQSETGVEAIR	643.77606	643.77606	3	VFGGLTLLD[HAyneDE]TM(	58090	1	FALSE
Soluble	*nrdF*	A0A5S4TDF4	Ribonucleoside-diphosphate reductase s	A0A5S4TDF4_STRPY	TRUE	TRUE	VFGGLTLLDTMQSETGVEAIR	507.42725	507.42725	4	VFGGLTLLD[HAyneDE]TM(	59724	1	TRUE
Soluble	*nrdF*	A0A5S4TDF4	Ribonucleoside-diphosphate reductase s	A0A5S4TDF4_STRPY	TRUE	TRUE	VFGGLTLLDTMQSETGVEAIR	507.42725	507.42725	4	VFGGLTLLD[HAyneDE]TM(	59744	1	TRUE
Soluble	*nrdF*	A0A5S4TDF4	Ribonucleoside-diphosphate reductase s	A0A5S4TDF4_STRPY	TRUE	TRUE	VFGGLTLLDTMQSETGVEAIR	502.1856	502.1856	4	VFGGLTLLD[HAyneDE]TM(	59686	1	TRUE
Soluble	*nrdF*	A0A5S4TDF4	Ribonucleoside-diphosphate reductase s	A0A5S4TDF4_STRPY	TRUE	TRUE	VFGGLTLLDTMQSETGVEAIR	437.17392	437.17392	4	VFGGLTLLD[HAyneDE]TM(	58027	2	FALSE
Soluble	*nrdF*	A0A5S4TDF4	Ribonucleoside-diphosphate reductase s	A0A5S4TDF4_STRPY	TRUE	TRUE	IPLSNDLDDWRK	220.4721	220.4721	3	IPLSND[HAyneDE]LDDWRI	33004	1	TRUE
Insoluble	*nusG*	A0A4U7GC48	Transcription termination/antitermination	A0A4U7GC48_STRPY	TRUE	TRUE	MLDSFDK	212.04277	212.04277	3	M[Oxidation (M)]LD[HAyne	27762	1	TRUE
Soluble	*pcp*	A0A4U7GRI8	Pyrrolidone-carboxylate peptidase	A0A4U7GRI8_STRPY	TRUE	TRUE	ILVTGFDPFGGEAINPALEAIK	563.71075	563.71075	4	ILVTGFD[HAyneDE]PFGGE	71268	1	TRUE
Soluble	*pcp*	A0A4U7GRI8	Pyrrolidone-carboxylate peptidase	A0A4U7GRI8_STRPY	TRUE	TRUE	AMVAAIHQAGLPASVSNTAGTFVCNHLM	2975.9067	2975.9067	5	AM[Oxidation (M)]VAAIHQ	56219	1	TRUE
Insoluble	*pepC*	A0A4U9TB08	Aminopeptidase	A0A4U9TB08_STRPY	TRUE	TRUE	QDAQILR	1014.0474	1014.0474	3	QD[HAyneDE]AQILR	25442	1	TRUE
Insoluble	*pepC*	A0A4U9TB08	Aminopeptidase	A0A4U9TB08_STRPY	TRUE	TRUE	QDAQILR	1014.0377	1014.0377	3	QD[HAyneDE]AQILR	25580	1	TRUE
Insoluble	*pepC*	A0A4U9TB08	Aminopeptidase	A0A4U9TB08_STRPY	TRUE	TRUE	QDAQILR	739.8702	739.8702	2	QD[HAyneDE]AQILR	25429	1	TRUE
Insoluble	*pepC*	A0A4U9TB08	Aminopeptidase	A0A4U9TB08_STRPY	TRUE	TRUE	LDYSESLMTHAMVLTGVDLDETGKPLK	511.22638	511.22638	4	LD[HAyneDE]YSESLM[Oxid	41346	3	TRUE
Insoluble	*pepV*	A0A5S4TGP8	Dipeptidase PepV	A0A5S4TGP8_STRPY	TRUE	TRUE	GVAFGAMFPGDENTMHQANEYMPLENI	686.7519	686.7519	4	GVAFGAMFPGD[HAyneDE]	50166	1	TRUE
Insoluble	*pepV*	A0A5S4TGP8	Dipeptidase PepV	A0A5S4TGP8_STRPY	FALSE	FALSE	DYLHVAGEVLHEDFAAEK	344.9955	344.9955	4	DYLHVAGEVLHED[HAyneD	54443	1	TRUE
Insoluble	*pepV*	A0A5S4TGP8	Dipeptidase PepV	A0A5S4TGP8_STRPY	FALSE	FALSE	DYLHVAGEVLHEDFAAEK	273.70584	273.70584	3	DYLHVAGEVLHED[HAyneD	55154	2	TRUE
Insoluble	*pfkB*	A0A4U7GWZ7	Tagatose-6-phosphate kinase	A0A4U7GWZ7_STRPY	TRUE	TRUE	LDIASTATGFLGGFTGR	205.25366	205.25366	2	LD[HAyneDE]IASTATGFLG(	65990	2	TRUE
Soluble	*pflB*	A0A4V6EK79	Formate acetyltransferase	A0A4V6EK79_STRPY	TRUE	TRUE	VFHEVLSMDDAAEAISSK	358.79007	358.79007	3	VFHEVLSM[Oxidation (M)]I	28790	1	TRUE
Insoluble	*pgi*	A0A5D3IK27	Glucose-6-phosphate isomerase	A0A5D3IK27_STRPY	TRUE	TRUE	VLESFAGQHEIDFLQGQVTEADK	253.7479	253.7479	3	VLESFAGQHEID[HAyneDE]	51054	1	TRUE
Insoluble	*pgi*	A0A5D3IK27	Glucose-6-phosphate isomerase	A0A5D3IK27_STRPY	TRUE	TRUE	VLESFAGQHEIDFLQGQVTEADK	238.94928	238.94928	4	VLESFAGQHEID[HAyneDE]	51049	1	TRUE
Insoluble	*pgk*	A0A5S4TDF1	Phosphoglycerate kinase	A0A5S4TDF1_STRPY	TRUE	TRUE	SIIGGGDSAAAAINLGR	461.3141	461.3141	3	SIIGGGD[HAyneDE]SAAAA	45607	1	TRUE
Insoluble	*pgk*	A0A5S4TDF1	Phosphoglycerate kinase	A0A5S4TDF1_STRPY	TRUE	TRUE	SIIGGGDSAAAAINLGR	202.43243	202.43243	2	SIIGGGD[HAyneDE]SAAAA	45620	1	TRUE
Insoluble	*pgk*	A0A5S4TDF1	Phosphoglycerate kinase	A0A5S4TDF1_STRPY	FALSE	FALSE	LILPVDSK	421.50073	421.50073	3	LILPVD[HAyneDE]SK	42421	1	TRUE
Insoluble	*pgk*	A0A5S4TDF1	Phosphoglycerate kinase	A0A5S4TDF1_STRPY	FALSE	FALSE	LILPVDSK	336.4967	336.4967	2	LILPVD[HAyneDE]SK	42418	1	TRUE
Insoluble	*pgk*	A0A5S4TDF1	Phosphoglycerate kinase	A0A5S4TDF1_STRPY	TRUE	TRUE	LGQDVVFPGVTR	346.65103	346.65103	3	LGQD[HAyneDE]VVFPGVTI	47979	1	TRUE
Insoluble	*pgk*	A0A5S4TDF1	Phosphoglycerate kinase	A0A5S4TDF1_STRPY	TRUE	TRUE	LGQDVVFPGVTR	343.71588	343.71588	3	LGQD[HAyneDE]VVFPGVTI	48034	1	TRUE
Insoluble	*pgk*	A0A5S4TDF1	Phosphoglycerate kinase	A0A5S4TDF1_STRPY	TRUE	TRUE	LGQDVVFPGVTR	185.37712	185.37712	2	LGQD[HAyneDE]VVFPGVTI	48057	1	TRUE
Insoluble	*pgk*	A0A5S4TDF1	Phosphoglycerate kinase	A0A5S4TDF1_STRPY	FALSE	FALSE	AQGIEIGNSLVEEDK	686.1419	686.1419	3	AQGIEIGNSLVEED[HAyneD	42492	1	TRUE
Insoluble	*pgk*	A0A5S4TDF1	Phosphoglycerate kinase	A0A5S4TDF1_STRPY	FALSE	FALSE	AQGIEIGNSLVEEDK	685.79645	685.79645	3	AQGIEIGNSLVEED[HAyneD	42483	1	TRUE
Insoluble	*pgk*	A0A5S4TDF1	Phosphoglycerate kinase	A0A5S4TDF1_STRPY	FALSE	FALSE	AQGIEIGNSLVEEDK	520.9774	520.9774	3	AQGIEIGNSLVEED[HAyneD	43470	2	TRUE
Insoluble	*pgk*	A0A5S4TDF1	Phosphoglycerate kinase	A0A5S4TDF1_STRPY	TRUE	TRUE	DTEGEAVSEGFLGLDIGPK	488.64923	488.64923	3	D[HAyneDE]TEGEAVSEGFL	63139	1	TRUE
Insoluble	*pgk*	A0A5S4TDF1	Phosphoglycerate kinase	A0A5S4TDF1_STRPY	TRUE	TRUE	LEEAINALEDGQVLLVENTR	340.16156	340.16156	3	LEEAINALED[HAyneDE]GQ	67004	1	TRUE
Insoluble	*pgk*	A0A5S4TDF1	Phosphoglycerate kinase	A0A5S4TDF1_STRPY	TRUE	TRUE	LEEAINALEDGQVLLVENTR	259.82843	259.82843	4	LEEAINALED[HAyneDE]GQ	64912	1	TRUE
Insoluble	*pgk*	A0A5S4TDF1	Phosphoglycerate kinase	A0A5S4TDF1_STRPY	TRUE	TRUE	LEEAINALEDGQVLLVENTR	259.84222	259.84222	4	LEEAINALED[HAyneDE]GQ	64898	1	TRUE
Insoluble	*pgk*	A0A5S4TDF1	Phosphoglycerate kinase	A0A5S4TDF1_STRPY	FALSE	FALSE	FEDVDGKK	611.2303	611.2303	3	FED[HAyneDE]VDGKK	13520	1	TRUE
Insoluble	*phnA*	A0A4V6YVP2	Alkylphosphonate utilization protein	A0A4V6YVP2_STRPY	TRUE	TRUE	LSDGDTITVVK	1467.9386	1467.9386	2	LSD[HAyneDE]GDTITVVK	31570	1	TRUE
Insoluble	*phnA*	A0A4V6YVP2	Alkylphosphonate utilization protein	A0A4V6YVP2_STRPY	TRUE	TRUE	LSDGDTITVVK	217.43298	217.43298	3	LSD[HAyneDE]GDTITVVK	31578	1	TRUE
Soluble	*phnA*	A0A4V6YVP2	Alkylphosphonate utilization protein	A0A4V6YVP2_STRPY	TRUE	TRUE	LSDGDTITVVK	1967.0189	1967.0189	2	LSD[HAyneDE]GDTITVVK	27846	1	TRUE
Soluble	*phnA*	A0A4V6YVP2	Alkylphosphonate utilization protein	A0A4V6YVP2_STRPY	TRUE	TRUE	LSDGDTITVVK	436.0277	436.0277	3	LSD[HAyneDE]GDTITVVK	27907	1	TRUE
Soluble	*phnA*	A0A4V6YVP2	Alkylphosphonate utilization protein	A0A4V6YVP2_STRPY	TRUE	TRUE	LSDGDTITVVK	436.06177	436.06177	3	LSD[HAyneDE]GDTITVVK	27900	1	TRUE
Soluble	*phnA*	A0A4V6YVP2	Alkylphosphonate utilization protein	A0A4V6YVP2_STRPY	TRUE	TRUE	LSDGDTITVVK	229.24312	229.24312	3	LSD[HAyneDE]GDTITVVK	26729	1	TRUE
Soluble	*phnA*	A0A4V6YVP2	Alkylphosphonate utilization protein	A0A4V6YVP2_STRPY	TRUE	TRUE	LSDGDTITVVK	436.05225	436.05225	3	LSDGD[HAyneDE]TITVVK	28371	1	TRUE
Soluble	*phoU*	A0A4U7INT0	Phosphate-specific transport system acc	A0A4U7INT0_STRPY	TRUE	TRUE	AYEIAASDEIIDQYFR	412.4731	412.4731	3	AYEIAASDEIID[HAyneDE]Q	59837	1	TRUE
Soluble	*phoU*	A0A4U7INT0	Phosphate-specific transport system acc	A0A4U7INT0_STRPY	TRUE	TRUE	EVIEEDDTINNFETK	417.43274	417.43274	3	EVIEEDD[HAyneDE]TINNFI	34971	3	TRUE
Soluble	*phoU*	A0A4U7INT0	Phosphate-specific transport system acc	A0A4U7INT0_STRPY	TRUE	TRUE	MGDHAASIAK	390.84515	390.84515	3	M[Oxidation (M)]GD[HAyne	7770	1	TRUE
Soluble	*phoU*	A0A4U7INT0	Phosphate-specific transport system acc	A0A4U7INT0_STRPY	TRUE	TRUE	MGDHAASIAK	390.84515	390.84515	3	M[Oxidation (M)]GD[HAyne	7780	1	TRUE
Soluble	*ppaC*	A0A5S4TGD7	Probable manganese-dependent inorgan	A0A5S4TGD7_STRPY	TRUE	TRUE	EVEVYGVVDHHR	559.9977	559.9977	3	EVEVYGVVD[HAyneDE]HHI	20262	2	TRUE
Soluble	*ppaC*	A0A5S4TGD7	Probable manganese-dependent inorgan	A0A5S4TGD7_STRPY	TRUE	TRUE	EVEVYGVVDHHR	469.82355	469.82355	3	EVEVYGVVD[HAyneDE]HHI	18520	1	TRUE
Soluble	*ppaC*	A0A5S4TGD7	Probable manganese-dependent inorgan	A0A5S4TGD7_STRPY	TRUE	TRUE	EVEVYGVVDHHR	469.9344	469.9344	3	EVEVYGVVD[HAyneDE]HHI	18531	1	TRUE
Soluble	*ppaC*	A0A5S4TGD7	Probable manganese-dependent inorgan	A0A5S4TGD7_STRPY	TRUE	TRUE	EVEVYGVVDHHR	561.1808	561.1808	3	EVEVYGVVD[HAyneDE]HHI	20436	2	FALSE
Soluble	*ppaC*	A0A5S4TGD7	Probable manganese-dependent inorgan	A0A5S4TGD7_STRPY	TRUE	TRUE	EVEVYGVVDHHR	469.76114	469.76114	3	EVEVYGVVD[HAyneDE]HHI	18698	2	FALSE
Soluble	*ppaC*	A0A5S4TGD7	Probable manganese-dependent inorgan	A0A5S4TGD7_STRPY	TRUE	TRUE	EVEVYGVVDHHR	372.63278	372.63278	4	EVEVYGVVD[HAyneDE]HHI	18584	1	TRUE
Soluble	*ppaC*	A0A5S4TGD7	Probable manganese-dependent inorgan	A0A5S4TGD7_STRPY	TRUE	TRUE	EVEVYGVVDHHR	392.18732	392.18732	4	EVEVYGVVD[HAyneDE]HHI	20294	1	TRUE
Soluble	*ppaC*	A0A5S4TGD7	Probable manganese-dependent inorgan	A0A5S4TGD7_STRPY	TRUE	TRUE	AQGSEQVILTDHNEFQQSIADIR	793.61536	793.61536	4	AQGSEQVILTD[HAyneDE]H	40076	1	TRUE
Soluble	*ppaC*	A0A5S4TGD7	Probable manganese-dependent inorgan	A0A5S4TGD7_STRPY	TRUE	TRUE	AQGSEQVILTDHNEFQQSIADIR	793.6163	793.6163	4	AQGSEQVILTD[HAyneDE]H	41153	2	TRUE
Soluble	*ppaC*	A0A5S4TGD7	Probable manganese-dependent inorgan	A0A5S4TGD7_STRPY	TRUE	TRUE	AQGSEQVILTDHNEFQQSIADIR	417.8759	417.8759	3	AQGSEQVILTD[HAyneDE]H	40090	1	TRUE
Insoluble	*proS*	A0A5S4TLV2	Proline--tRNA ligase	A0A5S4TLV2_STRPY	TRUE	TRUE	DSQEFMAITPAR	333.16498	333.16498	3	D[HAyneDE]SQEFM[Oxidat	30904	2	TRUE
Insoluble	*prsA*	A0A5S4TEX0	Foldase protein PrsA	A0A5S4TEX0_STRPY	TRUE	TRUE	GDTISVSDFYNETK	1505.3391	1505.3391	3	GD[HAyneDE]TISVSDFYNE	46400	1	TRUE
Insoluble	*prsA*	A0A5S4TEX0	Foldase protein PrsA	A0A5S4TEX0_STRPY	TRUE	TRUE	GDTISVSDFYNETK	768.64954	768.64954	2	GD[HAyneDE]TISVSDFYNE	46436	1	TRUE
Insoluble	*prsA*	A0A5S4TEX0	Foldase protein PrsA	A0A5S4TEX0_STRPY	TRUE	TRUE	FDSGATNVPTDVVK	622.24194	622.24194	3	FD[HAyneDE]SGATNVPTDV	33294	1	TRUE
Insoluble	*prsA*	A0A5S4TEX0	Foldase protein PrsA	A0A5S4TEX0_STRPY	TRUE	TRUE	FDSGATNVPTDVVK	243.89018	243.89018	2	FD[HAyneDE]SGATNVPTDV	33289	1	TRUE
Insoluble	*prsA*	A0A5S4TEX0	Foldase protein PrsA	A0A5S4TEX0_STRPY	TRUE	TRUE	FDSGATNVPTDVVK	255.12625	255.12625	2	FD[HAyneDE]SGATNVPTDV	33307	1	TRUE
Insoluble	*prsA*	A0A5S4TLN5	Foldase protein PrsA	A0A5S4TLN5_STRPY	TRUE	TRUE	GDTITVSDFYNETK	314.58185	314.58185	3	GD[HAyneDE]TITVSDFYNET	45848	1	TRUE
Insoluble	*prsA*	A0A5S4TLN5	Foldase protein PrsA	A0A5S4TLN5_STRPY	TRUE	TRUE	GDTITVSDFYNETK	166.97073	166.97073	2	GD[HAyneDE]TITVSDFYNET	45834	1	TRUE
Insoluble	*prsA*	A0A5S4TLN5	Foldase protein PrsA	A0A5S4TLN5_STRPY	TRUE	TRUE	GDTITVSDFYNETK	197.74915	197.74915	2	GD[HAyneDE]TITVSDFYNET	45828	1	TRUE
Soluble	*prsA*	A0A5S4TEX0	Foldase protein PrsA	A0A5S4TEX0_STRPY	TRUE	TRUE	GDTISVSDFYNETK	661.08923	661.08923	3	GD[HAyneDE]TISVSDFYNE	42344	1	TRUE
Soluble	*prsA*	A0A5S4TEX0	Foldase protein PrsA	A0A5S4TEX0_STRPY	TRUE	TRUE	GDTISVSDFYNETK	661.0935	661.0935	3	GD[HAyneDE]TISVSDFYNE	42535	1	TRUE
Soluble	*prsA*	A0A5S4TEX0	Foldase protein PrsA	A0A5S4TEX0_STRPY	TRUE	TRUE	GDTISVSDFYNETK	661.0889	661.0889	3	GD[HAyneDE]TISVSDFYNE	42356	1	TRUE
Soluble	*prsA*	A0A5S4TEX0	Foldase protein PrsA	A0A5S4TEX0_STRPY	TRUE	TRUE	GDTISVSDFYNETK	504.27573	504.27573	2	GD[HAyneDE]TISVSDFYNE	42363	1	TRUE
Soluble	*prsA*	A0A5S4TEX0	Foldase protein PrsA	A0A5S4TEX0_STRPY	TRUE	TRUE	GDTISVSDFYNETK	504.27573	504.27573	2	GD[HAyneDE]TISVSDFYNE	42378	1	TRUE
Soluble	*prsA*	A0A5S4TEX0	Foldase protein PrsA	A0A5S4TEX0_STRPY	TRUE	TRUE	FDSGATNVPTDVVK	487.34088	487.34088	3	FD[HAyneDE]SGATNVPTDV	29520	1	TRUE
Soluble	*prsA*	A0A5S4TEX0	Foldase protein PrsA	A0A5S4TEX0_STRPY	TRUE	TRUE	FDSGATNVPTDVVK	479.4807	479.4807	3	FD[HAyneDE]SGATNVPTDV	29510	1	TRUE
Soluble	*prsA*	A0A5S4TEX0	Foldase protein PrsA	A0A5S4TEX0_STRPY	TRUE	TRUE	FDSGATNVPTDVVK	202.22649	202.22649	2	FD[HAyneDE]SGATNVPTDV	29586	1	TRUE
Insoluble	*prsA.2*	A0A4U7FYU5	Ribose-phosphate pyrophosphokinase	A0A4U7FYU5_STRPY	TRUE	TRUE	LIVLDTIYLPK	254.57353	254.57353	2	LIVLD[HAyneDE]TIYLPK	64996	1	TRUE
Insoluble	*ptsH*	A0A4U9T4B3	Phosphocarrier protein HPr	A0A4U9T4B3_STRPY	TRUE	TRUE	DFHIVAETGIHAR	1362.5403	1362.5403	4	D[HAyneDE]FHIVAETGIHAR	23641	1	TRUE
Insoluble	*ptsH*	A0A4U9T4B3	Phosphocarrier protein HPr	A0A4U9T4B3_STRPY	TRUE	TRUE	DFHIVAETGIHAR	265.97205	265.97205	4	D[HAyneDE]FHIVAETGIHAR	27147	1	TRUE
Insoluble	*ptsH*	A0A4U9T4B3	Phosphocarrier protein HPr	A0A4U9T4B3_STRPY	TRUE	TRUE	DFHIVAETGIHAR	724.23865	724.23865	3	D[HAyneDE]FHIVAETGIHAR	23704	1	TRUE
Insoluble	*ptsH*	A0A4U9T4B3	Phosphocarrier protein HPr	A0A4U9T4B3_STRPY	TRUE	TRUE	DFHIVAETGIHAR	724.23865	724.23865	3	D[HAyneDE]FHIVAETGIHAR	23719	1	TRUE
Insoluble	*ptsH*	A0A4U9T4B3	Phosphocarrier protein HPr	A0A4U9T4B3_STRPY	TRUE	TRUE	DFHIVAETGIHAR	266.2062	266.2062	3	D[HAyneDE]FHIVAETGIHAR	24319	3	TRUE
Insoluble	*ptsH*	A0A4U9T4B3	Phosphocarrier protein HPr	A0A4U9T4B3_STRPY	TRUE	TRUE	FASDITLDYK	557.71826	557.71826	2	FASD[HAyneDE]ITLDYK	42471	1	TRUE
Insoluble	*ptsH*	A0A4U9T4B3	Phosphocarrier protein HPr	A0A4U9T4B3_STRPY	TRUE	TRUE	SIMGVMSLGVGQGADVTISAEGADAEDA	651.8313	651.8313	4	SIM[Oxidation (M)]GVM[O:	72880	1	TRUE
Insoluble	*ptsH*	A0A4U9T4B3	Phosphocarrier protein HPr	A0A4U9T4B3_STRPY	TRUE	TRUE	SIMGVMSLGVGQGADVTISAEGADAEDA	524.22736	524.22736	4	SIM[Oxidation (M)]GVM[O:	73021	1	TRUE
Soluble	*ptsH*	A0A4U9T4B3	Phosphocarrier protein HPr	A0A4U9T4B3_STRPY	TRUE	TRUE	DFHIVAETGIHAR	32202.17	32202.17	4	D[HAyneDE]FHIVAETGIHAR	24836	1	TRUE
Soluble	*ptsH*	A0A4U9T4B3	Phosphocarrier protein HPr	A0A4U9T4B3_STRPY	TRUE	TRUE	DFHIVAETGIHAR	6313.5127	6313.5127	4	D[HAyneDE]FHIVAETGIHAR	21620	2	TRUE
Soluble	*ptsH*	A0A4U9T4B3	Phosphocarrier protein HPr	A0A4U9T4B3_STRPY	TRUE	TRUE	DFHIVAETGIHAR	6306.0005	6306.0005	4	D[HAyneDE]FHIVAETGIHAR	20970	2	TRUE
Soluble	*ptsH*	A0A4U9T4B3	Phosphocarrier protein HPr	A0A4U9T4B3_STRPY	TRUE	TRUE	DFHIVAETGIHAR	577.3078	577.3078	4	D[HAyneDE]FHIVAETGIHAR	23143	1	FALSE
Soluble	*ptsH*	A0A4U9T4B3	Phosphocarrier protein HPr	A0A4U9T4B3_STRPY	TRUE	TRUE	DFHIVAETGIHAR	2254.4758	2254.4758	4	D[HAyneDE]FHIVAETGIHAR	22421	1	FALSE
Soluble	*ptsH*	A0A4U9T4B3	Phosphocarrier protein HPr	A0A4U9T4B3_STRPY	TRUE	TRUE	DFHIVAETGIHAR	699.2163	699.2163	4	D[HAyneDE]FHIVAETGIHAR	23777	1	FALSE
Soluble	*ptsH*	A0A4U9T4B3	Phosphocarrier protein HPr	A0A4U9T4B3_STRPY	TRUE	TRUE	DFHIVAETGIHAR	27204.562	27204.562	4	D[HAyneDE]FHIVAETGIHAR	24730	1	FALSE
Soluble	*ptsH*	A0A4U9T4B3	Phosphocarrier protein HPr	A0A4U9T4B3_STRPY	TRUE	TRUE	DFHIVAETGIHAR	638.46936	638.46936	4	D[HAyneDE]FHIVAETGIHAR	23125	1	FALSE
Soluble	*ptsH*	A0A4U9T4B3	Phosphocarrier protein HPr	A0A4U9T4B3_STRPY	TRUE	TRUE	DFHIVAETGIHAR	6308.0186	6308.0186	4	D[HAyneDE]FHIVAETGIHAR	20998	1	FALSE
Soluble	*ptsH*	A0A4U9T4B3	Phosphocarrier protein HPr	A0A4U9T4B3_STRPY	TRUE	TRUE	DFHIVAETGIHAR	4228.5522	4228.5522	4	D[HAyneDE]FHIVAETGIHAR	21986	1	FALSE
Soluble	*ptsH*	A0A4U9T4B3	Phosphocarrier protein HPr	A0A4U9T4B3_STRPY	TRUE	TRUE	DFHIVAETGIHAR	6305.9907	6305.9907	4	D[HAyneDE]FHIVAETGIHAR	21602	2	FALSE
Soluble	*ptsH*	A0A4U9T4B3	Phosphocarrier protein HPr	A0A4U9T4B3_STRPY	TRUE	TRUE	DFHIVAETGIHAR	30650.467	30650.467	4	D[HAyneDE]FHIVAETGIHAR	24805	1	FALSE
Soluble	*ptsH*	A0A4U9T4B3	Phosphocarrier protein HPr	A0A4U9T4B3_STRPY	TRUE	TRUE	DFHIVAETGIHAR	6312.0723	6312.0723	4	D[HAyneDE]FHIVAETGIHAR	21653	2	FALSE
Soluble	*ptsH*	A0A4U9T4B3	Phosphocarrier protein HPr	A0A4U9T4B3_STRPY	TRUE	TRUE	DFHIVAETGIHAR	6314.047	6314.047	4	D[HAyneDE]FHIVAETGIHAR	20832	1	FALSE
Soluble	*ptsH*	A0A4U9T4B3	Phosphocarrier protein HPr	A0A4U9T4B3_STRPY	TRUE	TRUE	DFHIVAETGIHAR	6306.326	6306.326	4	D[HAyneDE]FHIVAETGIHAR	20900	1	FALSE
Soluble	*ptsH*	A0A4U9T4B3	Phosphocarrier protein HPr	A0A4U9T4B3_STRPY	TRUE	TRUE	DFHIVAETGIHAR	6306.0044	6306.0044	4	D[HAyneDE]FHIVAETGIHAR	21326	1	FALSE
Soluble	*ptsH*	A0A4U9T4B3	Phosphocarrier protein HPr	A0A4U9T4B3_STRPY	TRUE	TRUE	DFHIVAETGIHAR	6308.009	6308.009	4	D[HAyneDE]FHIVAETGIHAR	21178	1	FALSE
Soluble	*ptsH*	A0A4U9T4B3	Phosphocarrier protein HPr	A0A4U9T4B3_STRPY	TRUE	TRUE	DFHIVAETGIHAR	6306.071	6306.071	4	D[HAyneDE]FHIVAETGIHAR	21170	1	FALSE
Soluble	*ptsH*	A0A4U9T4B3	Phosphocarrier protein HPr	A0A4U9T4B3_STRPY	TRUE	TRUE	DFHIVAETGIHAR	5862.849	5862.849	4	D[HAyneDE]FHIVAETGIHAR	20932	1	FALSE
Soluble	*ptsH*	A0A4U9T4B3	Phosphocarrier protein HPr	A0A4U9T4B3_STRPY	TRUE	TRUE	DFHIVAETGIHAR	183.6583	183.6583	4	D[HAyneDE]FHIVAETGIHAR	24671	1	FALSE
Soluble	*ptsH*	A0A4U9T4B3	Phosphocarrier protein HPr	A0A4U9T4B3_STRPY	TRUE	TRUE	DFHIVAETGIHAR	6314.1426	6314.1426	4	D[HAyneDE]FHIVAETGIHAR	21669	1	FALSE
Soluble	*ptsH*	A0A4U9T4B3	Phosphocarrier protein HPr	A0A4U9T4B3_STRPY	TRUE	TRUE	DFHIVAETGIHAR	6306.045	6306.045	4	D[HAyneDE]FHIVAETGIHAR	21089	1	FALSE
Soluble	*ptsH*	A0A4U9T4B3	Phosphocarrier protein HPr	A0A4U9T4B3_STRPY	TRUE	TRUE	DFHIVAETGIHAR	6308.0083	6308.0083	4	D[HAyneDE]FHIVAETGIHAR	21101	1	FALSE
Soluble	*ptsH*	A0A4U9T4B3	Phosphocarrier protein HPr	A0A4U9T4B3_STRPY	TRUE	TRUE	DFHIVAETGIHAR	6306.045	6306.045	4	D[HAyneDE]FHIVAETGIHAR	21074	1	FALSE
Soluble	*ptsH*	A0A4U9T4B3	Phosphocarrier protein HPr	A0A4U9T4B3_STRPY	TRUE	TRUE	DFHIVAETGIHAR	33654.293	33654.293	4	D[HAyneDE]FHIVAETGIHAR	24860	3	FALSE
Soluble	*ptsH*	A0A4U9T4B3	Phosphocarrier protein HPr	A0A4U9T4B3_STRPY	TRUE	TRUE	DFHIVAETGIHAR	1844.6176	1844.6176	4	D[HAyneDE]FHIVAETGIHAR	32530	2	FALSE
Soluble	*ptsH*	A0A4U9T4B3	Phosphocarrier protein HPr	A0A4U9T4B3_STRPY	TRUE	TRUE	DFHIVAETGIHAR	1375.888	1375.888	3	D[HAyneDE]FHIVAETGIHAR	22452	1	TRUE
Soluble	*ptsH*	A0A4U9T4B3	Phosphocarrier protein HPr	A0A4U9T4B3_STRPY	TRUE	TRUE	DFHIVAETGIHAR	450.13623	450.13623	3	D[HAyneDE]FHIVAETGIHAR	23208	1	TRUE
Soluble	*ptsH*	A0A4U9T4B3	Phosphocarrier protein HPr	A0A4U9T4B3_STRPY	TRUE	TRUE	DFHIVAETGIHAR	3516.3623	3516.3623	3	D[HAyneDE]FHIVAETGIHAR	20875	1	TRUE
Soluble	*ptsH*	A0A4U9T4B3	Phosphocarrier protein HPr	A0A4U9T4B3_STRPY	TRUE	TRUE	DFHIVAETGIHAR	643.7676	643.7676	3	D[HAyneDE]FHIVAETGIHAR	23157	1	FALSE
Soluble	*ptsH*	A0A4U9T4B3	Phosphocarrier protein HPr	A0A4U9T4B3_STRPY	TRUE	TRUE	DFHIVAETGIHAR	3697.0913	3697.0913	3	D[HAyneDE]FHIVAETGIHAR	20837	1	FALSE
Soluble	*ptsH*	A0A4U9T4B3	Phosphocarrier protein HPr	A0A4U9T4B3_STRPY	TRUE	TRUE	DFHIVAETGIHAR	3437.8662	3437.8662	3	D[HAyneDE]FHIVAETGIHAR	20931	1	FALSE
Soluble	*ptsH*	A0A4U9T4B3	Phosphocarrier protein HPr	A0A4U9T4B3_STRPY	TRUE	TRUE	DFHIVAETGIHAR	1713.6915	1713.6915	3	D[HAyneDE]FHIVAETGIHAR	22265	1	FALSE
Soluble	*ptsH*	A0A4U9T4B3	Phosphocarrier protein HPr	A0A4U9T4B3_STRPY	TRUE	TRUE	DFHIVAETGIHAR	23313.25	23313.25	3	D[HAyneDE]FHIVAETGIHAR	26533	1	FALSE
Soluble	*ptsH*	A0A4U9T4B3	Phosphocarrier protein HPr	A0A4U9T4B3_STRPY	TRUE	TRUE	DFHIVAETGIHAR	3452.3208	3452.3208	3	D[HAyneDE]FHIVAETGIHAR	21703	1	FALSE
Soluble	*ptsH*	A0A4U9T4B3	Phosphocarrier protein HPr	A0A4U9T4B3_STRPY	TRUE	TRUE	DFHIVAETGIHAR	353.68118	353.68118	3	D[HAyneDE]FHIVAETGIHAR	23735	1	FALSE
Soluble	*ptsH*	A0A4U9T4B3	Phosphocarrier protein HPr	A0A4U9T4B3_STRPY	TRUE	TRUE	DFHIVAETGIHAR	466.98932	466.98932	3	D[HAyneDE]FHIVAETGIHAR	23643	1	FALSE
Soluble	*ptsH*	A0A4U9T4B3	Phosphocarrier protein HPr	A0A4U9T4B3_STRPY	TRUE	TRUE	DFHIVAETGIHAR	498.55667	498.55667	3	D[HAyneDE]FHIVAETGIHAR	23649	1	FALSE
Soluble	*ptsH*	A0A4U9T4B3	Phosphocarrier protein HPr	A0A4U9T4B3_STRPY	TRUE	TRUE	DFHIVAETGIHAR	318.5589	318.5589	3	D[HAyneDE]FHIVAETGIHAR	23768	3	FALSE
Soluble	*ptsH*	A0A4U9T4B3	Phosphocarrier protein HPr	A0A4U9T4B3_STRPY	TRUE	TRUE	DFHIVAETGIHAR	851.6734	851.6734	5	D[HAyneDE]FHIVAETGIHAR	20901	1	TRUE
Soluble	*ptsH*	A0A4U9T4B3	Phosphocarrier protein HPr	A0A4U9T4B3_STRPY	TRUE	TRUE	FASDITLDYK	974.2417	974.2417	2	FASD[HAyneDE]ITLDYK	38360	1	TRUE
Soluble	*ptsH*	A0A4U9T4B3	Phosphocarrier protein HPr	A0A4U9T4B3_STRPY	TRUE	TRUE	FASDITLDYK	977.7055	977.7055	2	FASD[HAyneDE]ITLDYK	38221	1	TRUE
Soluble	*ptsH*	A0A4U9T4B3	Phosphocarrier protein HPr	A0A4U9T4B3_STRPY	TRUE	TRUE	FASDITLDYK	904.9407	904.9407	2	FASDITLD[HAyneDE]YK	36992	1	TRUE
Soluble	*ptsH*	A0A4U9T4B3	Phosphocarrier protein HPr	A0A4U9T4B3_STRPY	TRUE	TRUE	FASDITLDYK	713.9877	713.9877	3	FASDITLD[HAyneDE]YK	37014	1	TRUE
Soluble	*ptsH*	A0A4U9T4B3	Phosphocarrier protein HPr	A0A4U9T4B3_STRPY	TRUE	TRUE	FASDITLDYK	707.4782	707.4782	3	FASD[HAyneDE]ITLDYK	38234	1	TRUE
Soluble	*ptsH*	A0A4U9T4B3	Phosphocarrier protein HPr	A0A4U9T4B3_STRPY	TRUE	TRUE	ASKDFHIVAETGIHAR	650.4989	650.4989	4	[Acetyl (Protein N-term)]AS	20284	1	TRUE
Soluble	*ptsH*	A0A4U9T4B3	Phosphocarrier protein HPr	A0A4U9T4B3_STRPY	TRUE	TRUE	ASKDFHIVAETGIHAR	650.4989	650.4989	4	[Acetyl (Protein N-term)]AS	20271	1	TRUE
Soluble	*ptsH*	A0A4U9T4B3	Phosphocarrier protein HPr	A0A4U9T4B3_STRPY	TRUE	TRUE	ASKDFHIVAETGIHAR	317.69495	317.69495	5	[Acetyl (Protein N-term)]AS	20329	1	TRUE
Soluble	*ptsH*	A0A4U9T4B3	Phosphocarrier protein HPr	A0A4U9T4B3_STRPY	TRUE	TRUE	ASKDFHIVAETGIHAR	316.17706	316.17706	5	[Acetyl (Protein N-term)]AS	20337	1	TRUE

Soluble	ptsH	A0A4U9T4B3	Phosphocarrier protein HPr	A0A4U9T4B3_STRPY	TRUE	TRUE	SIMGVMSLGVGQGADVTISAEGADAEDA	1425.3364	1425.3364	4	SIM[Oxidation (M)]GVM[O	68401	2	TRUE
Insoluble	ptsP	A0A5S4TCC1	Phosphoenolpyruvate-protein phosphotr	A0A5S4TCC1_STRPY	TRUE	TRUE	LDVALQAAQDELSVIR	426.86548	426.86548	3	LD[HAyneDE]VALQAAQDE	67819	1	TRUE
Insoluble	ptsP	A0A5S4TCC1	Phosphoenolpyruvate-protein phosphotr	A0A5S4TCC1_STRPY	TRUE	TRUE	LDVALQAAQDELSVIR	371.5388	371.5388	3	LD[HAyneDE]VALQAAQDE	67858	1	TRUE
Insoluble	ptsP	A0A5S4TCC1	Phosphoenolpyruvate-protein phosphotr	A0A5S4TCC1_STRPY	TRUE	TRUE	LDVALQAAQDELSVIR	426.92957	426.92957	3	LD[HAyneDE]VALQAAQDE	67868	1	TRUE
Insoluble	ptsP	A0A5S4TCC1	Phosphoenolpyruvate-protein phosphotr	A0A5S4TCC1_STRPY	TRUE	TRUE	AYLLVQPDLSFETVTVADTNAEEAR	409.57883	409.57883	4	AYLLVQPD[HAyneDE]LSFE	67984	2	TRUE
Insoluble	pyk	A0A4U7H2M5	Pyruvate kinase	A0A4U7H2M5_STRPY	TRUE	TRUE	ETGNDHVQLFAK	1740.1442	1740.1442	3	ETGND[HAyneDE]HVQLFAI	27209	1	TRUE
Insoluble	pyk	A0A4U7H2M5	Pyruvate kinase	A0A4U7H2M5_STRPY	TRUE	TRUE	ETGNDHVQLFAK	1722.5636	1722.5636	3	ETGND[HAyneDE]HVQLFAI	27250	1	TRUE
Insoluble	pyk	A0A4U7H2M5	Pyruvate kinase	A0A4U7H2M5_STRPY	TRUE	TRUE	ETGNDHVQLFAK	1722.5745	1722.5745	3	ETGND[HAyneDE]HVQLFAI	27963	1	TRUE
Insoluble	pyk	A0A4U7H2M5	Pyruvate kinase	A0A4U7H2M5_STRPY	TRUE	TRUE	GDMGIEVPFEMVPVFQK	1587.873	1587.873	3	GD[HAyneDE]M[Oxidation	62576	1	TRUE
Insoluble	pyk	A0A4U7H2M5	Pyruvate kinase	A0A4U7H2M5_STRPY	TRUE	TRUE	GDMGIEVPFEMVPVFQK	1578.9696	1578.9696	3	GD[HAyneDE]M[Oxidation	62588	1	TRUE
Insoluble	pyk	A0A4U7H2M5	Pyruvate kinase	A0A4U7H2M5_STRPY	TRUE	TRUE	IENQQGIDNLDEIIEAADGIMIAR	258.1773	258.1773	3	IENQQGIDNLD[HAyneDE]I	76473	1	TRUE
Insoluble	pyk	A0A4U7H2M5	Pyruvate kinase	A0A4U7H2M5_STRPY	TRUE	TRUE	TDVIASAVK	412.42474	412.42474	2	TD[HAyneDE]VIASAVK	34762	1	TRUE
Insoluble	pyk	A0A4U7H2M5	Pyruvate kinase	A0A4U7H2M5_STRPY	TRUE	TRUE	TDVIASAVK	3095.1663	3095.1663	2	TD[HAyneDE]VIASAVK	34770	1	TRUE
Insoluble	pyk	A0A4U7H2M5	Pyruvate kinase	A0A4U7H2M5_STRPY	TRUE	TRUE	FRPDADILAVTFDEK	462.90054	462.90054	3	FRPDADILAVTFD[HAyneDE	56849	1	TRUE
Insoluble	pyk	A0A4U7H2M5	Pyruvate kinase	A0A4U7H2M5_STRPY	TRUE	TRUE	FRPDADILAVTFDEK	208.19385	208.19385	3	FRPD[HAyneDE]ADILAVTFI	51522	1	TRUE
Insoluble	pyk	A0A4U7H2M5	Pyruvate kinase	A0A4U7H2M5_STRPY	TRUE	TRUE	VAVEAGLVOSGDNIVIVAGVPVGTGGTNT	1028.6392	1028.6392	4	VAVEAGLVOSGD[HAyneDE	60167	2	TRUE
Insoluble	pyk	A0A4U7H2M5	Pyruvate kinase	A0A4U7H2M5_STRPY	TRUE	TRUE	VAVEAGLVOSGDNIVIVAGVPVGTGGTNT	1039.9619	1039.9619	4	VAVEAGLVOSGD[HAyneDE	60181	1	TRUE
Insoluble	pyk	A0A4U7H2M5	Pyruvate kinase	A0A4U7H2M5_STRPY	TRUE	TRUE	VAVEAGLVOSGDNIVIVAGVPVGTGGTNT	1039.9619	1039.9619	4	VAVEAGLVOSGD[HAyneDE	60217	1	TRUE
Insoluble	pyk	A0A4U7H2M5	Pyruvate kinase	A0A4U7H2M5_STRPY	TRUE	TRUE	VAVEAGLVOSGDNIVIVAGVPVGTGGTNT	1028.6392	1028.6392	4	VAVEAGLVOSGD[HAyneDE	60635	1	FALSE
Insoluble	pyk	A0A4U7H2M5	Pyruvate kinase	A0A4U7H2M5_STRPY	TRUE	TRUE	LDSSAFPR	870.78284	870.78284	3	LD[HAyneDE]SSAFPR	27080	2	TRUE
Insoluble	pyk	A0A4U7H2M5	Pyruvate kinase	A0A4U7H2M5_STRPY	TRUE	TRUE	LDSSAFPR	872.9327	872.9327	3	LD[HAyneDE]SSAFPR	26842	1	TRUE
Insoluble	pyk	A0A4U7H2M5	Pyruvate kinase	A0A4U7H2M5_STRPY	TRUE	TRUE	LDSSAFPR	735.213	735.213	2	LD[HAyneDE]SSAFPR	26886	1	TRUE
Insoluble	pyk	A0A4U7H2M5	Pyruvate kinase	A0A4U7H2M5_STRPY	TRUE	TRUE	LDSSAFPR	735.2111	735.2111	2	LD[HAyneDE]SSAFPR	26764	1	TRUE
Insoluble	pyk	A0A4U7H2M5	Pyruvate kinase	A0A4U7H2M5_STRPY	TRUE	TRUE	LDSSAFPR	207.87057	207.87057	2	LD[HAyneDE]SSAFPR	28851	1	TRUE
Insoluble	pyk	A0A4U7H2M5	Pyruvate kinase	A0A4U7H2M5_STRPY	TRUE	TRUE	LDSSAFPR	760.9852	760.9852	2	LD[HAyneDE]SSAFPR	26894	1	FALSE
Insoluble	pyk	A0A4U7H2M5	Pyruvate kinase	A0A4U7H2M5_STRPY	TRUE	TRUE	LDSSAFPR	753.8738	753.8738	2	LD[HAyneDE]SSAFPR	26793	1	FALSE
Insoluble	pyk	A0A4U7H2M5	Pyruvate kinase	A0A4U7H2M5_STRPY	TRUE	TRUE	LDSSAFPR	754.3779	754.3779	2	LD[HAyneDE]SSAFPR	26866	1	FALSE
Insoluble	pyk	A0A4U7H2M5	Pyruvate kinase	A0A4U7H2M5_STRPY	TRUE	TRUE	LDSSAFPR	181.90201	181.90201	2	LD[HAyneDE]SSAFPR	28816	1	FALSE
Insoluble	pyk	A0A4U7H2M5	Pyruvate kinase	A0A4U7H2M5_STRPY	TRUE	TRUE	FNFSHGDHK	498.4336	498.4336	3	FNFSHGD[HAyneDE]HK	13061	3	TRUE
Insoluble	raiA	A0A5S4TF36	Ribosome hibernation promoting factor	A0A5S4TF36_STRPY	TRUE	TRUE	LQMELLGHDFFIYTDSEDGATNILYR	545.1383	545.1383	3	LQM[Oxidation (M)]ELLGH	64958	1	TRUE
Insoluble	recA	A0A5S4TE17	Protein RecA	A0A5S4TE17_STRPY	TRUE	TRUE	AEIDGDIGDSHVGLQAR	577.04004	577.04004	4	AEIDGDIGD[HAyneDE]SHV	27780	3	TRUE
Insoluble	recA	A0A5S4TE17	Protein RecA	A0A5S4TE17_STRPY	TRUE	TRUE	AEIDGDIGDSHVGLQAR	421.33215	421.33215	3	AEIDGDIGD[HAyneDE]SHV	28263	1	TRUE
Insoluble	recA	A0A5S4TE17	Protein RecA	A0A5S4TE17_STRPY	TRUE	TRUE	AEIDGDIGDSHVGLQAR	392.1231	392.1231	3	AEIDGDIGD[HAyneDE]SHV	28229	2	TRUE
Insoluble	recA	A0A5S4TE17	Protein RecA	A0A5S4TE17_STRPY	TRUE	TRUE	AEIDGDIGDSHVGLQAR	387.6062	387.6062	3	AEIDGDIGD[HAyneDE]SHV	27696	1	TRUE
Insoluble	rfbA	A0A5S4TG78	Glucose-1-phosphate thymidylyltransfera	A0A5S4TG78_STRPY	TRUE	TRUE	FGVVEFDENMNAISIEEKPEVPK	1295.4012	1295.4012	4	FGVVEFD[HAyneDE]ENM[	47217	1	TRUE
Insoluble	rfbB	A0A5S4TQ46	dTDP-glucose 4,6-dehydratase	A0A5S4TQ46_STRPY	TRUE	TRUE	DAYDHVTDR	341.65836	341.65836	3	D[HAyneDE]AYDHVTDR	16281	1	TRUE
Insoluble	rfbB	A0A5S4TQ46	dTDP-glucose 4,6-dehydratase	A0A5S4TQ46_STRPY	TRUE	TRUE	DAYDHVTDR	341.65836	341.65836	3	D[HAyneDE]AYDHVTDR	16265	1	TRUE
Insoluble	rfbB	A0A5S4TQ46	dTDP-glucose 4,6-dehydratase	A0A5S4TQ46_STRPY	TRUE	TRUE	YAIDSTK	260.0232	260.0232	2	YAID[HAyneDE]STK	20894	1	TRUE
Insoluble	rplA	A0A4U7IS47	Large ribosomal subunit protein uL1	A0A4U7IS47_STRPY	TRUE	TRUE	AAGADFVGEDDLVAK	553.5645	553.5645	3	AAGADFVGED[HAyneDE]D	42954	1	TRUE
Insoluble	rplA	A0A4U7IS47	Large ribosomal subunit protein uL1	A0A4U7IS47_STRPY	TRUE	TRUE	AAGADFVGEDDLVAK	529.00024	529.00024	3	AAGAD[HAyneDE]FVGEDD	41736	1	TRUE
Insoluble	rplA	A0A4U7IS47	Large ribosomal subunit protein uL1	A0A4U7IS47_STRPY	TRUE	TRUE	AAGADFVGEDDLVAK	529.06085	529.06085	3	AAGAD[HAyneDE]FVGEDD	41745	1	TRUE
Insoluble	rplA	A0A4U7IS47	Large ribosomal subunit protein uL1	A0A4U7IS47_STRPY	TRUE	TRUE	TGTVTMDVAK	435.76727	435.76727	3	TGTVTM[Oxidation (M)]D[H	16182	1	TRUE
Insoluble	rplA	A0A4U7IS47	Large ribosomal subunit protein uL1	A0A4U7IS47_STRPY	TRUE	TRUE	FDASVEVAYNLNIDVR	212.13078	212.13078	3	FD[HAyneDE]ASVEVAYNLN	63869	3	TRUE
Insoluble	rplB	A0A4U7HP27	Large ribosomal subunit protein uL2	A0A4U7HP27_STRPY	TRUE	TRUE	TIEYDPNR	242.16235	242.16235	2	TIEYD[HAyneDE]PNR	20429	1	TRUE
Insoluble	rplB	A0A4U7HP27	Large ribosomal subunit protein uL2	A0A4U7HP27_STRPY	TRUE	TRUE	NMTSLDFAEITTSTPEK	414.7567	414.7567	3	NM[Oxidation (M)]TSLD[HA	45883	1	TRUE
Insoluble	rplB	A0A4U7HP27	Large ribosomal subunit protein uL2	A0A4U7HP27_STRPY	TRUE	TRUE	IVSGPDADIK	699.261	699.261	3	IVSGPD[HAyneDE]ADIK	23797	1	TRUE
Insoluble	rplB	A0A4U7HP27	Large ribosomal subunit protein uL2	A0A4U7HP27_STRPY	TRUE	TRUE	IVSGPDADIK	681.5015	681.5015	3	IVSGPDAD[HAyneDE]IK	23016	1	TRUE
Insoluble	rplB	A0A4U7HP27	Large ribosomal subunit protein uL2	A0A4U7HP27_STRPY	TRUE	TRUE	IVSGPDADIK	686.374	686.374	3	IVSGPDAD[HAyneDE]IK	23126	3	TRUE
Insoluble	rplB	A0A4U7HP27	Large ribosomal subunit protein uL2	A0A4U7HP27_STRPY	TRUE	TRUE	IVSGPDADIK	375.5157	375.5157	2	IVSGPDAD[HAyneDE]IK	23033	1	TRUE
Insoluble	rplB	A0A4U7HP27	Large ribosomal subunit protein uL2	A0A4U7HP27_STRPY	TRUE	TRUE	IVSGPDADIK	380.8444	380.8444	2	IVSGPDAD[HAyneDE]IK	23098	1	TRUE
Insoluble	rplB	A0A4U7HP27	Large ribosomal subunit protein uL2	A0A4U7HP27_STRPY	TRUE	TRUE	NKDGVEAVVK	246.9362	246.9362	3	NKD[HAyneDE]GVEAVVK	17364	1	TRUE
Insoluble	rplD	A0A4Q1PW15	Large ribosomal subunit protein uL4	A0A4Q1PW15_STRPY	TRUE	TRUE	LFDQTGK	325.91	325.91	2	LFD[HAyneDE]QTGK	27369	1	TRUE
Insoluble	rplE	A0A4U7HK24	Large ribosomal subunit protein uL5	A0A4U7HK24_STRPY	TRUE	TRUE	EQLIFPEISFDDVDK	1128.0258	1128.0258	2	EQLIFPEISFD[HAyneDE]DV	69658	1	TRUE
Insoluble	rplE	A0A4U7HK24	Large ribosomal subunit protein uL5	A0A4U7HK24_STRPY	TRUE	TRUE	EQLIFPEISFDDVDK	1146.4401	1146.4401	2	EQLIFPEISFD[HAyneDE]DV	69772	1	TRUE
Insoluble	rplE	A0A4U7HK24	Large ribosomal subunit protein uL5	A0A4U7HK24_STRPY	TRUE	TRUE	IVLNMGVGDAVSNAK	689.29626	689.29626	3	IVLNM[Oxidation (M)]GVG	31439	1	TRUE
Insoluble	rplE	A0A4U7HK24	Large ribosomal subunit protein uL5	A0A4U7HK24_STRPY	TRUE	TRUE	MYEFLDK	710.9542	710.9542	2	M[Oxidation (M)]YEFLD[HA	36350	1	TRUE
Insoluble	rplE	A0A4U7HK24	Large ribosomal subunit protein uL5	A0A4U7HK24_STRPY	TRUE	TRUE	MYEFLDK	551.13696	551.13696	3	M[Oxidation (M)]YEFLD[HA	36399	1	TRUE
Insoluble	rplJ	A0A4Q1QWJ0	Large ribosomal subunit protein uL10	A0A4Q1QWJ0_STRPY	TRUE	TRUE	GLTVDQDTVLR	185.28955	185.28955	2	GLTVD[HAyneDE]QDTVLR	36735	1	TRUE
Insoluble	rplJ	A0A4Q1QWJ0	Large ribosomal subunit protein uL10	A0A4Q1QWJ0_STRPY	TRUE	TRUE	DVFVGPSAVAFSNEDVIAPAK	292.1021	292.1021	2	D[HAyneDE]VFVGPSAVAFS	59945	1	TRUE
Insoluble	rplJ	A0A4Q1QWJ0	Large ribosomal subunit protein uL10	A0A4Q1QWJ0_STRPY	TRUE	TRUE	DVFVGPSAVAFSNEDVIAPAK	288.79404	288.79404	3	D[HAyneDE]VFVGPSAVAFS	59832	1	TRUE
Insoluble	rplJ	A0A4Q1QWJ0	Large ribosomal subunit protein uL10	A0A4Q1QWJ0_STRPY	TRUE	TRUE	DVFVGPSAVAFSNEDVIAPAK	249.75755	249.75755	3	D[HAyneDE]VFVGPSAVAFS	59858	1	TRUE
Insoluble	rplJ	A0A4Q1QWJ0	Large ribosomal subunit protein uL10	A0A4Q1QWJ0_STRPY	TRUE	TRUE	DVFVGPSAVAFSNEDVIAPAK	248.47488	248.47488	3	D[HAyneDE]VFVGPSAVAFS	59940	1	TRUE
Insoluble	rplJ	A0A4Q1QWJ0	Large ribosomal subunit protein uL10	A0A4Q1QWJ0_STRPY	TRUE	TRUE	DVFVGPSAVAFSNEDVIAPAK	271.43375	271.43375	3	D[HAyneDE]VFVGPSAVAFS	59894	1	FALSE
Insoluble	rplJ	A0A4Q1QWJ0	Large ribosomal subunit protein uL10	A0A4Q1QWJ0_STRPY	TRUE	TRUE	DVFVGPSAVAFSNEDVIAPAK	281.63986	281.63986	2	DVFVGPSAVAFSNED[HAyn	61282	1	TRUE
Insoluble	rplJ	A0A4Q1QWJ0	Large ribosomal subunit protein uL10	A0A4Q1QWJ0_STRPY	TRUE	TRUE	DVFVGPSAVAFSNEDVIAPAK	281.63986	281.63986	2	DVFVGPSAVAFSNED[HAyn	61277	2	TRUE
Insoluble	rplJ	A0A4Q1QWJ0	Large ribosomal subunit protein uL10	A0A4Q1QWJ0_STRPY	TRUE	TRUE	DVFVGPSAVAFSNEDVIAPAK	231.40572	231.40572	3	DVFVGPSAVAFSNED[HAyn	61327	1	TRUE
Insoluble	rplJ	A0A4Q1QWJ0	Large ribosomal subunit protein uL10	A0A4Q1QWJ0_STRPY	TRUE	TRUE	DVFVGPSAVAFSNEDVIAPAK	216.58743	216.58743	3	DVFVGPSAVAFSNED[HAyn	61389	1	TRUE
Insoluble	rplJ	A0A4Q1QWJ0	Large ribosomal subunit protein uL10	A0A4Q1QWJ0_STRPY	TRUE	TRUE	TADALEIK	456.51562	456.51562	2	TAD[HAyneDE]ALEIK	33118	1	TRUE
Insoluble	rplJ	A0A4Q1QWJ0	Large ribosomal subunit protein uL10	A0A4Q1QWJ0_STRPY	TRUE	TRUE	TADALEIK	358.63385	358.63385	3	TAD[HAyneDE]ALEIK	33234	1	TRUE
Insoluble	rplK	A0A4U7H3X4	Large ribosomal subunit protein uL11	A0A4U7H3X4_STRPY	TRUE	TRUE	TADQAGMIIPVVISVYEDK	477.72598	477.72598	3	TADQAGM[Oxidation (M)]I	68084	1	TRUE
Insoluble	rplK	A0A4U7H3X4	Large ribosomal subunit protein uL11	A0A4U7H3X4_STRPY	TRUE	TRUE	TADQAGMIIPVVISVYEDK	435.64468	435.64468	3	TADQAGM[Oxidation (M)]I	69734	1	TRUE
Insoluble	rplK	A0A4U7H3X4	Large ribosomal subunit protein uL11	A0A4U7H3X4_STRPY	TRUE	TRUE	TADQAGMIIPVVISVYEDK	345.30466	345.30466	4	TADQAGM[Oxidation (M)]I	68173	1	TRUE
Insoluble	rplK	A0A4U7H3X4	Large ribosomal subunit protein uL11	A0A4U7H3X4_STRPY	TRUE	TRUE	SFDFITK	296.82166	296.82166	3	SFD[HAyneDE]FITK	44208	1	TRUE
Insoluble	rplK	A0A4U7H3X4	Large ribosomal subunit protein uL11	A0A4U7H3X4_STRPY	TRUE	TRUE	SFDFITK	337.66803	337.66803	2	SFD[HAyneDE]FITK	44205	1	TRUE
Insoluble	rplK	A0A4U7H3X4	Large ribosomal subunit protein uL11	A0A4U7H3X4_STRPY	TRUE	TRUE	SFDFITK	355.51566	355.51566	2	SFD[HAyneDE]FITK	44141	1	TRUE
Soluble	rplK	A0A4U7H3X4	Large ribosomal subunit protein uL11	A0A4U7H3X4_STRPY	TRUE	TRUE	TADQAGMIIPVVISVYEDK	298.46933	298.46933	3	TADQAGM[Oxidation (M)]I	65619	2	TRUE
Soluble	rplK	A0A4U7H3X4	Large ribosomal subunit protein uL11	A0A4U7H3X4_STRPY	TRUE	TRUE	TADQAGMIIPVVISVYEDK	535.0772	535.0772	3	TAD[HAyneDE]QAGM[Oxid	65719	2	TRUE
Soluble	rplK	A0A4U7H3X4	Large ribosomal subunit protein uL11	A0A4U7H3X4_STRPY	TRUE	TRUE	TADQAGMIIPVVISVYEDK	635.23346	635.23346	3	TAD[HAyneDE]QAGM[Oxid	65823	2	TRUE
Soluble	rplK	A0A4U7H3X4	Large ribosomal subunit protein uL11	A0A4U7H3X4_STRPY	TRUE	TRUE	TADQAGMIIPVVISVYEDK	296.7644	296.7644	3	TAD[HAyneDE]QAGM[Oxid	65709	1	TRUE
Soluble	rplK	A0A4U7H3X4	Large ribosomal subunit protein uL11	A0A4U7H3X4_STRPY	TRUE	TRUE	SFDFITK	125.56533	125.56533	3	SFD[HAyneDE]FITK	39994	1	TRUE
Insoluble	rplL	A0A5S4TMV4	Large ribosomal subunit protein bL12	A0A5S4TMV4_STRPY	TRUE	TRUE	DSFDVELTSAGDK	776.82184	776.82184	3	D[HAyneDE]SFDVELTSAGD	41833	1	TRUE
Insoluble	rplL	A0A5S4TMV4	Large ribosomal subunit protein bL12	A0A5S4TMV4_STRPY	TRUE	TRUE	DSFDVELTSAGDK	776.82184	776.82184	3	D[HAyneDE]SFDVELTSAGD	41825	1	TRUE
Insoluble	rplL	A0A5S4TMV4	Large ribosomal subunit protein bL12	A0A5S4TMV4_STRPY	TRUE	TRUE	DSFDVELTSAGDK	470.2533	470.2533	2	D[HAyneDE]SFDVELTSAGD	43426	1	TRUE
Insoluble	rplL	A0A5S4TMV4	Large ribosomal subunit protein bL12	A0A5S4TMV4_STRPY	TRUE	TRUE	DSFDVELTSAGDK	524.20184	524.20184	2	D[HAyneDE]SFDVELTSAGD	43442	1	TRUE
Insoluble	rplL	A0A5S4TMV4	Large ribosomal subunit protein bL12	A0A5S4TMV4_STRPY	TRUE	TRUE	DSFDVELTSAGDK	622.5468	622.5468	2	D[HAyneDE]SFDVELTSAGD	41843	1	TRUE
Insoluble	rplL	A0A5S4TMV4	Large ribosomal subunit protein bL12	A0A5S4TMV4_STRPY	TRUE	TRUE	DSFDVELTSAGDK	584.81885	584.81885	3	DSFD[HAyneDE]VELTSAGD	42771	2	TRUE
Insoluble	rplL	A0A5S4TMV4	Large ribosomal subunit protein bL12	A0A5S4TMV4_STRPY	TRUE	TRUE	DSFDVELTSAGDKK	671.7579	671.7579	3	D[HAyneDE]SFDVELTSAGD	32071	1	TRUE
Insoluble	rplL	A0A5S4TMV4	Large ribosomal subunit protein bL12	A0A5S4TMV4_STRPY	TRUE	TRUE	DSFDVELTSAGDKK	420.7922	420.7922	3	D[HAyneDE]SFDVELTSAGD	33730	1	TRUE
Insoluble	rplL	A0A5S4TMV4	Large ribosomal subunit protein bL12	A0A5S4TMV4_STRPY	TRUE	TRUE	DSFDVELTSAGDKK	675.01666	675.01666	3	D[HAyneDE]SFDVELTSAGD	32394	1	TRUE
Insoluble	rplL	A0A5S4TMV4	Large ribosomal subunit protein bL12	A0A5S4TMV4_STRPY	TRUE	TRUE	DSFDVELTSAGDKK	124.20746	124.20746	4	D[HAyneDE]SFDVELTSAGD	32148	2	TRUE
Soluble	rplL	A0A5S4TMV4	Large ribosomal subunit protein bL12	A0A5S4TMV4_STRPY	TRUE	TRUE	DSFDVELTSAGDK	590.1188	590.1188	2	D[HAyneDE]SFDVELTSAGD	37649	1	TRUE
Soluble	rplL	A0A5S4TMV4	Large ribosomal subunit protein bL12	A0A5S4TMV4_STRPY	TRUE	TRUE	DSFDVELTSAGDK	588.9293	588.9293	2	D[HAyneDE]SFDVELTSAGD	37755	1	TRUE
Soluble	rplL	A0A5S4TMV4	Large ribosomal subunit protein bL12	A0A5S4TMV4_STRPY	TRUE	TRUE	DSFDVELTSAGDK	588.93195	588.93195	2	D[HAyneDE]SFDVELTSAGD	37676	1	TRUE
Soluble	rplL	A0A5S4TMV4	Large ribosomal subunit protein bL12	A0A5S4TMV4_STRPY	TRUE	TRUE	DSFDVELTSAGDK	588.6024	588.6024	2	D[HAyneDE]SFDVELTSAGD	37807	1	FALSE
Soluble	rplL	A0A5S4TMV4	Large ribosomal subunit protein bL12	A0A5S4TMV4_STRPY	TRUE	TRUE	DSFDVELTSAGDK	571.3096	571.3096	2	D[HAyneDE]SFDVELTSAGD	39406	1	FALSE
Soluble	rplL	A0A5S4TMV4	Large ribosomal subunit protein bL12	A0A5S4TMV4_STRPY	TRUE	TRUE	DSFDVELTSAGDK	308.25095	308.25095	3	D[HAyneDE]SFDVELTSAGD	39281	1	TRUE
Soluble	rplL	A0A5S4TMV4	Large ribosomal subunit protein bL12	A0A5S4TMV4_STRPY	TRUE	TRUE	DSFDVELTSAGDK	652.06885	652.06885	3	D[HAyneDE]SFDVELTSAGD	37851	1	TRUE
Soluble	rplL	A0A5S4TMV4	Large ribosomal subunit protein bL12	A0A5S4TMV4_STRPY	TRUE	TRUE	DSFDVELTSAGDK	652.18567	652.18567	3	D[HAyneDE]SFDVELTSAGD	37700	1	TRUE
Soluble	rplL	A0A5S4TMV4	Large ribosomal subunit protein bL12	A0A5S4TMV4_STRPY	TRUE	TRUE	DSFDVELTSAGDK	652.18567	652.18567	3	D[HAyneDE]SFDVELTSAGD	37687	1	FALSE
Soluble	rplL	A0A5S4TMV4	Large ribosomal subunit protein bL12	A0A5S4TMV4_STRPY	TRUE	TRUE	DSFDVELTSAGDK	646.60364	646.60364	3	D[HAyneDE]SFDVELTSAGD	37841	1	FALSE
Soluble	rplL	A0A5S4TMV4	Large ribosomal subunit protein bL12	A0A5S4TMV4_STRPY	TRUE	TRUE	DSFDVELTSAGDK	572.0266	572.0266	2	DSFD[HAyneDE]VELTSAGD	38637	1	TRUE
Soluble	rplL	A0A5S4TMV4	Large ribosomal subunit protein bL12	A0A5S4TMV4_STRPY	TRUE	TRUE	EASILELNDLVK	252.79045	252.79045	3	EASILELND[HAyneDE]LVK	55272	1	TRUE
Soluble	rplL	A0A5S4TMV4	Large ribosomal subunit protein bL12	A0A5S4TMV4_STRPY	TRUE	TRUE	EASILELNDLVK	198.2658	198.2658	2	EASILELND[HAyneDE]LVK	55291	1	TRUE
Soluble	rplL	A0A5S4TMV4	Large ribosomal subunit protein bL12	A0A5S4TMV4_STRPY	TRUE	TRUE	EASILELNDLVK	202.52608	202.52608	2	EASILELND[HAyneDE]LVK	55242	1	TRUE
Soluble	rplL	A0A5S4TMV4	Large ribosomal subunit protein bL12	A0A5S4TMV4_STRPY	TRUE	TRUE	DSFDVELTSAGDKK	1566.4746	1566.4746	3	D[HAyneDE]SFDVELTSAGD	28392	1	TRUE
Soluble	rplL	A0A5S4TMV4	Large ribosomal subunit protein bL12	A0A5S4TMV4_STRPY	TRUE	TRUE	DSFDVELTSAGDKK	1566.4746	1566.4746	3	D[HAyneDE]SFDVELTSAGD	28386	1	TRUE
Soluble	rplL	A0A5S4TMV4	Large ribosomal subunit protein bL12	A0A5S4TMV4_STRPY	TRUE	TRUE	DSFDVELTSAGDKK	1143.983	1143.983	3	D[HAyneDE]SFDVELTSAGD	29904	1	TRUE
Soluble	rplL	A0A5S4TMV4	Large ribosomal subunit protein bL12	A0A5S4TMV4_STRPY	TRUE	TRUE	DSFDVELTSAGDKK	1143.983	1143.983	3	D[HAyneDE]SFDVELTSAGD	29784	2	FALSE
Soluble	rplL	A0A5S4TMV4	Large ribosomal subunit protein bL12	A0A5S4TMV4_STRPY	TRUE	TRUE	DSFDVELTSAGDKK	555.8496	555.8496	4	D[HAyneDE]SFDVELTSAGD	28420	1	TRUE
Soluble	rplL	A0A5S4TMV4	Large ribosomal subunit protein bL12	A0A5S4TMV4_STRPY	TRUE	TRUE	DSFDVELTSAGDKK	171.81145	171.81145	4	D[HAyneDE]SFDVELTSAGD	29937	1	TRUE
Soluble	rplL	A0A5S4TMV4	Large ribosomal subunit protein bL12	A0A5S4TMV4_STRPY	TRUE	TRUE	DSFDVELTSAGDKK	181.43822	181.43822	4	D[HAyneDE]SFDVELTSAGD	29919	1	TRUE
Soluble	rplL	A0A5S4TMV4	Large ribosomal subunit protein bL12	A0A5S4TMV4_STRPY	TRUE	TRUE	DSFDVELTSAGDKK	1143.983	1143.983	3	DSFDVELTSAGD[HAyneDE	29296	1	TRUE
Insoluble	rplN	A0A4U7HAM6	Large ribosomal subunit protein uL14	A0A4U7HAM6_STRPY	TRUE	TRUE	FDDNAAVIIRDDK	2125.2544	2125.2544	3	FDD[HAyneDE]NAAVIIRDD	32075	1	TRUE
Insoluble	rplQ	A0A4U7HK03	Large ribosomal subunit protein bL17	A0A4U7HK03_STRPY	TRUE	TRUE	NEIASENYDEATDK	593.0414	593.0414	3	NEIASENYD[HAyneDE]EAT	21090	1	TRUE
Insoluble	rplQ	A0A4U7HK03	Large ribosomal subunit protein bL17	A0A4U7HK03_STRPY	TRUE	TRUE	NEIASENYDEATDK	174.48267	174.48267	2	NEIASENYDEATD[HAyneDE	20431	1	TRUE
Insoluble	rplR	A0A2W5CH87	Large ribosomal subunit protein uL18	A0A2W5CH87_STRPY	TRUE	TRUE	GISEVVFDR	728.96765	728.96765	2	GISEVVFD[HAyneDE]R	42945	1	TRUE
Insoluble	rplR	A0A2W5CH87	Large ribosomal subunit protein uL18	A0A2W5CH87_STRPY	TRUE	TRUE	GISEVVFDR	713.18774	713.18774	2	GISEVVFD[HAyneDE]R	42936	1	TRUE
Insoluble	rplV	A0A4U7IRS7	Large ribosomal subunit protein uL22	A0A4U7IRS7_STRPY	TRUE	TRUE	VADAIAILK	447.5284	447.5284	2	VAD[HAyneDE]AIAILK	47313	1	TRUE
Insoluble	rplV	A0A4U7IRS7	Large ribosomal subunit protein uL22	A0A4U7IRS7_STRPY	TRUE	TRUE	VADAIAILK	447.80774	447.80774	2	VAD[HAyneDE]AIAILK	46590	3	TRUE
Insoluble	rplV	A0A4U7IRS7	Large ribosomal subunit protein uL22	A0A4U7IRS7_STRPY	TRUE	TRUE	VADAIAILK	354.80252	354.80252	3	VAD[HAyneDE]AIAILK	47330	1	TRUE
Insoluble	rplW	A0A4U7IP51	Large ribosomal subunit protein uL23	A0A4U7IP51_STRPY	TRUE	TRUE	AIITLTADSK	201.39711	201.39711	2	AIITLTAD[HAyneDE]SK	39020	1	TRUE
Insoluble	rplW	A0A4U7IP51	Large ribosomal subunit protein uL23	A0A4U7IP51_STRPY	TRUE	TRUE	YTFEVDTR	526.933	526.933	2	YTFEVD[HAyneDE]TR	34218	3	TRUE
Insoluble	rplW	A0A4U7IP51	Large ribosomal subunit protein uL23	A0A4U7IP51_STRPY	TRUE	TRUE	YTFEVDTR	644.6971	644.6971	2	YTFEVD[HAyneDE]TR	34208	2	TRUE
Insoluble	rpmA	A0A4U7GKU3	Large ribosomal subunit protein bL27	A0A4U7GKU3_STRPY	TRUE	TRUE	AADGQTVSGGSILYR	1729.4728	1729.4728	3	AAD[HAyneDE]GQTVSGGS	30889	1	TRUE
Insoluble	rpmA	A0A4U7GKU3	Large ribosomal subunit protein bL27	A0A4U7GKU3_STRPY	TRUE	TRUE	AADGQTVSGGSILYR	1729.4635	1729.4635	3	AAD[HAyneDE]GQTVSGGS	31122	1	TRUE
Insoluble	rpmA	A0A4U7GKU3	Large ribosomal subunit protein bL27	A0A4U7GKU3_STRPY	TRUE	TRUE	AADGQTVSGGSILYR	327.89838	327.89838	2	AAD[HAyneDE]GQTVSGGS	31110	1	TRUE
Insoluble	rpmA	A0A4U7GKU3	Large ribosomal subunit protein bL27	A0A4U7GKU3_STRPY	TRUE	TRUE	GGDDTLFAK	295.71906	295.71906	3	GGDD[HAyneDE]TLFAK	36148	1	TRUE
Insoluble	rpmA	A0A4U7GKU3	Large ribosomal subunit protein bL27	A0A4U7GKU3_STRPY	TRUE	TRUE	GGDDTLFAK	237.29428	237.29428	2	GGD[HAyneDE]DTLFAK	34895	1	TRUE
Insoluble	rpmA	A0A4U7GKU3	Large ribosomal subunit protein bL27	A0A4U7GKU3_STRPY	TRUE	TRUE	GGDDTLFAK	223.77328	223.77328	3	GGD[HAyneDE]DTLFAK	34932	1	TRUE
Insoluble	rpmD	A0A4U7HLR5	Large ribosomal subunit protein uL30	A0A4U7HLR5_STRPY	TRUE	TRUE	GMVTAISHLVTVEDVK	555.4248	555.4248	4	GM[Oxidation (M)]VTAISHI	39355	3	TRUE
Insoluble	rpmE	A0A4U7G6P9	Large ribosomal subunit protein bL31B	A0A4U7G6P9_STRPY	TRUE	TRUE	DIHPDYRPVVFLDTTTGYQFLSGSTK	386.42236	386.42236	4	DIHPDYRPVVFLD[HAyneDE	58730	1	TRUE
Insoluble	rpmE	A0A4U7G6P9	Large ribosomal subunit protein bL31B	A0A4U7G6P9_STRPY	TRUE	TRUE	DIHPDYRPVVFLDTTTGYQFLSGSTK	266.3896	266.3896	4	DIHPD[HAyneDE]YRPVVFL	57284	1	TRUE
Insoluble	rpmE	A0A4U7G6P9	Large ribosomal subunit protein bL31B	A0A4U7G6P9_STRPY	TRUE	TRUE	VEISSDSHPFYTGR	815.0315	815.0315	3	VEISSD[HAyneDE]SHPFYT(	25824	1	TRUE
Insoluble	rpmE	A0A4U7G6P9	Large ribosomal subunit protein bL31B	A0A4U7G6P9_STRPY	TRUE	TRUE	VEISSDSHPFYTGR	815.0315	815.0315	3	VEISSD[HAyneDE]SHPFYT(	25830	2	TRUE
Insoluble	rpmE	A0A4U7G6P9	Large ribosomal subunit protein bL31B	A0A4U7G6P9_STRPY	TRUE	TRUE	VEISSDSHPFYTGR	814.5051	814.5051	3	VEISSD[HAyneDE]SHPFYT(	25853	2	TRUE
Insoluble	rpmE	A0A4U7G6P9	Large ribosomal subunit protein bL31B	A0A4U7G6P9_STRPY	TRUE	TRUE	VEISSDSHPFYTGR	523.6985	523.6985	4	VEISSD[HAyneDE]SHPFYT(	25841	1	TRUE
Insoluble	rpmE	A0A4U7G6P9	Large ribosomal subunit protein bL31B	A0A4U7G6P9_STRPY	TRUE	TRUE	VEISSDSHPFYTGR	523.72217	523.72217	4	VEISSD[HAyneDE]SHPFYT(	25849	1	TRUE
Soluble	rpmE	A0A4U7G6P9	Large ribosomal subunit protein bL31B	A0A4U7G6P9_STRPY	TRUE	TRUE	VEISSDSHPFYTGR	576.28326	576.28326	3	VEISSD[HAyneDE]SHPFYT(	22921	1	TRUE
Insoluble	rpmF	A0A4U7GEH1	Large ribosomal subunit protein bL32	A0A4U7GEH1_STRPY	TRUE	TRUE	LTAPSVQFDETTGDYSR	777.224	777.224	3	LTAPSVQFD[HAyneDE]ETT(	40397	1	TRUE
Insoluble	rpmF	A0A4U7GEH1	Large ribosomal subunit protein bL32	A0A4U7GEH1_STRPY	TRUE	TRUE	LTAPSVQFDETTGDYSR	620.20886	620.20886	2	LTAPSVQFD[HAyneDE]ETT(	40320	1	TRUE
Insoluble	rpmF	A0A4U7GEH1	Large ribosomal subunit protein bL32	A0A4U7GEH1_STRPY	TRUE	TRUE	LTAPSVQFDETTGDYSR	616.6098	616.6098	2	LTAPSVQFD[HAyneDE]ETT(	40352	1	TRUE
Insoluble	rpmF	A0A4U7GEH1	Large ribosomal subunit protein bL32	A0A4U7GEH1_STRPY	TRUE	TRUE	LTAPSVQFDETTGDYSR	620.209	620.209	2	LTAPSVQFD[HAyneDE]ETT(	40489	1	TRUE
Insoluble	rpmF	A0A4U7GEH1	Large ribosomal subunit protein bL32	A0A4U7GEH1_STRPY	TRUE	TRUE	LTAPSVQFDETTGDYSR	616.6098	616.6098	2	LTAPSVQFD[HAyneDE]ETT(	40330	1	FALSE
Insoluble	rpmF	A0A4U7GEH1	Large ribosomal subunit protein bL32	A0A4U7GEH1_STRPY	TRUE	TRUE	LTAPSVQFDETTGDYSR	279.9933	279.9933	3	LTAPSVQFDETTGD[HAyne	39560	1	TRUE
Insoluble	rpmF	A0A4U7GEH1	Large ribosomal subunit protein bL32	A0A4U7GEH1_STRPY	TRUE	TRUE	LTAPSVQFDETTGDYSR	279.29193	279.29193	3	LTAPSVQFDETTGD[HAyne	39622	1	TRUE
Insoluble	rpmF	A0A4U7GEH1	Large ribosomal subunit protein bL32	A0A4U7GEH1_STRPY	TRUE	TRUE	LTAPSVQFDETTGDYSR	265.41397	265.41397	4	LTAPSVQFD[HAyneDE]ETT(	41169	1	TRUE
Insoluble	rpmI	A0A4U7I3L7	Large ribosomal subunit protein bL35	A0A4U7I3L7_STRPY	TRUE	TRUE	AGLVSSGDFK	396.23834	396.23834	3	AGLVSSGD[HAyneDE]FK	30228	1	TRUE
Insoluble	rpmI	A0A4U7I3L7	Large ribosomal subunit protein bL35	A0A4U7I3L7_STRPY	TRUE	TRUE	AGLVSSGDFK	343.62793	343.62793	2	AGLVSSGD[HAyneDE]FK	30226	1	TRUE
Insoluble	rpoA	A0A4Q1R9V3	DNA-directed RNA polymerase subunit al	A0A4Q1R9V3_STRPY	TRUE	TRUE	TIEELDLSVR	256.75336	256.75336	3	TIEELD[HAyneDE]LSVR	43240	1	TRUE
Insoluble	rpoA	A0A4Q1R9V3	DNA-directed RNA polymerase subunit al	A0A4Q1R9V3_STRPY	TRUE	TRUE	TIEELDLSVR	194.65636	194.65636	2	TIEELD[HAyneDE]LSVR	43153	1	TRUE
Insoluble	rpoA	A0A4Q1R9V3	DNA-directed RNA polymerase subunit al	A0A4Q1R9V3_STRPY	TRUE	TRUE	TIEELDLSVR	194.66736	194.66736	2	TIEELD[HAyneDE]LSVR	43054	2	TRUE
Insoluble	rpoA	A0A4Q1R9V3	DNA-directed RNA polymerase subunit al	A0A4Q1R9V3_STRPY	TRUE	TRUE	TIEELDLSVR	194.66138	194.66138	2	TIEELD[HAyneDE]LSVR	43217	1	TRUE
Insoluble	rpoA	A0A4Q1R9V3	DNA-directed RNA polymerase subunit al	A0A4Q1R9V3_STRPY	TRUE	TRUE	TIEELDLSVR	194.65636	194.65636	2	TIEELD[HAyneDE]LSVR	43188	1	FALSE
Insoluble	rpoC	A0A5S4TEB9	DNA-directed RNA polymerase subunit b	A0A5S4TEB9_STRPY	TRUE	TRUE	TADSGYLTR	250.13414	250.13414	3	TAD[HAyneDE]SGYLTR	22527	1	TRUE
Insoluble	rpoC	A0A5S4TEB9	DNA-directed RNA polymerase subunit b	A0A5S4TEB9_STRPY	TRUE	TRUE	TADSGYLTR	291.33563	291.33563	3	TAD[HAyneDE]SGYLTR	22519	1	TRUE
Insoluble	rpoC	A0A5S4TEB9	DNA-directed RNA polymerase subunit b	A0A5S4TEB9_STRPY	TRUE	TRUE	TADSGYLTR	272.7186	272.7186	3	TAD[HAyneDE]SGYLTR	22548	1	TRUE
Soluble	rpoE	A0A5S4TDF8	Probable DNA-directed RNA polymerase	A0A5S4TDF8_STRPY	TRUE	TRUE	MKLDVFAGQEK	1004.6791	1004.6791	3	[Acetyl (Protein N-term)]M	31984	1	TRUE
Soluble	rpoE	A0A5S4TDF8	Probable DNA-directed RNA polymerase	A0A5S4TDF8_STRPY	TRUE	TRUE	MKLDVFAGQEK	358.1687	358.1687	2	[Acetyl (Protein N-term)]M	32148	1	TRUE
Insoluble	rpsA	A0A5S4TG69	30S ribosomal protein S1	A0A5S4TG69_STRPY	TRUE	TRUE	GFIPASMIDTR	563.7803	563.7803	3	GFIPASM[Oxidation (M)]ID	38173	1	TRUE
Insoluble	rpsA	A0A5S4TG69	30S ribosomal protein S1	A0A5S4TG69_STRPY	TRUE	TRUE	GFIPASMIDTR	397.70114	397.70114	2	GFIPASM[Oxidation (M)]ID	38154	1	TRUE
Insoluble	rpsA	A0A5S4TG69	30S ribosomal protein S1	A0A5S4TG69_STRPY	TRUE	TRUE	GFIPASMIDTR	396.93582	396.93582	2	GFIPASM[Oxidation (M)]ID	38291	1	TRUE
Insoluble	rpsA	A0A5S4TG69	30S ribosomal protein S1	A0A5S4TG69_STRPY	TRUE	TRUE	FVGQEFDAK	323.90054	323.90054	2	FVGQEFD[HAyneDE]AK	29950	1	TRUE
Insoluble	rpsA	A0A5S4TG69	30S ribosomal protein S1	A0A5S4TG69_STRPY	TRUE	TRUE	LTSFGAFIDLGGVDGLVHVTELSHER	723.26154	723.26154	3	LTSFGAFIDLGGVD[HAyneD	70335	1	TRUE
Insoluble	rpsA	A0A5S4TG69	30S ribosomal protein S1	A0A5S4TG69_STRPY	TRUE	TRUE	DVLSVGQEVTVK	634.9913	634.9913	3	D[HAyneDE]VLSVGQEVTV	42398	1	TRUE
Insoluble	rpsA	A0A5S4TG69	30S ribosomal protein S1	A0A5S4TG69_STRPY	TRUE	TRUE	DVLSVGQEVTVK	634.9499	634.9499	3	D[HAyneDE]VLSVGQEVTV	42452	1	TRUE
Insoluble	rpsA	A0A5S4TG69	30S ribosomal protein S1	A0A5S4TG69_STRPY	TRUE	TRUE	DVLSVGQEVTVK	318.1257	318.1257	2	D[HAyneDE]VLSVGQEVTV	42360	2	TRUE
Insoluble	rpsA	A0A5S4TG69	30S ribosomal protein S1	A0A5S4TG69_STRPY	TRUE	TRUE	DVLSVGQEVTVK	318.1257	318.1257	2	D[HAyneDE]VLSVGQEVTV	42349	1	TRUE
Insoluble	rpsA	A0A5S4TG69	30S ribosomal protein S1	A0A5S4TG69_STRPY	TRUE	TRUE	RDYELPETQTGFSMADLFGDIEL	264.6466	264.6466	3	RDYELPETQTGFSM[Oxidat	75735	1	TRUE
Insoluble	rpsA	A0A5S4TG69	30S ribosomal protein S1	A0A5S4TG69_STRPY	TRUE	TRUE	RDYELPETQTGFSMADLFGDIEL	227.1998	227.1998	3	RD[HAyneDE]YELPETQTGF	74884	1	TRUE
Insoluble	rpsA	A0A5S4TG69	30S ribosomal protein S1	A0A5S4TG69_STRPY	TRUE	TRUE	ELTNDRDADINDFVK	290.18518	290.18518	4	ELTND[HAyneDE]RDADIND	34586	2	TRUE
Insoluble	rpsA	A0A5S4TG69	30S ribosomal protein S1	A0A5S4TG69_STRPY	TRUE	TRUE	ELTNDRDADINDFVK	286.0041	286.0041	3	ELTNDRD[HAyneDE]ADIND	34506	1	TRUE
Insoluble	rpsA	A0A5S4TG69	30S ribosomal protein S1	A0A5S4TG69_STRPY	TRUE	TRUE	ELTNDRDADINDFVK	125.08191	125.08191	3	ELTNDRDAD[HAyneDE]INI	33151	1	TRUE
Insoluble	rpsA	A0A5S4TG69	30S ribosomal protein S1	A0A5S4TG69_STRPY	TRUE	TRUE	VLSIDEEAGR	1131.1392	1131.1392	3	VLSID[HAyneDE]EEAGR	31303	1	TRUE
Insoluble	rpsA	A0A5S4TG69	30S ribosomal protein S1	A0A5S4TG69_STRPY	TRUE	TRUE	LAQGDVVEGK	532.87396	532.87396	3	LAQGD[HAyneDE]VVEGK	22016	1	TRUE
Insoluble	rpsA	A0A5S4TG69	30S ribosomal protein S1	A0A5S4TG69_STRPY	TRUE	TRUE	LAQGDVVEGK	532.87396	532.87396	3	LAQGD[HAyneDE]VVEGK	22001	1	TRUE
Insoluble	rpsA	A0A5S4TG69	30S ribosomal protein S1	A0A5S4TG69_STRPY	TRUE	TRUE	LAQGDVVEGK	225.60869	225.60869	2	LAQGD[HAyneDE]VVEGK	21973	1	TRUE
Insoluble	rpsA	A0A5S4TG69	30S ribosomal protein S1	A0A5S4TG69_STRPY	TRUE	TRUE	VLEVNAADER	480.44135	480.44135	3	VLEVNAAD[HAyneDE]ER	25507	1	TRUE
Insoluble	rpsA	A0A5S4TG69	30S ribosomal protein S1	A0A5S4TG69_STRPY	TRUE	TRUE	VLEVNAADER	194.29	194.29	2	VLEVNAAD[HAyneDE]ER	25561	2	TRUE
Insoluble	rpsA	A0A5S4TG69	30S ribosomal protein S1	A0A5S4TG69_STRPY	TRUE	TRUE	VLEVNAADER	194.2498	194.2498	2	VLEVNAAD[HAyneDE]ER	25521	2	TRUE
Insoluble	rpsA	A0A5S4TG69	30S ribosomal protein S1	A0A5S4TG69_STRPY	TRUE	TRUE	DTDTVTFLVSK	484.67145	484.67145	2	D[HAyneDE]TDTVTFLVSK	46810	2	TRUE
Insoluble	rpsA	A0A5S4TG69	30S ribosomal protein S1	A0A5S4TG69_STRPY	TRUE	TRUE	DTDTVTFLVSK	477.94547	477.94547	2	D[HAyneDE]TDTVTFLVSK	46741	1	TRUE
Insoluble	rpsA	A0A5S4TG69	30S ribosomal protein S1	A0A5S4TG69_STRPY	TRUE	TRUE	DTDTVTFLVSK	349.33383	349.33383	2	DTD[HAyneDE]TVTFLVSK	53629	1	TRUE
Insoluble	rpsA	A0A5S4TG69	30S ribosomal protein S1	A0A5S4TG69_STRPY	TRUE	TRUE	AGDTVEVLVLR	281.65048	281.65048	2	AGD[HAyneDE]TVEVLVLR	52959	1	TRUE
Insoluble	rpsA	A0A5S4TG69	30S ribosomal protein S1	A0A5S4TG69_STRPY	TRUE	TRUE	AGDTVEVLVLR	276.80627	276.80627	3	AGD[HAyneDE]TVEVLVLR	53004	1	TRUE
Insoluble	rpsA	A0A5S4TG69	30S ribosomal protein S1	A0A5S4TG69_STRPY	TRUE	TRUE	EVDAAENR	566.6387	566.6387	3	EVD[HAyneDE]AAENR	14122	1	TRUE
Insoluble	rpsA	A0A5S4TG69	30S ribosomal protein S1	A0A5S4TG69_STRPY	TRUE	TRUE	EVDAAENR	566.65607	566.65607	3	EVD[HAyneDE]AAENR	14127	1	TRUE
Insoluble	rpsB	A0A4U7I356	Small ribosomal subunit protein uS2	A0A4U7I356_STRPY	TRUE	TRUE	LADQAYEFVR	872.11536	872.11536	3	LAD[HAyneDE]QAYEFVR	39872	1	TRUE
Insoluble	rpsB	A0A4U7I356	Small ribosomal subunit protein uS2	A0A4U7I356_STRPY	TRUE	TRUE	LADQAYEFVR	687.8471	687.8471	2	LAD[HAyneDE]QAYEFVR	39849	1	TRUE
Insoluble	rpsB	A0A4U7I356	Small ribosomal subunit protein uS2	A0A4U7I356_STRPY	TRUE	TRUE	LADQAYEFVR	687.8471	687.8471	2	LAD[HAyneDE]QAYEFVR	39860	1	TRUE
Insoluble	rpsB	A0A4U7I356	Small ribosomal subunit protein uS2	A0A4U7I356_STRPY	TRUE	TRUE	IPDVMYVVDPHK	470.7417	470.7417	3	IPD[HAyneDE]VM[Oxidatio	31751	1	TRUE
Insoluble	rpsB	A0A4U7I356	Small ribosomal subunit protein uS2	A0A4U7I356_STRPY	TRUE	TRUE	IPDVMYVVDPHK	1458.5192	1458.5192	4	IPD[HAyneDE]VM[Oxidatio	32090	2	TRUE
Insoluble	rpsB	A0A4U7I356	Small ribosomal subunit protein uS2	A0A4U7I356_STRPY	TRUE	TRUE	LGIPVVAMVDTNADPDDIDIIIPANDDAIR	373.8823	373.8823	4	LGIPVVAM[Oxidation (M)]V	73137	3	TRUE
Insoluble	rpsB	A0A4U7I356	Small ribosomal subunit protein uS2	A0A4U7I356_STRPY	TRUE	TRUE	FLGGIEDMPR	625.8701	625.8701	3	FLGGIED[HAyneDE]M[Oxid	36576	1	TRUE
Insoluble	rpsB	A0A4U7I356	Small ribosomal subunit protein uS2	A0A4U7I356_STRPY	TRUE	TRUE	FLGGIEDMPR	625.8701	625.8701	3	FLGGIED[HAyneDE]M[Oxid	36595	1	TRUE
Insoluble	rpsB	A0A4U7I356	Small ribosomal subunit protein uS2	A0A4U7I356_STRPY	TRUE	TRUE	FLGGIEDMPR	523.11255	523.11255	2	FLGGIED[HAyneDE]M[Oxid	36710	1	TRUE
Insoluble	rpsB	A0A4U7I356	Small ribosomal subunit protein uS2	A0A4U7I356_STRPY	TRUE	TRUE	DAAANDAVILFVGTK	340.99127	340.99127	2	DAAAND[HAyneDE]AVILFV	65850	1	TRUE
Insoluble	rpsB	A0A4U7I356	Small ribosomal subunit protein uS2	A0A4U7I356_STRPY	TRUE	TRUE	DAAANDAVILFVGTK	340.99127	340.99127	2	DAAAND[HAyneDE]AVILFV	65879	1	TRUE
Insoluble	rpsB	A0A4U7I356	Small ribosomal subunit protein uS2	A0A4U7I356_STRPY	TRUE	TRUE	QAAEAVADEATR	275.37915	275.37915	3	QAAEAVAD[HAyneDE]EATF	18262	1	TRUE
Insoluble	rpsB	A0A4U7I356	Small ribosomal subunit protein uS2	A0A4U7I356_STRPY	TRUE	TRUE	LADAIIEGR	449.46918	449.46918	2	LAD[HAyneDE]AIIEGR	35980	1	TRUE
Insoluble	rpsB	A0A4U7I356	Small ribosomal subunit protein uS2	A0A4U7I356_STRPY	TRUE	TRUE	LADAIIEGR	322.04767	322.04767	3	LAD[HAyneDE]AIIEGR	35984	1	TRUE
Soluble	rpsB	A0A4U7I356	Small ribosomal subunit protein uS2	A0A4U7I356_STRPY	TRUE	TRUE	IPDVMYVVDPHK	777.41547	777.41547	3	IPD[HAyneDE]VM[Oxidatio	28149	1	TRUE
Soluble	rpsB	A0A4U7I356	Small ribosomal subunit protein uS2	A0A4U7I356_STRPY	TRUE	TRUE	LADQAYEFVRDAAANDAVILFVGTKK	291.68753	291.68753	4	LAD[HAyneDE]QAYEFVRD/	64776	1	TRUE
Soluble	rpsB	A0A4U7I356	Small ribosomal subunit protein uS2	A0A4U7I356_STRPY	TRUE	TRUE	LADQAYEFVRDAAANDAVILFVGTKK	285.47775	285.47775	4	LAD[HAyneDE]QAYEFVRD/	64809	1	TRUE
Soluble	rpsB	A0A4U7I356	Small ribosomal subunit protein uS2	A0A4U7I356_STRPY	TRUE	TRUE	FLGGIEDMPR	622.0067	622.0067	3	FLGGIED[HAyneDE]M[Oxid	32281	1	TRUE
Soluble	rpsB	A0A4U7I356	Small ribosomal subunit protein uS2	A0A4U7I356_STRPY	TRUE	TRUE	FLGGIEDMPR	622.0067	622.0067	3	FLGGIED[HAyneDE]M[Oxid	32297	1	TRUE
Soluble	rpsB	A0A4U7I356	Small ribosomal subunit protein uS2	A0A4U7I356_STRPY	TRUE	TRUE	FLGGIEDMPR	431.30566	431.30566	2	FLGGIED[HAyneDE]M[Oxid	32335	1	TRUE
Soluble	rpsB	A0A4U7I356	Small ribosomal subunit protein uS2	A0A4U7I356_STRPY	TRUE	TRUE	RMEEEGTFDVLPK	474.25647	474.25647	3	RM[Oxidation (M)]EEEGTF	28154	1	TRUE
Soluble	rpsB	A0A4U7I356	Small ribosomal subunit protein uS2	A0A4U7I356_STRPY	TRUE	TRUE	LADQAYEFVR	706.0343	706.0343	2	LAD[HAyneDE]QAYEFVR	35697	1	TRUE
Soluble	rpsB	A0A4U7I356	Small ribosomal subunit protein uS2	A0A4U7I356_STRPY	TRUE	TRUE	LADQAYEFVR	693.0403	693.0403	2	LAD[HAyneDE]QAYEFVR	35786	1	TRUE
Soluble	rpsB	A0A4U7I356	Small ribosomal subunit protein uS2	A0A4U7I356_STRPY	TRUE	TRUE	LADQAYEFVR	624.96655	624.96655	3	LAD[HAyneDE]QAYEFVR	35750	1	TRUE

Soluble	*rpsB*	A0A4U7I356	Small ribosomal subunit protein uS2	A0A4U7I356_STRPY	TRUE	TRUE	LADAIIEGR	254.28085	254.28085	2	LAD[HAyneDE]AIIEGR	31839	1	TRUE
Soluble	*rpsB*	A0A4U7I356	Small ribosomal subunit protein uS2	A0A4U7I356_STRPY	TRUE	TRUE	LADAIIEGR	242.06987	242.06987	3	LAD[HAyneDE]AIIEGR	31854	1	TRUE
Soluble	*rpsB*	A0A4U7I356	Small ribosomal subunit protein uS2	A0A4U7I356_STRPY	TRUE	TRUE	LADAIIEGR	242.16725	242.16725	3	LAD[HAyneDE]AIIEGR	31868	1	TRUE
Soluble	*rpsB*	A0A4U7I356	Small ribosomal subunit protein uS2	A0A4U7I356_STRPY	TRUE	TRUE	KLGIPVVAMVDTNADPDDIDIIIPANDDAII	323.9751	323.9751	4	KLGIPVVAM[Oxidation (M)	59403	1	TRUE
Insoluble	*rpsC*	A0A4U7GEN8	Small ribosomal subunit protein uS3	A0A4U7GEN8_STRPY	TRUE	TRUE	ELADASVSTIEIER	576.2062	576.2062	2	ELAD[HAyneDE]ASVSTIEIE	41583	1	TRUE
Insoluble	*rpsC*	A0A4U7GEN8	Small ribosomal subunit protein uS3	A0A4U7GEN8_STRPY	TRUE	TRUE	ELADASVSTIEIER	609.93805	609.93805	2	ELAD[HAyneDE]ASVSTIEIE	41520	2	TRUE
Insoluble	*rpsC*	A0A4U7GEN8	Small ribosomal subunit protein uS3	A0A4U7GEN8_STRPY	TRUE	TRUE	QPDLDAHLVGENIAR	803.0651	803.0651	4	QPDLD[HAyneDE]AHLVGE	33664	1	TRUE
Insoluble	*rpsC*	A0A4U7GEN8	Small ribosomal subunit protein uS3	A0A4U7GEN8_STRPY	TRUE	TRUE	EYADYLHEDLAIR	592.7902	592.7902	3	EYAD[HAyneDE]YLHEDLAII	39405	1	TRUE
Insoluble	*rpsC*	A0A4U7GEN8	Small ribosomal subunit protein uS3	A0A4U7GEN8_STRPY	TRUE	TRUE	EYADYLHEDLAIR	456.07727	456.07727	4	EYADYLHED[HAyneDE]LAII	42703	1	TRUE
Insoluble	*rpsC*	A0A4U7GEN8	Small ribosomal subunit protein uS3	A0A4U7GEN8_STRPY	TRUE	TRUE	EYADYLHEDLAIR	391.32416	391.32416	3	EYADYLHED[HAyneDE]LAII	42692	1	TRUE
Insoluble	*rpsC*	A0A4U7GEN8	Small ribosomal subunit protein uS3	A0A4U7GEN8_STRPY	TRUE	TRUE	EYADYLHEDLAIR	391.32397	391.32397	3	EYADYLHED[HAyneDE]LAII	43227	1	TRUE
Insoluble	*rpsC*	A0A4U7GEN8	Small ribosomal subunit protein uS3	A0A4U7GEN8_STRPY	TRUE	TRUE	EYADYLHEDLAIR	187.8307	187.8307	2	EYADYLHED[HAyneDE]LAII	42685	1	TRUE
Insoluble	*rpsD*	A0A4Q1PT68	Small ribosomal subunit protein uS4	A0A4Q1PT68_STRPY	TRUE	TRUE	VDPGQVISVR	504.40717	504.40717	2	VD[HAyneDE]PGQVISVR	33078	1	TRUE
Insoluble	*rpsD*	A0A4Q1PT68	Small ribosomal subunit protein uS4	A0A4Q1PT68_STRPY	TRUE	TRUE	VDPGQVISVR	458.25885	458.25885	3	VD[HAyneDE]PGQVISVR	33195	1	TRUE
Insoluble	*rpsD*	A0A4Q1PT68	Small ribosomal subunit protein uS4	A0A4Q1PT68_STRPY	TRUE	TRUE	VDPGQVISVR	457.37747	457.37747	3	VD[HAyneDE]PGQVISVR	33256	1	TRUE
Insoluble	*rpsD*	A0A4Q1PT68	Small ribosomal subunit protein uS4	A0A4Q1PT68_STRPY	TRUE	TRUE	VDPGQVISVR	454.45364	454.45364	3	VD[HAyneDE]PGQVISVR	33268	1	TRUE
Insoluble	*rpsD*	A0A4Q1PT68	Small ribosomal subunit protein uS4	A0A4Q1PT68_STRPY	TRUE	TRUE	VDPGQVISVR	453.69028	453.69028	3	VD[HAyneDE]PGQVISVR	33071	2	FALSE
Insoluble	*rpsE*	A0A4U7FYX7	Small ribosomal subunit protein uS5	A0A4U7FYX7_STRPY	TRUE	TRUE	AVIELAGVADITSK	711.1548	711.1548	2	AVIELAGVAD[HAyneDE]ITS	53186	1	TRUE
Insoluble	*rpsE*	A0A4U7FYX7	Small ribosomal subunit protein uS5	A0A4U7FYX7_STRPY	TRUE	TRUE	AVIELAGVADITSK	587.3997	587.3997	3	AVIELAGVAD[HAyneDE]ITS	53199	1	TRUE
Insoluble	*rpsE*	A0A4U7FYX7	Small ribosomal subunit protein uS5	A0A4U7FYX7_STRPY	TRUE	TRUE	GISVSDLA	601.0027	601.0027	2	GISVSD[HAyneDE]LA	53863	1	TRUE
Insoluble	*rpsE*	A0A4U7FYX7	Small ribosomal subunit protein uS5	A0A4U7FYX7_STRPY	TRUE	TRUE	DNAVELEER	200.5019	200.5019	2	D[HAyneDE]NAVELEER	25448	1	TRUE
Insoluble	*rpsE*	A0A4U7FYX7	Small ribosomal subunit protein uS5	A0A4U7FYX7_STRPY	TRUE	TRUE	DNAVELEER	231.13382	231.13382	2	D[HAyneDE]NAVELEER	23970	1	TRUE
Insoluble	*rpsE*	A0A4U7FYX7	Small ribosomal subunit protein uS5	A0A4U7FYX7_STRPY	TRUE	TRUE	DNAVELEER	229.39395	229.39395	2	D[HAyneDE]NAVELEER	23988	1	TRUE
Insoluble	*rpsE*	A0A4U7FYX7	Small ribosomal subunit protein uS5	A0A4U7FYX7_STRPY	TRUE	TRUE	DNAVELEER	200.498	200.498	2	D[HAyneDE]NAVELEER	25549	1	FALSE
Insoluble	*rpsF*	A0A4U7IWQ5	Small ribosomal subunit protein bS6	A0A4U7IWQ5_STRPY	TRUE	TRUE	FDSILTDNGATVVESK	236.30731	236.30731	2	FD[HAyneDE]SILTDNGATV	44946	1	TRUE
Insoluble	*rpsF*	A0A4U7IWQ5	Small ribosomal subunit protein bS6	A0A4U7IWQ5_STRPY	TRUE	TRUE	LAYEINDFR	613.40485	613.40485	2	LAYEIND[HAyneDE]FR	40908	1	TRUE
Insoluble	*rpsF*	A0A4U7IWQ5	Small ribosomal subunit protein bS6	A0A4U7IWQ5_STRPY	TRUE	TRUE	LAYEINDFR	621.29755	621.29755	2	LAYEIND[HAyneDE]FR	40965	1	TRUE
Insoluble	*rpsF*	A0A4U7IWQ5	Small ribosomal subunit protein bS6	A0A4U7IWQ5_STRPY	TRUE	TRUE	LAYEINDFR	652.37115	652.37115	3	LAYEIND[HAyneDE]FR	40961	1	TRUE
Insoluble	*rpsF*	A0A4U7IWQ5	Small ribosomal subunit protein bS6	A0A4U7IWQ5_STRPY	TRUE	TRUE	EGLYHIVNLEATDAAALNEFDR	572.6604	572.6604	4	EGLYHIVNLEATDAAALNEF	59140	1	TRUE
Insoluble	*rpsF*	A0A4U7IWQ5	Small ribosomal subunit protein bS6	A0A4U7IWQ5_STRPY	TRUE	TRUE	EGLYHIVNLEATDAAALNEFDR	408.14383	408.14383	4	EGLYHIVNLEATD[HAyneDE	58709	1	TRUE
Soluble	*rpsF*	A0A4U7IWQ5	Small ribosomal subunit protein bS6	A0A4U7IWQ5_STRPY	TRUE	TRUE	LAYEINDFR	1434.3276	1434.3276	2	LAYEIND[HAyneDE]FR	36812	1	TRUE
Soluble	*rpsF*	A0A4U7IWQ5	Small ribosomal subunit protein bS6	A0A4U7IWQ5_STRPY	TRUE	TRUE	FDSILTDNGATVVESK	385.7927	385.7927	2	FD[HAyneDE]SILTDNGATV	40809	1	TRUE
Soluble	*rpsF*	A0A4U7IWQ5	Small ribosomal subunit protein bS6	A0A4U7IWQ5_STRPY	TRUE	TRUE	FDSILTDNGATVVESK	377.27213	377.27213	2	FD[HAyneDE]SILTDNGATV	41837	1	TRUE
Soluble	*rpsF*	A0A4U7IWQ5	Small ribosomal subunit protein bS6	A0A4U7IWQ5_STRPY	TRUE	TRUE	FDSILTDNGATVVESK	679.1225	679.1225	3	FDSILTD[HAyneDE]NGATV	41628	1	TRUE
Soluble	*rpsF*	A0A4U7IWQ5	Small ribosomal subunit protein bS6	A0A4U7IWQ5_STRPY	TRUE	TRUE	FDSILTDNGATVVESK	673.58453	673.58453	3	FD[HAyneDE]SILTDNGATV	40886	1	TRUE
Soluble	*rpsF*	A0A4U7IWQ5	Small ribosomal subunit protein bS6	A0A4U7IWQ5_STRPY	TRUE	TRUE	EGLYHIVNLEATDAAALNEFDR	920.9329	920.9329	4	EGLYHIVNLEATDAAALNEF	55084	1	TRUE
Soluble	*rpsF*	A0A4U7IWQ5	Small ribosomal subunit protein bS6	A0A4U7IWQ5_STRPY	TRUE	TRUE	EGLYHIVNLEATDAAALNEFDR	775.12756	775.12756	4	EGLYHIVNLEATDAAALNEF	55075	1	TRUE
Soluble	*rpsF*	A0A4U7IWQ5	Small ribosomal subunit protein bS6	A0A4U7IWQ5_STRPY	TRUE	TRUE	EGLYHIVNLEATDAAALNEFDR	775.12756	775.12756	4	EGLYHIVNLEATD[HAyneDE	54701	1	TRUE
Soluble	*rpsF*	A0A4U7IWQ5	Small ribosomal subunit protein bS6	A0A4U7IWQ5_STRPY	TRUE	TRUE	EGLYHIVNLEATDAAALNEFDR	775.12756	775.12756	4	EGLYHIVNLEATD[HAyneDE	54560	2	TRUE
Soluble	*rpsF*	A0A4U7IWQ5	Small ribosomal subunit protein bS6	A0A4U7IWQ5_STRPY	TRUE	TRUE	EGLYHIVNLEATDAAALNEFDR	775.12756	775.12756	4	EGLYHIVNLEATD[HAyneDE	54577	2	TRUE
Soluble	*rpsF*	A0A4U7IWQ5	Small ribosomal subunit protein bS6	A0A4U7IWQ5_STRPY	TRUE	TRUE	EGLYHIVNLEATDAAALNEFDR	827.2468	827.2468	3	EGLYHIVNLEATD[HAyneDE	54816	1	TRUE
Soluble	*rpsF*	A0A4U7IWQ5	Small ribosomal subunit protein bS6	A0A4U7IWQ5_STRPY	TRUE	TRUE	LAYEINDFREGLYHIVNLEATDAAALNEFD	288.74646	288.74646	4	LAYEINDFREGLYHIVNLEAT	69736	1	TRUE
Soluble	*rpsF*	A0A4U7IWQ5	Small ribosomal subunit protein bS6	A0A4U7IWQ5_STRPY	TRUE	TRUE	LAYEINDFREGLYHIVNLEATDAAALNEFD	258.02448	258.02448	4	LAYEIND[HAyneDE]FREGL	70857	1	TRUE
Insoluble	*rpsG*	A0A5S4TWV1	Small ribosomal subunit protein uS7	A0A5S4TWV1_STRPY	TRUE	TRUE	EATGNDALEVFETAMDNIMPVLEVR	352.17093	352.17093	4	EATGNDALEVFETAM[Oxida	74534	1	TRUE
Insoluble	*rpsG*	A0A5S4TWV1	Small ribosomal subunit protein uS7	A0A5S4TWV1_STRPY	TRUE	TRUE	EATGNDALEVFETAMDNIMPVLEVR	359.42548	359.42548	4	EATGNDALEVFETAM[Oxida	74483	1	TRUE
Insoluble	*rpsG*	A0A5S4TWV1	Small ribosomal subunit protein uS7	A0A5S4TWV1_STRPY	TRUE	TRUE	EIMDAANNTGASVK	443.3371	443.3371	3	EIM[Oxidation (M)]D[HAyn	17545	1	TRUE
Insoluble	*rpsG*	A0A5S4TWV1	Small ribosomal subunit protein uS7	A0A5S4TWV1_STRPY	TRUE	TRUE	EIMDAANNTGASVK	402.9799	402.9799	3	EIM[Oxidation (M)]D[HAyn	17558	1	TRUE
Insoluble	*rpsH*	A0A4U7I6C1	Small ribosomal subunit protein uS8	A0A4U7I6C1_STRPY	TRUE	TRUE	VMTDPIADFLTR	662.55	662.55	3	VM[Oxidation (M)]TD[HAyn	70816	1	TRUE
Insoluble	*rpsH*	A0A4U7I6C1	Small ribosomal subunit protein uS8	A0A4U7I6C1_STRPY	TRUE	TRUE	VMTDPIADFLTR	600.4199	600.4199	3	VM[Oxidation (M)]TD[HAyn	71266	1	TRUE
Insoluble	*rpsH*	A0A4U7I6C1	Small ribosomal subunit protein uS8	A0A4U7I6C1_STRPY	TRUE	TRUE	VMTDPIADFLTR	477.09055	477.09055	2	VM[Oxidation (M)]TD[HAyn	71188	2	TRUE
Insoluble	*rpsH*	A0A4U7I6C1	Small ribosomal subunit protein uS8	A0A4U7I6C1_STRPY	TRUE	TRUE	VMTDPIADFLTR	477.09055	477.09055	2	VM[Oxidation (M)]TD[HAyn	71214	2	TRUE
Insoluble	*rpsH*	A0A4U7I6C1	Small ribosomal subunit protein uS8	A0A4U7I6C1_STRPY	TRUE	TRUE	VMTDPIADFLTR	194.79004	194.79004	3	VM[Oxidation (M)]TDPIAD[	60561	1	TRUE
Insoluble	*rpsH*	A0A4U7I6C1	Small ribosomal subunit protein uS8	A0A4U7I6C1_STRPY	TRUE	TRUE	NVEVIEDDK	453.14645	453.14645	2	NVEVIED[HAyneDE]DK	26357	1	TRUE
Insoluble	*rpsI*	A0A4U7HVR5	Small ribosomal subunit protein uS9	A0A4U7HVR5_STRPY	TRUE	TRUE	DVEEYIPHADLR	600.09454	600.09454	3	DVEEYIPHAD[HAyneDE]LR	39318	1	TRUE
Insoluble	*rpsI*	A0A4U7HVR5	Small ribosomal subunit protein uS9	A0A4U7HVR5_STRPY	TRUE	TRUE	DVEEYIPHADLR	370.12643	370.12643	3	D[HAyneDE]VEEYIPHADLR	37040	1	TRUE
Insoluble	*rpsI*	A0A4U7HVR5	Small ribosomal subunit protein uS9	A0A4U7HVR5_STRPY	TRUE	TRUE	DVEEYIPHADLR	370.12634	370.12634	3	D[HAyneDE]VEEYIPHADLR	37058	1	TRUE
Insoluble	*rpsI*	A0A4U7HVR5	Small ribosomal subunit protein uS9	A0A4U7HVR5_STRPY	TRUE	TRUE	LIINQPFAVTSTEGSYDVFVNVVGGGYGG	377.59427	377.59427	4	LIINQPFAVTSTEGSYD[HAyn	76354	1	TRUE
Insoluble	*rpsI*	A0A4U7HVR5	Small ribosomal subunit protein uS9	A0A4U7HVR5_STRPY	TRUE	TRUE	LIINQPFAVTSTEGSYDVFVNVVGGGYGG	297.25842	297.25842	3	LIINQPFAVTSTEGSYD[HAyn	76343	1	TRUE
Insoluble	*rpsI*	A0A4U7HVR5	Small ribosomal subunit protein uS9	A0A4U7HVR5_STRPY	TRUE	TRUE	ALLQVDPDFR	615.08386	615.08386	3	ALLQVDPD[HAyneDE]FR	49443	1	TRUE
Insoluble	*rpsI*	A0A4U7HVR5	Small ribosomal subunit protein uS9	A0A4U7HVR5_STRPY	TRUE	TRUE	ALLQVDPDFR	588.56964	588.56964	3	ALLQVD[HAyneDE]PDFR	47913	1	TRUE
Insoluble	*rpsI*	A0A4U7HVR5	Small ribosomal subunit protein uS9	A0A4U7HVR5_STRPY	TRUE	TRUE	ALLQVDPDFR	603.87885	603.87885	3	ALLQVD[HAyneDE]PDFR	48020	1	TRUE
Insoluble	*rpsI*	A0A4U7HVR5	Small ribosomal subunit protein uS9	A0A4U7HVR5_STRPY	TRUE	TRUE	ALLQVDPDFR	505.23178	505.23178	2	ALLQVD[HAyneDE]PDFR	48135	3	TRUE
Insoluble	*rpsI*	A0A4U7HVR5	Small ribosomal subunit protein uS9	A0A4U7HVR5_STRPY	TRUE	TRUE	ALLQVDPDFR	291.2061	291.2061	2	ALLQVD[HAyneDE]PDFR	47965	2	TRUE
Insoluble	*rpsI*	A0A4U7HVR5	Small ribosomal subunit protein uS9	A0A4U7HVR5_STRPY	TRUE	TRUE	ALLQVDPDFR	505.76788	505.76788	2	ALLQVD[HAyneDE]PDFR	47985	1	TRUE
Insoluble	*rpsI*	A0A4U7HVR5	Small ribosomal subunit protein uS9	A0A4U7HVR5_STRPY	TRUE	TRUE	ALLQVDPDFR	501.7621	501.7621	2	ALLQVDPD[HAyneDE]FR	49449	1	TRUE
Insoluble	*rpsJ*	A0A4U7HLX9	Small ribosomal subunit protein uS10	A0A4U7HLX9_STRPY	TRUE	TRUE	LVDIINPTQK	905.34534	905.34534	3	LVD[HAyneDE]IINPTQK	44001	2	TRUE
Insoluble	*rpsJ*	A0A4U7HLX9	Small ribosomal subunit protein uS10	A0A4U7HLX9_STRPY	TRUE	TRUE	LDLPSGVNVEIKL	466.47693	466.47693	3	LD[HAyneDE]LPSGVNVEIK	63168	1	TRUE
Insoluble	*rpsJ*	A0A4U7HLX9	Small ribosomal subunit protein uS10	A0A4U7HLX9_STRPY	TRUE	TRUE	LDLPSGVNVEIKL	466.50458	466.50458	3	LD[HAyneDE]LPSGVNVEIK	62992	1	TRUE
Insoluble	*rpsJ*	A0A4U7HLX9	Small ribosomal subunit protein uS10	A0A4U7HLX9_STRPY	TRUE	TRUE	LDLPSGVNVEIKL	216.23495	216.23495	2	LD[HAyneDE]LPSGVNVEIK	63102	1	TRUE
Insoluble	*rpsJ*	A0A4U7HLX9	Small ribosomal subunit protein uS10	A0A4U7HLX9_STRPY	TRUE	TRUE	TLDTAAEK	523.3014	523.3014	3	TLD[HAyneDE]TAAEK	16107	1	TRUE
Insoluble	*rpsJ*	A0A4U7HLX9	Small ribosomal subunit protein uS10	A0A4U7HLX9_STRPY	TRUE	TRUE	TLDTAAEK	512.55566	512.55566	3	TLD[HAyneDE]TAAEK	16112	1	TRUE
Insoluble	*rpsJ*	A0A4U7HLX9	Small ribosomal subunit protein uS10	A0A4U7HLX9_STRPY	TRUE	TRUE	TLDTAAEK	512.86584	512.86584	3	TLD[HAyneDE]TAAEK	16282	1	TRUE
Insoluble	*rpsJ*	A0A4U7HLX9	Small ribosomal subunit protein uS10	A0A4U7HLX9_STRPY	TRUE	TRUE	TLDTAAEK	410.6546	410.6546	2	TLD[HAyneDE]TAAEK	16141	1	TRUE
Insoluble	*rpsJ*	A0A4U7HLX9	Small ribosomal subunit protein uS10	A0A4U7HLX9_STRPY	TRUE	TRUE	LDLPSGVNVEIKL	450.45004	450.45004	3	LD[HAyneDE]LPSGVNVEIK	47324	1	TRUE
Insoluble	*rpsL*	A0A4U7GZU7	Small ribosomal subunit protein uS12	A0A4U7GZU7_STRPY	TRUE	TRUE	SDSPALNIGYNSHK	1711.2806	1711.2806	3	SD[HAyneDE]SPALNIGYNS	24333	1	TRUE
Insoluble	*rpsL*	A0A4U7GZU7	Small ribosomal subunit protein uS12	A0A4U7GZU7_STRPY	TRUE	TRUE	SDSPALNIGYNSHK	1711.3135	1711.3135	3	SD[HAyneDE]SPALNIGYNS	24881	1	TRUE
Insoluble	*rpsL*	A0A4U7GZU7	Small ribosomal subunit protein uS12	A0A4U7GZU7_STRPY	TRUE	TRUE	SDSPALNIGYNSHK	386.90836	386.90836	4	SD[HAyneDE]SPALNIGYNS	24810	2	TRUE
Insoluble	*rpsL*	A0A4U7GZU7	Small ribosomal subunit protein uS12	A0A4U7GZU7_STRPY	TRUE	TRUE	SDSPALNIGYNSHK	399.47098	399.47098	4	SD[HAyneDE]SPALNIGYNS	24875	2	TRUE
Insoluble	*rpsL*	A0A4U7GZU7	Small ribosomal subunit protein uS12	A0A4U7GZU7_STRPY	TRUE	TRUE	GALDTAGVADR	486.2745	486.2745	3	GALD[HAyneDE]TAGVADR	23550	1	TRUE
Insoluble	*rpsL*	A0A4U7GZU7	Small ribosomal subunit protein uS12	A0A4U7GZU7_STRPY	TRUE	TRUE	GALDTAGVADR	486.36044	486.36044	3	GALD[HAyneDE]TAGVADR	23543	1	TRUE
Insoluble	*rpsL*	A0A4U7GZU7	Small ribosomal subunit protein uS12	A0A4U7GZU7_STRPY	TRUE	TRUE	GALDTAGVADR	243.25023	243.25023	2	GALD[HAyneDE]TAGVADR	23545	1	TRUE
Insoluble	*rpsL*	A0A4U7GZU7	Small ribosomal subunit protein uS12	A0A4U7GZU7_STRPY	TRUE	TRUE	GALDTAGVADR	246.51268	246.51268	2	GALD[HAyneDE]TAGVADR	23547	1	TRUE
Insoluble	*rpsL*	A0A4U7GZU7	Small ribosomal subunit protein uS12	A0A4U7GZU7_STRPY	TRUE	TRUE	GALDTAGVADRK	379.80948	379.80948	3	GALD[HAyneDE]TAGVADRI	15418	1	TRUE
Insoluble	*rpsL*	A0A4U7GZU7	Small ribosomal subunit protein uS12	A0A4U7GZU7_STRPY	TRUE	TRUE	GALDTAGVADRK	379.75012	379.75012	3	GALD[HAyneDE]TAGVADRI	15486	1	TRUE
Insoluble	*rpsL*	A0A4U7GZU7	Small ribosomal subunit protein uS12	A0A4U7GZU7_STRPY	TRUE	TRUE	GALDTAGVADRK	379.75012	379.75012	3	GALD[HAyneDE]TAGVADRI	15482	1	TRUE
Insoluble	*rpsL*	A0A4U7GZU7	Small ribosomal subunit protein uS12	A0A4U7GZU7_STRPY	TRUE	TRUE	GALDTAGVADRK	177.58897	177.58897	3	GALDTAGVAD[HAyneDE]RI	17334	1	TRUE
Insoluble	*rpsM*	A0A4U7GJF0	Small ribosomal subunit protein uS13	A0A4U7GJF0_STRPY	TRUE	TRUE	ILAAAGISEDIR	981.39343	981.39343	2	ILAAAGISED[HAyneDE]IR	40768	1	TRUE
Insoluble	*rpsM*	A0A4U7GJF0	Small ribosomal subunit protein uS13	A0A4U7GJF0_STRPY	TRUE	TRUE	ILAAAGISEDIR	922.4538	922.4538	3	ILAAAGISED[HAyneDE]IR	40771	1	TRUE
Insoluble	*rpsP*	A0A4V6YCY5	Small ribosomal subunit protein bS16	A0A4V6YCY5_STRPY	TRUE	TRUE	INVADSR	255.00838	255.00838	3	INVAD[HAyneDE]SR	18679	1	TRUE
Insoluble	*rpsQ*	A0A5S4TIG6	Small ribosomal subunit protein uS17	A0A5S4TIG6_STRPY	TRUE	TRUE	AHDENNVAK	127.7814	127.7814	3	AHD[HAyneDE]ENNVAK	6791	1	TRUE
Insoluble	*rpsR*	A0A2W5AW28	Small ribosomal subunit protein bS18	A0A2W5AW28_STRPY	TRUE	TRUE	IEYVDYK	296.53876	296.53876	2	IEYVD[HAyneDE]YK	26692	1	TRUE
Insoluble	*rpsR*	A0A2W5AW28	Small ribosomal subunit protein bS18	A0A2W5AW28_STRPY	TRUE	TRUE	IEYVDYK	296.5821	296.5821	2	IEYVD[HAyneDE]YK	26700	1	TRUE
Insoluble	*rpsR*	A0A2W5AW28	Small ribosomal subunit protein bS18	A0A2W5AW28_STRPY	TRUE	TRUE	VDFIAANK	354.89972	354.89972	2	VD[HAyneDE]FIAANK	34012	1	TRUE
Insoluble	*rpsR*	A0A2W5AW28	Small ribosomal subunit protein bS18	A0A2W5AW28_STRPY	TRUE	TRUE	VDFIAANK	331.8328	331.8328	3	VD[HAyneDE]FIAANK	30148	1	TRUE
Insoluble	*rpsR*	A0A2W5AW28	Small ribosomal subunit protein bS18	A0A2W5AW28_STRPY	TRUE	TRUE	VDFIAANK	314.016	314.016	3	VD[HAyneDE]FIAANK	30157	1	TRUE
Insoluble	*rpsR*	A0A2W5AW28	Small ribosomal subunit protein bS18	A0A2W5AW28_STRPY	TRUE	TRUE	DTELLSR	535.4218	535.4218	2	D[HAyneDE]TELLSR	28539	2	TRUE
Insoluble	*rpsS*	A0A2W5AJM0	Small ribosomal subunit protein uS19	A0A2W5AJM0_STRPY	TRUE	TRUE	HVPVYIQEDMVGHK	4361.648	4361.648	4	HVPVYIQED[HAyneDE]M[C	19374	3	TRUE
Insoluble	*rpsS*	A0A2W5AJM0	Small ribosomal subunit protein uS19	A0A2W5AJM0_STRPY	TRUE	TRUE	HVPVYIQEDMVGHK	2222.4443	2222.4443	3	HVPVYIQED[HAyneDE]M[C	19609	1	TRUE
Insoluble	*rpsS*	A0A2W5AJM0	Small ribosomal subunit protein uS19	A0A2W5AJM0_STRPY	TRUE	TRUE	HVPVYIQEDMVGHK	2222.4443	2222.4443	3	HVPVYIQED[HAyneDE]M[C	19619	1	TRUE
Insoluble	*secA*	A0A4U7ITZ8	Protein translocase subunit SecA	A0A4U7ITZ8_STRPY	TRUE	TRUE	IDHTDLLYPTLESK	349.4362	349.4362	3	ID[HAyneDE]HTDLLYPTLES	40873	1	TRUE
Insoluble	*secA*	A0A4U7ITZ8	Protein translocase subunit SecA	A0A4U7ITZ8_STRPY	TRUE	TRUE	IDHTDLLYPTLESK	338.7018	338.7018	3	ID[HAyneDE]HTDLLYPTLES	40734	1	TRUE
Insoluble	*secA*	A0A4U7ITZ8	Protein translocase subunit SecA	A0A4U7ITZ8_STRPY	TRUE	TRUE	IDHTDLLYPTLESK	346.33252	346.33252	3	ID[HAyneDE]HTDLLYPTLES	40715	1	TRUE
Insoluble	*secA*	A0A4U7ITZ8	Protein translocase subunit SecA	A0A4U7ITZ8_STRPY	TRUE	TRUE	IDHTDLLYPTLESK	338.7067	338.7067	3	ID[HAyneDE]HTDLLYPTLES	40863	1	FALSE
Insoluble	*secA*	A0A4U7ITZ8	Protein translocase subunit SecA	A0A4U7ITZ8_STRPY	TRUE	TRUE	IDHTDLLYPTLESK	134.30577	134.30577	2	ID[HAyneDE]HTDLLYPTLES	40785	1	TRUE
Insoluble	*secA*	A0A4U7ITZ8	Protein translocase subunit SecA	A0A4U7ITZ8_STRPY	TRUE	TRUE	IDHTDLLYPTLESK	134.30577	134.30577	2	ID[HAyneDE]HTDLLYPTLES	40767	1	TRUE
Insoluble	*secA*	A0A4U7ITZ8	Protein translocase subunit SecA	A0A4U7ITZ8_STRPY	TRUE	TRUE	MFQDMIGAIEFDVTR	137.5615	137.5615	2	M[Oxidation (M)]FQD[HAyn	61253	2	TRUE
Insoluble	*tgt*	A0A4Q1QZD0	Queuine tRNA-ribosyltransferase	A0A4Q1QZD0_STRPY	TRUE	TRUE	GVDMFDCVLPTR	584.0386	584.0386	3	GVDMFD[HAyneDE]C[Carb	50832	2	TRUE
Insoluble	*tig*	A0A4V6EN07	Trigger factor	A0A4V6EN07_STRPY	TRUE	TRUE	QGISPEMYFQLTGTTQEDLHNQYSAEADI	918.4438	918.4438	4	QGISPEM[Oxidation (M)]YI	48119	1	TRUE
Insoluble	*tig*	A0A4V6EN07	Trigger factor	A0A4V6EN07_STRPY	TRUE	TRUE	QGISPEMYFQLTGTTQEDLHNQYSAEADI	890.7667	890.7667	4	QGISPEM[Oxidation (M)]YI	47528	1	TRUE
Insoluble	*tig*	A0A4V6EN07	Trigger factor	A0A4V6EN07_STRPY	TRUE	TRUE	QGISPEMYFQLTGTTQEDLHNQYSAEADI	918.4438	918.4438	4	QGISPEM[Oxidation (M)]YI	47916	2	TRUE
Insoluble	*tig*	A0A4V6EN07	Trigger factor	A0A4V6EN07_STRPY	TRUE	TRUE	QGISPEMYFQLTGTTQEDLHNQYSAEADI	607.1594	607.1594	5	QGISPEM[Oxidation (M)]YI	43245	1	TRUE
Insoluble	*tig*	A0A4V6EN07	Trigger factor	A0A4V6EN07_STRPY	TRUE	TRUE	GDNFSLELGSGQFIPGFEDQLVGAK	575.91644	575.91644	4	GDNFSLELGSGQFIPGFED[	73515	1	TRUE
Insoluble	*tig*	A0A4V6EN07	Trigger factor	A0A4V6EN07_STRPY	TRUE	TRUE	GDNFSLELGSGQFIPGFEDQLVGAK	280.8421	280.8421	4	GD[HAyneDE]NFSLELGSG	74029	1	TRUE
Insoluble	*tig*	A0A4V6EN07	Trigger factor	A0A4V6EN07_STRPY	TRUE	TRUE	GDNFSLELGSGQFIPGFEDQLVGAK	343.28864	343.28864	4	GD[HAyneDE]NFSLELGSG	74017	1	TRUE
Insoluble	*tig*	A0A4V6EN07	Trigger factor	A0A4V6EN07_STRPY	TRUE	TRUE	GDNFSLELGSGQFIPGFEDQLVGAK	854.57104	854.57104	3	GDNFSLELGSGQFIPGFED[	73549	1	TRUE
Insoluble	*tig*	A0A4V6EN07	Trigger factor	A0A4V6EN07_STRPY	TRUE	TRUE	AEGFEATDSEIEQEINDLATEYNMPADOV	266.78928	266.78928	4	AEGFEATD[HAyneDE]SEIE(	75853	1	TRUE
Insoluble	*tig*	A0A4V6EN07	Trigger factor	A0A4V6EN07_STRPY	TRUE	TRUE	AEGFEATDSEIEQEINDLATEYNMPADOV	343.15768	343.15768	4	AEGFEATD[HAyneDE]SEIE(	72274	1	TRUE
Insoluble	*tig*	A0A4V6EN07	Trigger factor	A0A4V6EN07_STRPY	TRUE	TRUE	AEGFEATDSEIEQEINDLATEYNMPADOV	343.19336	343.19336	4	AEGFEATD[HAyneDE]SEIE(	72170	1	TRUE
Insoluble	*tig*	A0A4V6EN07	Trigger factor	A0A4V6EN07_STRPY	TRUE	TRUE	AEGFEATDSEIEQEINDLATEYNMPADOV	343.19336	343.19336	4	AEGFEATD[HAyneDE]SEIE(	72137	1	FALSE
Insoluble	*tig*	A0A4V6EN07	Trigger factor	A0A4V6EN07_STRPY	TRUE	TRUE	AGDEVEVNVTFPESYQAEDLAGK	559.0471	559.0471	3	AGD[HAyneDE]EVEVNVTFF	52227	1	TRUE
Soluble	*tig*	A0A4V6EN07	Trigger factor	A0A4V6EN07_STRPY	TRUE	TRUE	QGISPEMYFQLTGTTQEDLHNQYSAEADI	2577.669	2577.669	4	QGISPEM[Oxidation (M)]YI	43501	1	TRUE
Soluble	*tig*	A0A4V6EN07	Trigger factor	A0A4V6EN07_STRPY	TRUE	TRUE	QGISPEMYFQLTGTTQEDLHNQYSAEADI	2434.516	2434.516	4	QGISPEM[Oxidation (M)]YI	43292	1	TRUE
Soluble	*tig*	A0A4V6EN07	Trigger factor	A0A4V6EN07_STRPY	TRUE	TRUE	EVPELDDELAK	624.5294	624.5294	3	EVPELD[HAyneDE]DELAK	35269	1	TRUE
Soluble	*tig*	A0A4V6EN07	Trigger factor	A0A4V6EN07_STRPY	TRUE	TRUE	EVPELDDELAK	579.6101	579.6101	3	EVPELD[HAyneDE]DELAK	35258	1	TRUE
Soluble	*tig*	A0A4V6EN07	Trigger factor	A0A4V6EN07_STRPY	TRUE	TRUE	EVPELDDELAK	568.81995	568.81995	3	EVPELDD[HAyneDE]ELAK	36540	1	TRUE
Soluble	*tig*	A0A4V6EN07	Trigger factor	A0A4V6EN07_STRPY	TRUE	TRUE	EVPELDDELAK	555.8979	555.8979	2	EVPELD[HAyneDE]DELAK	35183	1	TRUE
Soluble	*tig*	A0A4V6EN07	Trigger factor	A0A4V6EN07_STRPY	TRUE	TRUE	EVPELDDELAK	545.41174	545.41174	2	EVPELD[HAyneDE]DELAK	35299	1	TRUE
Soluble	*tig*	A0A4V6EN07	Trigger factor	A0A4V6EN07_STRPY	TRUE	TRUE	QGISPEMYFQLTGTTQEDLHNQYSAEADI	1062.5082	1062.5082	4	QGISPEM[Oxidation (M)]YI	39561	1	TRUE
Soluble	*tig*	A0A4V6EN07	Trigger factor	A0A4V6EN07_STRPY	TRUE	TRUE	QGISPEMYFQLTGTTQEDLHNQYSAEADI	996.9202	996.9202	4	QGISPEM[Oxidation (M)]YI	39318	1	TRUE
Soluble	*tig*	A0A4V6EN07	Trigger factor	A0A4V6EN07_STRPY	TRUE	TRUE	AGDEVEVNVTFPESYQAEDLAGK	1511.3905	1511.3905	3	AGD[HAyneDE]EVEVNVTFF	48374	1	TRUE
Soluble	*tig*	A0A4V6EN07	Trigger factor	A0A4V6EN07_STRPY	TRUE	TRUE	AGDEVEVNVTFPESYQAEDLAGK	1511.3905	1511.3905	3	AGD[HAyneDE]EVEVNVTFF	48358	1	TRUE
Soluble	*tig*	A0A4V6EN07	Trigger factor	A0A4V6EN07_STRPY	TRUE	TRUE	SLLSADMLK	464.53125	464.53125	3	SLLSAD[HAyneDE]M[Oxida	34014	1	TRUE
Soluble	*tig*	A0A4V6EN07	Trigger factor	A0A4V6EN07_STRPY	TRUE	TRUE	GDNFSLELGSGQFIPGFEDQLVGAK	1577.102	1577.102	4	GDNFSLELGSGQFIPGFED[	69230	2	TRUE
Soluble	*tig*	A0A4V6EN07	Trigger factor	A0A4V6EN07_STRPY	TRUE	TRUE	GDNFSLELGSGQFIPGFEDQLVGAK	1572.8817	1572.8817	4	GD[HAyneDE]NFSLELGSG	69726	1	TRUE
Soluble	*tig*	A0A4V6EN07	Trigger factor	A0A4V6EN07_STRPY	TRUE	TRUE	GDNFSLELGSGQFIPGFEDQLVGAK	1564.6721	1564.6721	4	GD[HAyneDE]NFSLELGSG	70221	1	TRUE
Soluble	*tig*	A0A4V6EN07	Trigger factor	A0A4V6EN07_STRPY	TRUE	TRUE	GDNFSLELGSGQFIPGFEDQLVGAK	1572.8817	1572.8817	4	GD[HAyneDE]NFSLELGSG	69868	1	TRUE
Soluble	*tig*	A0A4V6EN07	Trigger factor	A0A4V6EN07_STRPY	TRUE	TRUE	GDNFSLELGSGQFIPGFEDQLVGAK	1523.4535	1523.4535	3	GD[HAyneDE]NFSLELGSG	70062	2	TRUE
Soluble	*tig*	A0A4V6EN07	Trigger factor	A0A4V6EN07_STRPY	TRUE	TRUE	GDNFSLELGSGQFIPGFEDQLVGAK	1557.4314	1557.4314	3	GD[HAyneDE]NFSLELGSG	69815	1	TRUE
Soluble	*tig*	A0A4V6EN07	Trigger factor	A0A4V6EN07_STRPY	TRUE	TRUE	GDNFSLELGSGQFIPGFEDQLVGAK	1525.8513	1525.8513	3	GD[HAyneDE]NFSLELGSG	69935	2	TRUE
Soluble	*tig*	A0A4V6EN07	Trigger factor	A0A4V6EN07_STRPY	TRUE	TRUE	NLVVEVDASK	820.81067	820.81067	2	NLVVEVD[HAyneDE]ASK	30187	1	TRUE
Soluble	*tig*	A0A4V6EN07	Trigger factor	A0A4V6EN07_STRPY	TRUE	TRUE	NLVVEVDASK	312.70346	312.70346	3	NLVVEVD[HAyneDE]ASK	31512	2	TRUE
Soluble	*tig*	A0A4V6EN07	Trigger factor	A0A4V6EN07_STRPY	TRUE	TRUE	AEGFEATDSEIEQEINDLATEYNMPADOV	528.2046	528.2046	4	AEGFEATD[HAyneDE]SEIE(	68009	1	TRUE
Soluble	*tig*	A0A4V6EN07	Trigger factor	A0A4V6EN07_STRPY	TRUE	TRUE	AEGFEATDSEIEQEINDLATEYNMPADOV	887.8029	887.8029	3	AEGFEATD[HAyneDE]SEIE(	67974	1	TRUE
Soluble	*tig*	A0A4V6EN07	Trigger factor	A0A4V6EN07_STRPY	TRUE	TRUE	AEGFEATDSEIEQEINDLATEYNMPADOV	894.65894	894.65894	3	AEGFEATD[HAyneDE]SEIE(	67905	1	TRUE
Soluble	*tig*	A0A4V6EN07	Trigger factor	A0A4V6EN07_STRPY	TRUE	TRUE	AEGFEATDSEIEQEINDLATEYNMPADOV	887.52844	887.52844	3	AEGFEATDSEIEQEIND[HAy	67817	1	TRUE
Soluble	*tig*	A0A4V6EN07	Trigger factor	A0A4V6EN07_STRPY	TRUE	TRUE	IDVVSMEK	304.9663	304.9663	3	ID[HAyneDE]VVSM[Oxidati	20105	1	TRUE
Soluble	*tig*	A0A4V6EN07	Trigger factor	A0A4V6EN07_STRPY	TRUE	TRUE	EVSDEDVDAK	155.18428	155.18428	2	EVSD[HAyneDE]EDVDAK	14943	1	TRUE
Soluble	*tig*	A0A4V6EN07	Trigger factor	A0A4V6EN07_STRPY	TRUE	TRUE	EVSDEDVDAK	132.4287	132.4287	3	EVSD[HAyneDE]EDVDAK	14983	1	TRUE
Soluble	*tig*	A0A4V6EN07	Trigger factor	A0A4V6EN07_STRPY	TRUE	TRUE	EVSDEDVDAK	243.42773	243.42773	3	EVSD[HAyneDE]EDVDAK	14988	1	TRUE
Soluble	*tig*	A0A4V6EN07	Trigger factor	A0A4V6EN07_STRPY	TRUE	TRUE	DLNAPGFR	287.9799	287.9799	2	D[HAyneDE]LNAPGFR	27650	1	TRUE
Soluble	*tig*	A0A4V6EN07	Trigger factor	A0A4V6EN07_STRPY	TRUE	TRUE	DLNAPGFR	287.9799	287.9799	2	D[HAyneDE]LNAPGFR	27626	1	TRUE
Soluble	*tig*	A0A4V6EN07	Trigger factor	A0A4V6EN07_STRPY	TRUE	TRUE	DLNAPGFR	309.30524	309.30524	2	D[HAyneDE]LNAPGFR	27703	1	TRUE
Soluble	*tig*	A0A4V6EN07	Trigger factor	A0A4V6EN07_STRPY	TRUE	TRUE	DLNAPGFR	305.55646	305.55646	2	D[HAyneDE]LNAPGFR	27694	1	FALSE
Soluble	*tig*	A0A4V6EN07	Trigger factor	A0A4V6EN07_STRPY	TRUE	TRUE	DLNAPGFR	219.21992	219.21992	3	D[HAyneDE]LNAPGFR	27681	1	TRUE
Soluble	*tig*	A0A4V6EN07	Trigger factor	A0A4V6EN07_STRPY	TRUE	TRUE	DLNAPGFR	291.2349	291.2349	3	D[HAyneDE]LNAPGFR	27705	1	TRUE
Insoluble	*tkt*	A0A5S4TNA3	Transketolase	A0A5S4TNA3_STRPY	TRUE	TRUE	LAMDTQEALEAEGIHVR	130.113	130.113	3	LAM[Oxidation (M)]D[HAyn	32025	1	TRUE
Insoluble	*tkt*	A0A5S4TNA3	Transketolase	A0A5S4TNA3_STRPY	TRUE	TRUE	TFDAIDQLAVNTVR	302.6762	302.6762	2	[Acetyl (Protein N-term)]TF	66394	1	TRUE
Insoluble	*tkt*	A0A5S4TNA3	Transketolase	A0A5S4TNA3_STRPY	TRUE	TRUE	DGNDLEEIAAAIEAAK	257.01498	257.01498	3	D[HAyneDE]GNDLEEIAAAII	72456	1	TRUE
Insoluble	*tkt*	A0A5S4TNA3	Transketolase	A0A5S4TNA3_STRPY	TRUE	TRUE	DGNDLEEIAAAIEAAK	254.84714	254.84714	3	DGND[HAyneDE]LEEIAAAII	72442	1	TRUE
Insoluble	*tmpC*	A0A4U7HDF6	BMP family ABC transporter substrate-bi	A0A4U7HDF6_STRPY	TRUE	TRUE	DNVASVTFADHEAAYLAGIAAAK	702.7598	702.7598	3	DNVASVTFAD[HAyneDE]H	60069	1	TRUE
Insoluble	*tmpC*	A0A4U7HDF6	BMP family ABC transporter substrate-bi	A0A4U7HDF6_STRPY	TRUE	TRUE	DNVASVTFADHEAAYLAGIAAAK	703.296	703.296	3	DNVASVTFAD[HAyneDE]H	60044	1	TRUE
Insoluble	*tmpC*	A0A4U7HDF6	BMP family ABC transporter substrate-bi	A0A4U7HDF6_STRPY	TRUE	TRUE	TIAAAQYAAGADVIYQAAGGTGAGVFNEA	1743.226	1743.226	4	TIAAAQYAAGAD[HAyneDE]	63688	1	TRUE
Insoluble	*tmpC*	A0A4U7HDF6	BMP family ABC transporter substrate-bi	A0A4U7HDF6_STRPY	TRUE	TRUE	FVIIDDIIEGK	137.5292	137.5292	2	FVIID[HAyneDE]DIIEGK	63456	1	TRUE
Insoluble	*tmpC*	A0A4U7HDF6	BMP family ABC transporter substrate-bi	A0A4U7HDF6_STRPY	TRUE	TRUE	SVDDTIQVK	671.6821	671.6821	3	SVD[HAyneDE]DTIQVK	26658	1	TRUE
Insoluble	*tmpC*	A0A4U7HDF6	BMP family ABC transporter substrate-bi	A0A4U7HDF6_STRPY	TRUE	TRUE	SVDDTIQVK	669.91327	669.91327	3	SVD[HAyneDE]DTIQVK	26303	1	TRUE
Insoluble	*tmpC*	A0A4U7HDF6	BMP family ABC transporter substrate-bi	A0A4U7HDF6_STRPY	TRUE	TRUE	SVDDTIQVK	667.38104	667.38104	3	SVD[HAyneDE]DTIQVK	26676	1	TRUE
Insoluble	*tmpC*	A0A4U7HDF6	BMP family ABC transporter substrate-bi	A0A4U7HDF6_STRPY	TRUE	TRUE	SVDDTIQVK	669.91327	669.91327	3	SVDD[HAyneDE]TIQVK	26318	1	TRUE
Insoluble	*tmpC*	A0A4U7HDF6	BMP family ABC transporter substrate-bi	A0A4U7HDF6_STRPY	TRUE	TRUE	SVDDTIQVK	677.19794	677.19794	3	SVDD[HAyneDE]TIQVK	26244	1	TRUE
Insoluble	*tmpC*	A0A4U7HDF6	BMP family ABC transporter substrate-bi	A0A4U7HDF6_STRPY	TRUE	TRUE	SVDDTIQVK	260.18036	260.18036	2	SVDD[HAyneDE]TIQVK	26285	1	TRUE
Insoluble	*tmpC*	A0A4U7HDF6	BMP family ABC transporter substrate-bi	A0A4U7HDF6_STRPY	TRUE	TRUE	VAMVTDTGGVDDK	344.62042	344.62042	3	VAM[Oxidation (M)]VTD[HA	18972	1	TRUE
Insoluble	*tmpC*	A0A4U7HDF6	BMP family ABC transporter substrate-bi	A0A4U7HDF6_STRPY	TRUE	TRUE	VAMVTDTGGVDDK	344.5831	344.5831	3	VAM[Oxidation (M)]VTD[HA	18993	1	TRUE
Insoluble	*tmpC*	A0A4U7HDF6	BMP family ABC transporter substrate-bi	A0A4U7HDF6_STRPY	TRUE	TRUE	VAMVTDTGGVDDK	344.62042	344.62042	3	VAM[Oxidation (M)]VTD[HA	18948	1	TRUE
Insoluble	*tpiA*	A0A5S4TNV8	Triosephosphate isomerase	A0A5S4TNV8_STRPY	TRUE	TRUE	DVVAADFGQEVADK	425.97778	425.97778	2	DVVAAD[HAyneDE]FGQEV	43011	1	TRUE
Insoluble	*tpiA*	A0A5S4TNV8	Triosephosphate isomerase	A0A5S4TNV8_STRPY	TRUE	TRUE	VLAEMGADYVVIGHSER	789.1882	789.1882	3	VLAEM[Oxidation (M)]GAD	31542	1	TRUE
Insoluble	*tpiA*	A0A5S4TNV8	Triosephosphate isomerase	A0A5S4TNV8_STRPY	TRUE	TRUE	VLAEMGADYVVIGHSER	519.95776	519.95776	3	VLAEM[Oxidation (M)]GAD	29975	1	TRUE
Insoluble	*tpiA*	A0A5S4TNV8	Triosephosphate isomerase	A0A5S4TNV8_STRPY	TRUE	TRUE	VLAEMGADYVVIGHSER	519.95776	519.95776	3	VLAEM[Oxidation (M)]GAD	29991	1	TRUE
Insoluble	*tpiA*	A0A5S4TNV8	Triosephosphate isomerase	A0A5S4TNV8_STRPY	TRUE	TRUE	VLAEMGADYVVIGHSER	641.2004	641.2004	4	VLAEM[Oxidation (M)]GAD	30001	1	TRUE
Insoluble	*tpiA*	A0A5S4TNV8	Triosephosphate isomerase	A0A5S4TNV8_STRPY	TRUE	TRUE	VLAEMGADYVVIGHSER	641.2004	641.2004	4	VLAEM[Oxidation (M)]GAD	29977	1	TRUE
Insoluble	*tpiA*	A0A5S4TNV8	Triosephosphate isomerase	A0A5S4TNV8_STRPY	TRUE	TRUE	VLAEMGADYVVIGHSER	641.2192	641.2192	4	VLAEM[Oxidation (M)]GAD	30014	1	TRUE
Soluble	*tpiA*	A0A5S4TNV8	Triosephosphate isomerase	A0A5S4TNV8_STRPY	TRUE	TRUE	VLAEMGADYVVIGHSER	1232.2394	1232.2394	3	VLAEM[Oxidation (M)]GAD	26544	1	TRUE
Soluble	*tpiA*	A0A5S4TNV8	Triosephosphate isomerase	A0A5S4TNV8_STRPY	TRUE	TRUE	VLAEMGADYVVIGHSER	1233.5137	1233.5137	3	VLAEM[Oxidation (M)]GAD	26653	1	TRUE
Soluble	*tpiA*	A0A5S4TNV8	Triosephosphate isomerase	A0A5S4TNV8_STRPY	TRUE	TRUE	VLAEMGADYVVIGHSER	456.21252	456.21252	4	VLAEM[Oxidation (M)]GAD	25452	2	TRUE
Soluble	*tpiA*	A0A5S4TNV8	Triosephosphate isomerase	A0A5S4TNV8_STRPY	TRUE	TRUE	VLAEMGADYVVIGHSER	1502.4606	1502.4606	4	VLAEM[Oxidation (M)]GAD	26546	1	TRUE
Soluble	*tpiA*	A0A5S4TNV8	Triosephosphate isomerase	A0A5S4TNV8_STRPY	TRUE	TRUE	VLAEMGADYVVIGHSER	1509.2749	1509.2749	4	VLAEM[Oxidation (M)]GAD	26742	1	TRUE
Soluble	*tpiA*	A0A5S4TNV8	Triosephosphate isomerase	A0A5S4TNV8_STRPY	TRUE	TRUE	VLAEMGADYVVIGHSER	1506.6982	1506.6982	4	VLAEM[Oxidation (M)]GAD	26662	1	FALSE
Soluble	*tpiA*	A0A5S4TNV8	Triosephosphate isomerase	A0A5S4TNV8_STRPY	TRUE	TRUE	DYFHETDEDINKK	595.9178	595.9178	3	DYFHETDED[HAyneDE]INK	17937	1	TRUE
Soluble	*tpiA*	A0A5S4TNV8	Triosephosphate isomerase	A0A5S4TNV8_STRPY	TRUE	TRUE	DYFHETDEDINKK	460.9572	460.9572	3	D[HAyneDE]YFHETDEDINK	15611	1	TRUE
Soluble	*tpiA*	A0A5S4TNV8	Triosephosphate isomerase	A0A5S4TNV8_STRPY	TRUE	TRUE	DYFHETDEDINKK	271.45215	271.45215	3	D[HAyneDE]YFHETDEDINK	13225	1	TRUE
Soluble	*tpiA*	A0A5S4TNV8	Triosephosphate isomerase	A0A5S4TNV8_STRPY	TRUE	TRUE	DYFHETDEDINKK	271.49643	271.49643	3	D[HAyneDE]YFHETDEDINK	13238	1	TRUE
Soluble	*tpiA*	A0A5S4TNV8	Triosephosphate isomerase	A0A5S4TNV8_STRPY	TRUE	TRUE	DYFHETDEDINKK	418.66687	418.66687	4	D[HAyneDE]YFHETDEDINK	13226	1	TRUE
Soluble	*tpiA*	A0A5S4TNV8	Triosephosphate isomerase	A0A5S4TNV8_STRPY	TRUE	TRUE	DYFHETDEDINKK	890.6433	890.6433	4	D[HAyneDE]YFHETDEDINK	15630	1	TRUE
Soluble	*tpiA*	A0A5S4TNV8	Triosephosphate isomerase	A0A5S4TNV8_STRPY	TRUE	TRUE	DYFHETDEDINKK	890.62756	890.62756	4	D[HAyneDE]YFHETDEDINK	15572	1	TRUE
Soluble	*tpiA*	A0A5S4TNV8	Triosephosphate isomerase	A0A5S4TNV8_STRPY	TRUE	TRUE	SATQDDAQNMCK	840.27496	840.27496	3	SATQDD[HAyneDE]AQNM	7951	1	TRUE
Soluble	*tpiA*	A0A5S4TNV8	Triosephosphate isomerase	A0A5S4TNV8_STRPY	TRUE	TRUE	SATQDDAQNMCK	814.076	814.076	3	SATQD[HAyneDE]DAQNM	8556	1	TRUE
Soluble	*tpiA*	A0A5S4TNV8	Triosephosphate isomerase	A0A5S4TNV8_STRPY	TRUE	TRUE	DVVAADFGQEVADK	893.06604	893.06604	2	DVVAAD[HAyneDE]FGQEV	38843	1	TRUE
Soluble	*tpiA*	A0A5S4TNV8	Triosephosphate isomerase	A0A5S4TNV8_STRPY	TRUE	TRUE	DVVAADFGQEVADK	893.08563	893.08563	2	DVVAAD[HAyneDE]FGQEV	38882	1	TRUE
Soluble	*tpiA*	A0A5S4TNV8	Triosephosphate isomerase	A0A5S4TNV8_STRPY	TRUE	TRUE	DVVAADFGQEVADK	893.08264	893.08264	2	DVVAAD[HAyneDE]FGQEV	38857	1	TRUE
Soluble	*tpiA*	A0A5S4TNV8	Triosephosphate isomerase	A0A5S4TNV8_STRPY	TRUE	TRUE	DVVAADFGQEVADK	1050.7427	1050.7427	3	DVVAAD[HAyneDE]FGQEV	38900	1	TRUE
Soluble	*tpiA*	A0A5S4TNV8	Triosephosphate isomerase	A0A5S4TNV8_STRPY	TRUE	TRUE	DVVAADFGQEVADK	1018.91034	1018.91034	3	DVVAAD[HAyneDE]FGQEV	38891	1	TRUE
Soluble	*tpiA*	A0A5S4TNV8	Triosephosphate isomerase	A0A5S4TNV8_STRPY	TRUE	TRUE	DVVAADFGQEVADK	643.85596	643.85596	3	DVVAADFGQEVAD[HAyneD	38871	1	TRUE
Soluble	*tpiA*	A0A5S4TNV8	Triosephosphate isomerase	A0A5S4TNV8_STRPY	TRUE	TRUE	RDYFHETDEDINKK	358.63696	358.63696	4	RDYFHETD[HAyneDE]EDIN	12246	1	TRUE
Soluble	*tpiA*	A0A5S4TNV8	Triosephosphate isomerase	A0A5S4TNV8_STRPY	TRUE	TRUE	AVRDVVAADFGQEVADK	341.72852	341.72852	3	AVRDVVAAD[HAyneDE]FG	33551	1	TRUE
Soluble	*tpiA*	A0A5S4TNV8	Triosephosphate isomerase	A0A5S4TNV8_STRPY	TRUE	TRUE	AVRDVVAADFGQEVADK	401.98422	401.98422	3	AVRDVVAAD[HAyneDE]FG	33541	1	TRUE
Insoluble	*trxA*	A0A5S4TDJ9	Thioredoxin	A0A5S4TDJ9_STRPY	TRUE	TRUE	ALEVTDATFVEETK	178.52606	178.52606	2	ALEVTD[HAyneDE]ATFVEE	42980	1	TRUE
Soluble	*trxA*	A0A5S4TDJ9	Thioredoxin	A0A5S4TDJ9_STRPY	TRUE	TRUE	ALEVTDATFVEETK	665.3081	665.3081	2	ALEVTD[HAyneDE]ATFVEE	38677	1	TRUE
Soluble	*trxA*	A0A5S4TDJ9	Thioredoxin	A0A5S4TDJ9_STRPY	TRUE	TRUE	ALEVTDATFVEETK	660.7177	660.7177	2	ALEVTD[HAyneDE]ATFVEE	38990	1	TRUE
Soluble	*trxA*	A0A5S4TDJ9	Thioredoxin	A0A5S4TDJ9_STRPY	TRUE	TRUE	ALEVTDATFVEETK	665.3082	665.3082	2	ALEVTD[HAyneDE]ATFVEE	38688	1	TRUE
Soluble	*trxA*	A0A5S4TDJ9	Thioredoxin	A0A5S4TDJ9_STRPY	TRUE	TRUE	ALEVTDATFVEETK	660.7357	660.7357	2	ALEVTD[HAyneDE]ATFVEE	38930	1	FALSE
Soluble	*trxA*	A0A5S4TDJ9	Thioredoxin	A0A5S4TDJ9_STRPY	TRUE	TRUE	ALEVTDATFVEETK	826.2777	826.2777	3	ALEVTD[HAyneDE]ATFVEE	38646	1	TRUE
Soluble	*trxA*	A0A5S4TDJ9	Thioredoxin	A0A5S4TDJ9_STRPY	TRUE	TRUE	ALEVTDATFVEETK	798.80286	798.80286	3	ALEVTD[HAyneDE]ATFVEE	38693	1	TRUE

Soluble	*trxA*	A0A5S4TDJ9	Thioredoxin	A0A5S4TDJ9_STRPY	TRUE	TRUE	KDGEVVK	1348.9536	1348.9536	3	KD[HAyneDE]GEVVK	7781	1	TRUE
Soluble	*trxA*	A0A5S4TDJ9	Thioredoxin	A0A5S4TDJ9_STRPY	TRUE	TRUE	KDGEVVK	1349.0533	1349.0533	3	KD[HAyneDE]GEVVK	7843	1	TRUE
Soluble	*trxA*	A0A5S4TDJ9	Thioredoxin	A0A5S4TDJ9_STRPY	TRUE	TRUE	KDGEVVK	324.72644	324.72644	3	KD[HAyneDE]GEVVK	7161	1	TRUE
Soluble	*trxA*	A0A5S4TDJ9	Thioredoxin	A0A5S4TDJ9_STRPY	TRUE	TRUE	KDGEVVK	291.67337	291.67337	2	KD[HAyneDE]GEVVK	7753	1	TRUE
Soluble	*trxA*	A0A5S4TDJ9	Thioredoxin	A0A5S4TDJ9_STRPY	TRUE	TRUE	MDVDENPETAR	798.4506	798.4506	3	M[Oxidation (M)]DVD[HAyn	14698	1	TRUE
Soluble	*trxA*	A0A5S4TDJ9	Thioredoxin	A0A5S4TDJ9_STRPY	TRUE	TRUE	MDVDENPETAR	758.9445	758.9445	3	M[Oxidation (M)]DVD[HAyn	14475	1	TRUE
Soluble	*trxA*	A0A5S4TDJ9	Thioredoxin	A0A5S4TDJ9_STRPY	TRUE	TRUE	MDVDENPETAR	730.1334	730.1334	3	M[Oxidation (M)]D[HAyneD	13583	1	TRUE
Soluble	*trxA*	A0A5S4TDJ9	Thioredoxin	A0A5S4TDJ9_STRPY	TRUE	TRUE	MDVDENPETAR	712.4761	712.4761	3	M[Oxidation (M)]D[HAyneD	13678	1	TRUE
Soluble	*trxA*	A0A5S4TDJ9	Thioredoxin	A0A5S4TDJ9_STRPY	TRUE	TRUE	MDVDENPETAR	737.19055	737.19055	3	M[Oxidation (M)]D[HAyneD	13754	1	TRUE
Soluble	*trxA*	A0A5S4TDJ9	Thioredoxin	A0A5S4TDJ9_STRPY	TRUE	TRUE	MDVDENPETAR	744.945	744.945	3	M[Oxidation (M)]D[HAyneD	13687	1	FALSE
Soluble	*trxA*	A0A5S4TDJ9	Thioredoxin	A0A5S4TDJ9_STRPY	TRUE	TRUE	MDVDENPETAR	296.53705	296.53705	2	M[Oxidation (M)]D[HAyneD	13635	1	TRUE
Insoluble	*tsf*	A0A5S4TRH8	Elongation factor Ts	A0A5S4TRH8_STRPY	TRUE	TRUE	TVEAYLDSVNAK	434.29147	434.29147	3	TVEAYLD[HAyneDE]SVNAK	35614	1	TRUE
Insoluble	*tsf*	A0A5S4TRH8	Elongation factor Ts	A0A5S4TRH8_STRPY	TRUE	TRUE	TVEAYLDSVNAK	219.13478	219.13478	2	TVEAYLD[HAyneDE]SVNAK	35628	1	TRUE
Insoluble	*tsf*	A0A5S4TRH8	Elongation factor Ts	A0A5S4TRH8_STRPY	TRUE	TRUE	TVEAYLDSVNAK	219.29733	219.29733	2	TVEAYLD[HAyneDE]SVNAK	35624	1	TRUE
Insoluble	*tsf*	A0A5S4TRH8	Elongation factor Ts	A0A5S4TRH8_STRPY	TRUE	TRUE	TVEAYLDSVNAK	217.90424	217.90424	2	TVEAYLD[HAyneDE]SVNAK	35654	3	TRUE
Insoluble	*tsf*	A0A5S4TRH8	Elongation factor Ts	A0A5S4TRH8_STRPY	TRUE	TRUE	IGVISVVEGGDDALAK	410.19983	410.19983	2	IGVISVVEGGD[HAyneDE]D	53195	1	TRUE
Insoluble	*tsf*	A0A5S4TRH8	Elongation factor Ts	A0A5S4TRH8_STRPY	TRUE	TRUE	IGVISVVEGGDDALAK	358.28436	358.28436	3	IGVISVVEGGD[HAyneDE]D	53180	1	TRUE
Insoluble	*tsf*	A0A5S4TRH8	Elongation factor Ts	A0A5S4TRH8_STRPY	TRUE	TRUE	VDQAYTLLAQVYIMDDSK	802.9977	802.9977	3	VDQAYTLLAQVYIM[Oxidati	66739	1	TRUE
Insoluble	*tsf*	A0A5S4TRH8	Elongation factor Ts	A0A5S4TRH8_STRPY	TRUE	TRUE	DELAQLNHAIELDNESR	386.2129	386.2129	3	DELAQLNHAIELD[HAyneD	49274	1	TRUE
Insoluble	*tsf*	A0A5S4TRH8	Elongation factor Ts	A0A5S4TRH8_STRPY	TRUE	TRUE	AQLSDDVITAAEADIK	698.1213	698.1213	3	AQLSDDVITAAEAD[HAyneD	58778	1	TRUE
Insoluble	*tsf*	A0A5S4TRH8	Elongation factor Ts	A0A5S4TRH8_STRPY	TRUE	TRUE	AQLSDDVITAAEADIK	578.5102	578.5102	3	AQLSDDVITAAEAD[HAyneD	58918	1	TRUE
Insoluble	*tsf*	A0A5S4TRH8	Elongation factor Ts	A0A5S4TRH8_STRPY	TRUE	TRUE	AQLSDDVITAAEADIK	698.1213	698.1213	3	AQLSDDVITAAEAD[HAyneD	58746	1	TRUE
Insoluble	*tsf*	A0A5S4TRH8	Elongation factor Ts	A0A5S4TRH8_STRPY	TRUE	TRUE	AQLSDDVITAAEADIK	431.688	431.688	3	AQLSD[HAyneDE]DVITAAE	54183	1	TRUE
Insoluble	*tsf*	A0A5S4TRH8	Elongation factor Ts	A0A5S4TRH8_STRPY	TRUE	TRUE	AQLSDDVITAAEADIK	382.47882	382.47882	3	AQLSD[HAyneDE]DVITAAE	54288	1	TRUE
Insoluble	*tsf*	A0A5S4TRH8	Elongation factor Ts	A0A5S4TRH8_STRPY	TRUE	TRUE	AQLSDDVITAAEADIK	270.19547	270.19547	4	AQLSDDVITAAEAD[HAyneD	58798	1	TRUE
Insoluble	*tsf*	A0A5S4TRH8	Elongation factor Ts	A0A5S4TRH8_STRPY	TRUE	TRUE	AQLSDDVITAAEADIK	269.88284	269.88284	2	AQLSDD[HAyneDE]VITAAE	58901	1	TRUE
Insoluble	*tsf*	A0A5S4TRH8	Elongation factor Ts	A0A5S4TRH8_STRPY	TRUE	TRUE	AQLSDDVITAAEADIK	230.34933	230.34933	2	AQLSD[HAyneDE]DVITAAE	54153	1	TRUE
Insoluble	*tsf*	A0A5S4TRH8	Elongation factor Ts	A0A5S4TRH8_STRPY	TRUE	TRUE	AQLSDDVITAAEADIK	233.18828	233.18828	2	AQLSD[HAyneDE]DVITAAE	54223	1	TRUE
Insoluble	*tsf*	A0A5S4TRH8	Elongation factor Ts	A0A5S4TRH8_STRPY	TRUE	TRUE	AMVDKPALPFLK	611.3708	611.3708	3	AM[Oxidation (M)]VD[HAyn	41991	1	TRUE
Insoluble	*tsf*	A0A5S4TRH8	Elongation factor Ts	A0A5S4TRH8_STRPY	TRUE	TRUE	KANDFESEVAATMAAALNN	236.23558	236.23558	3	KAND[HAyneDE]FESEVAAT	48873	2	TRUE
Insoluble	*tuf*	A0A5S4TIN0	Elongation factor Tu	A0A5S4TIN0_STRPY	FALSE	FALSE	DLLSEYDFPGDDLPVIQGSALK	1004.5073	1004.5073	3	D[HAyneDE]LLSEYDFPGD	73526	1	TRUE
Insoluble	*tuf*	A0A5S4TIN0	Elongation factor Tu	A0A5S4TIN0_STRPY	FALSE	FALSE	DLLSEYDFPGDDLPVIQGSALK	1007.4896	1007.4896	3	D[HAyneDE]LLSEYDFPGD	73668	1	TRUE
Insoluble	*tuf*	A0A5S4TIN0	Elongation factor Tu	A0A5S4TIN0_STRPY	FALSE	FALSE	DLLSEYDFPGDDLPVIQGSALK	892.0792	892.0792	3	DLLSEYDFPGD[HAyneDE]D	75201	1	TRUE
Insoluble	*tuf*	A0A5S4TIN0	Elongation factor Tu	A0A5S4TIN0_STRPY	FALSE	FALSE	DLLSEYDFPGDDLPVIQGSALK	640.144	640.144	4	DLLSEYDFPGDD[HAyneDE	75312	1	TRUE
Insoluble	*tuf*	A0A5S4TIN0	Elongation factor Tu	A0A5S4TIN0_STRPY	FALSE	FALSE	DLLSEYDFPGDDLPVIQGSALK	629.7382	629.7382	4	DLLSEYDFPGDD[HAyneDE	75301	1	TRUE
Insoluble	*tuf*	A0A5S4TIN0	Elongation factor Tu	A0A5S4TIN0_STRPY	TRUE	TRUE	QLDEGLAGDNVGILLR	2262.4993	2262.4993	3	QLDEGLAGD[HAyneDE]NV	59755	1	TRUE
Insoluble	*tuf*	A0A5S4TIN0	Elongation factor Tu	A0A5S4TIN0_STRPY	TRUE	TRUE	QLDEGLAGDNVGILLR	2272.3184	2272.3184	3	QLDEGLAGD[HAyneDE]NV	59758	1	TRUE
Insoluble	*tuf*	A0A5S4TIN0	Elongation factor Tu	A0A5S4TIN0_STRPY	TRUE	TRUE	QLDEGLAGDNVGILLR	1961.735	1961.735	3	QLD[HAyneDE]EGLAGDNV	58446	1	TRUE
Insoluble	*tuf*	A0A5S4TIN0	Elongation factor Tu	A0A5S4TIN0_STRPY	TRUE	TRUE	QLDEGLAGDNVGILLR	1026.641	1026.641	2	QLD[HAyneDE]EGLAGDNV	58009	1	TRUE
Insoluble	*tuf*	A0A5S4TIN0	Elongation factor Tu	A0A5S4TIN0_STRPY	TRUE	TRUE	QLDEGLAGDNVGILLR	1026.641	1026.641	2	QLD[HAyneDE]EGLAGDNV	58018	1	TRUE
Insoluble	*tuf*	A0A5S4TIN0	Elongation factor Tu	A0A5S4TIN0_STRPY	TRUE	TRUE	QLDEGLAGDNVGILLR	595.65845	595.65845	2	QLDEGLAGD[HAyneDE]NV	59650	2	TRUE
Insoluble	*tuf*	A0A5S4TIN0	Elongation factor Tu	A0A5S4TIN0_STRPY	TRUE	TRUE	QLDEGLAGDNVGILLR	2254.1138	2254.1138	2	QLDEGLAGD[HAyneDE]NV	59611	1	TRUE
Insoluble	*tuf*	A0A5S4TIN0	Elongation factor Tu	A0A5S4TIN0_STRPY	TRUE	TRUE	QLDEGLAGDNVGILLR	2307.8975	2307.8975	2	QLDEGLAGD[HAyneDE]NV	59465	2	TRUE
Insoluble	*tuf*	A0A5S4TIN0	Elongation factor Tu	A0A5S4TIN0_STRPY	TRUE	TRUE	QLDEGLAGDNVGILLR	2254.1138	2254.1138	2	QLDEGLAGD[HAyneDE]NV	59639	3	FALSE
Insoluble	*tuf*	A0A5S4TIN0	Elongation factor Tu	A0A5S4TIN0_STRPY	TRUE	TRUE	QLDEGLAGDNVGILLR	569.6031	569.6031	4	QLDEGLAGD[HAyneDE]NV	59108	2	TRUE
Insoluble	*tuf*	A0A5S4TIN0	Elongation factor Tu	A0A5S4TIN0_STRPY	TRUE	TRUE	QLDEGLAGDNVGILLR	295.94473	295.94473	4	QLDEGLAGD[HAyneDE]NV	59137	1	TRUE
Insoluble	*tuf*	A0A5S4TIN0	Elongation factor Tu	A0A5S4TIN0_STRPY	TRUE	TRUE	QLDEGLAGDNVGILLR	565.6827	565.6827	4	QLDEGLAGD[HAyneDE]NV	59251	1	TRUE
Insoluble	*tuf*	A0A5S4TIN0	Elongation factor Tu	A0A5S4TIN0_STRPY	TRUE	TRUE	DYASIDAAPEER	1098.5817	1098.5817	3	DYASID[HAyneDE]AAPEER	30401	1	TRUE
Insoluble	*tuf*	A0A5S4TIN0	Elongation factor Tu	A0A5S4TIN0_STRPY	TRUE	TRUE	DYASIDAAPEER	489.3399	489.3399	3	D[HAyneDE]YASIDAAPEER	26862	1	TRUE
Insoluble	*tuf*	A0A5S4TIN0	Elongation factor Tu	A0A5S4TIN0_STRPY	TRUE	TRUE	DYASIDAAPEER	517.2802	517.2802	3	D[HAyneDE]YASIDAAPEER	26837	1	TRUE
Insoluble	*tuf*	A0A5S4TIN0	Elongation factor Tu	A0A5S4TIN0_STRPY	TRUE	TRUE	DYASIDAAPEER	516.3566	516.3566	3	D[HAyneDE]YASIDAAPEER	27543	1	TRUE
Insoluble	*tuf*	A0A5S4TIN0	Elongation factor Tu	A0A5S4TIN0_STRPY	TRUE	TRUE	DYASIDAAPEER	516.3566	516.3566	3	D[HAyneDE]YASIDAAPEER	27570	1	FALSE
Insoluble	*tuf*	A0A5S4TIN0	Elongation factor Tu	A0A5S4TIN0_STRPY	TRUE	TRUE	DYASIDAAPEER	302.1534	302.1534	2	DYASID[HAyneDE]AAPEER	30483	2	TRUE
Insoluble	*tuf*	A0A5S4TIN0	Elongation factor Tu	A0A5S4TIN0_STRPY	TRUE	TRUE	DYASIDAAPEER	243.14009	243.14009	2	D[HAyneDE]YASIDAAPEER	27581	1	TRUE
Insoluble	*tuf*	A0A5S4TIN0	Elongation factor Tu	A0A5S4TIN0_STRPY	TRUE	TRUE	DYASIDAAPEER	238.65266	238.65266	2	D[HAyneDE]YASIDAAPEER	27569	1	TRUE
Insoluble	*tuf*	A0A5S4TIN0	Elongation factor Tu	A0A5S4TIN0_STRPY	TRUE	TRUE	VNDEIEIVGIK	1619.5936	1619.5936	2	VND[HAyneDE]EIEIVGIK	47192	1	TRUE
Insoluble	*tuf*	A0A5S4TIN0	Elongation factor Tu	A0A5S4TIN0_STRPY	TRUE	TRUE	VNDEIEIVGIK	1200.1857	1200.1857	3	VND[HAyneDE]EIEIVGIK	47258	2	TRUE
Insoluble	*tuf*	A0A5S4TIN0	Elongation factor Tu	A0A5S4TIN0_STRPY	TRUE	TRUE	VNDEIEIVGIK	1202.5494	1202.5494	3	VND[HAyneDE]EIEIVGIK	47698	1	TRUE
Insoluble	*tuf*	A0A5S4TIN0	Elongation factor Tu	A0A5S4TIN0_STRPY	TRUE	TRUE	VNDEIEIVGIK	1202.554	1202.554	3	VND[HAyneDE]EIEIVGIK	47821	1	TRUE
Insoluble	*tuf*	A0A5S4TIN0	Elongation factor Tu	A0A5S4TIN0_STRPY	FALSE	FALSE	NMITGAAQMDGAILVVASTDGPMPQTR	491.44955	491.44955	4	NM[Oxidation (M)]ITGAAQ	42318	1	TRUE
Insoluble	*tuf*	A0A5S4TIN0	Elongation factor Tu	A0A5S4TIN0_STRPY	FALSE	FALSE	NMITGAAQMDGAILVVASTDGPMPQTR	479.6095	479.6095	4	NM[Oxidation (M)]ITGAAQ	42368	1	TRUE
Insoluble	*tuf*	A0A5S4TIN0	Elongation factor Tu	A0A5S4TIN0_STRPY	TRUE	TRUE	TTDVTGSIELPAGTEMVMPGDNVTINVELI	1217.7024	1217.7024	5	TTD[HAyneDE]VTGSIELPAG	67017	1	TRUE
Insoluble	*tuf*	A0A5S4TIN0	Elongation factor Tu	A0A5S4TIN0_STRPY	TRUE	TRUE	TTDVTGSIELPAGTEMVMPGDNVTINVELI	826.62476	826.62476	5	TTDVTGSIELPAGTEM[Oxida	67837	1	TRUE
Insoluble	*tuf*	A0A5S4TIN0	Elongation factor Tu	A0A5S4TIN0_STRPY	TRUE	TRUE	TTDVTGSIELPAGTEMVMPGDNVTINVELI	789.787	789.787	5	TTDVTGSIELPAGTEM[Oxida	67368	1	TRUE
Insoluble	*tuf*	A0A5S4TIN0	Elongation factor Tu	A0A5S4TIN0_STRPY	TRUE	TRUE	VNDEIEIVGIKEETK	735.11444	735.11444	4	VND[HAyneDE]EIEIVGIKEE	36995	1	TRUE
Insoluble	*tuf*	A0A5S4TIN0	Elongation factor Tu	A0A5S4TIN0_STRPY	TRUE	TRUE	VNDEIEIVGIKEETK	477.58524	477.58524	3	VND[HAyneDE]EIEIVGIKEE	36926	1	TRUE
Insoluble	*tuf*	A0A5S4TIN0	Elongation factor Tu	A0A5S4TIN0_STRPY	TRUE	TRUE	PLLLPVEDVFSITGR	210.65837	210.65837	3	PLLLPVED[HAyneDE]VFSIT	77110	1	TRUE
Insoluble	*tuf*	A0A5S4TIN0	Elongation factor Tu	A0A5S4TIN0_STRPY	TRUE	TRUE	PLLLPVEDVFSITGR	85.38152	85.38152	2	PLLLPVED[HAyneDE]VFSIT	77103	1	TRUE
Insoluble	*tuf*	A0A5S4TIN0	Elongation factor Tu	A0A5S4TIN0_STRPY	TRUE	TRUE	DYASIDAAPEERER	358.4868	358.4868	3	D[HAyneDE]YASIDAAPEER	20602	1	TRUE
Insoluble	*tuf*	A0A5S4TIN0	Elongation factor Tu	A0A5S4TIN0_STRPY	TRUE	TRUE	DTDKPLLLPVEDVFSITGR	399.8306	399.8306	3	D[HAyneDE]TDKPLLLPVED	70214	1	TRUE
Insoluble	*tuf*	A0A5S4TIN0	Elongation factor Tu	A0A5S4TIN0_STRPY	TRUE	TRUE	DTDKPLLLPVEDVFSITGR	334.99927	334.99927	4	D[HAyneDE]TDKPLLLPVED	70506	2	TRUE
Insoluble	*tuf*	A0A5S4TIN0	Elongation factor Tu	A0A5S4TIN0_STRPY	TRUE	TRUE	DTDKPLLLPVEDVFSITGR	324.32956	324.32956	4	D[HAyneDE]TDKPLLLPVED	70478	1	TRUE
Insoluble	*tuf*	A0A5S4TIN0	Elongation factor Tu	A0A5S4TIN0_STRPY	TRUE	TRUE	DTDKPLLLPVEDVFSITGR	324.32956	324.32956	4	DTD[HAyneDE]KPLLLPVED	70454	1	TRUE
Insoluble	*tuf*	A0A5S4TIN0	Elongation factor Tu	A0A5S4TIN0_STRPY	TRUE	TRUE	DTDKPLLLPVEDVFSITGR	334.99927	334.99927	4	DTD[HAyneDE]KPLLLPVED	70517	2	TRUE
Insoluble	*tuf*	A0A5S4TIN0	Elongation factor Tu	A0A5S4TIN0_STRPY	TRUE	TRUE	DTDKPLLLPVEDVFSITGR	324.3576	324.3576	4	DTD[HAyneDE]KPLLLPVED	70430	2	TRUE
Insoluble	*typA*	A0A5S4TJ47	Large ribosomal subunit assembly factor	A0A5S4TJ47_STRPY	TRUE	TRUE	NVAIIAHVDHGK	242.92781	242.92781	3	NVAIIAHVD[HAyneDE]HGK	17439	1	TRUE
Insoluble	*typA*	A0A5S4TJ47	Large ribosomal subunit assembly factor	A0A5S4TJ47_STRPY	TRUE	TRUE	NVAIIAHVDHGK	243.04927	243.04927	3	NVAIIAHVD[HAyneDE]HGK	17344	1	TRUE
Insoluble	*typA*	A0A5S4TJ47	Large ribosomal subunit assembly factor	A0A5S4TJ47_STRPY	TRUE	TRUE	NVAIIAHVDHGK	243.08492	243.08492	3	NVAIIAHVD[HAyneDE]HGK	17432	1	TRUE
Insoluble	*tyrS*	A0A5S4TJM7	Tyrosine--tRNA ligase	A0A5S4TJM7_STRPY	TRUE	TRUE	IDDQLTVIR	296.05328	296.05328	3	ID[HAyneDE]DQLTVIR	38562	2	TRUE
Insoluble	*tyrS*	A0A5S4TJM7	Tyrosine--tRNA ligase	A0A5S4TJM7_STRPY	TRUE	TRUE	IDDQLTVIR	128.9248	128.9248	3	IDD[HAyneDE]QLTVIR	41456	3	TRUE
Insoluble	*xylG*	A0A5S4TFS7	ABC transporter ATP-binding protein	A0A5S4TFS7_STRPY	TRUE	TRUE	SIETVDVAGATSQDLAEMMVGR	593.46643	593.46643	3	SIETVDVAGATSQD[HAyneD	43698	2	TRUE
Insoluble	*xylG*	A0A5S4TFS7	ABC transporter ATP-binding protein	A0A5S4TFS7_STRPY	TRUE	TRUE	SIETVDVAGATSQDLAEMMVGR	593.46643	593.46643	3	SIETVDVAGATSQD[HAyneD	43731	2	TRUE
Insoluble	*xylG*	A0A5S4TFS7	ABC transporter ATP-binding protein	A0A5S4TFS7_STRPY	TRUE	TRUE	SIETVDVAGATSQDLAEMMVGR	277.26755	277.26755	3	SIETVD[HAyneDE]VAGATS(	42488	1	TRUE
Soluble	*xylM*	A0A4U9C148	4-oxalocrotonate tautomerase	A0A4U9C148_STRPY	TRUE	TRUE	PFVTIDLFEGR	322.1326	322.1326	3	[Acetyl (Protein N-term)]PF	72668	1	TRUE
Soluble	*xylM*	A0A4U9C148	4-oxalocrotonate tautomerase	A0A4U9C148_STRPY	TRUE	TRUE	PFVTIDLFEGR	154.27464	154.27464	2	PFVTID[HAyneDE]LFEGR	62350	1	TRUE
Soluble	*xylM*	A0A4U9C148	4-oxalocrotonate tautomerase	A0A4U9C148_STRPY	TRUE	TRUE	PFVTIDLFEGR	154.27287	154.27287	2	PFVTID[HAyneDE]LFEGR	62274	1	TRUE
Insoluble	*yeeN*	A0A4U7GIP0	Probable transcriptional regulatory prote	A0A4U7GIP0_STRPY	TRUE	TRUE	VYHNVADF	655.9622	655.9622	2	VYHNVAD[HAyneDE]F	35726	1	TRUE
Insoluble	*yeeN*	A0A4U7GIP0	Probable transcriptional regulatory prote	A0A4U7GIP0_STRPY	TRUE	TRUE	VYHNVADF	547.7842	547.7842	2	VYHNVAD[HAyneDE]F	35763	1	TRUE
Insoluble	*yeeN*	A0A4U7GIP0	Probable transcriptional regulatory prote	A0A4U7GIP0_STRPY	TRUE	TRUE	VYHNVADF	655.9622	655.9622	2	VYHNVAD[HAyneDE]F	35751	1	TRUE
Insoluble	*yeeN*	A0A4U7GIP0	Probable transcriptional regulatory prote	A0A4U7GIP0_STRPY	TRUE	TRUE	VYHNVADF	398.32318	398.32318	3	VYHNVAD[HAyneDE]F	35714	1	TRUE
Insoluble	*yeeN*	A0A4U7GIP0	Probable transcriptional regulatory prote	A0A4U7GIP0_STRPY	TRUE	TRUE	VYHNVADF	671.5857	671.5857	3	VYHNVAD[HAyneDE]F	35722	1	TRUE

Table S3
List of all 1,335 peptides identified in the proteomic study that contained an HAyne-labeled aspartate residue
